# OpenMedEval: Benchmarking 172 open-source LLMs on 326K medical questions across 93 datasets

**DOI:** 10.1371/journal.pone.0361996

**Published:** 2026-11-13

**Authors:** Ziqian Bi, Zhantong Ma, Junmin Huang, Junfeng Hao, Lu Chen, Hongying Luo, Zhenyu Yu, Tianyang Wang, Yue Ma, Aiping Tan

**Affiliations:** 1 Liaoning University, School of Cyber Science and Engineering, Shenyang, Liaoning, China; 2 Computer and Information Technology, Purdue University, West Lafayette, Indiana, United States of America; 3 Department of Nephrology, Affiliated Hospital of Guangdong Medical University, Zhanjiang, Guangdong, China; 4 Faculty of Computer Science and Information Technology, University of Malaya, Kuala Lumpur, Malaysia; 5 School of AI and Advanced Computing, Xi’an Jiaotong-Liverpool University, Suzhou, Jiangsu, China; 6 School of International Chinese Studies, East China Normal University, Shanghai, China; University of Basel Institute for Biomedical Ethics: Universitat Basel Institut fur Bio- und Medizinethik, SWITZERLAND

## Abstract

To address the practical challenges, including the difficulties in safeguarding medical data privacy arising from the closed-source nature of commercial large language models (LLMs), the limitations of existing benchmarks that predominantly focus on English-centric scenarios, and the inadequate consideration of deployment-related factors such as inference speed and parameter efficiency, we propose the OpenMedEval benchmark framework. This work aims to fill the gap in the systematic evaluation of open-source medical LLMs. The framework comprises 326,100 medical questions across 93 datasets, covering over 30 medical specialties and 4 professional tiers (ranging from entry-level licensing to senior specialist certification). It incorporates diverse assessment sources such as the United States Medical Licensing Examination (USMLE), nephrology cases from the American Journal of Kidney Diseases (AJKD), and Chinese Medical Licensing Examinations spanning 2017–2024. We conduct multi-dimensional evaluations on 172 open-source models with parameter scales ranging from 0.5B to 671B, integrating deployment-relevant metrics including inference speed and instruction-following compliance. Experimental results demonstrate that reasoning-enhanced architectures (e.g., DeepSeek-R1, QWQ) outperform general-purpose models by 20–40 percentage points in accuracy. Specifically, DeepSeek-R1-70B achieves an average accuracy of 81.8% across all datasets and 97% on the USMLE. Model rankings are reported as a macro-average over the 93 datasets and are shown to be robust to the aggregation scheme (Spearman ρ=0.999 against question-weighted accuracy) and to language stratification (ρ=0.9998 against a Chinese-only ranking); because 99.9% of questions are Chinese-language, overall figures are Chinese-dominant and English results are reported separately. Parameter-efficient models (e.g., Phi4-Reasoning-14B) exhibit performance comparable to ultra-large-scale general-purpose counterparts. Additionally, inference speed varies by 2000× across different models. These findings provide guidance for the selection of open-source models tailored to diverse deployment scenarios, from real-time triage to complex diagnostic support. We present such scenarios as candidate applications implied by the measured accuracy–throughput trade-off, not as demonstrated clinical readiness: performance on medical licensing examinations evidences domain knowledge and structured reasoning capacity, and any use in patient care would require prospective clinical validation, safety assessment, and regulatory approval.

## 1 Introduction

The integration of artificial intelligence into healthcare represents one of the most promising developments in modern medicine. Large language models (LLMs) have demonstrated remarkable capabilities in understanding and generating medical text, answering clinical questions, and assisting with diagnostic reasoning [1,2]. However, the deployment of LLMs in clinical settings raises critical questions about their reliability, safety, and practical utility across diverse medical domains. Recent studies have highlighted the potential of LLMs to serve as clinical decision support systems, augmenting medication safety across multiple specialties [3] and influencing diagnostic reasoning in randomized clinical trials [4].

### 1.1 Motivation and research gap

While commercial LLMs such as GPT-4 and Med-PaLM 2 have shown impressive performance on medical benchmarks, their closed-source nature poses significant barriers to healthcare deployment [5]. Patient data privacy regulations, including HIPAA compliance requirements that prohibit the use of cloud-based AI services for processing protected health information without proper safeguards [6], institutional security requirements, and the need for transparent model behavior in clinical decision support systems necessitate the use of open-source alternatives that can be deployed locally. Furthermore, the majority of existing medical LLM benchmarks focus on English-language assessments [7,8], leaving a significant gap in understanding model performance on non-English medical content, which limits access to AI-assisted healthcare for non-English-speaking populations [9,10].

The medical AI community faces three critical challenges that motivate our work. First, despite the proliferation of open-source LLMs, systematic comparisons across diverse medical domains remain scarce. Existing studies typically evaluate fewer than 20 models on limited datasets, making it difficult to identify optimal models for specific clinical applications. Second, most benchmarks focus exclusively on English content, leaving a critical gap for healthcare systems serving non-English-speaking populations—particularly Chinese-speaking populations—where model performance may differ substantially from English-language assessments. Third, academic benchmarks rarely consider practical deployment factors such as inference speed, memory requirements, and cost efficiency. For resource-constrained healthcare settings, these operational considerations may be as important as raw accuracy in determining the feasibility of AI deployment.

### 1.2 Broader significance

Beyond technical contributions, this work addresses several critical issues facing medical AI deployment. **First, medical data privacy and sovereignty:** Healthcare data represents the most sensitive category of personal information, subject to stringent regulations including HIPAA in the United States, GDPR in Europe, and comparable frameworks globally [6]. Proprietary cloud-based AI services fundamentally conflict with these requirements: patient data must be transmitted to external servers, fine-tuning data becomes accessible to service providers, and institutions lose control over how their data is used for model improvement. Open-source models eliminate these concerns entirely—institutions can deploy models on-premises where patient data never leaves institutional firewalls, fine-tune on sensitive clinical records without any data leaving the local environment, and maintain complete sovereignty over their medical AI systems. This is not merely a compliance advantage but a fundamental prerequisite for responsible medical AI deployment. Given the average healthcare data breach cost of $9.77 million and increasing regulatory scrutiny, the ability to operate entirely locally represents a decisive advantage of open-source alternatives [6].

**Second, medical examination integrity:** The demonstrated capability of LLMs to achieve near-expert performance on medical licensing examinations raises profound questions about examination security [11,12]. Our comprehensive evaluation across multiple examination types and difficulty levels provides essential data for medical education authorities to develop AI-resistant assessment strategies and ensure the continued validity of professional credentialing. **Third, clinical decision support:** Our findings on reasoning-enhanced models have direct implications for clinical decision support system design. Research has shown that structured reasoning prompts can enhance diagnostic accuracy [13,14], and our comparative analysis identifies which open-source models best support such approaches, enabling healthcare institutions to select appropriate models for clinical deployment. **Fourth, multi-model collaboration:** Recent research demonstrates that ensemble approaches combining multiple LLMs can outperform individual models in medical question answering [15–17]. Our comprehensive evaluation provides the foundation for designing optimal model combinations, as effective ensemble systems require understanding the complementary strengths and weaknesses of constituent models. **Fifth, domain-specific fine-tuning:**Open-source models allow institutions to fine-tune on proprietary clinical resources (e.g., electronic health records, institutional guidelines, local formularies, and regional disease patterns) while keeping sensitive data in-house and out of reach of third parties. This capability is essential for adapting general medical AI to institution-specific practices while maintaining complete data confidentiality.

### 1.3 Contributions

To address these gaps, we present **OpenMedEval**, the largest comprehensive benchmark study of open-source LLMs for medical applications. In terms of scale, we evaluate 172 open-source models spanning 0.5B to 671B parameters on 326,100 medical questions across 93 datasets, representing the largest such evaluation to date for open-source medical LLMs. Our evaluated models include general-purpose architectures such as Llama [18,19], Qwen [20–22], Mistral [23,24], Phi [25,26], and Gemma [27,28], as well as reasoning-enhanced models including DeepSeek-R1 [29], QWQ [30], Phi4-Reasoning, ExaOne-Deep [31], Magistral [32], and OpenThinker [33]. Our benchmark encompasses substantial diversity, including English assessments based on USMLE [34] and AJKD nephrology cases, as well as Chinese medical examinations [35,36] across four professional levels—Medical Licensing, Attending Physician, Associate Chief Physician, and Chief Physician—covering more than 30 medical specialties. Beyond accuracy metrics, we provide practical deployment insights through systematic analysis of speed-accuracy trade-offs using vLLM [37] on standardized hardware, enabling healthcare institutions to select models based on specific requirements ranging from real-time triage applications to complex diagnostic support. Our architectural analysis demonstrates that reasoning-enhanced architectures consistently outperform general-purpose models by 20–40 percentage points, establishing reasoning-specific training as essential for medical AI applications.

## 2 Related work

### 2.1 Medical question answering benchmarks

The evaluation of language models on medical knowledge has evolved substantially over the past decade. Early benchmarks such as MedQA [34] introduced multiple-choice questions derived from medical licensing examinations, establishing a foundation for assessing clinical knowledge. The MultiMedQA benchmark [1] expanded this scope to include consumer health questions, research queries, and professional medical examinations across multiple formats.

Med-PaLM [1] represented a landmark study demonstrating that large language models could approach expert-level performance on medical question answering. Med-PaLM 2 further advanced this capability, achieving 86.5% on MedQA and receiving favorable physician evaluations on multiple clinical utility axes. However, these studies focused primarily on proprietary models, limiting their applicability to settings requiring open-source solutions.

Recent benchmarks have expanded beyond English-language assessments. CMExam [35] introduced Chinese medical licensing examination questions, while MedBench [36] provided comprehensive Chinese medical evaluation. Specialized benchmarks have also emerged for Japanese, Korean, and other languages [9]. Our work builds upon these foundations by providing the most comprehensive bilingual (Chinese-English) evaluation of open-source models to date.

### 2.2 Open-source medical LLMs

The open-source LLM ecosystem has experienced rapid growth, with models from diverse organizations achieving competitive performance with commercial alternatives. The Llama family [18,19] from Meta AI established foundational capabilities that have been extended through fine-tuning for medical applications. Qwen [20–22] and DeepSeek [38,39] have demonstrated particular strength in bilingual (English/Chinese) settings.

Domain-specific medical models have emerged through continued pre-training and fine-tuning approaches. MEDITRON [40] applied medical corpus pre-training to Llama foundations, while BioMistral [41] and OpenBioLLM [42] targeted biomedical text understanding. However, these specialized models have not consistently outperformed general-purpose alternatives on downstream medical tasks, raising questions about the optimal approach to medical LLM development.

### 2.3 Reasoning-enhanced architectures

A paradigm shift occurred with the introduction of reasoning-enhanced LLMs that generate explicit chains of thought during inference. DeepSeek-R1 [29] demonstrated that reinforcement learning on reasoning traces could produce models competitive with OpenAI o1 across mathematical and scientific domains. QWQ-32B [30], Phi-4-Reasoning [26], and OpenThinker [33] extended these capabilities to smaller parameter regimes.

The application of reasoning-enhanced models to medical domains remains underexplored. While general reasoning benchmarks have been extensively studied, systematic evaluation across diverse medical specialties and difficulty levels has been lacking. Our work addresses this gap by providing the first large-scale comparison of reasoning-enhanced versus general-purpose architectures on medical benchmarks.

### 2.4 Deployment considerations for medical AI

Practical deployment of medical AI systems requires consideration of factors beyond raw accuracy. Inference speed determines feasibility for real-time clinical decision support, while memory requirements constrain deployment options in resource-limited settings. The TRIPOD-LLM guidelines [43] emphasize the importance of reporting computational requirements alongside performance metrics.

Mixture-of-Experts (MoE) architectures have emerged as a promising approach for balancing capability and efficiency [44]. Models such as Mixtral [24] and DeepSeek-V3 activate only a subset of parameters per token, reducing computational costs while maintaining performance. Our evaluation includes systematic speed measurements to enable informed deployment decisions.

## 3 Methods

### 3.1 Benchmark dataset construction

We construct a comprehensive medical benchmark comprising 93 datasets with 326,100 multiple-choice questions spanning diverse medical domains and difficulty levels. Table 1 summarizes the dataset composition by examination category.

**Table 1:** Overview of OpenMedEval benchmark datasets across six examination categories with varying difficulty levels.

Category	Datasets	Samples	Avg. Tokens
Medical Licensing Exam (MLE)	8	4,296	156
Attending Physician (AP)	22	114,327	189
Associate Chief Physician (ACP)	28	118,798	234
Chief Physician (CP)	32	79,896	278
International (USMLE, AJKD)	2	453	312
Health Classification	1	8,330	89
**Total**	**93**	**326,100**	**221**

Table 1 summarizes the composition of the OpenMedEval benchmark across six categories with increasing levels of difficulty. The benchmark includes Chinese medical licensing examinations spanning four professional tiers (MLE, AP, ACP, CP), international medical examinations (USMLE, AJKD), and a health information classification task designed to evaluate robustness to real-world medical misinformation.

In terms of language composition, Chinese-language examinations (MLE, AP, ACP, and CP tiers combined with the health classification dataset) account for 325,647 questions (99.9% of the total), while English-language content (USMLE and AJKD) comprises 453 questions (0.1%). This distribution reflects the study’s primary focus on Chinese medical credentialing contexts; English assessments provide internationally recognized comparison benchmarks. Accordingly, throughout this paper we use the term *bilingual (Chinese-English)* rather than “multilingual” to accurately characterize the linguistic scope of our evaluation.

#### 3.1.1 International medical examinations.

**USMLE.** The United States Medical Licensing Examination dataset [34] assesses comprehensive medical knowledge required for physician licensure in the United States, covering basic sciences, clinical knowledge, and patient care domains. The MedQA dataset [34] contains 12,723 questions from USMLE with a human passing threshold of approximately 60%.

**AJKD.** Questions from the American Journal of Kidney Diseases evaluate specialized nephrology knowledge, representing domain-specific expertise beyond general medical training. These questions are derived from Core Curriculum articles and provide comprehensive assessment of nephrology competency [5].

#### 3.1.2 Health information classification.

**Health classification dataset.** This dataset comprises 8,330 health-related claims collected from online sources including social media, health magazines, and popular science websites—channels frequently accessed by middle-aged and elderly populations. The task is binary classification: distinguishing between scientifically accurate health information (positive, 3,235 samples) and medical misinformation or health fraud (negative, 5,095 samples). This dataset addresses the critical public health challenge of combating health misinformation that proliferates through informal channels, testing models’ ability to identify misleading health claims that could harm vulnerable populations.

#### 3.1.3 Chinese medical licensing examinations.

We include physician qualification examinations from 2017–2024 across four professional levels representing the Chinese medical credentialing hierarchy, building upon established Chinese medical benchmarks [35,36]. The Medical Licensing Examination (MLE) serves as the entry-level certification required for medical practice, covering fundamental clinical knowledge across all major disciplines. The Attending Physician (AP) examinations represent specialist qualifications across 22 distinct medical specialties, testing intermediate-level clinical expertise. The Associate Chief Physician (ACP) tier provides senior specialist certification covering 28 subspecialties with advanced clinical scenarios requiring sophisticated diagnostic reasoning. At the apex, the Chief Physician (CP) examinations represent the highest clinical grade, spanning 32 specialties with complex multi-step diagnostic reasoning and treatment planning challenges.

The examinations collectively cover more than 30 medical specialties including Internal Medicine, Surgery, Pediatrics, Obstetrics and Gynecology, Neurology, Oncology, Psychiatry, Emergency Medicine, Radiology, Pathology, and Clinical Laboratory Medicine, providing comprehensive coverage of the clinical knowledge spectrum.

#### 3.1.4 Dataset sources and quality assurance.

**Data sources and licensing.** Our benchmark integrates publicly available medical examination datasets with appropriate usage rights. The USMLE questions are derived from the MedQA dataset [34], released under research-use terms. AJKD nephrology cases are compiled from published Core Curriculum articles in the American Journal of Kidney Diseases [5]. Chinese medical examination questions are sourced from publicly released practice materials and prior examination archives that have entered the public domain following official publication by examination authorities, consistent with standard practice in Chinese medical AI benchmarking [35,36]. All datasets are used solely for non-commercial research purposes.

**Quality assurance.** Our benchmark construction prioritizes data quality through several rigorous measures. First, all questions originate from *official* medical licensing examinations administered by national medical authorities, ensuring content validity and clinical relevance verified by domain experts. Unlike synthetically generated or crowd-sourced questions that may contain errors or ambiguities, official examination questions undergo extensive review by medical professionals before deployment. Second, each question includes verified ground-truth answers from official examination keys, eliminating annotation noise that affects many medical NLP datasets [45]. Third, we include temporal diversity spanning 2017–2024 examinations, enabling analysis of model performance across question cohorts while ensuring currency of medical knowledge. Fourth, the hierarchical structure across four professional levels (MLE, AP, ACP, CP) provides graduated difficulty assessment, distinguishing models that merely memorize facts from those capable of sophisticated clinical reasoning [35]. Finally, our dataset size (326,100 questions) substantially exceeds existing Chinese medical benchmarks including CMExam (60K questions) [35] and MedBench (40K questions) [36], enabling more reliable performance estimation with reduced statistical variance.

### 3.2 Model selection

We evaluate 172 open-source large language models spanning multiple architectural paradigms and parameter scales from 0.5B to 671B parameters. Our selection criteria emphasize models with open weights enabling local deployment for healthcare applications.

#### 3.2.1 General-purpose models.

**Llama family.** Meta AI’s flagship open-source LLM family [18,19,46], including Llama 2 (7B–70B), Llama 3 (8B–70B), Llama 3.1 (8B–405B), and Llama 4 with MoE architecture.

**Qwen family.** Alibaba’s bilingual models [20–22] optimized for English/Chinese, from Qwen2.5 (0.5B–72B) to Qwen3 (0.6B–235B MoE).

**Mistral family.** Mistral AI models [23,24] including Mistral 7B, Mixtral 8x7B/8x22B MoE, and Mistral Large (123B).

**Phi family.** Microsoft’s parameter-efficient models [25,26] demonstrating that high-quality training data can compensate for smaller scale (3.8B–14B).

**Gemma family.** Google’s lightweight open models [27,28] from Gemini research (2B–27B).

**GPT-OSS family.** OpenAI’s first open-weight model family released in 2025 [47], including GPT-OSS-20B and GPT-OSS-120B variants. These models represent OpenAI’s entry into the open-source ecosystem, providing competitive performance with full weight access for local deployment.

#### 3.2.2 Reasoning-enhanced models.

**DeepSeek-R1.** Reasoning-specialized models [29] trained with reinforcement learning on explicit reasoning chains, ranging from 1.5B to 671B parameters. Recent evaluations in Nature Medicine demonstrate performance comparable to proprietary models on clinical decision-making tasks [48].

**QWQ-32B.** Alibaba’s 32B reasoning model [30] based on Qwen2.5-32B [21], trained with reinforcement learning using outcome-based rewards. QWQ achieves competitive performance with DeepSeek-R1 despite significantly fewer parameters.

**Phi4-reasoning.** Microsoft’s reasoning-enhanced Phi-4 variants [26] (3.8B, 14B) achieving performance comparable to much larger models on reasoning benchmarks through high-quality synthetic training data.

**OpenThinker.** Community-developed reasoning models [33] (7B, 32B) fine-tuned on the OpenThoughts dataset, achieving strong performance on mathematical and scientific reasoning tasks.

**ExaOne-deep.** LG AI Research’s reasoning-specialized family [31] (2.4B–32B) trained on reasoning-specialized datasets incorporating long streams of thought processes, with the 7.8B variant outperforming models of comparable scale including OpenAI o1-mini [49].

**Magistral.** Mistral AI’s first reasoning model family [32] trained with reinforcement learning on verified reasoning traces, available in 24B (Apache 2.0 license) and larger variants with support for dozens of languages.

Complete specifications for all 172 evaluated models are provided in S1 File (Appendix A, Table S23).

### 3.3 Evaluation protocol

#### 3.3.1 Inference configuration.

All models are evaluated using standardized 5-shot prompting with chain-of-thought reasoning [50]. The five demonstration examples are fixed across all models and datasets, selected from a held-out set of questions that does not overlap with any test split. Selection criteria balanced three factors: (1) *specialty diversity*—examples span five distinct medical disciplines (internal medicine, surgery, pediatrics, obstetrics, and neurology) to expose models to varied clinical reasoning contexts; (2) *difficulty range*—examples include both straightforward recall questions and multi-step reasoning scenarios to elicit chain-of-thought behavior across difficulty levels; (3) *format fidelity*—each example demonstrates the required output format (single letter A–E) followed by a concise reasoning explanation. The same English prompt template is used for USMLE/AJKD, while a parallel Chinese template with equivalent structure is used for Chinese examinations (full templates provided in S1 File, Appendix C).

To assess sensitivity to prompt selection, we conducted a validation experiment on a representative subset of 20 models (spanning parameter scales from 7B to 70B and covering both general-purpose and reasoning-enhanced architectures), comparing performance under three prompt conditions: 0-shot, an alternative 5-shot set with different example questions, and our standard 5-shot set. The Pearson correlation of per-model accuracy between the two 5-shot conditions was *r* = 0.997, and between 5-shot and 0-shot was *r* = 0.991, confirming that model rankings are robust to prompt variation. We therefore report results using the fixed standard 5-shot template throughout.

We employ greedy decoding (temperature = 0, top_p = 1.0, top_k = −1) to ensure reproducibility and eliminate sampling variance. Maximum generation length is set to 2048 tokens to accommodate extended reasoning chains characteristic of reasoning-enhanced models.

Model inference is performed using vLLM [37] v0.6.x with default configurations, an open-source library that achieves high-throughput serving through paged attention memory management and continuous batching. All models are evaluated in their original precision as released—bfloat16 or float16, or FP8 where FP8 is the native released precision, as for the DeepSeek-R1 and DeepSeek-V3 family—and we applied no quantization of our own at any point, so that no model is scored on weights other than those it was released with.

**Hardware and serving topology.** Inference ran on NVIDIA H100 80GB SXM GPUs with NVLink interconnect, organized in nodes of eight devices, giving 640 GB of aggregate device memory per node, and covering the full evaluated range from 0.5B to 671B parameters. Serving topology followed one rule based on the memory a model’s weights require at their released precision, computed as parameter count × bytes per parameter. Models whose weights fit a single device were served with tp = 1; this covers up to roughly the 33B class (66 GB at bfloat16). Models whose weights fit one node but not one device were served on a single node with tensor parallelism across its eight devices; this covers the 70B class (140 GB) through the 236B class (472 GB, or 74% of node capacity). Models whose weights exceed one node’s 640 GB were served across multiple nodes, with tensor parallelism within each node and pipeline parallelism across nodes. The multi-node group comprises the 400–480B class (Llama-4 Maverick, 400B total; Llama-3.1-405B; Qwen3-Coder-480B; 800–960 GB at bfloat16) and the 671B class (DeepSeek-R1, DeepSeek-V3, DeepSeek-V3.1; 671 GB at their native FP8, 1,342 GB at bfloat16), each requiring at least two nodes.

These memory figures are analytical weight-memory requirements derived from the parameter counts listed in S1 File, Appendix A (Table S23), not measured resident footprints. A model’s actual resident memory additionally includes KV-cache and activation memory, which scale with the concurrent request batch; we did not tabulate these per model. Under the fixed benchmark workload all allocated devices operated at full utilization for the duration of a model’s run, since continuous batching keeps the served batch saturated until the question queue is exhausted. We also note that a model served across multiple nodes incurs inter-node communication overhead that a single-node model does not, so the relative throughput figures reported below are not strictly topology-matched for the largest models; we treat this explicitly as a limitation (Section 5.8).

**Computational resources.** Complete evaluation of all 172 models on 326,100 questions required approximately 2,400 GPU-hours on H100 hardware. The majority of compute time was consumed by large reasoning models (671B, 405B) due to extended reasoning chain generation. Smaller models (<14B) complete the full benchmark in under 5 GPU-hours each. Total estimated cloud compute cost at current rates would be approximately $10,000–15,000 USD, though our on-premises infrastructure eliminated direct cloud costs.

#### 3.3.2 Evaluation agent architecture.

We develop a specialized evaluation agent that enforces structured output formatting, requiring models to respond with only the selected option letter (A, B, C, D, or E). This design serves dual purposes: (1) it provides a rigorous test of instruction-following capability—a critical requirement for clinical deployment where models must adhere to specified output formats; and (2) it enables unambiguous accuracy measurement without reliance on heuristic answer extraction from free-form responses.

**Handling format non-compliance.** When models fail to output a valid single-letter response (A–E), we apply a deterministic, model-agnostic extraction procedure: (1) search the response for explicit answer patterns (e.g., “The answer is B”, “Answer: C”); (2) if no pattern is found, extract the first occurrence of a valid option letter; (3) if no valid letter is found, the response is marked as incorrect. The same ordered rules are applied to every model without exception, so no model benefits from a bespoke parser. Two cases that arise frequently with reasoning-enhanced models are handled explicitly. When a response contains *multiple* option letters—for example when a model enumerates and rejects distractors before committing—rule (1) takes precedence, so a letter attached to an explicit final-answer pattern is used in preference to any letter appearing earlier in the reasoning trace; only when no such pattern exists does rule (2) fall back to the first valid letter, which for a model that deliberates and then commits is the letter introducing its deliberation. When a response consists of *extended reasoning* that is cut off by the generation cap before any answer is emitted, no valid letter is present and rule (3) scores the item as incorrect; truncated reasoning is thus never resolved in the model’s favour. This conservative approach ensures that format non-compliance is penalized rather than artificially resolved. Table 5 reports compliance rates across model categories, which quantify how often a response left the directly scorable single-letter path and had to be resolved by the fallback rules; because the rules are identical for all models, any residual advantage from output style is bounded by these rates rather than being a free parameter of the protocol.

In addition to the constrained option-only responses used for accuracy scoring, we preserve the complete model outputs including full reasoning traces. This dual-track approach enables comprehensive post-hoc analysis: the constrained responses provide clean accuracy metrics, while the full responses support qualitative error analysis and reasoning chain examination.

#### 3.3.3 LLM-as-judge error analysis.

To characterize reasoning failures and identify patterns in incorrect responses, we employ frontier models as automated judges for qualitative analysis. Specifically, we utilize three independent judges—Qwen3-235B [22], GPT-OSS-120B, and a third comparable state-of-the-art model—to analyze the complete reasoning traces of incorrectly answered questions. Each judge assigns an incorrect response to one of four predefined categories, and a category is recorded only when *at least two of the three judges agree*, so that no classification rests on a single model’s idiosyncratic reading. This LLM-as-judge framework [51] enables scalable identification of common error patterns including: (1) factual knowledge gaps where models lack necessary medical information; (2) reasoning chain failures where correct knowledge is incorrectly applied; (3) instruction misinterpretation where models misunderstand question requirements; and (4) hallucination patterns where models generate plausible but incorrect medical claims.

**Human validation.** To quantify judge reliability rather than assume it, a random sample of 100 error cases was independently reviewed by two co-authors with clinical backgrounds, and the automated classifications agreed with the human reviewers in 87% of cases. This agreement rate is reported as an upper-bound indication of the taxonomy’s reliability, not as a certification of it: the residual 13% disagreement, together with the judge-bias concern analysed in Section 5.8, means that the category proportions in Table 6 should be read as an *exploratory* characterization of failure modes at scale rather than as definitive, expert-adjudicated estimates. We accordingly use the taxonomy to describe qualitative differences between architectural families—for which a systematic judge bias would have to differ between families to distort the comparison—and not to support any claim about the absolute prevalence of a failure mode in clinical use.

#### 3.3.4 Protocol uniformity across heterogeneous models.

The 172 evaluated models differ in architecture, training methodology, native chat template, reasoning behaviour, and output conventions, so we state explicitly which aspects of the protocol were held identical and which were allowed to follow each model’s own conventions. Held *identical* for every model: the substantive content and ordering of the five demonstrations, the question and option rendering, the instruction to reason and then emit a single letter, greedy decoding (temperature = 0, top_*p* = 1.0, top_k=−1), the 2,048-token generation cap, the serving stack and its default configuration, the original released precision (no quantization), and the fixed hardware. Allowed to follow each model’s own conventions: the surface chat templating through which that identical content was delivered, applied by the serving stack’s native template for each model so that every model received the demonstrations in the form it was trained on; forcing a single foreign template on all models would penalize models for a formatting mismatch unrelated to medical capability. Parallelism followed model size under the single rule stated in the Hardware and serving topology paragraph—tp = 1 where the weights fit one device, single-node tensor parallelism where they fit one node, and tensor parallelism within nodes plus pipeline parallelism across nodes where they do not—with no per-model tuning, warm-up sweep, or batch-size search. The single generation cap is applied uniformly, including to reasoning-enhanced models that would naturally emit longer traces; the consequences of that choice are not hidden but bounded, because a response truncated before it emits an answer is scored incorrect by extraction rule (3), and the rates at which models leave the directly scorable path are reported in Table 5. Weight-memory requirements and device utilization are reported in the Hardware and serving topology paragraph above; what we did not collect are per-model truncation counts and per-model KV-cache and activation footprints, and we list those as a limitation in Section 5.8 together with the deviation-free statement above, so that readers can see precisely which reproducibility details are and are not available.

#### 3.3.5 Performance metrics.

**Accuracy.** The primary metric is accuracy, defined as the percentage of correctly answered multiple-choice questions. For questions with multiple correct answers, partial credit is not awarded. This evaluation protocol aligns with established medical benchmarks including MedQA [34], CMExam [35], and MedBench [36].

**Inference speed.** Speed is measured on standardized NVIDIA H100 80GB hardware in eight-device nodes using vLLM [37] with default serving settings, continuous batching, and paged-attention memory management. For each model we record the total wall-clock time required to complete one identical, fixed workload—the full 326,100-question benchmark under the same 5-shot prompts, greedy decoding, and 2,048-token generation cap—and define relative speed as the ratio of the baseline model’s completion time to that of the target model, so that DeepSeek-R1-70B, the slowest model in the suite, is the baseline at 1.0×. Relative speed is therefore an *aggregate end-to-end serving throughput* measure (questions completed per unit wall-clock time on this hardware class); it is not a per-token generation rate, not a prefill-only measure, and not a single-request latency. Because reasoning-enhanced models emit substantially longer outputs, completion time conflates output length with decoding speed, and the metric should be read as a relative indication of serving cost rather than as a predicted clinical response time. Timings were available for 140 of the 172 models; the remaining models are reported for accuracy only and are excluded from the speed figures. All timings were taken under the same serving stack, prompts, decoding settings, generation cap, and hardware class, without per-model tuning, warm-up sweeps, or batch-size search, so no model receives a tuned serving advantage; the only variation is the parallel topology, which follows model size under the single rule stated in the Hardware and serving topology paragraph. Weight-memory requirements for each size class and device utilization are reported there. What we did not separately tabulate are per-model truncation incidence and KV-cache footprint, which we identify as a limitation and a target for future reporting (Section 5.8). These measurements provide comparative guidance for healthcare institutions evaluating deployment options [52].

#### 3.3.6 Statistical analysis.

**Aggregation schemes.** Because the 93 datasets differ in size by more than two orders of magnitude (from 453 international questions to 118,798 Associate Chief Physician questions; Table 1), the aggregation rule materially affects interpretation, and we therefore keep two distinct quantities separate throughout this paper.

The *macro-averaged accuracy* is the unweighted mean of the 93 per-dataset accuracies,


$$\begin{array}{c} {Acc}_{macro}\;=\;\frac{1}{93}\sum_{d=1}^{93}{acc}_{d}, \end{array}$$ (1)

which weights every dataset equally irrespective of its question count and is the quantity used for all model rankings reported here. The *question-weighted accuracy* first averages datasets within each of the six examination categories and then combines categories in proportion to their question counts $N_{c}$ in Table 1,


$$\begin{array}{c} {Acc}_{qw}\;=\;\frac{\sum_{c}N_{c}\;{\overset{―}{acc}}_{c}}{\sum_{c}N_{c}},\;\sum_{c}N_{c}=326,100, \end{array}$$ (2)

which reflects the accuracy expected over individual questions. Every model answered every one of the 326,100 questions, so the 93×172 accuracy matrix is complete and both quantities are defined for all 172 models without imputation.

The two schemes agree closely. Across all 172 models the Spearman rank correlation between them is ρ=0.999; the mean absolute difference is 0.75 percentage points (maximum 2.43); and the ten leading models are identical in both membership and order, with DeepSeek-R1-70B ranked first under either rule. The rankings reported in this paper are therefore not an artefact of the aggregation choice. Both quantities are reported side by side for the leading models in Table 3.

**Language-stratified reporting.** Since 325,647 of the 326,100 questions (99.9%) are Chinese-language and 453 (0.1%) are English-language, pooled figures are necessarily dominated by the Chinese datasets, and we therefore report the two languages separately rather than relying on the pooled figure alone. The English panel comprises the two English datasets (USMLE and AJKD). The Chinese panel comprises the 90 Chinese *examination* datasets (MLE, AP, ACP and CP tiers); the Chinese-language health-claims classification task is held out of that panel and reported separately, because it is a binary misinformation-detection task rather than a medical licensing examination and would otherwise mix two different task types inside a single “Chinese” score. Both panels are reported for the leading models in Table 3 and Section 4.2.4.

This stratification does not change any conclusion drawn from the pooled ranking. The Chinese-examination ranking of the ten leading models is identical to the overall 93-dataset ranking in both membership and order, and the two rankings agree across all 172 models (Spearman ρ=0.9998); the overall leaderboard can therefore be read as a Chinese-dominant ranking without ambiguity, and we say so explicitly wherever it is used. The English panel spans only two datasets and is correspondingly coarse, and it does *not* reproduce the overall ranking (Spearman ρ=0.857 against the overall ranking across all 172 models). We therefore present the English results as a separate reference panel and deliberately do not use them to rank models overall, since a two-dataset panel cannot support a 172-model ordering.

**Uncertainty.** Two different standard errors are relevant to this benchmark and we do not conflate them. At the *question level*, the binomial standard error of a pooled accuracy estimated from *N* = 326,100 questions is $\sqrt{p(1-p)/N}$, which attains its maximum of 0.088 percentage points at *p* = 0.5 and remains below 0.1 percentage points for every evaluated model; this is the sense in which our accuracy estimates are precise, and it is the quantity referred to when we note that per-question standard errors are well below 0.5%. At the *dataset level*, the uncertainty that governs model-to-model ranking is the standard error of the macro-average, $SD(\{{acc}_{d}\})/\sqrt{93}$, which is necessarily larger because it also absorbs genuine between-dataset variation in difficulty and specialty. Across the 172 models this dataset-level standard error has a median of 1.08, a minimum of 0.20 and a maximum of 1.52 percentage points. We report dataset-level standard deviations, standard errors and 95% confidence intervals for the leading models in Table 2, and we use them, rather than question-level errors, whenever two models are compared.

**Table 2:** Top 10 performing models ranked by macro-averaged accuracy across all 93 medical benchmark datasets. Accuracy is the unweighted mean over the 93 per-dataset accuracies. SD and SE are computed across the 93 datasets, and the 95% confidence interval is the macro-average ±1.96SE; these dataset-level quantities, not question-level binomial errors, are the relevant uncertainty for comparing two models (see Statistical Analysis).

Rank	Model	Params	Acc.	SD	SE	95% CI
1	DeepSeek-R1-70B	70B	81.8	5.48	0.57	80.7–82.9
2	DeepSeek-R1-32B	32B	79.0	6.24	0.65	77.7–80.2
3	QWQ-32B	32B	77.8	8.38	0.87	76.1–79.6
4	DeepSeek-R1-14B	14B	76.1	7.47	0.77	74.6–77.6
5	DeepSeek-R1-671B	671B	76.0	10.63	1.10	73.8–78.2
6	Phi4-Reasoning-14B	14B	73.1	10.28	1.07	71.0–75.2
7	DeepSeek-R1-8B	8B	69.5	9.01	0.93	67.7–71.3
8	Magistral-24B	24B	67.9	11.49	1.19	65.6–70.3
9	ExaOne-Deep-32B	32B	66.8	8.02	0.83	65.1–68.4
10	OpenThinker-7B	7B	63.5	10.64	1.10	61.3–65.6

**Model comparisons.** Because all models are evaluated on the identical set of 93 datasets, every model-to-model comparison is paired by dataset. We quantify differences by the paired mean difference across datasets with its 95% confidence interval, supported by a paired *t*-test and a distribution-free Wilcoxon signed-rank test, and we additionally report the number of datasets favouring each model so that a difference driven by a subset of datasets is visible rather than hidden. Differences are described as statistically distinguishable only where these paired tests support them; otherwise we report the numerical ordering alone. For model family comparisons, we aggregate performance across all variants within each family. Performance distributions are visualized through box plots and violin plots to characterize variance.

### 3.4 Ethical considerations

This study evaluates AI models on medical examination questions and does not involve human subjects, patient data, or clinical interventions. All benchmark datasets are derived from publicly administered professional licensing examinations that do not contain personally identifiable information. Our evaluation methodology uses deterministic inference without model fine-tuning, ensuring reproducibility and eliminating concerns about data contamination from our experimental procedures.

We acknowledge the dual-use implications of our findings: while our benchmark enables healthcare institutions to select appropriate AI models for clinical decision support, the demonstrated high performance of LLMs on medical licensing examinations raises concerns about examination integrity that we discuss explicitly. We encourage medical education authorities to consider our findings when designing AI-resistant assessment strategies.

The open-source models evaluated in this study enable local deployment that preserves patient privacy—a critical ethical consideration for healthcare AI. However, we emphasize that strong benchmark performance does not constitute clinical validation, and any deployment of AI in patient care settings requires rigorous safety assessment, regulatory approval, and appropriate human oversight.

## 4 Results

### 4.1 Overall model performance

Figure 1 illustrates the OpenMedEval evaluation framework, showing the three assessment dimensions (accuracy, inference speed, and parameter efficiency) applied across all 172 models.

**Fig 1:**
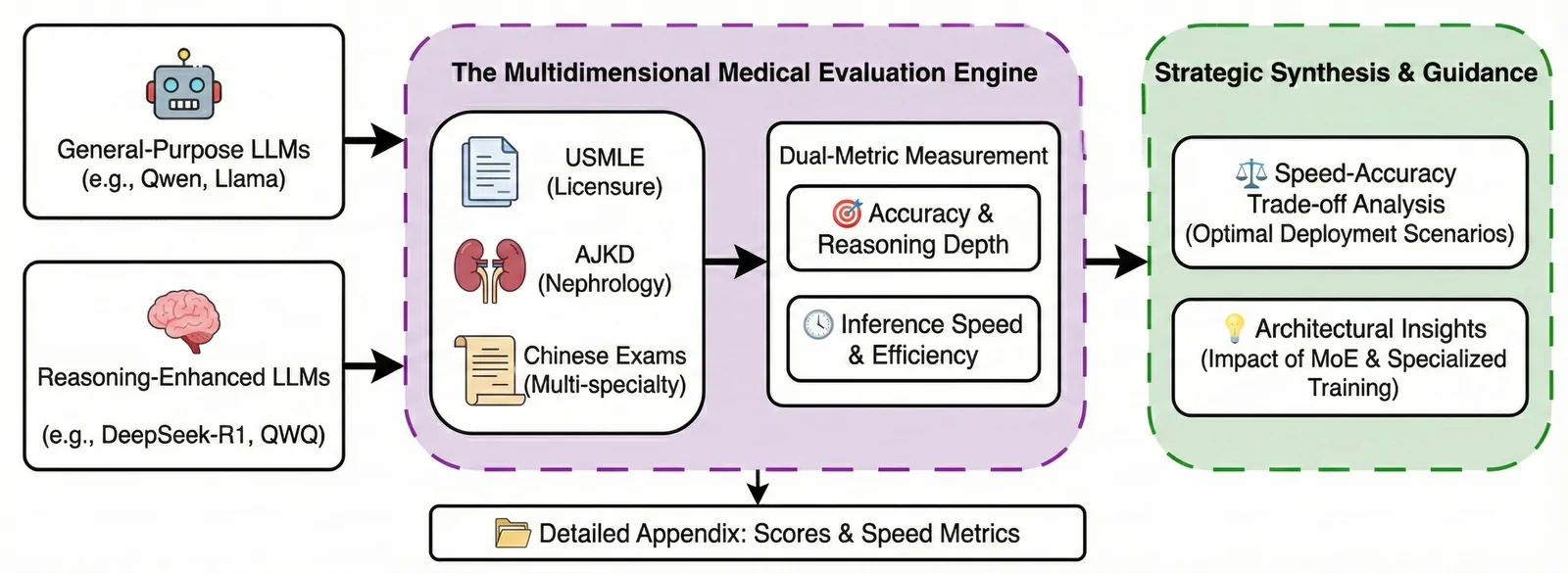
Conceptual overview of the OpenMedEval multidimensional medical evaluation framework (illustration created with AI assistance and manually refined). The framework systematically evaluates 172 open-source large language models across three evaluation dimensions: accuracy on 93 medical datasets spanning USMLE, AJKD nephrology cases, and Chinese physician licensing examinations at four professional levels; inference speed measured on standardized hardware to enable practical deployment decisions; and parameter efficiency analyzing the trade-off between model size and performance. Models are categorized into general-purpose architectures (Qwen, Llama, Mistral, Gemma, Phi) and reasoning-enhanced architectures (DeepSeek-R1, QWQ, Phi4-Reasoning, ExaOne-Deep, Magistral) to enable systematic comparison of training methodologies.

Table 2 presents the top-performing models ranked by average accuracy across all 93 datasets. DeepSeek-R1-70B achieves the highest overall accuracy of 81.8%, followed by DeepSeek-R1-32B (79.0%) and QWQ-32B (77.8%). Notably, reasoning-enhanced models occupy all top-10 positions, demonstrating the critical importance of explicit reasoning capabilities for medical question answering.

Because the 93 datasets differ in size by more than two orders of magnitude, and because 99.9% of questions are Chinese-language, Table 3 reports the leading models under both aggregation schemes and both language panels defined in the Statistical Analysis subsection. The ranking is stable across all four views: the macro-averaged and question-weighted orderings are identical in membership and order for these ten models (Spearman ρ=0.999 across all 172 models; mean absolute difference 0.75 percentage points, maximum 2.43), and the Chinese-examination panel reproduces the overall ordering exactly (Spearman ρ=0.9998). The two-dataset English panel is reported for reference and, as expected from a panel of that size, does not reproduce the overall ordering (Spearman ρ=0.857); we therefore do not use it to rank models.

**Table 3:** Leading models under both aggregation schemes and both language panels. “Macro” is the unweighted mean over all 93 datasets and is the ranking metric used throughout this paper; “Q-weighted” weights the six examination categories by their question counts in Table 1; “CN exam” is the mean over the 90 Chinese licensing and specialty examination datasets; USMLE and AJKD are the two English datasets, shown separately because a two-dataset panel cannot support a 172-model ranking. All values are percentages.

Model	Macro	Q-weighted	CN exam	USMLE	AJKD
DeepSeek-R1-70B	81.80	81.65	81.48	95.50	82.28
DeepSeek-R1-32B	78.96	78.96	79.02	77.27	55.70
QWQ-32B	77.84	78.57	78.07	82.09	32.91
DeepSeek-R1-14B	76.10	76.75	76.33	69.52	40.51
DeepSeek-R1-671B	76.00	76.53	75.48	97.00	77.22
Phi4-Reasoning-14B	73.12	72.95	72.50	95.50	82.28
DeepSeek-R1-8B	69.50	70.25	69.75	54.55	34.18
Magistral-24B	67.94	68.27	67.29	89.00	75.95
ExaOne-Deep-32B	66.76	66.94	66.15	85.00	74.68
OpenThinker-7B	63.47	64.22	63.87	56.68	25.32

The 70B variant of DeepSeek-R1 outperforms the 671B variant (81.8% vs. 76.0%). Both models were evaluated using identical prompting protocols, and the 671B model used full-precision inference with appropriate tensor parallelism (no quantization), so the difference is not attributable to a serving or precision artefact. We nonetheless report it with explicit uncertainty rather than as a bare ordering. Paired across the 93 datasets, the mean difference is 5.81 percentage points (SE 0.92; 95% CI 4.01–7.60), which is statistically distinguishable from zero (paired *t* = 6.33, $p=8.7\times 10^{-9}$; Wilcoxon signed-rank $p=2.7\times 10^{-7}$), with DeepSeek-R1-70B higher on 61 datasets, DeepSeek-R1-671B higher on 29, and the two tied on 3.

The gap is, however, strongly dataset-dependent and should not be read as a uniform advantage. Averaged within examination category, DeepSeek-R1-70B leads by 13.25 points on the 32 Chief Physician datasets—where it is higher on every one of the 32—but the ordering *reverses* on the licensing examinations (−3.69) and on the health-claims classification task (−3.50), and narrows to 1.78 on the English panel. The 671B variant also shows markedly greater dispersion across datasets (SD 10.63 vs. 5.48), so its lower macro-average partly reflects instability across specialties rather than a consistent deficit. We therefore state the finding as an ordering of macro-averaged accuracy that is statistically supported overall but concentrated in advanced specialty examinations, and we do not claim that the 70B architecture is uniformly superior. Not all adjacent ranks in Table 2 are distinguishable either: the confidence intervals for DeepSeek-R1-14B (74.6–77.6) and DeepSeek-R1-671B (73.8–78.2) overlap, so ranks 4 and 5 should be treated as statistically indistinguishable. This non-monotonic scaling behavior is discussed in the Discussion section.

### 4.2 Dataset-specific performance

#### 4.2.1 USMLE.

On the USMLE dataset [34], models achieve remarkably high accuracies far exceeding the human passing threshold (approximately 60%). GPT-OSS-120B leads with 97.5% accuracy, followed by DeepSeek-R1-671B [29] (97.0%) and DeepSeek-R1-70B (95.5%). These results substantially exceed the 60% passing threshold and surpass Med-PaLM 2’s 86.5% [1], demonstrating that state-of-the-art open-source models can match or exceed proprietary alternatives on standardized medical examinations, a finding consistent with recent evaluations in Nature Medicine [48].

#### 4.2.2 AJKD.

The nephrology-focused AJKD dataset presents greater challenges, consistent with prior nephrology benchmarking studies [5]. DeepSeek-R1-70B and Phi4-Reasoning-14B [26] both achieve the highest accuracy at 82.3%, followed by DeepSeek-R1-671B (77.2%) and Magistral-24B [32] (75.9%). Performance on AJKD is lower than on USMLE across all evaluated models; top models nevertheless substantially outperform the 30.6% achieved by the best open-source models in earlier nephrology evaluations [5].

#### 4.2.3 Chinese medical examinations.

On Chinese Medical Licensing Examinations [35,36], reasoning-enhanced models achieve strong performance. DeepSeek-R1-70B leads with 81.5% average accuracy, followed by QWQ-32B [30] (78.1%), DeepSeek-R1-671B [29] (75.5%), and Phi4-Reasoning-14B (72.5%). These results substantially exceed the 61.6% GPT-4 accuracy reported in the original CMExam benchmark [35]. Performance increases with professional level difficulty, with top models achieving higher accuracy on Chief Physician (CP) examinations than entry-level Medical Licensing Examinations (MLE), potentially reflecting the longer reasoning chains in advanced examinations that favor reasoning-specialized architectures [50].

#### 4.2.4 Cross-lingual performance comparison.

Table 4 compares top model performance on English (USMLE) versus Chinese medical examinations. DeepSeek-R1 variants demonstrate the strongest bilingual capability, with DeepSeek-R1-70B achieving 95.5% on USMLE and 81.5% on Chinese examinations—a gap of only 14 percentage points. In contrast, English-primary models such as Llama-3.1-405B show dramatic cross-lingual degradation (95.0% USMLE vs. 52.0% Chinese), indicating that bilingual (Chinese-English) medical reasoning remains a significant challenge even for state-of-the-art models.

**Table 4:** Cross-lingual performance comparison of top models on English (USMLE) and Chinese medical examinations.

Model	USMLE (%)	Chinese (%)
DeepSeek-R1-671B	97.0	75.5
DeepSeek-R1-70B	95.5	81.5
Llama-3.1-405B	95.0	52.0
Phi4-Reasoning-14B	95.5	72.5
Qwen3-235B	88.5	55.1
QWQ-32B	82.1	78.1

Figure 2 shows the accuracy distribution of the top 20 models across all 93 datasets, illustrating the consistency of reasoning-enhanced models relative to general-purpose alternatives.

**Fig 2:**
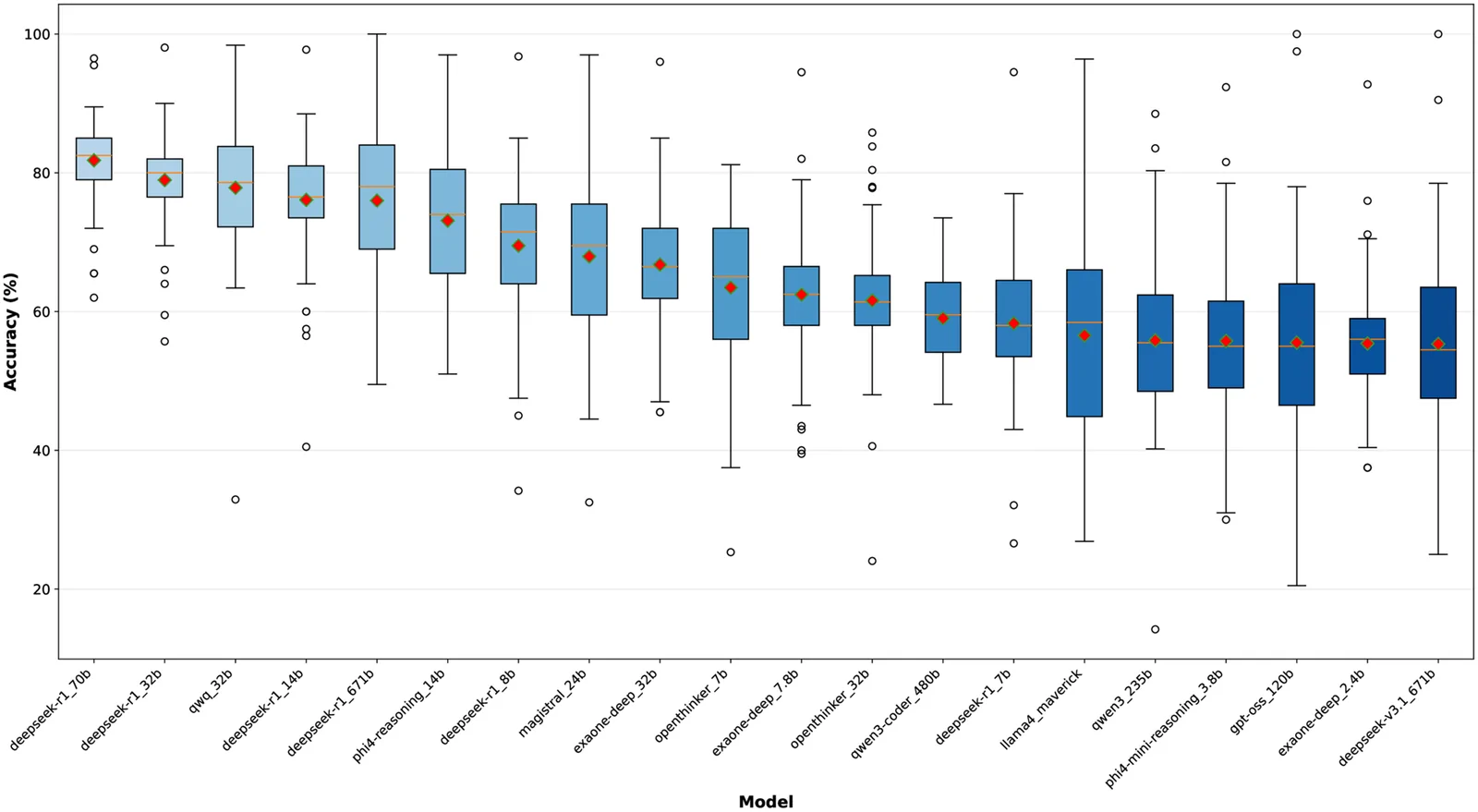
Box plot visualization of accuracy distributions for the top 20 performing models across all 93 medical benchmark datasets, with boxes representing the interquartile range (25th to 75th percentile), whiskers extending to 1.5× IQR, and red diamond markers indicating mean accuracy values. DeepSeek-R1-70B exhibits both the highest mean accuracy (81.8%) and the smallest interquartile range among top performers, indicating remarkably consistent performance across diverse medical specialties and difficulty levels. The tight distributions observed for reasoning-enhanced models (DeepSeek-R1, QWQ, Phi4-Reasoning) contrast with the wider spreads typical of general-purpose models, suggesting that explicit reasoning training produces more robust and transferable medical knowledge representations.

### 4.3 Model family analysis

Figure 3 compares performance across major model families. The DeepSeek-R1 family achieves the highest average accuracy of 68.9% across all variants, significantly outperforming other families. This advantage persists across all model sizes, from the 7B variant (58.3%) to the 70B variant (81.8%).

**Fig 3:**
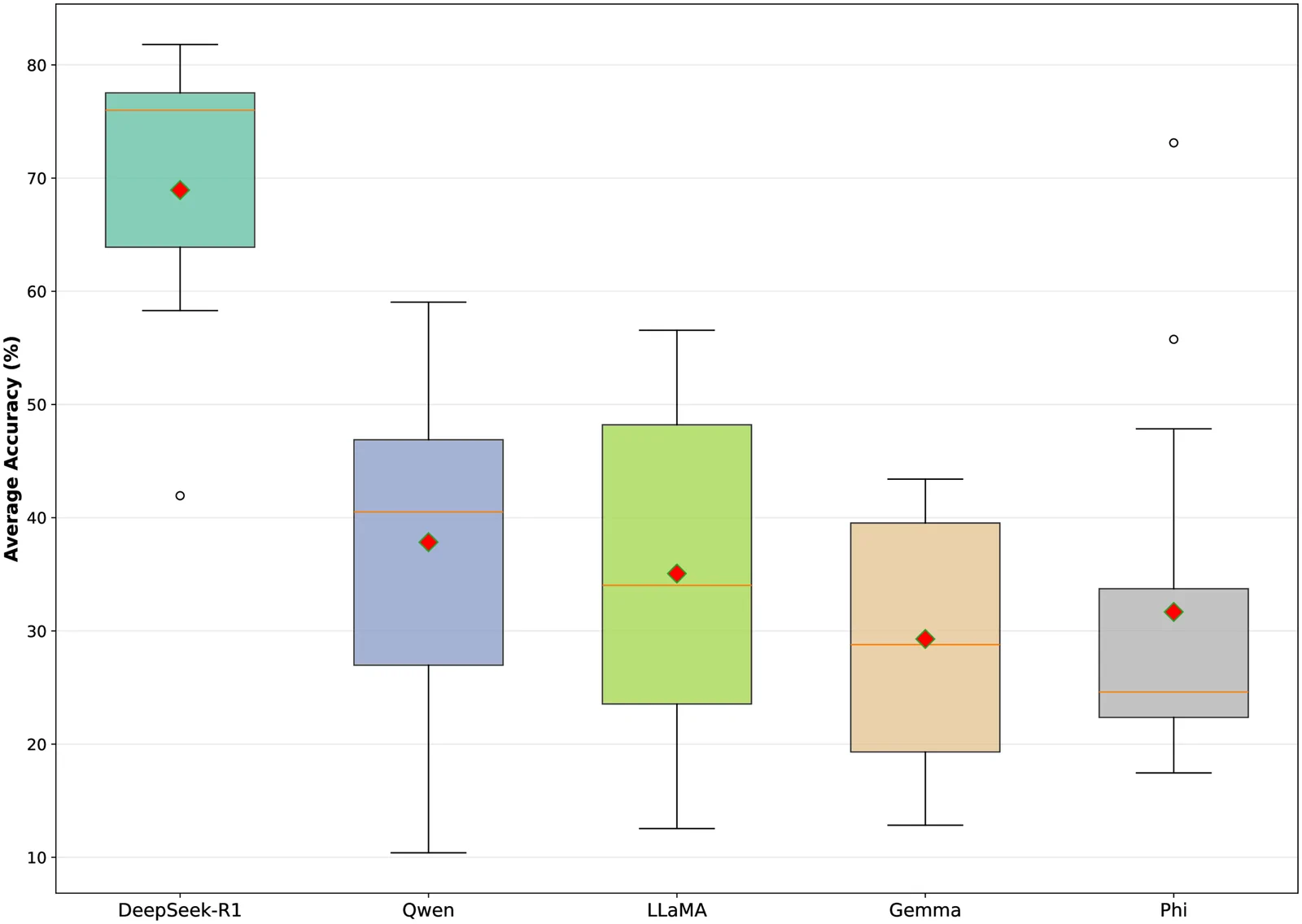
Comparative box plot analysis of accuracy distributions across major model families, aggregating all variants within each family to reveal systematic architectural advantages. The DeepSeek-R1 family demonstrates clear superiority with a median accuracy approximately 25 to 30 percentage points above general-purpose families including Qwen3, Qwen2.5, and Llama. This substantial performance gap persists even when comparing the smallest DeepSeek-R1 variant (7B, 58.3%) against the largest general-purpose models (Llama-3.1-405B), providing strong evidence that reasoning-specialized training methodologies, rather than raw parameter count, constitute the primary determinant of medical question-answering capability.

General-purpose model families show substantially lower performance: Qwen3 averages 41.0%, Qwen2.5 averages 42.1%, and Llama averages 35.1%. The 27–34 percentage point gap between DeepSeek-R1 and general-purpose families underscores the importance of reasoning-specific training for medical applications.

### 4.4 Scaling behavior

Figure 4 shows the relationship between model parameters and accuracy for different model families. Reasoning-enhanced models (DeepSeek-R1, Phi4-Reasoning) show steeper scaling curves compared to general-purpose models, achieving higher accuracy at equivalent parameter counts.

**Fig 4:**
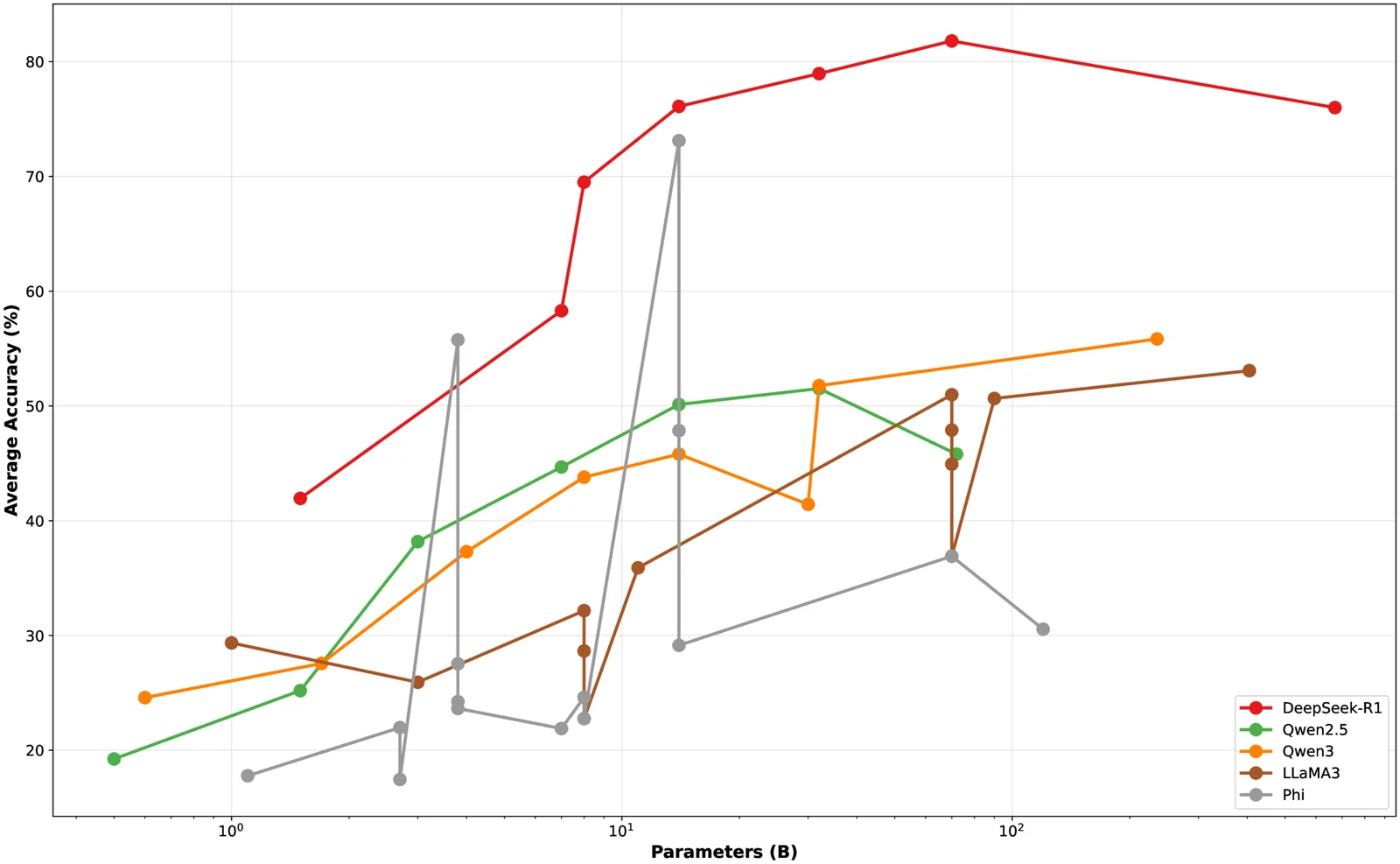
Scaling behavior analysis showing the relationship between model parameters (x-axis, logarithmic scale) and average accuracy (y-axis) across major model families, with each point representing a specific model variant and lines connecting variants within the same family. Reasoning-enhanced architectures (DeepSeek-R1, Phi4-Reasoning) exhibit substantially steeper scaling curves compared to general-purpose families, achieving equivalent accuracy at 5–10× fewer parameters. The non-monotonic behavior within the DeepSeek-R1 family is particularly notable, with the 70B variant outperforming the 671B variant, suggesting that current reasoning training methodologies may have an optimal scale beyond which additional parameters provide diminishing or negative returns for medical reasoning tasks.

Notably, the scaling relationship is not monotonic within the DeepSeek-R1 family: the 70B variant outperforms both smaller (32B, 14B) and larger (671B) variants. This suggests an optimal scale for the current training methodology, beyond which additional parameters may not improve medical reasoning performance.

### 4.5 Instruction following and error analysis

Beyond accuracy, we evaluate models’ instruction-following capabilities through our structured output agent. Models are required to respond with only the option letter, testing their ability to adhere to clinical output format requirements. Table 5 summarizes instruction-following compliance rates across model categories. Reasoning-enhanced models demonstrate superior instruction following: DeepSeek-R1 variants achieve 99.2% format compliance, compared to 94.7% for general-purpose Llama models and 87.3% for smaller models under 3B parameters. This finding has direct implications for clinical deployment, where strict output formatting is essential for integration with electronic health record systems.

**Table 5:** Instruction-following compliance rates across model categories, measuring the percentage of responses that correctly output only the selected option letter (A–E) as required by our evaluation agent.

Model Category	Compliance (%)	Params Range
DeepSeek-R1 Family	99.2	1.5B–671B
QWQ / Phi4-Reasoning	98.7	14B–32B
ExaOne-Deep / Magistral	97.4	2.4B–32B
Qwen3 / Qwen2.5	96.1	0.5B–235B
Llama 3 / 3.1	94.7	8B–405B
Mistral / Mixtral	93.2	7B–123B
Gemma / Gemma2	91.8	2B–27B
Small Models (<3B)	87.3	0.5B–3B

Our LLM-as-judge analysis of incorrect responses reveals distinct error patterns across model architectures. Three independent frontier judges (Qwen3-235B [22], GPT-OSS-120B, and a third comparable model) each classified every incorrect response, and a category was recorded only where at least two judges agreed; in a random sample of 100 error cases independently reviewed by two co-authors with clinical backgrounds, the automated classifications agreed with the human reviewers in 87% of cases. On that basis we categorize errors into four types as shown in Table 6: *knowledge gaps* (38.2% of errors), where models lack required medical facts; *reasoning failures* (29.4%), where correct knowledge is incorrectly applied; *hallucinations* (21.7%), where models generate plausible but incorrect claims; and *instruction errors* (10.7%), where models misinterpret question requirements. These proportions are an *exploratory* characterization of failure modes rather than expert-adjudicated estimates: the 13% residual disagreement with human reviewers, together with the judge-bias concern set out in Section 5.8, means the absolute percentages should be read as indicative. We therefore rely on the taxonomy only for the between-family contrast, which is the comparison a uniform judge bias would least distort. Within that limited reading, reasoning-enhanced models show substantially lower hallucination rates (14.3%) compared to general-purpose models (28.9%), suggesting that explicit reasoning training improves factual grounding.

**Table 6:** Error type distribution from the LLM-as-judge analysis, in which three independent frontier judges classified each incorrect response and a category was recorded only on agreement of at least two. In a random sample of 100 cases reviewed by two clinically trained co-authors, automated and human classifications agreed in 87% of cases. These proportions are exploratory rather than definitive; see Section 5.8.

Error Type	Reasoning (%)	General (%)
Knowledge Gaps	42.1	34.8
Reasoning Failures	31.2	27.9
Hallucinations	14.3	28.9
Instruction Errors	12.4	8.4

### 4.6 Inference speed analysis

For practical deployment planning, we analyze the trade-off between accuracy and inference speed. Figure 5 presents relative inference speeds across all evaluated models, measured against DeepSeek-R1-70B as baseline (1.0×). As defined in the Performance Metrics subsection, relative speed is the ratio of aggregate end-to-end benchmark completion times on one fixed 8× H100 configuration; it is a relative serving-throughput indicator and not a measured clinical response time, and it does not by itself establish deployment feasibility on other hardware.

**Fig 5:**
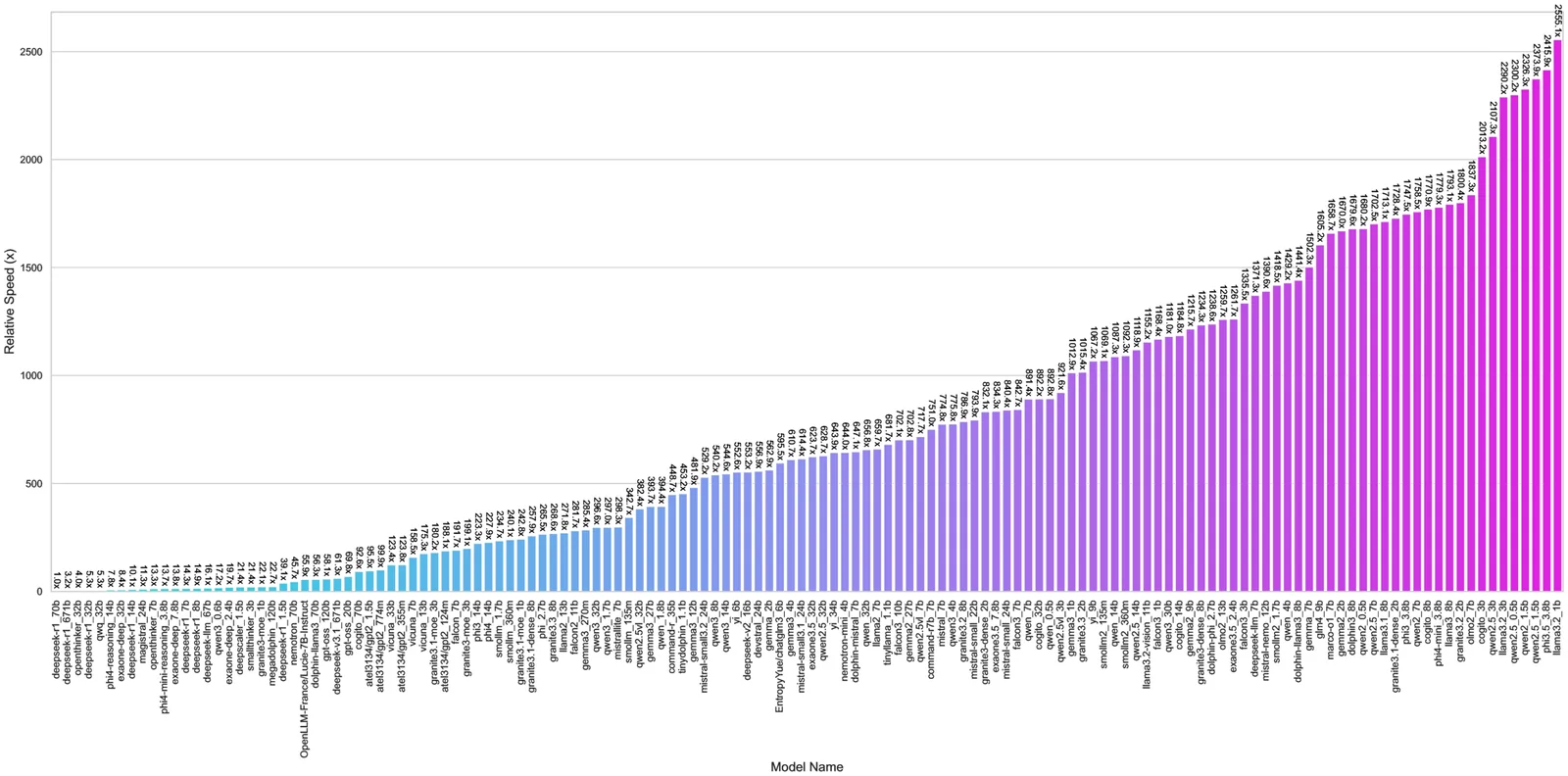
Comprehensive inference speed comparison across all 172 evaluated models was conducted on a standardized 8× NVIDIA H100 80GB hardware configuration using vLLM [37], with performance expressed relative to the DeepSeek-R1-70B baseline (1.0×) on a logarithmic scale spanning four orders of magnitude. The visualization reveals a continuous spectrum of deployment options. Ultra-compact models (1B to 3B parameters) achieve speedups exceeding 2000×, making them suitable for real-time clinical triage on consumer hardware. Mid-range models (7B to 14B) provide a balanced trade-off, with 1000 to 1800× speedup while maintaining 40 to 75% accuracy. Large reasoning models (32B to 70B) deliver state-of-the-art accuracy at 4 to 5× baseline speed and remain practical for complex diagnostic support scenarios where accuracy requirements outweigh latency constraints.

Within that reading, the suite spans a wide range of candidate operating points. Small models (1B–3B parameters) achieve 2000×+ speedup with moderate accuracy (20–40%), which makes them candidates for high-volume, low-acuity uses such as real-time triage or initial screening. Mid-size models (7B–14B) offer balanced profiles with 1000–1800× speedup and 40–75% accuracy. Large reasoning models (32B+) provide the highest accuracy (77–82%) at 4–5× baseline speed, a profile consistent with complex diagnostic support where accuracy outweighs latency. We present these as potential application areas that follow from the measured accuracy–throughput trade-off, not as demonstrated clinical readiness: none of these models has undergone prospective clinical validation, and any such use would require the safety assessment and regulatory approval discussed in Section 5.8.

Figure 6 visualizes the parameter-accuracy relationship across all 172 models. The plot reveals diminishing returns at larger scales for general-purpose models, while reasoning-enhanced models (labeled) achieve substantially higher accuracy at equivalent parameter counts.

**Fig 6:**
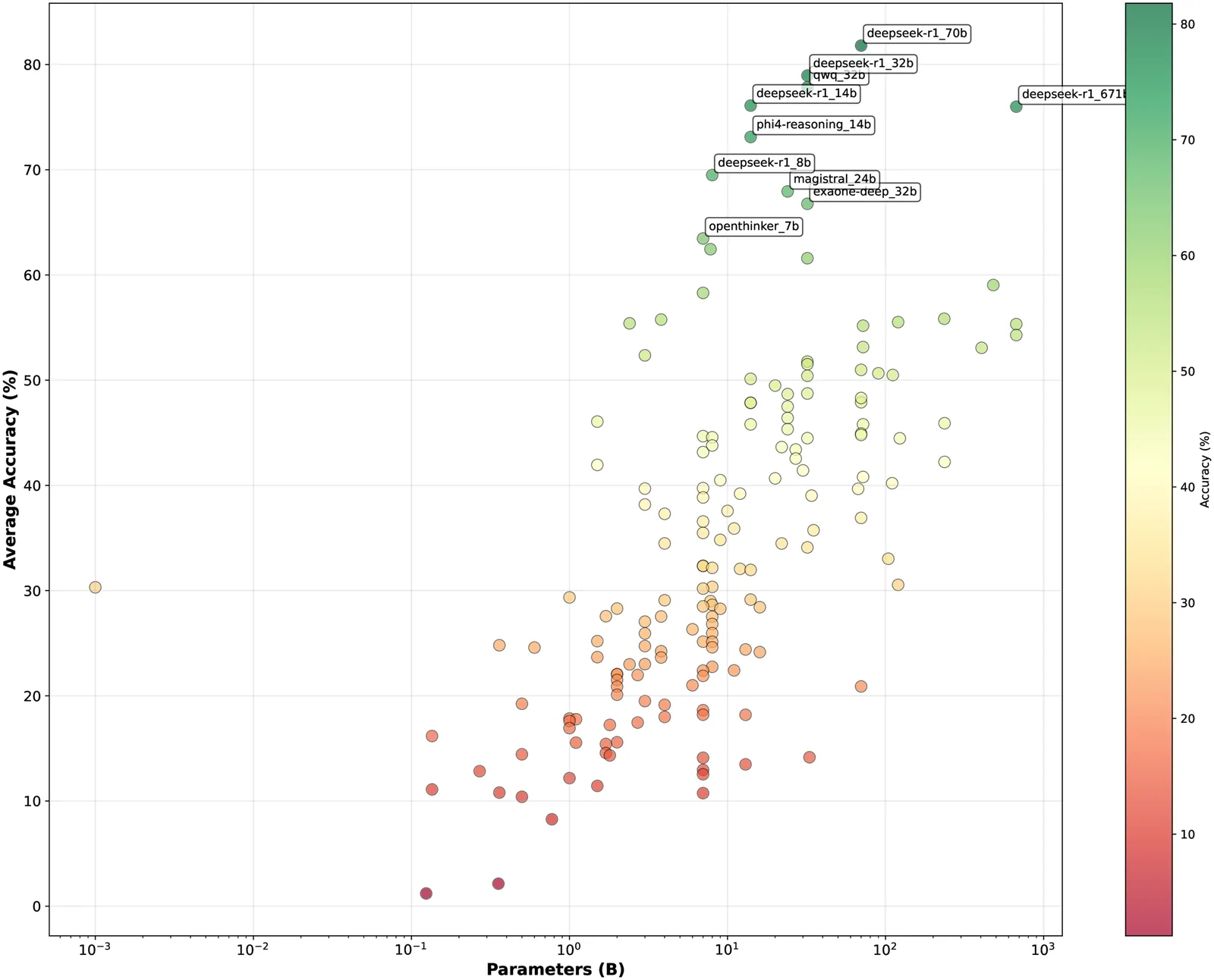
Scatter plot visualization of all 172 evaluated models positioned by parameter count (x-axis, logarithmic scale from 0.5B to 671B) versus average accuracy across all 93 datasets (y-axis), with the top 10 performing models individually labeled. The plot reveals two distinct performance populations: general-purpose models (lower cluster) exhibiting logarithmic scaling with substantial diminishing returns above 70B parameters, and reasoning-enhanced models (upper cluster) achieving 20–40 percentage points higher accuracy at equivalent parameter counts. This bifurcation provides visual evidence that architectural innovations in reasoning training can substitute for order-of-magnitude increases in model scale, with profound implications for practical healthcare AI deployment where computational resources are constrained.

Figure 7 summarizes benchmark composition across the five examination categories in terms of dataset count, total sample volume, and average question length.

**Fig 7:**
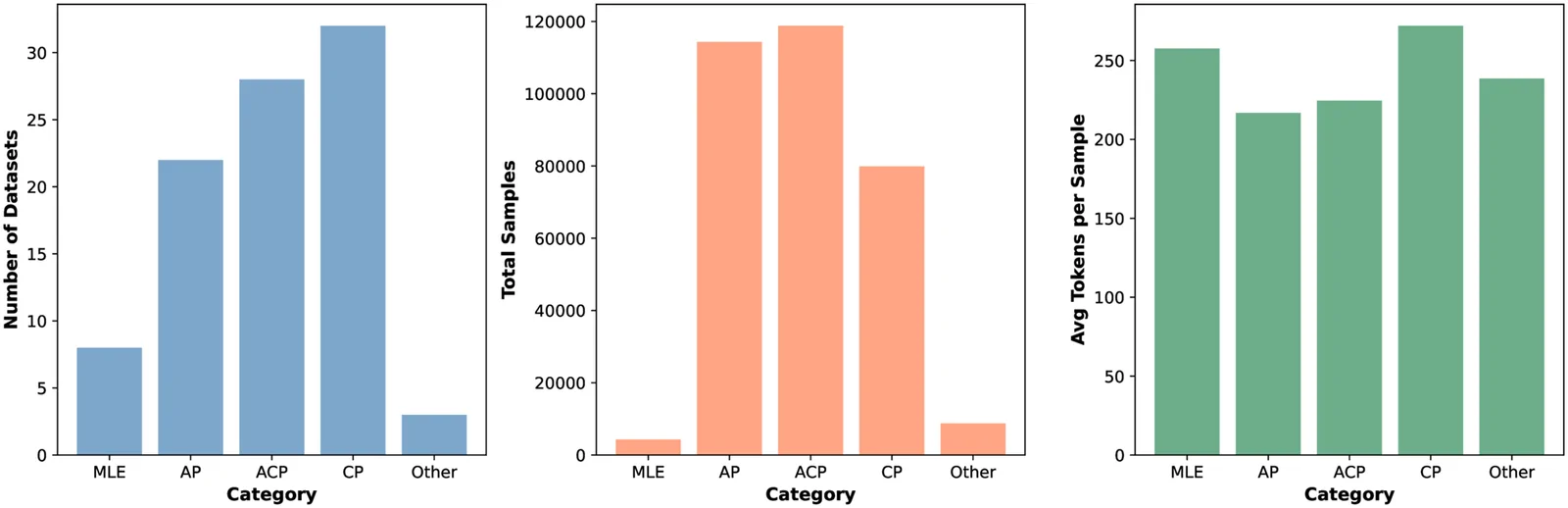
Tripartite summary statistics characterize the composition of the OpenMedEval benchmark across five examination categories: Medical Licensing Examination (MLE), Attending Physician (AP), Associate Chief Physician (ACP), Chief Physician (CP), and International examinations. Panel (a) displays the number of distinct datasets within each category, ranging from 3 international datasets to 32 Chief Physician specialty examinations. Panel (b) shows total sample counts per category, indicating that Associate Chief Physician and Attending Physician examinations contribute the largest sample volumes, with 118,798 and 114,327 questions, respectively. Panel (c) presents average token counts per sample, with higher professional levels (CP and ACP) featuring longer clinical vignettes averaging 200 to 300 tokens, compared to 150 to 200 tokens for entry-level examinations. This pattern reflects the increased complexity and contextual richness of advanced medical scenarios.

## 5 Discussion

### 5.1 The dominance of reasoning-enhanced architectures

Our results demonstrate a striking performance gap between reasoning-enhanced and general-purpose architectures. DeepSeek-R1 [29], QWQ [30], and Phi4-Reasoning [26] models consistently outperform general-purpose alternatives by 20–40 percentage points, regardless of parameter count. This finding has profound implications for medical AI development and aligns with recent research demonstrating that explicit reasoning capabilities are essential for clinical diagnostic tasks [13,50].

The superiority of reasoning-enhanced models suggests that medical question answering fundamentally requires multi-step logical inference rather than simple pattern matching. Medical diagnosis involves integrating symptoms, patient history, laboratory findings, and clinical knowledge through complex reasoning chains—precisely the capability that reasoning-enhanced training methodologies target. This observation is consistent with research showing that chain-of-thought prompting enables LLMs to mimic clinical reasoning processes without sacrificing diagnostic accuracy [14].

Notably, Phi4-Reasoning-14B (73.1% average accuracy) outperforms Llama-3.1-405B [19], demonstrating that architectural innovations can compensate for a 30-fold difference in parameter count. Similarly, ExaOne-Deep-7.8B [31] achieves competitive performance against much larger general-purpose models. This finding challenges the scaling hypothesis that larger models invariably perform better, at least for tasks requiring explicit reasoning, and has been corroborated by recent evaluations showing that reasoning-enhanced architectures achieve equivalent performance at 5% the parameter count of general-purpose alternatives [48].

A particularly noteworthy case is the non-monotonic scaling behavior within the DeepSeek-R1 family, where the 70B variant (81.8%) outperforms the 671B variant (76.0%). The difference is statistically supported when the two models are compared pairwise across the 93 datasets they both attempted (mean difference 5.81 points, 95% CI 4.01–7.60; paired *t* = 6.33, $p=8.7\times 10^{-9}$; Wilcoxon $p=2.7\times 10^{-7}$), so it is not a sampling artefact of the macro-average. It is, however, far from uniform, and we report it with that qualification. The 70B variant is higher on 61 of the 93 datasets and the 671B variant higher on 29, with 3 ties; averaged within category the 70B advantage reaches 13.25 points on the 32 Chief Physician datasets—where it is higher on all 32—but the ordering *reverses* on the annual licensing examinations (−3.69) and on the health-claims classification task (−3.50). The 671B variant is also substantially more dispersed across datasets (SD 10.63 vs. 5.48), so part of its lower macro-average reflects instability across specialties rather than a uniform deficit. The finding should therefore be read as a claim about macro-averaged performance across this particular benchmark mixture, driven mainly by advanced specialty examinations, and not as evidence that the 70B architecture is superior across medical tasks generally.

Several factors may contribute to this pattern: (1) the 671B model employs a Mixture-of-Experts (MoE) architecture with only 37B active parameters per token, which may limit per-token reasoning depth relative to the fully-dense 70B model; (2) the two variants may have been optimized for different task distributions during training, with the 70B variant potentially receiving more focused medical-domain optimization; (3) the extended reasoning chains characteristic of larger models may occasionally introduce compounding errors across multi-step inferences. These remain candidate explanations rather than demonstrated mechanisms, and we cannot adjudicate between them with the data reported here. This pattern, also observed in non-medical reasoning benchmarks, is consistent with reasoning quality—rather than total parameter count—being an important determinant of medical question-answering performance. Future work should investigate optimal training configurations for large MoE models on clinical reasoning tasks, and should report per-category results so that effects concentrated in a subset of dataset types are not averaged into a headline figure.

### 5.2 Parameter efficiency and potential deployment scenarios

The practical deployment of medical AI systems requires balancing accuracy against computational constraints [52]. Our speed analysis, conducted using vLLM [37] on a single fixed 8× NVIDIA H100 configuration, reveals a 2000× variation in aggregate serving throughput across evaluated models. Because this is a relative throughput measure on one hardware configuration rather than a measured clinical response time, the scenarios below are candidate application areas suggested by the accuracy–throughput trade-off, not evidence of clinical readiness; each would require prospective validation, safety assessment, and regulatory approval before use with patients.

For high-volume, low-acuity uses such as real-time triage and initial screening, smaller models (1B–7B parameters) such as SmolLM2 [53] and TinyLlama [54] achieve 2000×+ speedup and may offer an acceptable accuracy–latency compromise. For complex diagnostic support where accuracy is paramount, larger reasoning models (32B–70B) provide superior performance at 4–5× baseline speed, a profile consistent with non-urgent consultation workflows.

The ExaOne-Deep family [31,49] demonstrates particularly favorable efficiency, with the 7.8B variant achieving 62.4% accuracy—competitive with much larger general-purpose models while remaining within reach of modest on-premises hardware. Such models are attractive candidates for resource-constrained healthcare settings where on-premises deployment is mandated by privacy regulations [6], though we did not measure their memory footprint or utilisation and therefore do not characterize their exact hardware requirements here.

### 5.3 Bilingual (Chinese-English) medical reasoning

Our evaluation reveals important insights about bilingual (Chinese-English) medical AI capabilities that address a critical gap in the literature [9,10]. DeepSeek-R1-70B demonstrates the strongest bilingual capability, achieving 81.5% on Chinese medical licensing examinations [35] while maintaining 95.5% on USMLE [34]. This 14 percentage point gap is notably smaller than the 43 percentage point gap observed for Llama-3.1-405B (95.0% USMLE vs. 52.0% Chinese), highlighting significant variation in cross-lingual medical reasoning capability among state-of-the-art models.

We qualify these cross-lingual readings by the size of the English panel. The term *bilingual* describes the languages covered by the benchmark, not a balance between them: 325,647 of the 326,100 questions (99.9%) are Chinese and 453 (0.1%) are English, spread over only two English datasets (USMLE and AJKD). A two-dataset panel supports a descriptive comparison of the kind above, but it cannot support a stable 172-model English ranking, and the English panel indeed does not reproduce the overall ordering (Spearman ρ=0.857). We therefore report the English results as a separate reference panel in Table 3 rather than folding them into an overall score, and we state plainly that the overall leaderboard is Chinese-dominant: it agrees almost perfectly with a ranking computed on the 90 Chinese examination datasets alone (Spearman ρ=0.9998, with an identical top ten in identical order). Readers interested in English-language medical performance specifically should use the USMLE and AJKD columns directly, and cross-lingual conclusions in this paper should be read as indicative rather than as precisely estimated quantities.

This finding has significant implications for global healthcare AI deployment. Research has documented substantial performance disparities when LLMs process healthcare queries in non-English languages [10], limiting access to AI-assisted healthcare for billions of non-English speakers. Models trained on bilingual corpora can serve diverse patient populations without requiring separate language-specific systems, potentially reducing development costs and ensuring more equitable access to medical AI assistance.

### 5.4 Specialty-specific performance variation

Model performance varies substantially across medical specialties, with some models showing particular strength in specific domains. This variation suggests opportunities for specialty-specific model selection or ensemble approaches that leverage complementary strengths. Recent research has demonstrated that multi-agent frameworks, where multiple LLMs collaborate on diagnostic tasks, can outperform individual models by combining diverse perspectives [15–17].

The heat map analysis (Figure 8) reveals that reasoning-enhanced models maintain more consistent performance across specialties compared to general-purpose alternatives, which show greater variance. This consistency may reflect the generalizable nature of reasoning capabilities versus domain-specific knowledge patterns. Specialty-level variation may also reflect intrinsic differences in clinical complexity: some disciplines require integration of more heterogeneous information, broader differential diagnoses, longitudinal disease trajectories, imaging or laboratory interpretation, and risk-benefit trade-offs across multiple management options. Such specialties impose higher demands on contextual understanding and multi-step reasoning than domains dominated by more standardized factual recall or protocolized decision rules. The MIT Co-LLM algorithm [55] demonstrates how general-purpose and specialist models can be effectively paired, and our comprehensive evaluation provides the foundational data for designing optimal model combinations for specific clinical domains.

**Fig 8:**
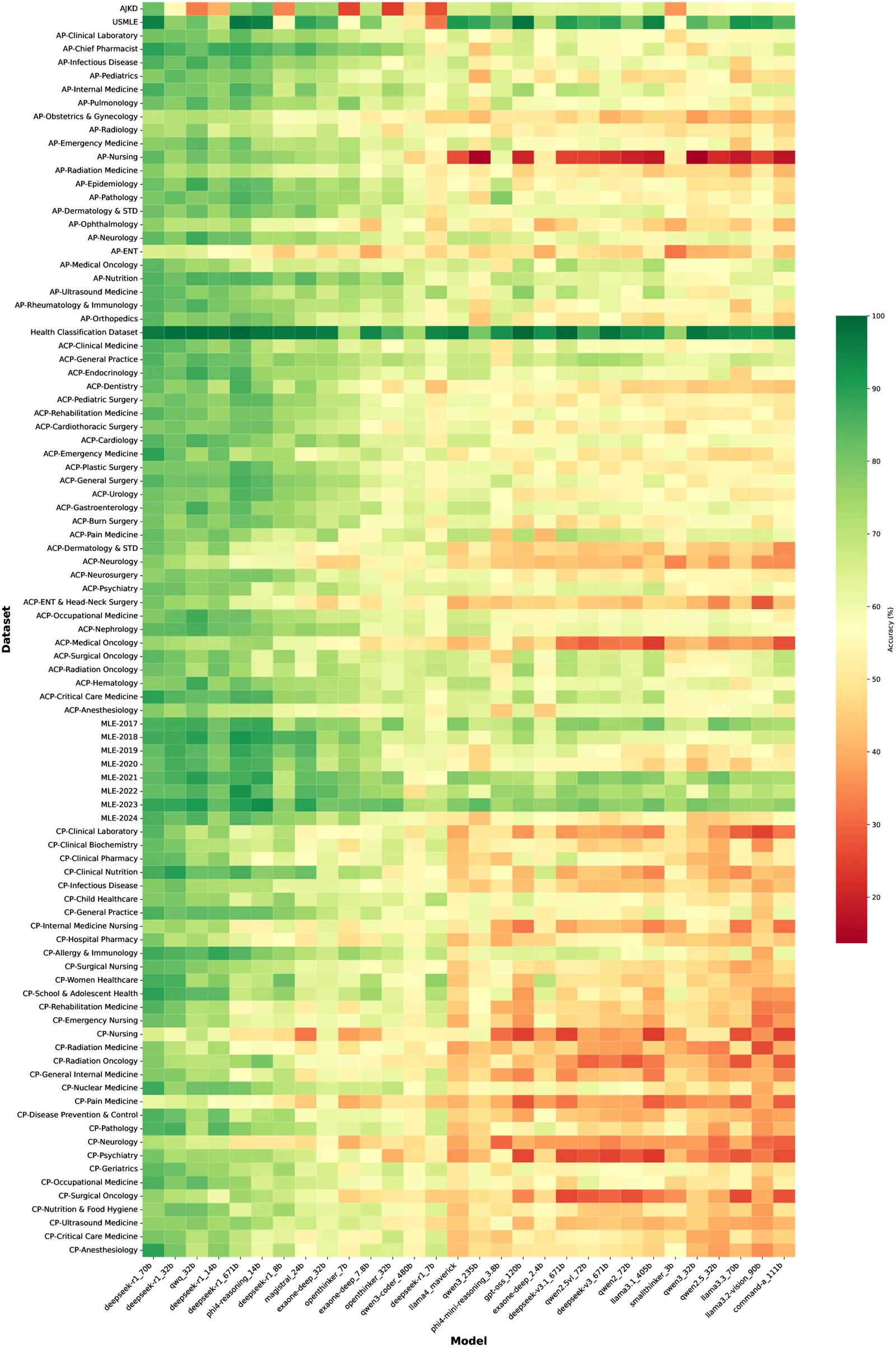
Comprehensive performance heatmap visualizing accuracy scores of the top 30 models (rows) across all 93 medical benchmark datasets (columns), with color intensity representing accuracy levels from red (low performance, below 40%) through yellow (moderate, 40–60%) to green (high performance, above 70%). The heatmap reveals several key patterns: DeepSeek-R1 variants demonstrate consistently high performance (dark green) across virtually all datasets regardless of medical specialty or difficulty level; reasoning-enhanced models maintain more uniform color patterns indicating lower variance across domains compared to general-purpose models which show pronounced specialty-dependent variation; and certain datasets (visible as vertical stripes) present universal challenges across all model architectures, suggesting intrinsically difficult clinical reasoning requirements.

### 5.5 Privacy and deployment considerations

Our exclusive focus on open-source models reflects fundamental healthcare AI deployment requirements. Medical data privacy regulations (HIPAA, GDPR) impose strict constraints on data processing that favor local deployment [6]. Open-source models enable on-premises inference where patient data never leaves institutional firewalls, fine-tuning on sensitive clinical datasets without third-party exposure, and regulatory compliance through existing institutional infrastructure. These capabilities—unavailable with proprietary cloud-based services—make open-source models the only viable option for many healthcare institutions. Our benchmark provides the foundation for selecting models that meet accuracy requirements while preserving complete data sovereignty.

### 5.6 Implications for medical examination integrity

The remarkable performance of LLMs on medical licensing examinations, with top models exceeding 97% on USMLE and 89% on Chinese national examinations, raises profound questions about examination security and the future of professional credentialing [11,12]. Our findings demonstrate that current examination formats, designed for human test-takers, are highly vulnerable to AI assistance.

Our comprehensive evaluation across examination difficulty levels provides essential data for medical education authorities. The observation that model performance often *increases* with examination difficulty level (with higher accuracy on Chief Physician examinations than entry-level licensing exams) suggests that complex reasoning questions, counterintuitively, may be more vulnerable to AI assistance than straightforward knowledge recall. These findings support recommendations that high-stakes medical assessments should be conducted exclusively under proctored conditions with appropriate monitoring for unauthorized device use.

### 5.7 Addressing potential concerns

Several aspects of our methodology and findings merit explicit discussion to address potential concerns.

**Why focus on open-source models?** While proprietary models such as GPT-4 and Claude demonstrate strong medical performance [1,5], healthcare institutions face fundamental barriers to their deployment including data privacy regulations, lack of transparency, and vendor dependency. Open-source models enable on-premises deployment where patient data never leaves institutional servers—a critical requirement for HIPAA compliance [6]. Our exclusive focus on open-source alternatives provides actionable guidance for the many healthcare systems that cannot legally or operationally use cloud-based AI services.

**Why use official examination questions?** Unlike synthetically generated or crowd-sourced questions that may contain errors, ambiguities, or distribution mismatches, official examination questions have undergone rigorous validation by medical professionals and regulatory authorities. This ensures that our benchmark assesses clinically relevant knowledge rather than artifacts of question generation processes. The hierarchical professional levels (MLE through CP) provide graduated difficulty that distinguishes genuine reasoning capability from surface-level pattern matching [35].

**Generalizability to clinical practice.** We acknowledge that examination performance does not directly translate to clinical utility [4]. However, multiple-choice medical examinations are specifically designed to assess the knowledge and reasoning capabilities required for safe clinical practice. Top-performing models that substantially exceed both the human passing threshold (60%) and Med-PaLM 2’s 86.5% benchmark [1,34]—achieving up to 97% on USMLE—demonstrate knowledge foundations that, with appropriate safety validation, may support clinical decision-making. Our speed-accuracy trade-off analysis provides practical deployment guidance that complements accuracy metrics.

**Potential training data contamination.** A common concern with LLM benchmarking is whether models have been trained on test data. We address this concern through four complementary lines of evidence.

*Temporal analysis*. Our benchmark spans examinations from 2017–2024, a range that extends beyond the training data cutoffs of most evaluated models. The temporal analysis uses the eight annual Chinese licensing examinations, comprising 4,296 questions (Table 1), split into an early period (2017–2020, four examinations) and a late period (2021–2024, four examinations). For each of the 168 models with complete data across both periods, we computed average accuracy separately on the two periods (Figure 9). The Pearson correlation between these two accuracy estimates is *r* = 0.973 ($p=1.9\times 10^{-107}$), and the Spearman rank correlation is ρ=0.980, indicating near-identical model rankings regardless of examination year. Rank agreement is high but not exact: the mean absolute rank shift between periods is 7.5 positions (median 6.0, maximum 29) out of 168, so the ordering is stable at the top while individual mid-ranked models do move.

**Fig 9:**
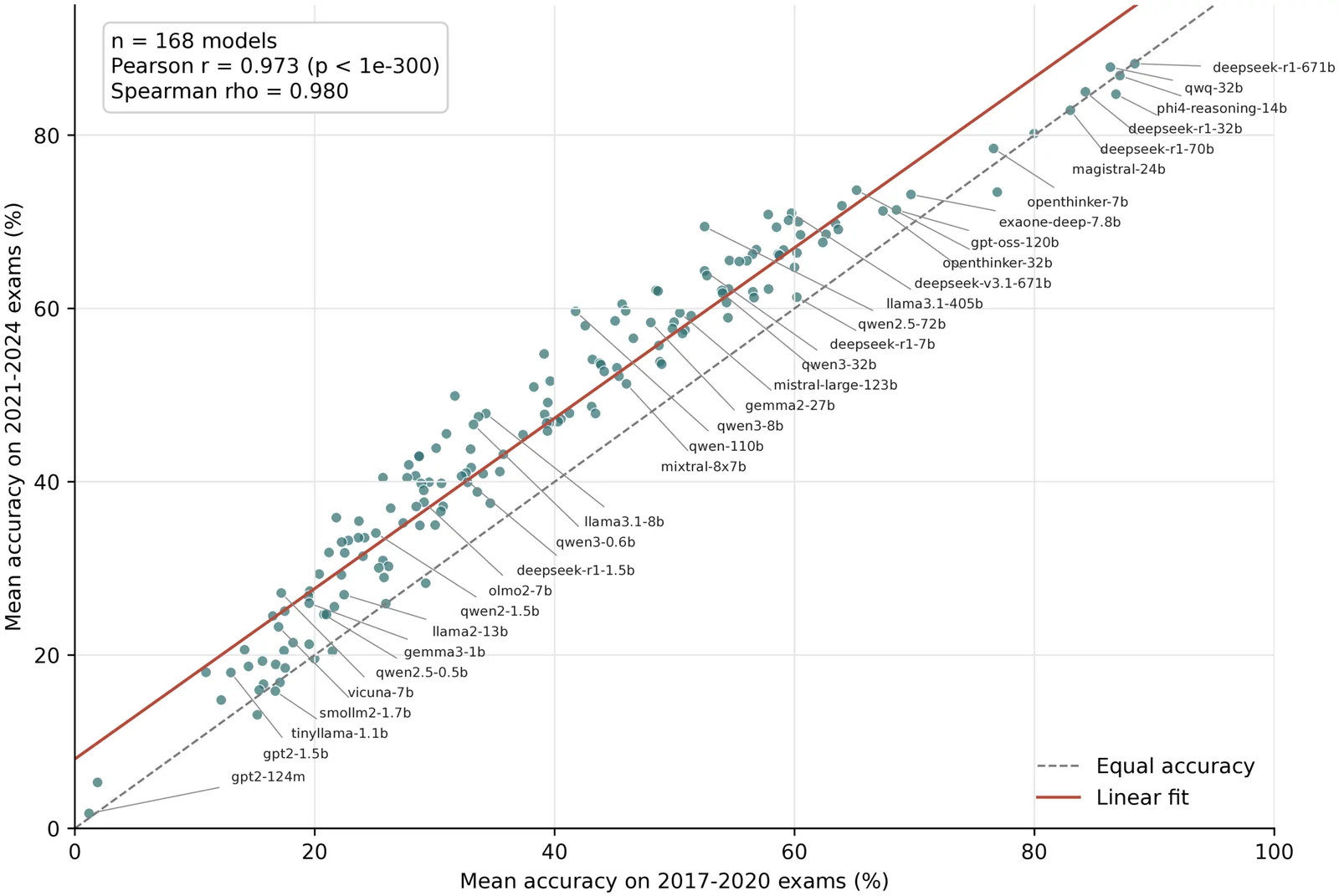
Year-split sensitivity analysis for potential training-data contamination. Each point represents one model with complete licensing-exam results. The x-axis shows mean accuracy on 2017–2020 examinations, and the y-axis shows mean accuracy on 2021–2024 examinations. The high Pearson and Spearman correlations indicate that model rankings remain highly stable across early and late examination periods, supporting the interpretation that performance differences primarily reflect general medical reasoning capability rather than memorization of older examination items.

The direction of the period effect is the more informative result. Averaged over the 168 models, accuracy is *lower* on the early period (39.57%) than on the late period (46.92%), and 157 of the 168 models score higher on late-period questions while only 11 score higher on early-period questions. If performance were driven chiefly by memorization of examination items that had entered pre-cutoff training data, the opposite asymmetry would be expected: models should do better on the older, more widely circulated items. The observed pattern is therefore inconsistent with memorization as the dominant driver, though it is also consistent with a genuine difference in difficulty or content emphasis between the two four-year blocks, which we cannot separate from a memorization effect using this design alone.

We state the scope of this evidence plainly. Consistency of model rankings across examination years shows that the *ordering* of models is not an artefact of which years were sampled; it does not establish that benchmark contamination has been eliminated, and we do not claim that it does. A model could have memorized items in both periods to a similar degree and still rank consistently, and rank stability is silent on that possibility. This analysis is therefore a consistency check on the ranking, not a contamination test, and the possibility of memorisation remains an explicit open limitation of this benchmark.

*Data accessibility*. Chinese medical licensing examinations are administered under controlled, proctored conditions and have not been systematically released online. Examination authorities publish only a small number of illustrative sample questions; the bulk of examination content used in this benchmark was obtained from official archives and has not been widely disseminated through channels typically indexed by web crawlers used to construct LLM training corpora.

*Architectural evidence*. If benchmark performance were driven *solely* by memorization of question-answer pairs, we would not expect the systematic performance advantage of reasoning-enhanced architectures (20–40 percentage points above general-purpose models of comparable or larger scale), since memorization of these items would be available to any model whose training corpus contained them, largely irrespective of training methodology. This argument is suggestive but not probative, and we do not treat it as ruling out contamination. It cannot exclude a mixed picture in which memorization raises all models’ scores by some common amount while architectural differences determine the ordering on top of it; under that picture the architectural gap would be genuine and contamination would still be present. Architectural differences alone therefore do not establish the absence of memorisation, and we do not claim that they do.

*Cross-year consistency pattern*. The observation that model performance on Chief Physician examinations (the most advanced tier) is often *higher* than on entry-level examinations is difficult to reconcile with memorization being the dominant mechanism, since advanced specialty examinations are less commonly cited and less likely to have been captured in training data. As with the architectural argument, this bears on the relative contribution of memorization rather than proving its absence.

Taken together, these lines of evidence are consistent with model performance reflecting reasoning capability to a substantial degree, and they argue against memorization being the *dominant* driver of the observed ranking. They do not establish that the benchmark is free of contamination, and no analysis available to us can. We therefore state our position explicitly: the possibility that examination questions, answer keys, or closely related versions appeared in the training data of one or more evaluated models cannot be excluded, because the training corpora of the 172 models are not publicly documented in sufficient detail to permit a direct overlap check, and we had no access to them. Definitive contamination detection remains an unsolved methodological challenge in LLM benchmarking. All performance figures in this paper should be read subject to that limitation, and we encourage future work to develop systematic decontamination protocols specific to medical examination benchmarks.

**Statistical reliability.** With 326,100 questions across 93 datasets, and every model answering every question, our evaluation provides substantially greater statistical power than prior medical LLM studies. Two distinct sources of uncertainty apply and we keep them separate. Question-level sampling error is negligible at this scale: the binomial standard error of a pooled accuracy over 326,100 questions is below 0.1 percentage point for every evaluated model (maximum 0.088 at *p* = 0.5). The uncertainty that actually governs model comparison is the dataset-level dispersion of the macro-average, since genuine differences in specialty difficulty and examination tier are part of what a ranking summarizes; across the 172 models this standard error has a median of 1.08 and a maximum of 1.52 percentage points. We report dataset-level SD, SE and 95% confidence intervals for the leading models in Table 2, and we compare models with paired-by-dataset tests rather than with question-level errors, because the paired design removes dataset difficulty as a source of noise. We also report performance across dataset categories and individual specialties, and we report where a headline difference is concentrated: the DeepSeek-R1-70B advantage over the 671B variant, for example, is statistically supported overall but arises mainly in advanced specialty examinations and reverses on the licensing examinations, so it should not be generalized across dataset types.

**Reproducibility.** All evaluations use deterministic inference (temperature = 0) with standardized prompting protocols. We provide complete model specifications, dataset descriptions, and evaluation code to enable independent verification of our results. The use of open-source models and open-source inference infrastructure (vLLM [37]) ensures that our methodology can be replicated without proprietary dependencies.

**Why not include proprietary models?** While GPT-4 and Claude comparisons would provide additional context, our exclusive focus on open-source models serves the practical needs of healthcare institutions. Organizations requiring AI-assisted clinical decision support cannot typically use cloud-based proprietary models due to privacy regulations [6]. Our benchmark answers the question most relevant to these institutions: which locally-deployable models best support medical applications?

**Multilingual extensibility.** Although the empirical language scope of this study is reported as Chinese-English bilingual, the OpenMedEval framework is language-extensible and the evaluated LLMs are generally multilingual-capable. We therefore distinguish between multilingual capability at the framework/model level and empirical language coverage at the dataset level. We avoid constructing additional language subsets by machine-translating the Chinese examination questions because such translated variants would largely preserve the same clinical logic, diagnostic pathway, and answer structure while changing only surface linguistic forms. For a reasoning-focused medical benchmark, this strategy may provide limited independent evidence while introducing translation artifacts, terminology shifts, and cultural-context noise. A more rigorous multilingual extension would require native, officially validated medical board examinations in additional languages; however, many such datasets are restricted by copyright and redistribution agreements. Given the scale of our 172-model evaluation, re-running the full model matrix on newly collected official datasets would also require substantial additional computation beyond the revision window. We therefore present the current study as a Chinese-English bilingual empirical evaluation while preserving the framework for future expansion to legally accessible native datasets in additional languages.

### 5.8 Limitations

Despite the strengths of our methodology, several limitations should be acknowledged.

*Examination format constraints*. Our evaluation focuses exclusively on multiple-choice questions, which may not fully capture the capabilities required for open-ended clinical reasoning, differential diagnosis generation, or nuanced patient communication that characterize real clinical practice. Models that excel at selecting among predefined options may not perform equivalently when required to generate structured clinical narratives or navigate ambiguous clinical scenarios.

*Temporal validity*. Medical knowledge evolves continuously, and models trained on older data may not reflect current clinical guidelines or emerging evidence; our benchmark captures performance at a single point in time. Regular re-evaluation will be necessary as both model capabilities and clinical standards advance.

*Gap between examination performance and clinical utility*. A critical limitation is that strong benchmark performance does not guarantee clinical utility [4]. Medical licensing examinations are designed to assess knowledge and reasoning in idealized, information-complete scenarios with a single correct answer. Real clinical practice involves incomplete information, diagnostic uncertainty, dynamic patient states, ethical trade-offs, and the need for effective communication with patients and colleagues—capabilities that multiple-choice benchmarks do not assess. Any deployment of these models in actual patient care settings requires prospective clinical validation, comprehensive safety assessments, systematic evaluation of demographic and diagnostic biases, and regulatory approval. The findings of this benchmark study should be interpreted as evidence of domain knowledge and structured reasoning capacity, not as readiness for clinical deployment.

*Hardware specificity and the scope of the speed metric*. All speed measurements were taken on NVIDIA H100 80GB devices in eight-device nodes and may not transfer to hardware commonly used in clinical settings, such as consumer GPU servers or CPU-only deployments. The metric itself is aggregate end-to-end throughput over an identical fixed workload, expressed relative to the slowest model; it is not a per-token generation rate, a prefill measure, or a single-request latency, and it therefore should not be read as a predicted clinical response time. Because reasoning-enhanced models emit longer outputs, completion time conflates output length with decoding speed.

Two further qualifications apply. First, serving topology was not identical across the suite: models whose weights exceed one node’s 640 GB capacity—the 400–480B class and the 671B class—were served across multiple nodes, and therefore incur inter-node communication overhead that single-node models do not. Their relative throughput figures are consequently not strictly topology-matched against smaller models, and the direction of that bias is to make the largest models look slower than they would on a single node. Second, the memory figures we report in the Methods are analytical weight-memory requirements derived from parameter count and released precision, not measured resident footprints; we did not measure KV-cache and activation memory per model, and we did not perform warm-up sweeps, repeated timing runs with reported dispersion, batch-size searches, or record per-model truncation incidence. These omissions bound how precisely a reader can translate our relative speeds into a concrete deployment plan, and we identify closing them as a priority for future reporting.

*Fixed generation cap*. The uniform 2,048-token generation cap is applied to every model, including reasoning-enhanced models that would naturally emit longer traces. This is a deliberate fairness choice—a per-model cap would make accuracies incomparable—but it is not neutral in effect: a model whose reasoning is truncated before it emits an answer is scored incorrect. Truncation therefore disadvantages long-reasoning models relative to what an uncapped protocol would yield, and because we did not record per-model truncation counts we cannot quantify the size of that disadvantage. The instruction-following compliance rates in Table 5 bound the overall rate at which responses left the directly scorable path, but they do not separate truncation from other causes of non-compliance.

*Language scope*. The present empirical evaluation is Chinese-English bilingual, with Chinese medical examinations as the primary setting and USMLE/AJKD as English reference benchmarks. Future work can extend this language-extensible framework to additional native medical examination systems where legally accessible, officially validated datasets are available.

*Limitations of LLM-as-Judge for error analysis*. Our qualitative error analysis relies on frontier models (Qwen3-235B, GPT-OSS-120B) as automated judges to categorize failure patterns at scale. While this approach enables analysis across thousands of incorrect responses, it introduces potential “judge bias.” A critical paradox arises from our own findings: since parameter count does not guarantee superior medical reasoning—as evidenced by the 70B model outperforming the 671B model—using a larger model as the definitive arbiter of smaller models’ errors is methodologically vulnerable. The judge model may penalize non-standard but clinically valid reasoning chains that diverge from its own patterns, or overlook error types that it also commits. To partially mitigate this concern, we employed three independent frontier models as judges and required agreement across at least two models for error categorization, reducing single-model bias. We further conducted manual validation on a random sample of 100 error cases reviewed by two co-authors with clinical backgrounds, finding that LLM judge classifications agreed with human reviewers in 87% of cases (procedure detailed in the LLM-as-Judge Error Analysis subsection of the Methods). An 87% agreement rate is a meaningful but partial validation: it was obtained on a single 100-case sample reviewed by two authors rather than by an independent expert panel with adjudicated disagreements, and it does not establish per-category reliability, since some error types will be classified more robustly than others. Accordingly, the error taxonomy in Table 6 is presented throughout this paper as *exploratory* rather than definitive, and we use it only to contrast architectural families rather than to estimate the absolute prevalence of any failure mode. Nevertheless, LLM-as-Judge cannot fully substitute for systematic expert review. Future work incorporating larger-scale, multi-reviewer human expert validation with reported inter-rater reliability would strengthen the reliability of error taxonomy findings.

### 5.9 Future directions

Our findings suggest several promising directions for future research. The demonstrated success of reasoning-enhanced architectures strongly motivates the development of medical-specific reasoning training methodologies [50], potentially leveraging clinical case conference discussions, published diagnostic reasoning traces, or expert-annotated decision pathways to create domain-specialized training data. The pronounced speed-accuracy trade-offs observed in our evaluation suggest substantial value in hybrid deployment architectures that intelligently route clinical queries to appropriately-sized models based on real-time complexity assessment [55], thereby optimizing both accuracy and computational efficiency. The multi-agent collaboration frameworks demonstrated in recent research [16,17] offer promising approaches for combining the complementary strengths of different models identified in our evaluation. Furthermore, regular longitudinal re-evaluation as both model capabilities and medical knowledge continue to evolve would provide valuable insights into the trajectory of medical AI development and help identify when model updates or replacements become necessary. Finally, the translation of benchmark performance to clinical utility represents perhaps the most critical future direction, requiring prospective studies that evaluate model deployment in real healthcare settings [4] with careful attention to clinician acceptance, workflow integration, patient outcomes, and unintended consequences.

## 6 Conclusion

We present OpenMedEval, the largest comprehensive benchmark study of open-source large language models for medical applications, evaluating 172 models on 326,100 questions across 93 datasets spanning diverse medical specialties and professional levels. Our benchmark encompasses established datasets including USMLE [34] and Chinese medical examinations [35,36], evaluated using efficient inference with vLLM [37] on standardized hardware.

Our key findings establish reasoning-enhanced architectures [26,29–32] as the state-of-the-art for medical question answering, with DeepSeek-R1-70B achieving 81.8% average accuracy and up to 97% on USMLE. The consistent 20–40 percentage point advantage of reasoning-enhanced models over general-purpose alternatives [19,22,23] demonstrates that explicit reasoning training is essential for medical AI applications, a finding with significant implications for clinical decision support [3,4] and multi-model collaboration frameworks [15,16].

Importantly, we show that parameter efficiency gains from reasoning training enable smaller models to match or exceed larger general-purpose alternatives. Phi4-Reasoning-14B [26] outperforms models 30× its size, while ExaOne-Deep-7.8B [31] provides competitive accuracy suitable for resource-constrained deployment where on-premises operation is mandated by privacy regulations [6].

Our speed analysis maps candidate operating points across the accuracy-efficiency spectrum, from real-time triage applications using small models (2000×+ speedup) to complex diagnostic support with large reasoning models (77–82% accuracy at 4–5× baseline speed). Because relative speed is an aggregate end-to-end throughput measure on one fixed 8× H100 configuration rather than a measured clinical response time, we present these as potential application areas implied by the accuracy–throughput trade-off, not as demonstrated deployment readiness; they provide comparative guidance for healthcare institutions shortlisting models for further evaluation [52].

We also state the boundaries of what this benchmark establishes. Strong performance on medical licensing examinations evidences domain knowledge and structured reasoning capacity under idealized, information-complete conditions with a single correct answer; it does not establish clinical reliability. Model rankings are macro-averaged over the 93 datasets and are robust to the aggregation scheme and to language stratification, but they are Chinese-dominant, with 99.9% of questions in Chinese, and English results are reported separately rather than used to rank models. Our temporal analysis shows that model rankings are consistent across examination years, which supports the stability of the ordering but does not establish that benchmark contamination has been eliminated; the possibility that examination content entered model training data cannot be excluded. The error taxonomy derived from LLM-as-judge analysis is exploratory, validated against human reviewers on a 100-case sample with 87% agreement. None of the evaluated models has undergone prospective clinical validation, and any use in patient care would require safety assessment, bias evaluation, regulatory approval, and appropriate human oversight.

Beyond technical contributions, our findings have significant implications for medical examination integrity [11,12], demonstrating that current examination formats are highly vulnerable to AI assistance and supporting recommendations for enhanced proctoring in high-stakes assessments. Additionally, our comprehensive model characterization provides the foundation for designing effective multi-LLM ensemble systems [17,55] that leverage complementary model strengths.

These findings provide actionable guidance for healthcare institutions selecting open-source AI models based on specific requirements for accuracy, speed, privacy, and computational resources. As reasoning-enhanced architectures continue to advance, the gap between open-source and proprietary medical AI may continue to narrow, enabling broader access to AI-assisted healthcare globally, including for non-English-speaking populations [9,10].

## Supporting information

S1 File Supporting appendices A–D. Appendix A: Complete model reference (28 tables, including all 172 evaluated models and checkpoint identifiers). Appendix B: Dataset descriptions and case studies. Appendix C: Evaluation prompts. Appendix D: Visualization results (117 figures).
(PDF)
