# Supplementary material for: OpenMedEval: Benchmarking 172 open-source LLMs on 326K medical questions across 93 datasets
Source: PLoS One. 2026 Nov 13;21(11):e0361996. doi: 10.1371/journal.pone.0361996 (PMC13766258; doi:10.1371/journal.pone.0361996)
Supplement: Supplementary file 1 [file pone.0361996.s001.pdf]

Supporting Information

OpenMedEval: Benchmarking 172 Open-Source LLMs on 326K Medical Questions Across 93 Datasets

S1 File. Supporting appendices A–D.

Appendix A: Complete Model Reference

This appendix provides a comprehensive reference of all 172 distinct models evaluated in this benchmark study, organized by developer and model family, with key specifications including parameter counts, release dates, and notable features. Note that the model variant table at the end includes some naming variations and quantization variants, but the primary benchmark results are reported for 172 unique model configurations.

DeepSeek Models

DeepSeek is a Chinese AI company that has developed a series of large language models known for their strong reasoning capabilities and cost-effective training. Table S1 summarizes the key DeepSeek models included in this benchmark.

Table S1: DeepSeek Model Family

**Model**	**Parameters**	**Release**	**Key Features**
DeepSeek-LLM	7B, 67B	2024	Base LLM, bilingual (EN/ZH), 2T tokens
DeepSeek-V2	16B, 236B	May 2024	MoE, Multi-head Latent Attention (MLA)
DeepSeek-V2.5	236B	2024	Improved V2 with enhanced capabilities
DeepSeek-V3	671B (37B active)	Dec 2024	MoE, 128K context, trained for $6M
DeepSeek-V3.1	840B	Aug 2025	Hybrid model, thinking/non-thinking modes
DeepSeek-R1	1.5B–671B	Jan 2025	Reasoning-focused, matches OpenAI o1

Meta Llama Models

Meta's Llama series represents one of the most influential open-source LLM families, with each generation bringing significant architectural improvements. Table S2 summarizes the Llama variants included in this benchmark.

Alibaba Qwen Models

Qwen (Tongyi Qianwen) is Alibaba Cloud's flagship LLM series, known for strong bilingual capabilities in Chinese and English. Table S3 summarizes the Qwen models evaluated in this benchmark.

Table S2: Meta Llama Model Family

Model	Parameters	Release	Key Features
Llama 2	7B, 13B, 70B	Jul 2023	4K context, RLHF fine-tuned
Llama 3	8B, 70B	Apr 2024	8K context, improved tokenizer
Llama 3.1	8B, 70B, 405B	Jul 2024	128K context, multilingual, tool use
Llama 3.2	1B, 3B, 11B, 90B	Sep 2024	First multimodal Llama (vision)
Llama 3.3	70B	Dec 2024	405B performance at 70B size
Llama 4 Scout	17B active/109B total	Apr 2025	MoE, 10M context window
Llama 4 Maverick	17B active/400B total	Apr 2025	MoE, 1M context, multimodal

Table S3: Alibaba Qwen Model Family

Model	Parameters	Release	Key Features
Qwen	0.5B–110B	2023–2024	Bilingual base models
Qwen2	0.5B–72B	Jun 2024	Improved architecture
Qwen2.5	0.5B–72B	Sep 2024	Added 3B, 14B, 32B variants
Qwen2.5-VL	3B–72B	Jan 2025	Vision-language models
QWQ-32B	32B	Nov 2024	Reasoning model, Apache 2.0
Qwen3	0.6B–235B	Apr 2025	Dense & MoE, 36T tokens, 119 languages

Mistral AI Models

Mistral AI, a French company founded in 2023, has developed efficient models known for their strong performance relative to size. Table S4 lists the Mistral family models evaluated in this benchmark.

Microsoft Phi Models

Microsoft's Phi series demonstrates that smaller models with high-quality training data can achieve remarkable performance. Table S5 lists the Phi models evaluated in this benchmark.

Google Gemma Models

Gemma is Google's family of lightweight open models, built from Gemini research and technology. Table S6 lists the Gemma models evaluated in this benchmark.

Cohere Command Models

Cohere's Command series focuses on enterprise applications with strong instruction-following and RAG capabilities. Table S7 lists the Cohere models included in this benchmark.

Table S4: Mistral AI Model Family

Model	Parameters	Release	Key Features
Mistral 7B	7B	Sep 2023	Grouped-Query Attention, Sliding Window
Mixtral 8x7B	45B (12B active)	Jan 2024	Sparse MoE, outperforms Llama 2 70B
Mixtral 8x22B	141B (39B active)	Apr 2024	64K context, SMoE architecture
Mistral Small	22B–24B	Feb 2024	Enterprise-grade, cost-efficient
Mistral Large	123B	Feb 2024	Flagship model, high-complexity tasks
Mistral Nemo	12B	Jul 2024	NVIDIA collaboration, multilingual
Magistral Small	24B	Jun 2025	Reasoning model, 128K context
Devstral	24B	May 2025	Coding-focused, Apache 2.0

Table S5: Microsoft Phi Model Family

Model	Parameters	Release	Key Features
Phi-2	2.7B	Dec 2023	Textbook-quality data training
Phi-3	3.8B, 14B	Apr 2024	Strong reasoning capabilities
Phi-3.5	3.8B	Aug 2024	Improved instruction following
Phi-4	14B	Dec 2024	Complex reasoning, math excellence
Phi-4-mini	3.8B	Feb 2025	Efficient, grouped-query attention
Phi-4-multimodal	5.6B	Feb 2025	Vision capabilities
Phi-4-reasoning	14B	May 2025	Rivals larger models on reasoning

Table S6: Google Gemma Model Family

Model	Parameters	Release	Key Features
Gemma	2B, 7B	Feb 2024	Lightweight, responsible AI
Gemma 2	2B, 9B, 27B	Jun 2024	Improved performance
Gemma 3	270M, 1B, 4B, 12B, 27B	Mar 2025	Multimodal, 128K context, 140+ languages
Gemma 3n	Various	2025	Mobile-first, edge deployment

Table S7: Cohere Command Model Family

Model	Parameters	Release	Key Features
Command-R	35B	Mar 2024	128K context, 10 languages
Command-R+	104B	Apr 2024	Enterprise-scale, RAG-optimized
Command-R7B	7B	2024	Lightweight variant
Command-A	111B	Mar 2025	256K context, 150% higher throughput

TII Falcon Models

The Technology Innovation Institute (TII) in UAE developed the Falcon series, known for efficient training on the RefinedWeb dataset. Table S8 lists the Falcon models evaluated in this benchmark.

Table S8: TII Falcon Model Family

Model	Parameters	Release	Key Features
Falcon	7B	2023	Multi-query attention
Falcon 2	11B	May 2024	5.5T tokens, VLM variant
Falcon 3	1B, 3B, 7B, 10B	Dec 2024	14T tokens, 32K context

LG AI Research EXAONE Models

LG AI Research developed EXAONE, with EXAONE Deep specifically optimized for reasoning tasks. Table S9 lists the EXAONE models evaluated in this benchmark.

Table S9: LG EXAONE Model Family

Model	Parameters	Release	Key Features
EXAONE 3.5	2.4B, 7.8B, 32B	Dec 2024	Improved instruction compliance
EXAONE Deep	2.4B, 7.8B, 32B	Mar 2025	Reasoning-optimized, math/code focus

Bespoke Labs OpenThinker Models

OpenThinker is developed by the Open Thoughts team (Bespoke Labs and DataComp community) for open-source reasoning. Table S10 lists the OpenThinker models evaluated in this benchmark.

Table S10: OpenThinker Model Family

Model	Parameters	Release	Key Features
OpenThinker-7B	7B	Jan 2025	Fine-tuned from Qwen2.5
OpenThinker-32B	32.8B	Feb 2025	16K context, math/code reasoning
OpenThinker2	7B, 32B	Apr 2025	Outperforms DeepSeek-R1-32B

Deep Cogito Models

Deep Cogito develops hybrid reasoning models using Iterated Distillation and Amplification (IDA). Table S11 lists the Cogito models evaluated in this benchmark.

Table S11: Cogito Model Family

Model	Parameters	Release	Key Features
Cogito v1	3B, 8B, 14B, 32B, 70B	Apr 2025	Hybrid reasoning, 128K context
Cogito v2.1	671B (MoE)	2025	Best US open-weight model claimed

Shanghai AI Lab InternLM Models

InternLM is developed by Shanghai AI Lab in collaboration with SenseTime, CUHK, and Fudan University. Table S12 lists the InternLM models evaluated in this benchmark.

Table S12: InternLM Model Family

Model	Parameters	Release	Key Features
InternLM2	1.8B, 7B, 20B	Jan 2024	Math-focused variants available
InternLM2.5	1.8B, 7B, 20B	Jul–Aug 2024	1M context support
InternLM3	8B	Jan 2025	4T tokens, 75% cost reduction

01.AI Yi Models

Yi is developed by 01.AI with strong bilingual (English/Chinese) capabilities. Table S13 lists the Yi models evaluated in this benchmark.

Table S13: 01.AI Yi Model Family

Model	Parameters	Release	Key Features
Yi	6B, 34B	Nov 2023	3.1T tokens, bilingual, Apache 2.0
Yi-9B	9B	Mar 2024	Best code/math in Yi series
Yi-VL	6B, 34B	Jan 2024	Vision-language models

Zhipu AI GLM Models

ChatGLM/GLM-4 is developed by Zhipu AI (Z.ai), originating from Tsinghua University. Table S14 lists the GLM models evaluated in this benchmark.

IBM Granite Models

IBM's Granite series focuses on enterprise applications with strong safety measures. Table S15 lists the Granite models evaluated in this benchmark.

Allen AI OLMo Models

OLMo (Open Language Model) emphasizes open science and full reproducibility. Table S16 lists the OLMo models evaluated in this benchmark.

Table S14: Zhipu AI GLM Model Family

Model	Parameters	Release	Key Features
ChatGLM3	6B	2023	Bilingual, dialogue-optimized
GLM-4	9B	Jan 2024	128K context, tool use
GLM-4-9B-Chat-1M	9B	2024	1M context length

Table S15: IBM Granite Model Family

Model	Parameters	Release	Key Features
Granite 3.0	1B, 2B, 3B, 8B	Oct 2024	Dense & MoE, 12T tokens
Granite 3.1	1B–8B	Dec 2024	128K context, embedding models
Granite 3.2	2B, 8B	Feb 2025	Chain-of-thought reasoning

Specialized Reasoning Models

Additional reasoning-focused models evaluated in this benchmark are listed in Table S17.

NVIDIA Models

NVIDIA's language models focus on enterprise deployment and optimization. Table S18 lists the NVIDIA models evaluated in this benchmark.

Moonshot AI Models

Moonshot AI developed Moonlight using the novel Muon optimizer. Table S19 lists the Moonlight model evaluated in this benchmark.

Dolphin Models

Dolphin is a fine-tuned model series by Eric Hartford, known for uncensored responses. Table S20 lists the Dolphin models evaluated in this benchmark.

Compact and Edge Models

Models optimized for resource-constrained environments are listed in Table S21.

Table S16: Allen AI OLMo Model Family

Model	Parameters	Release	Key Features
OLMo	1B, 7B	Feb 2024	Fully open: data, code, weights
OLMo 2	1B, 7B, 13B, 32B	Nov 2024–May 2025	5T tokens, staged training
OLMo 3	7B, 32B	Nov 2025	Think variant for reasoning

Table S17: Other Reasoning Models

Model	Developer	Parameters	Release	Key Features
SmallThinker-3B	PowerInfer	3B	2025	Draft model for QwQ-32B
DeepScaleR-1.5B	Agentica/UC Berkeley	1.5B	Feb 2025	Beats OpenAI o1 on AIME
Marco-O1	Alibaba AIDC	7B	Nov 2024	MCTS integration, CoT fine-tuning

Table S18: NVIDIA Model Family

Model	Parameters	Release	Key Features
Nemotron-70B	70B	Oct 2024	Based on Llama 3.1, RLHF trained
Nemotron-mini	4B	2024	Efficient deployment

Table S19: Moonshot AI Model Family

Model	Parameters	Release	Key Features
Moonlight-16B	16B (3B active)	Feb 2025	MoE, Muon optimizer, 5.7T tokens

Table S20: Dolphin Model Family

Model	Parameters	Release	Key Features
Dolphin-Mistral	7B	2024	Based on Mistral 0.2
Dolphin-Mixtral	8x7B, 8x22B	2024	Coding-focused, uncensored
Dolphin-Llama3	8B, 70B	2024	Based on Llama 3
Dolphin 3.0	8B	2024	Agentic, function calling
MegaDolphin	120B	2024	Extended context, emotional intelligence
TinyDolphin	1.1B	2024	Based on TinyLlama

Table S21: Compact Model Family

Model	Developer	Parameters	Release	Key Features
TinyLlama	J. Zhang et al.	1.1B	Sep 2023	3T tokens, Llama 2 architecture
SmolLM	Hugging Face	135M, 360M, 1.7B	Jul 2024	Edge deployment
SmolLM2	Hugging Face	135M, 360M, 1.7B	Nov 2024	11T tokens, state-of-the-art small
Lucie-7B	OpenLLM-France	7B	Jan 2025	French-focused, 3T tokens

Legacy and Baseline Models

Older models included for baseline comparison are listed in Table S22.

Table S22: Legacy Models

Model	Developer	Parameters	Release	Key Features
GPT-2	OpenAI	124M–1.5B	2019	Autoregressive baseline
Vicuna	LMSYS	7B, 13B, 33B	Mar 2023	ShareGPT fine-tuned

Complete Model Variant List

Table S23 provides a complete enumeration of all 172 evaluated models, organized alphabetically by model identifier.

Note: All models are evaluated using their official releases from HuggingFace Hub or original developer repositories. For reproducibility, Table S24 specifies the exact checkpoint identifiers for key models.

Complete model identifiers for all 172 evaluated models are provided in our code repository.

Appendix B: Dataset Descriptions and Case Studies

This appendix provides detailed descriptions of all medical benchmark datasets used in our evaluation, along with representative case studies demonstrating the complexity and clinical reasoning required for each examination type.

International Medical Benchmarks

AJKD: American Journal of Kidney Diseases

Source: American Journal of Kidney Diseases Clinical Case Studies
Domain: Nephrology
Question Type: Complex clinical vignettes with multiple-choice questions
Language: English
Difficulty: Expert-level nephrology knowledge

Description: This dataset consists of clinical case studies published in the American Journal of Kidney Diseases, one of the premier nephrology journals. Questions involve complex patient scenarios requiring integration of pathophysiology, clinical presentation, laboratory interpretation, and treatment decision-making in kidney diseases.

Table S23: All 172 Evaluated Models (alphabetical)

chatglm3_6b	cogito_3b	cogito_8b	cogito_14b
cogito_32b	cogito_70b	command-a_111b	command-r_35b
command-r-plus_104b	command-r7b_7b	deepscaler_1.5b	deepseek-llm_7b
deepseek-llm_67b	deepseek-r1_1.5b	deepseek-r1_7b	deepseek-r1_8b
deepseek-r1_14b	deepseek-r1_32b	deepseek-r1_70b	deepseek-r1_671b
deepseek-v2_16b	deepseek-v2_236b	deepseek-v2.5_236b	deepseek-v3_671b
deepseek-v3.1_671b	devstral_24b	dolphin-llama3_8b	dolphin-llama3_70b
dolphin-mistral_7b	dolphin-phi_2.7b	dolphin3_8b	exaone-deep_2.4b
exaone-deep_7.8b	exaone-deep_32b	exaone3.5_2.4b	exaone3.5_7.8b
exaone3.5_32b	falcon_7b	falcon2_11b	falcon3_1b
falcon3_3b	falcon3_7b	falcon3_10b	gemma_2b
gemma_7b	gemma2_2b	gemma2_9b	gemma2_27b
gemma3_270m	gemma3_1b	gemma3_4b	gemma3_12b
gemma3_27b	gemma3n_e2b	gemma3n_e4b	glm4_9b
gpt-oss_20b	gpt-oss_120b	gpt2_124m	gpt2_355m
gpt2_774m	gpt2_1.5b	granite3-dense_2b	granite3-dense_8b
granite3-moe_1b	granite3-moe_3b	granite3.1-dense_2b	granite3.1-dense_8b
granite3.1-moe_1b	granite3.1-moe_3b	granite3.2_2b	granite3.2_8b
granite3.3_2b	granite3.3_8b	internlm2_1.8b	internlm2_7b
internlm2_20b	internlm2_1m	internlm2.5	internlm3-8b-instruct
llama2_7b	llama2_13b	llama2_70b	llama3_8b
llama3_70b	llama3.1_8b	llama3.1_70b	llama3.1_405b
llama3.2_1b	llama3.2_3b	llama3.2-vision_11b	llama3.2-vision_90b
llama3.3_70b	llama4_maverick	llama4_scout	Lucie-7B-Instruct
magistral_24b	marco-o1_7b	megadolphin_120b	mistral_7b
mistral-large_123b	mistral-nemo_12b	mistral-small_22b	mistral-small_24b
mistral-small3.1_24b	mistral-small3.2_24b	mistrallite_7b	mixtral_8x7b
mixtral_8x22b	moonlight_16b	nemotron_70b	nemotron-mini_4b
olmo2_7b	olmo2_13b	openthinker_7b	openthinker_32b
phi_2.7b	phi3_3.8b	phi3_14b	phi3.5_3.8b
phi4_14b	phi4-mini_3.8b	phi4-mini-reasoning_3.8b	phi4-reasoning_14b
qwen_0.5b	qwen_1.8b	qwen_4b	qwen_7b
qwen_14b	qwen_32b	qwen_72b	qwen_110b
qwen2_0.5b	qwen2_1.5b	qwen2_7b	qwen2_72b
qwen2.5_0.5b	qwen2.5_1.5b	qwen2.5_3b	qwen2.5_7b
qwen2.5_14b	qwen2.5_32b	qwen2.5_72b	qwen2.5vl_3b
qwen2.5vl_7b	qwen2.5vl_32b	qwen2.5vl_72b	qwen3_0.6b
qwen3_1.7b	qwen3_4b	qwen3_8b	qwen3_14b
qwen3_30b	qwen3_32b	qwen3_235b	qwen3-coder_480b
qwq_32b	smallthinker_3b	smollm_135m	smollm_360m
smollm_1.7b	smollm2_135m	smollm2_360m	smollm2_1.7b
tinydolphin_1.1b	tinyllama_1.1b	vicuna_7b	vicuna_13b
vicuna_33b	yi_6b	yi_9b	yi_34b

Table S24: Model Checkpoint Identifiers for Key Evaluated Models

**Model**	**HuggingFace Identifier / Source**
DeepSeek-R1-671B	deepseek-ai/DeepSeek-R1
DeepSeek-R1-70B	deepseek-ai/DeepSeek-R1-Distill-Llama-70B
DeepSeek-R1-32B	deepseek-ai/DeepSeek-R1-Distill-Qwen-32B
QWQ-32B	Qwen/QwQ-32B-Preview
Phi4-Reasoning-14B	microsoft/Phi-4-reasoning
Qwen3-235B	Qwen/Qwen3-235B-A22B
Llama-3.1-405B	meta-llama/Llama-3.1-405B-Instruct
ExaOne-Deep-7.8B	LGAI-EXAONE/EXAONE-Deep-7.8B
Magistral-24B	mistralai/Magistral-Small-2506
OpenThinker-7B	open-thoughts/OpenThinker-7B

Case Study: Septic Shock with Volume Management

Clinical Scenario: A 54-year-old woman with type 2 diabetes was admitted to the medical intensive care unit for septic shock due to ascending cholangitis. She received 3 liters of intravenous fluids and was treated with piperacillin/tazobactam and norepinephrine. An endoscopic retrograde cholangiopancreatography was performed successfully with choledocholithiasis retrieval on hospital day 3. Her blood pressure improved to 98/55 by day 4, and norepinephrine was discontinued. On her current examination, she has pitting edema of her upper and lower extremities. She has no jugular venous distention, her lungs are clear, and she has 3-second capillary refill. Her urine output has been at least 0.5 mL/kg per hour.
Question: What is the mechanism of the most likely cause of extracellular volume excess in this patient?
Options:

(A) Decreased cardiac inotropy

(B) Increased antidiuretic hormone secretion

(C) Increased capillary permeability

(D) Decreased oncotic pressure

Answer: (C) Increased capillary permeability

USMLE: United States Medical Licensing Examination

Source: USMLE Step 1/2/3 Practice Questions
Domain: General Medicine (all specialties)
Question Type: Clinical vignettes with single best answer
Language: English
Difficulty: Medical licensing standard

Description: The USMLE dataset contains questions spanning all major medical specialties, testing basic science knowledge, clinical reasoning, and patient management skills. Questions are designed to assess readiness for independent medical practice in the United States.

Case Study: Infective Endocarditis Management

Clinical Scenario: A 67-year-old woman with congenital bicuspid aortic valve is admitted to the hospital because of a 2-day history of fever and chills. Current medication is lisinopril. Temperature is 38.0°C (100.4°F), pulse is 90/min, respirations are 20/min, and blood pressure is 110/70 mm Hg. Cardiac examination shows a grade 3/6 systolic murmur that is best heard over the second right intercostal space. Blood culture grows viridans streptococci susceptible to penicillin. In addition to penicillin, an antibiotic synergistic to penicillin is administered that may help shorten the duration of this patient's drug treatment.

Question: Which of the following is the most likely mechanism of action of this additional antibiotic on bacteria?

Options:

(A) Binding to DNA-dependent RNA polymerase

(B) Binding to the 30S ribosomal protein

(C) Competition with p-aminobenzoic acid

(D) Inhibition of dihydrofolate reductase

(E) Inhibition of DNA gyrase

Answer: (B) Binding to the 30S ribosomal protein (Aminoglycosides)

Chinese Clinical Practitioner Licensing Examination

The Chinese Clinical Practitioner Examination (执业医师资格考试) is the national medical licensing examination in China, required for all physicians to practice medicine legally. We include examinations from 2017 to 2024.

Chinese Physician Licensing Exam 2017–2024

Source: National Medical Licensing Examination of China
Domain: General Clinical Medicine
Question Type: Multiple-choice (single and multiple answer)
Language: Chinese (translated to English in dataset)
Coverage: 8 annual examinations (2017–2024)
Difficulty: Medical licensing standard

Description: These datasets contain questions from China's national physician licensing examinations, covering fundamental medical sciences, clinical medicine, preventive medicine, and medical ethics. The examination is required for medical graduates to obtain their practicing license.

Case Study: Biochemistry and Pharmacology

Question: The chemical bond that can maintain the higher structure of protein molecules is:
Options:

(A) Ionic bond
(B) Hydrogen bond
(C) Hydrophobic bond
(D) Salt bond
(E) Peptide bond

Answer: (B) Hydrogen bond
Analysis: The molecular structure of protein is divided into primary, secondary, tertiary and quaternary structures, the latter three are collectively referred to as higher structure. The chemical bond that can maintain the higher structure of protein molecules (including secondary, tertiary and quaternary structures) is hydrogen bond.

Attending Physician Specialty Examinations

The Attending Physician Examination (主治医师考试) is an intermediate-level professional qualification in China, required for physicians to advance from resident to attending physician status. We include 22 specialty-specific examinations, listed in Table S25.

Attending Physician: Neurology (神经内科)

Domain: Neurology
Level: Intermediate Professional Qualification
Question Type: Multiple-choice with detailed analysis

Case Study: Headache Classification

Question: Migraine is classified as:
Options:

(A) Traumatic headache
(B) Myogenic headache
(C) Reflex headache
(D) Vascular headache
(E) Psychogenic headache

Answer: (D) Vascular headache
Analysis: Migraine is classified as a vascular headache, characterized by recurrent episodes of pulsating head pain often associated with autonomic symptoms, resulting from neurovascular dysfunction.

Table S25: Attending Physician (主治医师) Specialty Examination Datasets

**Specialty (English)**	**Specialty (Chinese)**	**Domain**
Clinical Laboratory	临床检验	Laboratory Medicine
Chief Pharmacist	主任药师	Pharmacy
Infectious Diseases	传染病	Internal Medicine
Pediatrics	儿科	Pediatrics
Internal Medicine	内科	Internal Medicine
Respiratory Medicine	呼吸内科	Internal Medicine
Obstetrics & Gynecology	妇产科	OB/GYN
Medical Imaging	影像医学	Radiology
Emergency Medicine	急诊	Emergency
Nursing	护理	Nursing
Radiation Medicine	放射医学	Radiology
Epidemiology	流行病学	Public Health
Pathology	病理	Pathology
Dermatology & Venereology	皮肤性病	Dermatology
Ophthalmology	眼科	Ophthalmology
Neurology	神经内科	Neurology
Otolaryngology	耳鼻喉	ENT
Medical Oncology	肿瘤内科	Oncology
Nutrition	营养	Nutrition
Ultrasound Medicine	超声医学	Radiology
Rheumatology & Immunology	风湿免疫	Internal Medicine
Orthopedics	骨科	Surgery

Attending Physician: Rheumatology & Immunology (风湿免疫)

Domain: Rheumatology and Clinical Immunology
Level: Intermediate Professional Qualification
Focus Areas: Autoimmune diseases, inflammatory arthritis, connective tissue disorders

Case Study: Proteinuria in Autoimmune Disease

Question: Which of the following is a disease that is unlikely to cause massive proteinuria?
Options:

(A) Systemic lupus erythematosus

(B) Diabetic nephropathy

(C) Gouty kidney

(D) Henoch-Schönlein purpura nephritis

(E) Renal amyloidosis

Answer: (C) Gouty kidney
Analysis: Gouty kidney (uric acid nephropathy) results from hyperuricemia causing renal damage. Unlike SLE, diabetic nephropathy, Henoch-Schönlein purpura nephritis, and renal amyloidosis—which commonly cause massive proteinuria through glomerular injury—gouty kidney typically presents with mild proteinuria due to tubulointerstitial rather than glomerular damage.

Associate Chief Physician Specialty Examinations

The Associate Chief Physician Examination (副高/副主任医师考试) is an advanced professional qualification in China, representing senior specialist status. We include 28 specialty-specific examinations, listed in Table S26.

Associate Chief Physician: Cardiovascular Medicine (心血管内科)

Domain: Cardiovascular Internal Medicine
Level: Senior Professional Qualification
Focus Areas: Heart failure, coronary artery disease, arrhythmias, valvular disease

Table S26: Associate Chief Physician (副主任医师) Specialty Examination Datasets

**Specialty (English)**	**Specialty (Chinese)**	**Domain**
Clinical Medicine	临床医学	General Medicine
General Practice	全科	Primary Care
Endocrinology	内分泌	Internal Medicine
Dentistry/Oral Medicine	口腔	Dentistry
Pediatric Surgery	小儿外科	Surgery
Rehabilitation Medicine	康复医学	Rehabilitation
Cardiothoracic Surgery	心胸外科	Surgery
Cardiovascular Medicine	心血管内科	Internal Medicine
Emergency Medicine	急诊	Emergency
Plastic Surgery	整形外科	Surgery
General Surgery	普通外科	Surgery
Urology	泌尿外科	Surgery
Gastroenterology	消化内科	Internal Medicine
Burn Surgery	烧伤外科	Surgery
Pain Medicine	疼痛	Anesthesiology
Dermatology	皮肤性病	Dermatology
Neurology	神经内科	Neurology
Neurosurgery	神经外科	Surgery
Psychiatry	精神科	Psychiatry
Otolaryngology-Head & Neck	耳鼻喉头颈外科	ENT/Surgery
Occupational Medicine	职业病	Occupational Health
Nephrology	肾内科	Internal Medicine
Medical Oncology	肿瘤内科	Oncology
Surgical Oncology	肿瘤外科	Surgery
Radiation Oncology	肿瘤放疗	Oncology
Hematology	血液病	Internal Medicine
Critical Care Medicine	重症医学	Critical Care
Anesthesiology	麻醉	Anesthesiology

Case Study: Coronary Artery Disease Classification

Question: Variant angina pectoris is primarily caused by:
Options:

(A) Coronary artery atherosclerosis with stenosis ≥75%

(B) Coronary artery atherosclerosis with stenosis ≤50%

(C) Coronary artery spasm

(D) Complete occlusive thrombosis in the coronary artery

(E) Incomplete thrombosis due to ruptured coronary plaque

Answer: (C) Coronary artery spasm
Analysis: Variant angina (Prinzmetal's angina) is caused by coronary artery spasm rather than fixed atherosclerotic stenosis. It characteristically occurs at rest, often at night, and is associated with transient ST-segment elevation. In contrast, stable angina requires ≥75% stenosis, unstable angina involves plaque rupture with incomplete thrombosis, and acute MI involves complete thrombotic occlusion.

Associate Chief Physician: Nephrology (肾内科)

Domain: Nephrology
Level: Senior Professional Qualification
Focus Areas: Acute kidney injury, chronic kidney disease, glomerulonephritis, dialysis

Case Study: Diabetic Nephropathy Classification

Question: Early diabetic nephropathy is characterized by:
Options:

(A) Overflow proteinuria

(B) Microalbuminuria

(C) Macroalbuminuria

(D) Orthostatic proteinuria

(E) Tissue proteinuria

Answer: (B) Microalbuminuria
Analysis: Early diabetic nephropathy (also called the microalbuminuria stage) typically develops 10–15 years after diabetes onset. It is characterized by urinary albumin excretion of 30–300 mg/day, detectable only by sensitive assays. Progression to macroalbuminuria (>300 mg/day) indicates overt diabetic nephropathy. Overflow proteinuria occurs in conditions like multiple myeloma where abnormal proteins are filtered.

Chief Physician Specialty Examinations

The Chief Physician Examination (正高/主任医师考试) represents the highest level of professional medical qualification in China. We include 32 specialty-specific examinations, listed in Table S27.

Table S27: Chief Physician (主任医师) Specialty Examination Datasets

**Specialty (English)**	**Specialty (Chinese)**	**Domain**
Clinical Laboratory Testing	临床检验	Laboratory Medicine
Clinical Biochemistry	临床生化检验	Laboratory Medicine
Clinical Pharmacy	临床药学	Pharmacy
Clinical Nutrition	临床营养	Nutrition
Infectious Diseases	传染病	Internal Medicine
Child Health Care	儿童保健	Pediatrics
General Practice	全科医学	Primary Care
Internal Medicine Nursing	内科护理	Nursing
Hospital Pharmacy	医院药学	Pharmacy
Allergy/Hypersensitivity	变态反应	Immunology
Surgical Nursing	外科护理	Nursing
Women's Health	妇女保健	OB/GYN
School & Adolescent Health	学校卫生与儿少卫生	Public Health
Rehabilitation Medicine	康复医学	Rehabilitation
Emergency Nursing	急诊护理	Nursing
Nursing (General)	护理	Nursing
Radiation Medicine	放射医学	Radiology
Radiation Oncology	放射肿瘤	Oncology
General Internal Medicine	普通内科	Internal Medicine
Nuclear Medicine	核医学	Nuclear Medicine
Pain Medicine	疼痛医学	Anesthesiology
Disease Prevention & Control	疾病预防控制	Public Health
Pathology	病理	Pathology
Neurology	神经内科	Neurology
Psychiatry	精神病	Psychiatry
Geriatric Medicine	老年医学	Geriatrics
Occupational Medicine	职业病	Occupational Health
Surgical Oncology	肿瘤外科	Surgery
Nutrition & Food Hygiene	营养与食品卫生	Public Health
Ultrasound Medicine	超声医学	Radiology
Critical Care Medicine	重症医学	Critical Care
Anesthesiology	麻醉	Anesthesiology

Chief Physician: Nursing (护理)

Domain: Advanced Nursing Practice
Level: Senior Professional Qualification (Highest)
Focus Areas: Clinical nursing, patient safety, nursing management, education

Case Study: Postoperative Pain Management with PCIA

Clinical Scenario: A 68-year-old female was admitted for skin and sclera jaundice. Abdominal CT showed "pancreatic head space-occupying lesions" and she was scheduled for pancreaticoduodenectomy under general anesthesia. Patient-controlled intravenous analgesia (PCIA) was planned for postoperative pain management.
Question: Regarding the use of analgesia pumps, the content of preoperative education should include:
Options:

(A) Educate patients on possible adverse effects of postoperative pain on the body

(B) Inform patients of their right to a pain-free postoperative experience

(C) Emphasize that postoperative pain can be completely relieved

(D) Explain key points of using PCIA and teach patients to self-administer when they feel pain

(E) Inform patients how to express pain and precautions to take

(F) Avoid informing patients of analgesic adverse reactions to prevent psychological burden

Answer: (A), (B), (D), (E)
Analysis: Before implementing PCA analgesia, the principle and key points of the PCA pump should be introduced to patients and their families. Patients should be taught how to self-administer medication when they feel pain. The purpose of pain treatment is to eliminate or relieve pain—not guarantee complete relief (C is incorrect). Patients should be informed of adverse reactions to better cooperate with treatment (F is incorrect).

Chief Physician: Critical Care Medicine (重症医学)

Domain: Intensive Care Medicine
Level: Senior Professional Qualification (Highest)
Focus Areas: Mechanical ventilation, hemodynamic monitoring, sepsis, multi-organ failure

Case Study: Pain Assessment

Question: The following descriptions about pain are correct:
Options:

(A) Postoperative pain is a complex physiological and psychological response of the human body to tissue damage and repair process

(B) Pain is both a subjective discomfort and a protective mechanism

(C) Pain has two meanings: pain sensation and pain reaction

(D) Postoperative pain is a normal phenomenon

(E) Pethidine (meperidine) is the first choice for treatment of post-operative pain

Answer: (A), (B), (C)
Analysis: Pain involves both physiological and psychological components. While postoperative pain occurs commonly, it should not be accepted as "normal" without proper management (D incorrect). Pethidine is not first-line for postoperative pain due to its metabolite normeperidine causing neurotoxicity (E incorrect).

Health Classification Dataset

Health Information Classification Dataset (健康分类数据集)

Source: Health advice and lifestyle recommendations
Domain: Public Health / Health Literacy
Task Type: Binary classification (positive/negative health advice)
Language: Chinese (translated to English)

Description: This dataset contains health-related statements that need to be classified as either evidence-based positive health advice or potentially misleading health claims. This tests models' ability to distinguish between scientifically supported health recommendations and unsubstantiated claims.

Examples of Health Statements

Positive (Evidence-Based):

- "Use low-temperature cooking methods such as steaming, boiling and stewing to retain nutrients and reduce the production of harmful substances"
- "Learn the correct nursing skills for bedridden patients turning over"

Classification Task: Models must determine whether health advice statements are evidence-based (positive) or potentially misleading (negative).

Dataset Statistics Summary

Table S28 provides a consolidated summary of all 93 medical benchmark datasets organized by examination category.

The complete benchmark suite covers 93 distinct medical datasets spanning:

Table S28: Summary of All Medical Benchmark Datasets

Category	Dataset	Count	Description
International	AJKD	1	Nephrology case studies
	USMLE	1	US Medical Licensing Exam
Chinese Licensing	执业医师 (2017–2024)	8	Annual licensing examinations
Attending Physician	主治医师	22	Specialty examinations
Associate Chief	副主任医师	28	Senior specialty examinations
Chief Physician	主任医师	32	Highest-level specialty exams
Classification	健康分类	1	Health advice classification
**Total**		**93**	

- **2 international benchmarks** (English): AJKD nephrology cases and USMLE licensing questions
- **8 annual licensing examinations** (Chinese): Physician licensing exams from 2017–2024
- **82 specialty-specific examinations** (Chinese): Covering internal medicine, surgery, nursing, pharmacy, public health, and allied health fields
- **1 health classification task**: Binary classification of health advice quality

This comprehensive coverage enables evaluation of LLM medical capabilities across different languages, difficulty levels, and clinical specialties, providing insights into both general medical knowledge and specialty-specific expertise.

Appendix C: Evaluation Prompts

This section provides the complete prompt templates used for model evaluation. All models are evaluated using identical 5-shot chain-of-thought prompting to ensure fair comparison.

English Prompt Template (USMLE, AJKD)

5-Shot Medical QA Prompt (English)

```
You are a medical expert.  Answer the following
multiple-choice question by selecting the single best answer.
First, reason through the question step by step, then provide
your final answer as a single letter (A, B, C, D, or E).
[5 demonstration examples with reasoning chains]
Question:  {question}
```

```
Options:
A. {option_a}
B. {option_b}
C. {option_c}
D. {option_d}
E. {option_e}
Think step by step, then provide your answer.
```

Chinese Prompt Template (中文考试)

5-Shot Medical QA Prompt (Chinese)

```
你是一位医学专家。请回答以下选择题，选择最佳答案。首先逐步分析问题，然后给出你的最终答案（单个字母 A、B、C、D 或 E）。
[5 包含推理链的示例]
问题：{question}
选项：
A. {option_a}
B. {option_b}
C. {option_c}
D. {option_d}
E. {option_e}
请逐步思考，然后给出答案。
```

Answer Extraction

Model responses are processed through a structured extraction agent that identifies the final answer letter from the reasoning trace. The agent enforces single-letter output (A–E) and handles common response formats including "The answer is X", "Answer: X", and standalone letter responses. Responses that cannot be parsed to a valid option are marked as incorrect.

Appendix D: Visualization Results

This appendix presents comprehensive visualizations of benchmark results across all evaluated models and datasets.

Dataset Statistics

Figures S1–S5 present complementary statistical views of the 93 benchmark datasets: sample size distribution (Figure S1), mean token count per dataset (Figure S2), token count range (Figure S3), the aggregate token distribution across all questions (Figure S4), and the relationship between dataset size and average question length (Figure S5).

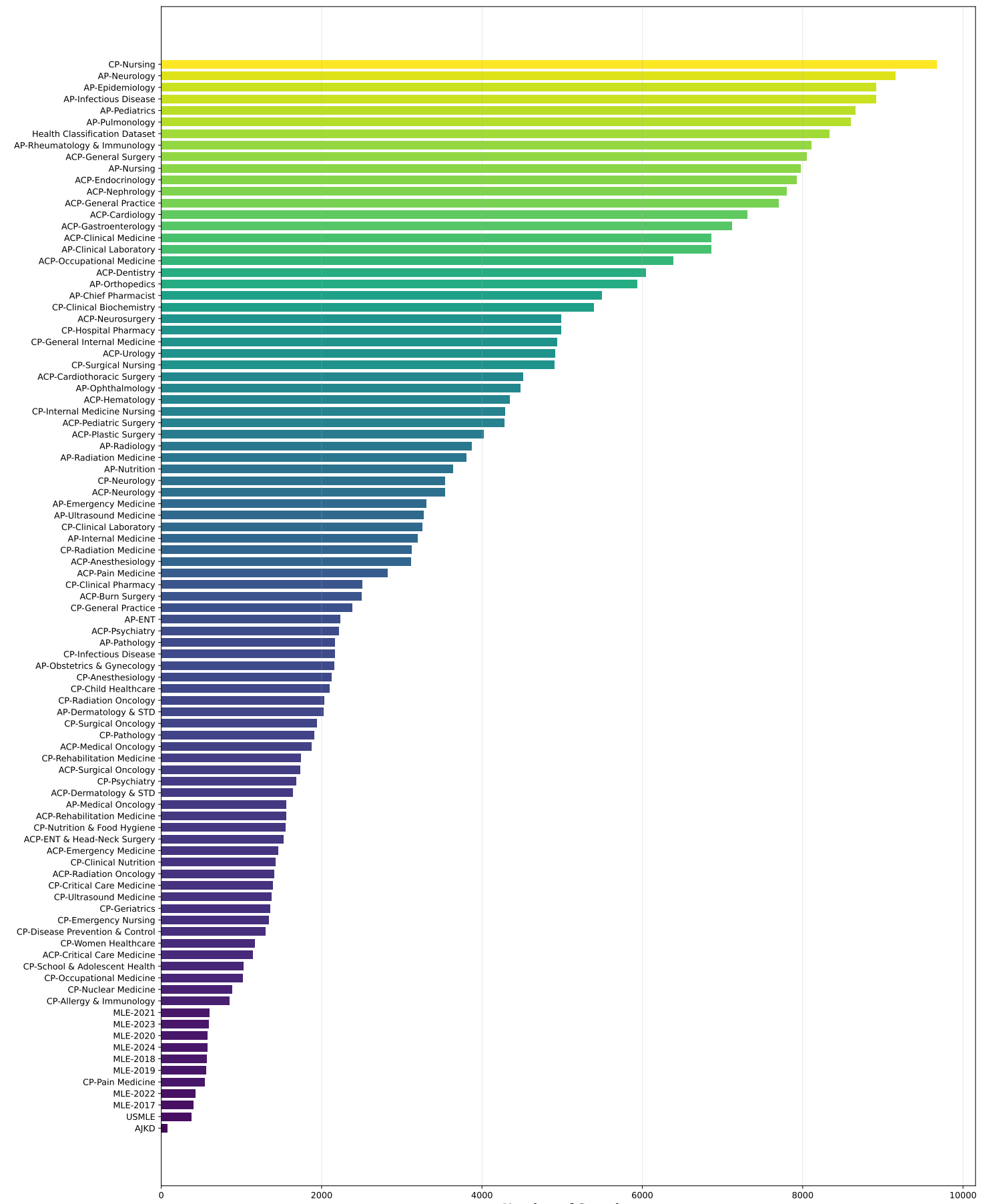

Figure S1: Horizontal bar chart displaying the sample size distribution across all 93 medical benchmark datasets, sorted in descending order by number of questions. The distribution reveals substantial heterogeneity, with the largest datasets containing over 10,000 samples (primarily from Associate Chief Physician and Chief Physician specialty examinations) while smaller datasets such as international benchmarks contain 1,000–3,000 samples. This size variation reflects the different availability and scope of medical examination materials across professional levels and specialties.

Performance Heatmaps

Figures S6–S11 present performance heatmaps across different dataset subsets: Medical Licensing Examination (Figure S6), Attending Physician (Figure S7), Associate Chief Physician (Figure S8), Chief Physician (Figure S9), international benchmarks (Figure S10), and cross-year performance

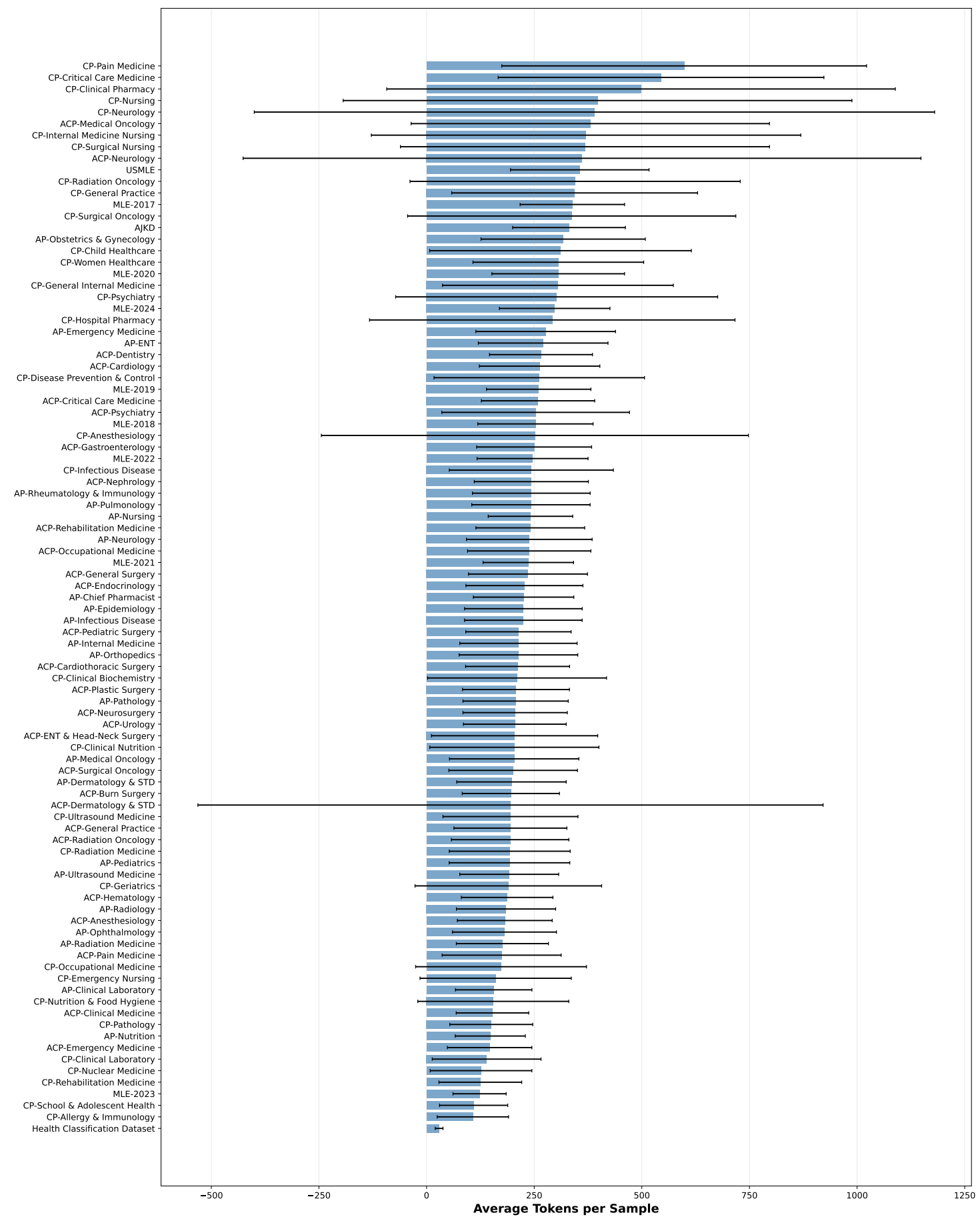

Figure S2: Bar chart presenting the average token count per sample for each of the 93 benchmark datasets, with error bars representing one standard deviation to indicate within-dataset variability. Token counts serve as a proxy for question complexity and context length, with advanced professional examinations (Chief Physician, Associate Chief Physician) typically featuring longer clinical vignettes averaging 200–400 tokens compared to 100–200 tokens for entry-level licensing examinations. The substantial standard deviations observed in many datasets reflect the mixture of brief knowledge-recall questions and extended case-based reasoning scenarios within each examination.

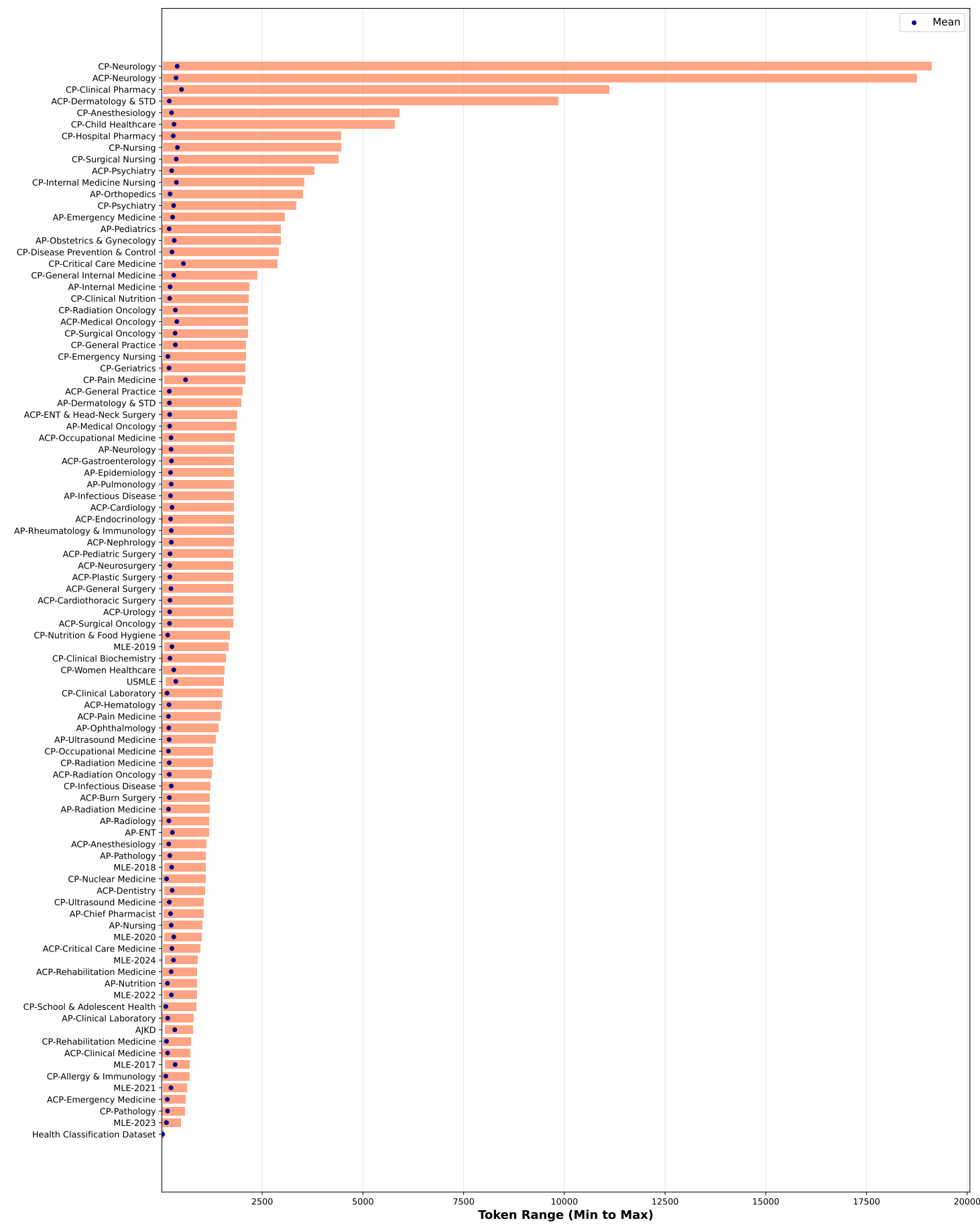

Figure S3: Range plot illustrating the minimum-to-maximum token count span for each benchmark dataset, with blue dots marking mean values and vertical bars showing the full range from shortest to longest questions. This visualization highlights the extreme variability within certain datasets, where some questions require only 50 tokens while extended clinical scenarios within the same examination may exceed 800 tokens. Understanding this within-dataset heterogeneity is essential for interpreting model performance, as longer questions typically demand more sophisticated reasoning chains and working memory capacity.

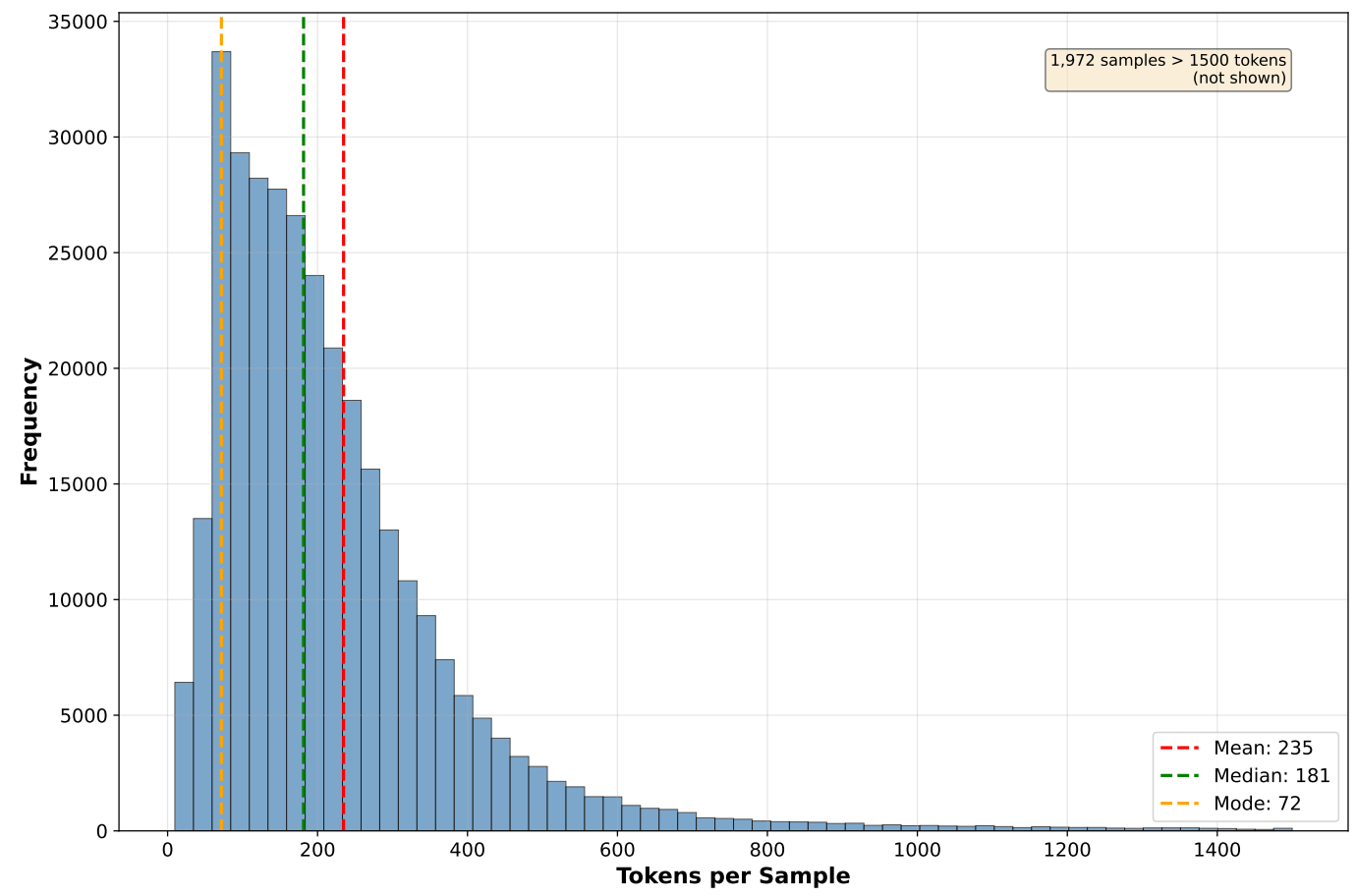

Figure S4: Histogram displaying the aggregate distribution of token counts across all 326,100 medical questions in the benchmark, with red and green dashed vertical lines indicating mean and median values respectively. The right-skewed distribution (mean > median) reflects the predominance of concise knowledge-assessment questions alongside a long tail of extended clinical vignettes requiring comprehensive patient history, laboratory findings, and multi-step diagnostic reasoning. The modal range of 100–200 tokens corresponds to typical single-concept medical questions, while the extended right tail represents complex case-based scenarios characteristic of advanced professional examinations.

correlations (Figure S11).

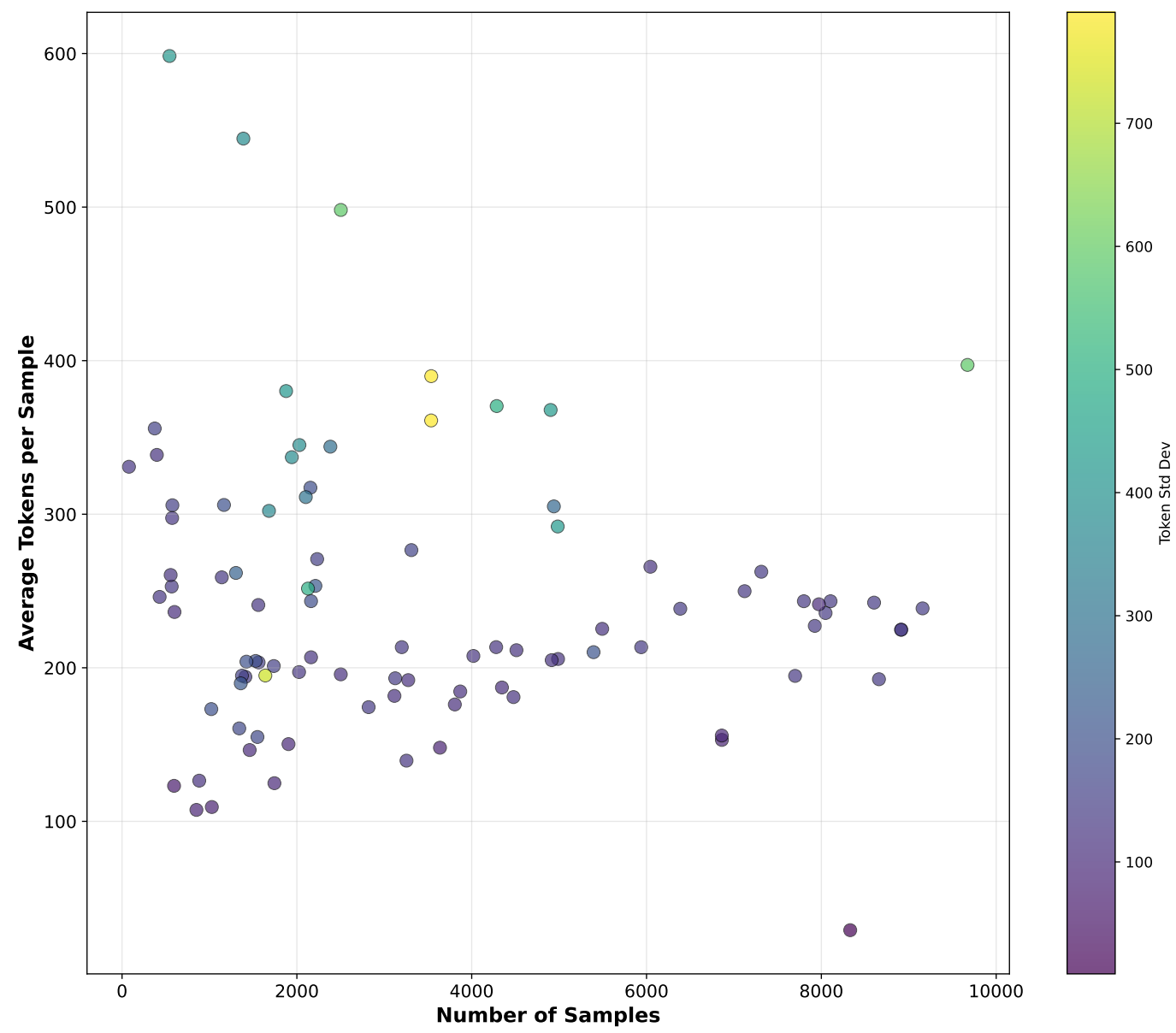

Figure S5: Scatter plot examining the relationship between dataset size (number of samples, x-axis) and average token count per sample (y-axis), with point color encoding token count standard deviation within each dataset. The absence of strong correlation suggests that dataset size and question complexity are largely independent characteristics determined by examination design rather than data collection constraints. Datasets with high color intensity (large standard deviation) represent examinations with heterogeneous question formats, mixing brief recall items with extended clinical cases.

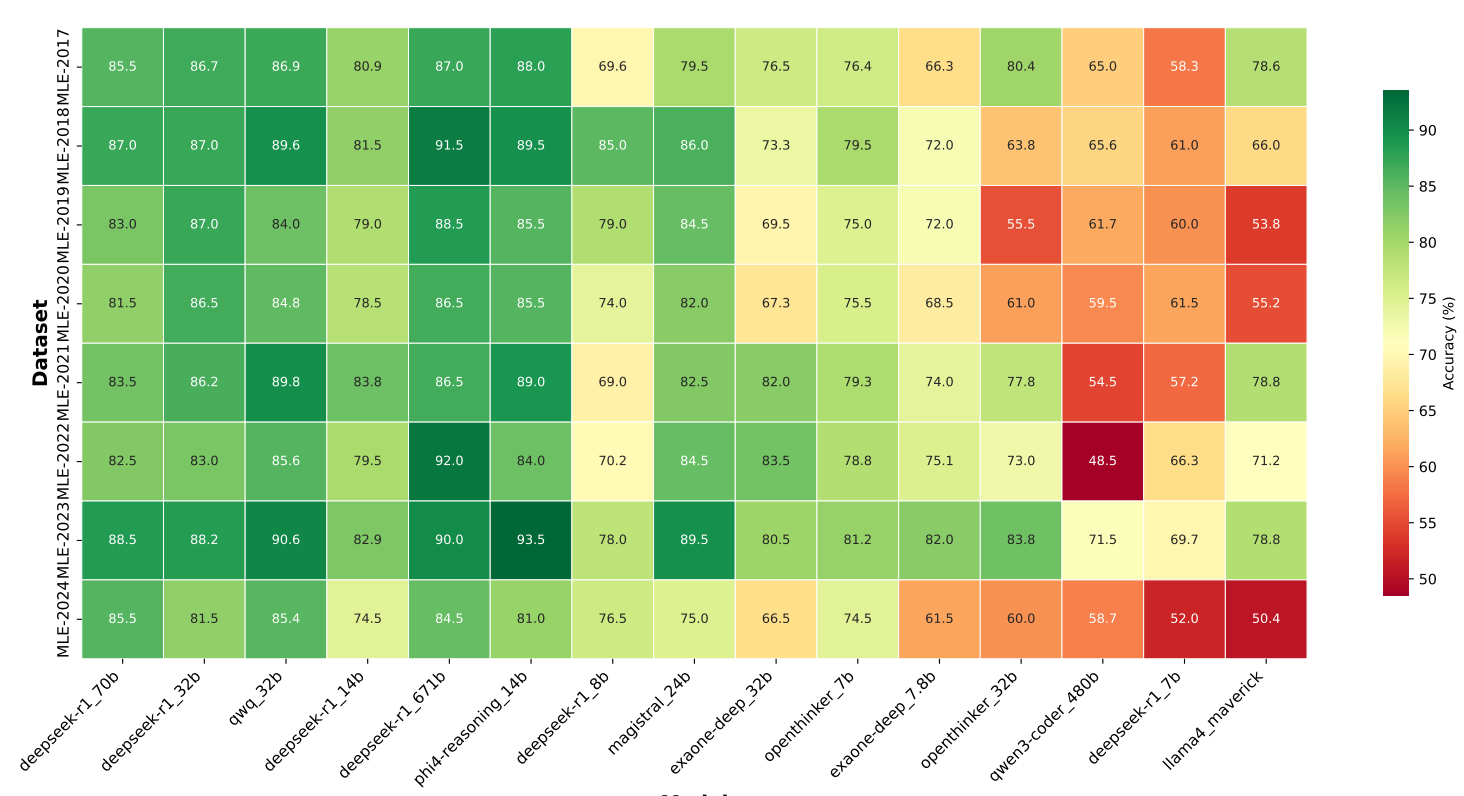

Figure S6: Performance heatmap visualizing model accuracy across Chinese Medical Licensing Examination (MLE) datasets from 2017 to 2024, with rows representing the top-performing models and columns representing annual examination editions. Color gradients range from red (low accuracy, below 50%) through yellow (moderate, 50–70%) to green (high accuracy, above 80%). The temporal consistency of model rankings across examination years suggests that the underlying medical knowledge requirements remain stable, while the gradual difficulty increase observable in recent years may reflect evolving examination standards or expanded content coverage.

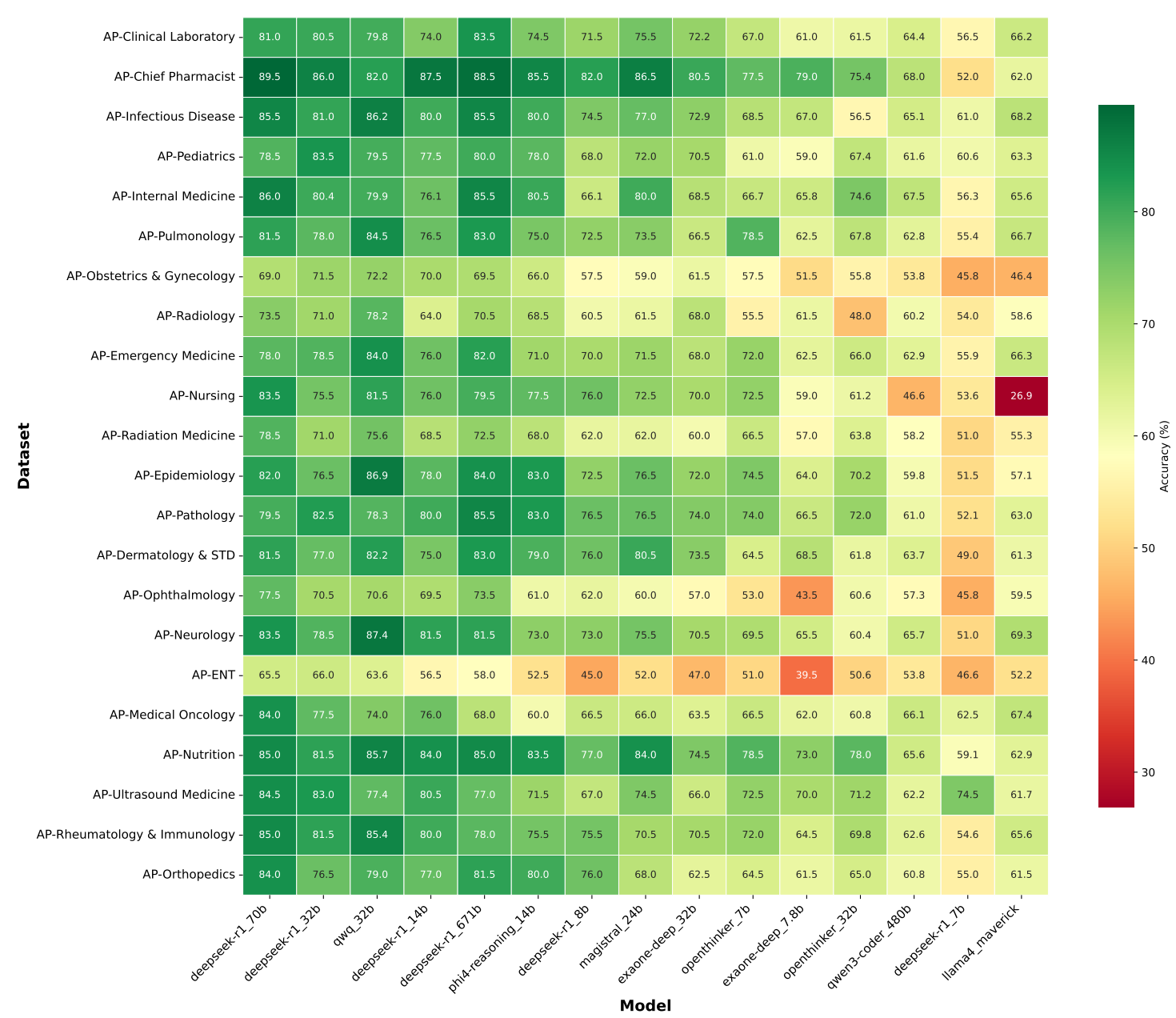

Figure S7: Performance heatmap displaying model accuracy across 22 Attending Physician (AP) specialty examination datasets, enabling identification of specialty-specific strengths and weaknesses for each model architecture. The visualization reveals that certain specialties (e.g., Internal Medicine, Pathology) consistently yield higher accuracy across models, while others (e.g., specialized surgical subspecialties) present more uniform challenges. Reasoning-enhanced models demonstrate more homogeneous color patterns across specialties, suggesting that explicit reasoning training produces more generalizable medical knowledge representations compared to specialty-dependent performance patterns observed in general-purpose models.

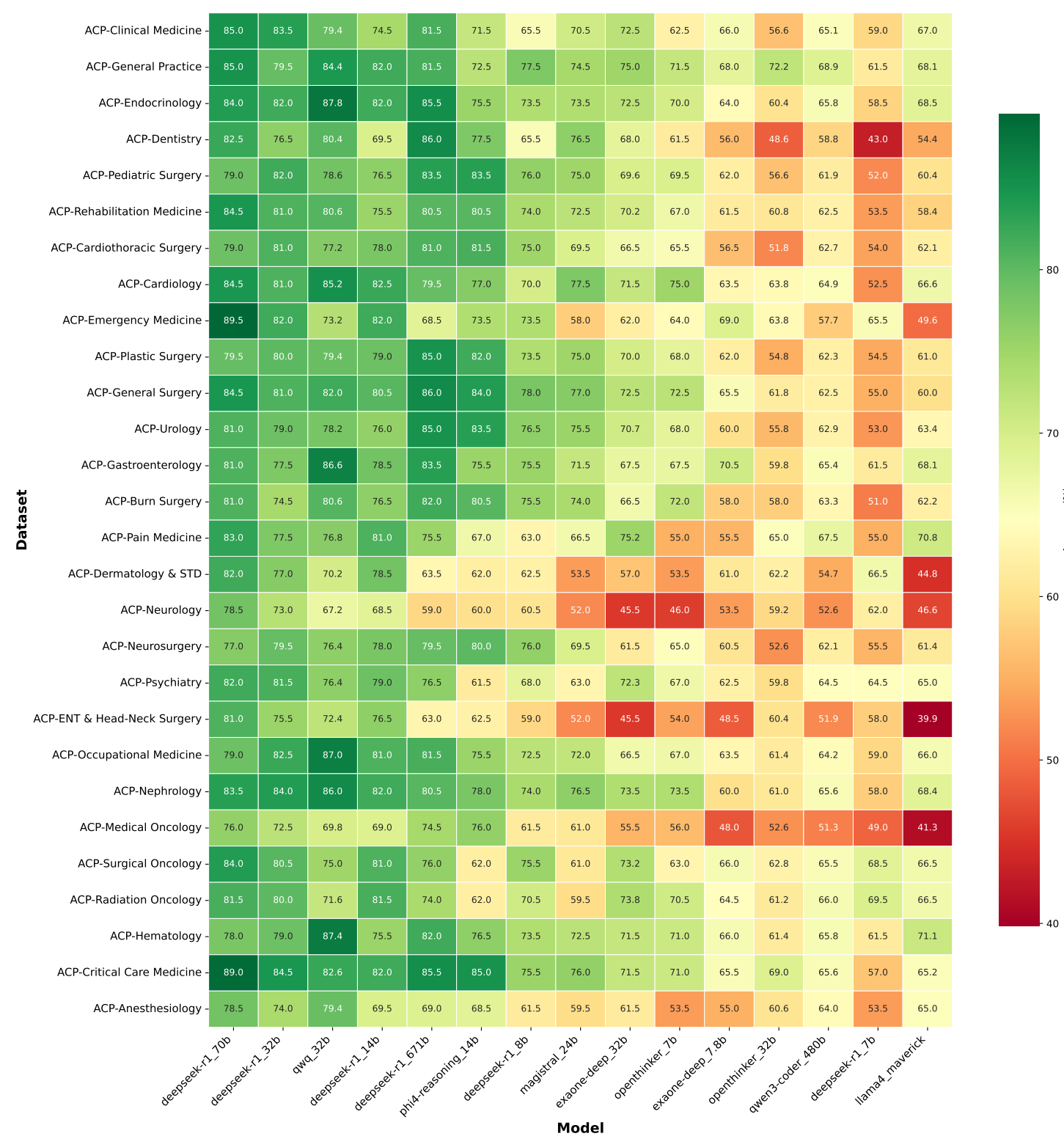

Figure S8: Performance heatmap presenting model accuracy across 28 Associate Chief Physician (ACP) specialty examination datasets, representing senior specialist-level medical knowledge. The ACP examinations feature more complex clinical scenarios with multi-step diagnostic reasoning requirements, and the heatmap reveals that performance gaps between reasoning-enhanced and general-purpose models widen at this professional level. Certain subspecialties cluster with similar difficulty profiles, potentially reflecting shared underlying clinical reasoning patterns or overlapping medical knowledge domains.

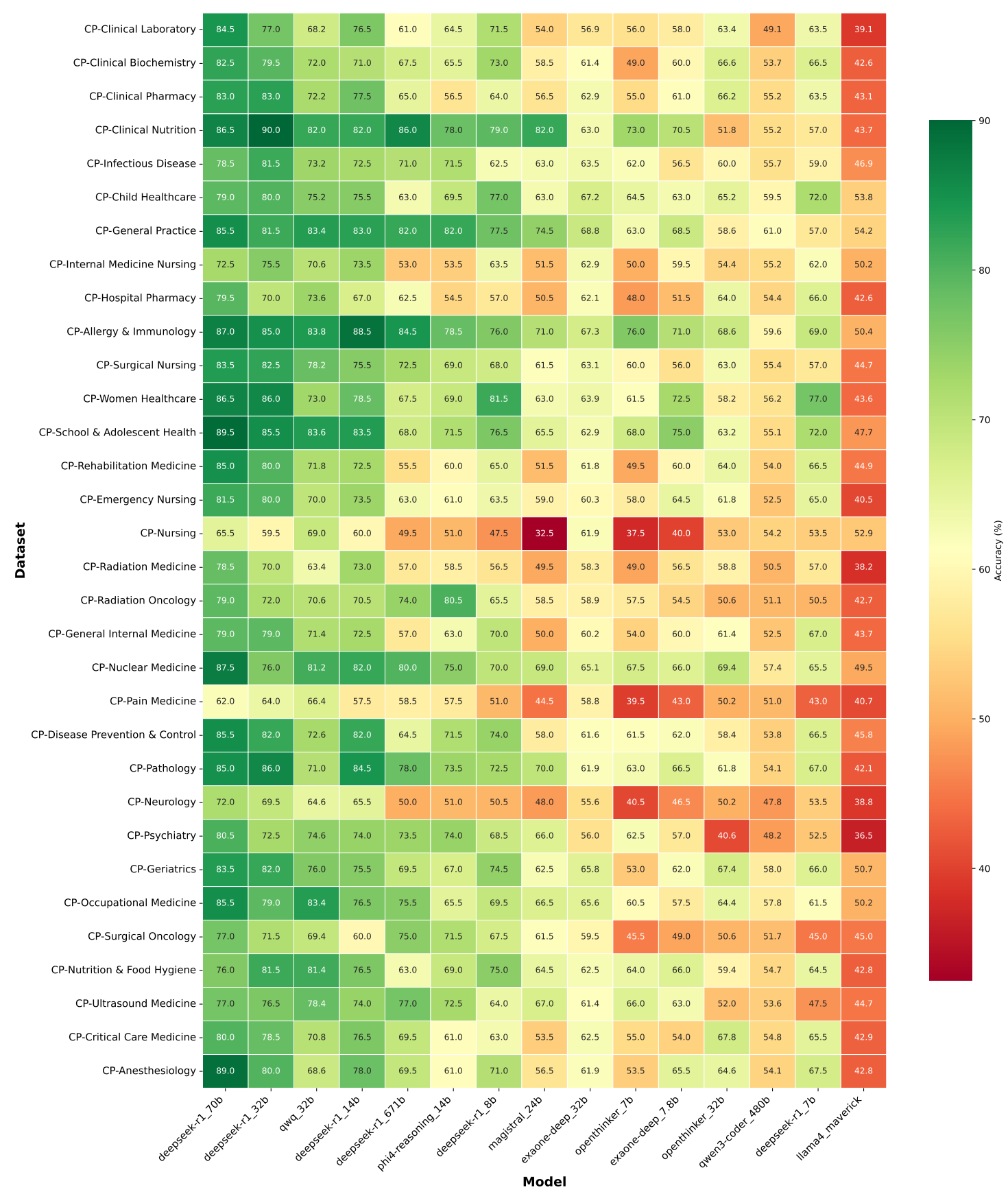

Figure S9: Performance heatmap illustrating model accuracy across 32 Chief Physician (CP) specialty examination datasets, representing the highest level of professional medical qualification in China. Despite the advanced difficulty level, reasoning-enhanced models maintain strong performance (predominantly green coloring), while general-purpose models show more variable performance with pronounced specialty-dependent patterns. The CP examinations require integration of extensive clinical experience with complex diagnostic reasoning, and the observed performance patterns suggest that reasoning-specialized training effectively captures these higher-order cognitive processes.

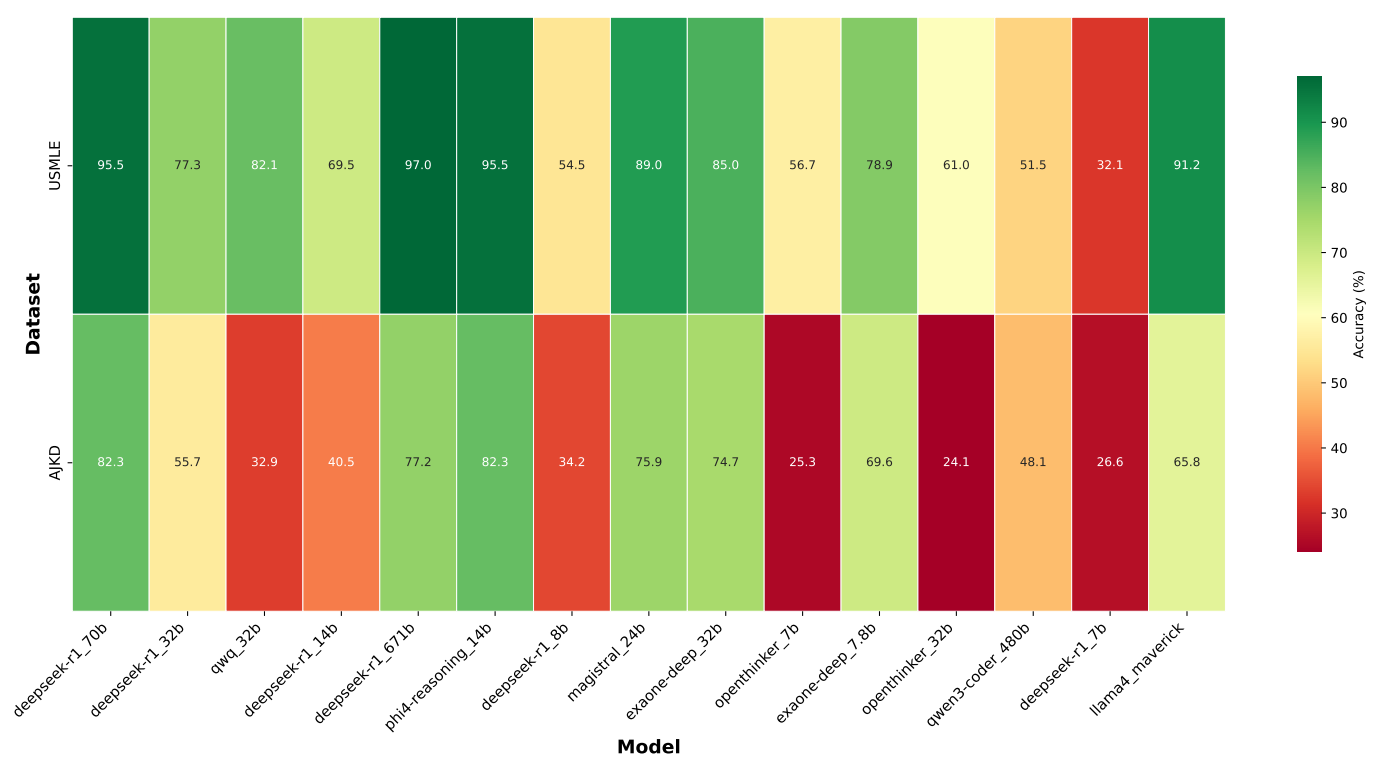

Figure S10: Performance heatmap comparing model accuracy on international English-language medical benchmarks: the United States Medical Licensing Examination (USMLE) and American Journal of Kidney Diseases (AJKD) nephrology cases. The USMLE column reveals near-ceiling performance for top reasoning models (approaching 97%), substantially exceeding the human passing threshold of 60%, while the specialized AJKD nephrology dataset presents more differentiated challenge with top models achieving approximately 82%. The cross-dataset comparison enables assessment of whether models achieving high USMLE scores maintain performance on subspecialty content requiring deeper domain expertise.

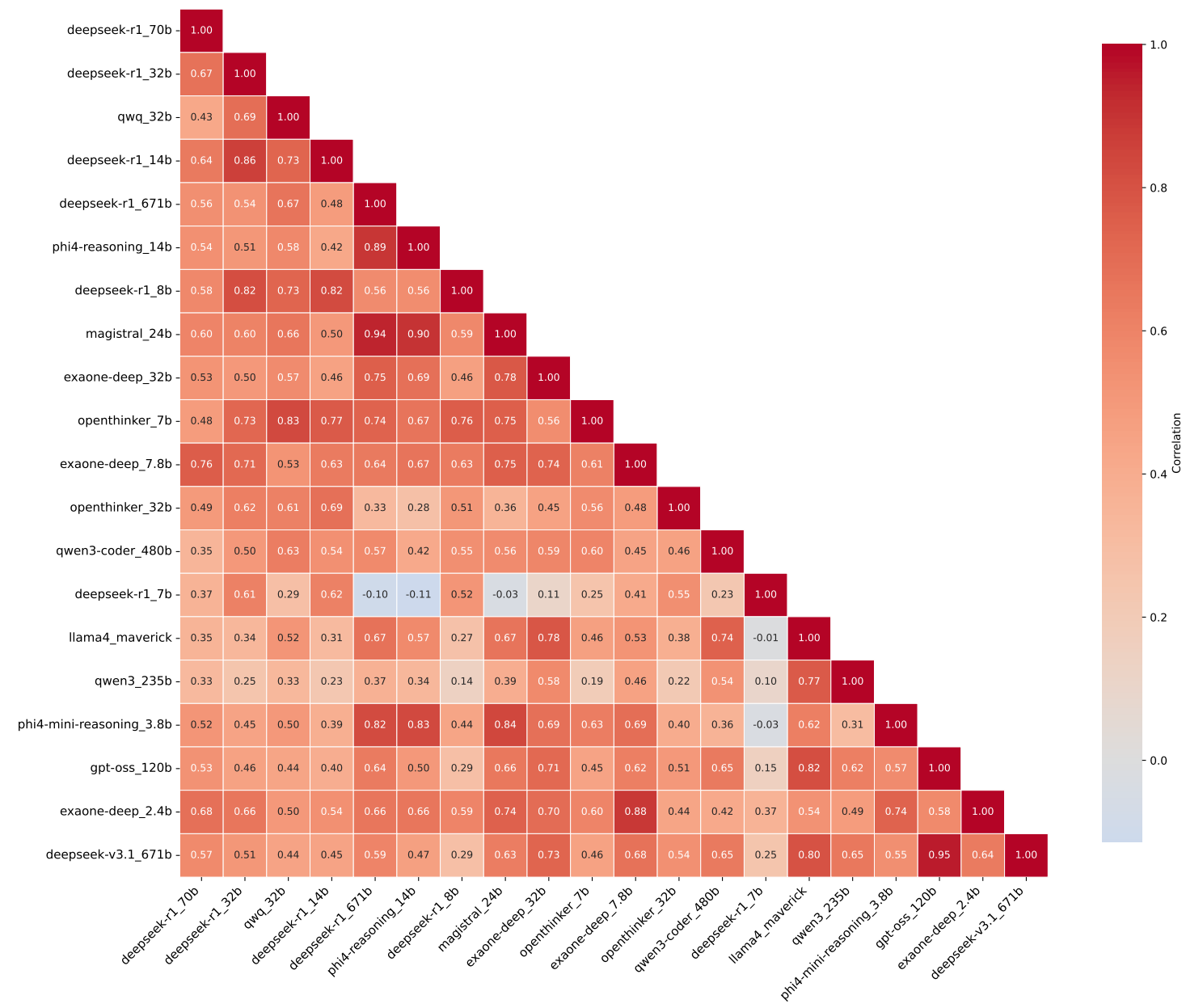

Figure S11: Symmetric correlation matrix quantifying pairwise performance similarity between the top 20 models based on their accuracy vectors across all 93 datasets. High correlation values (warm colors) between models suggest similar patterns of dataset-specific strengths and weaknesses, potentially reflecting shared architectural features or training methodologies. The block diagonal structure reveals clustering of models by family (e.g., DeepSeek-R1 variants exhibiting high mutual correlation), while off-diagonal patterns identify unexpected similarities between architecturally distinct models that may share underlying knowledge representations or reasoning strategies.

Multi-dimensional Performance Comparison

Figures S12–S16 present radar chart visualizations comparing top model performance profiles across different examination tiers and specialty axes: MLE (Figure S12), AP (Figure S13), ACP (Figure S14), CP (Figure S15), and DeepSeek-R1 intra-family scaling (Figure S16).

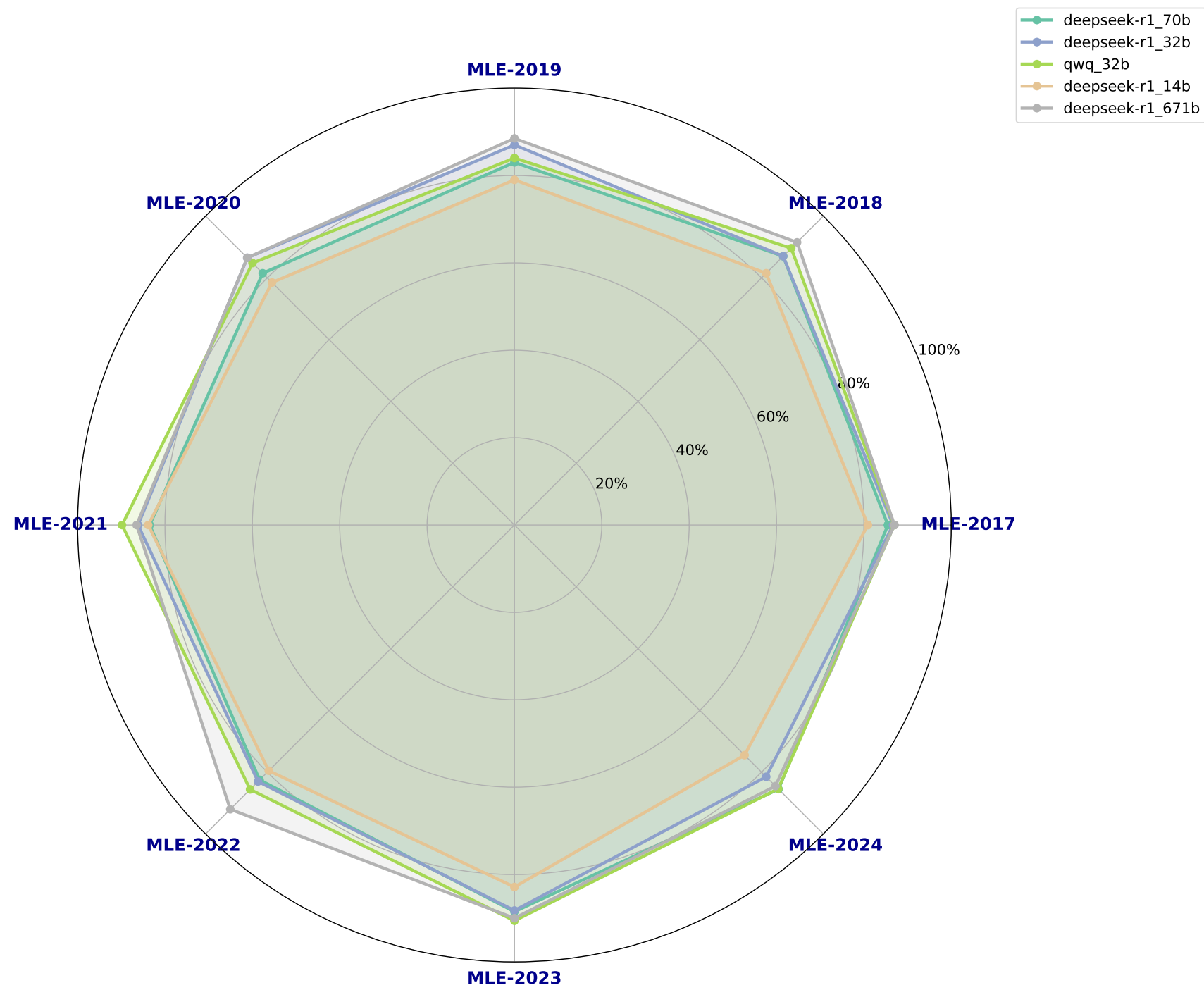

Figure S12: Multi-axis radar chart comparing the top 5 performing models across Medical Licensing Examination (MLE) datasets from 2017 to 2024, with each radial axis representing a different examination year and the distance from center indicating accuracy percentage. The near-complete overlap of reasoning-enhanced models (DeepSeek-R1, QWQ) across all axes demonstrates their consistent temporal performance, while any visible asymmetries would indicate year-specific difficulty variations or content changes that differentially affect model architectures.

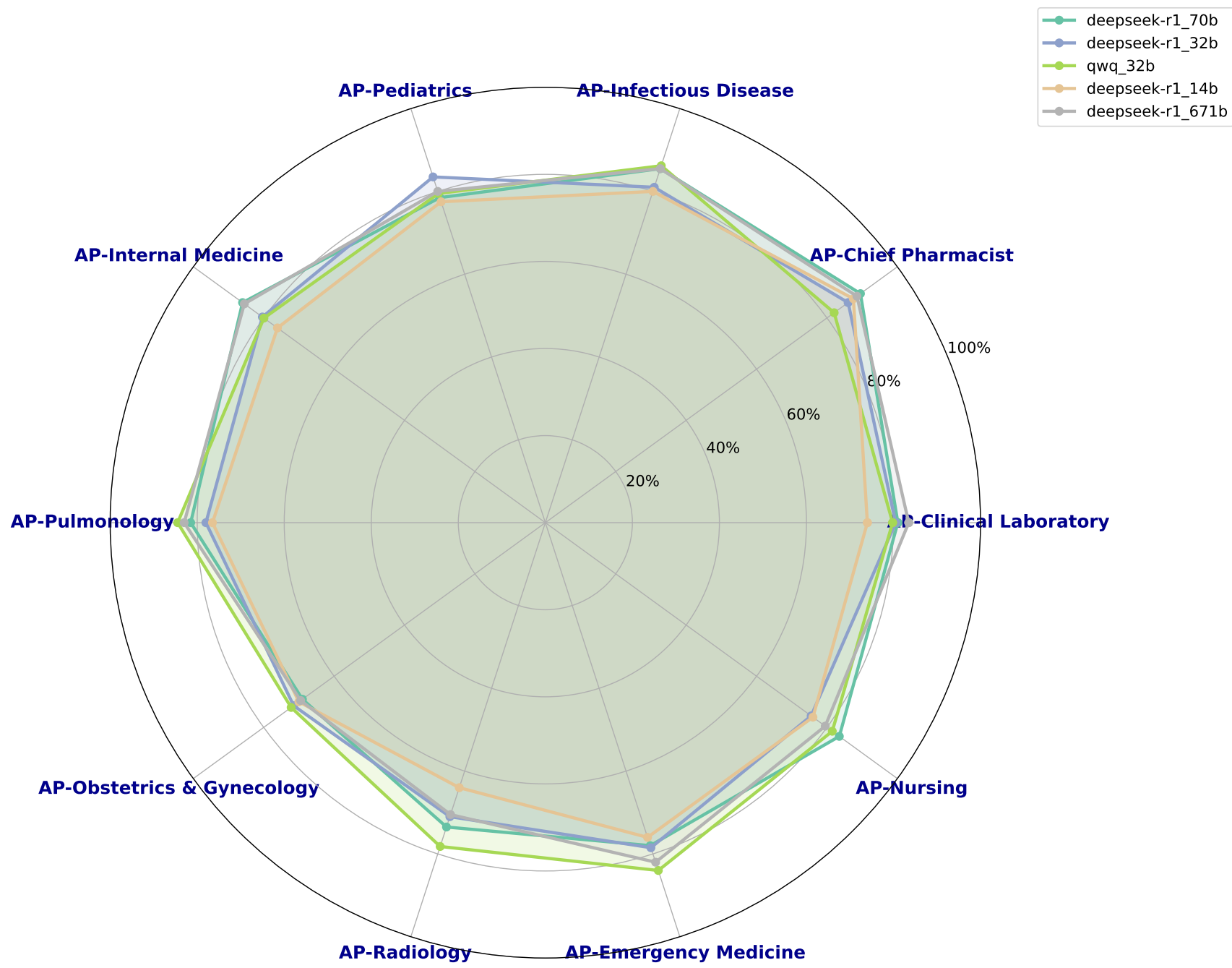

Figure S13: Radar chart visualization comparing the top 5 models across 22 Attending Physician (AP) specialty examination datasets, with each radial axis representing a distinct medical specialty. The polygon shapes reveal each model's specialty-specific performance profile: more circular shapes indicate uniform cross-specialty performance (characteristic of reasoning-enhanced models), while irregular polygons with pronounced vertices and indentations would suggest specialty-dependent strengths and weaknesses typical of models with uneven domain knowledge distribution.

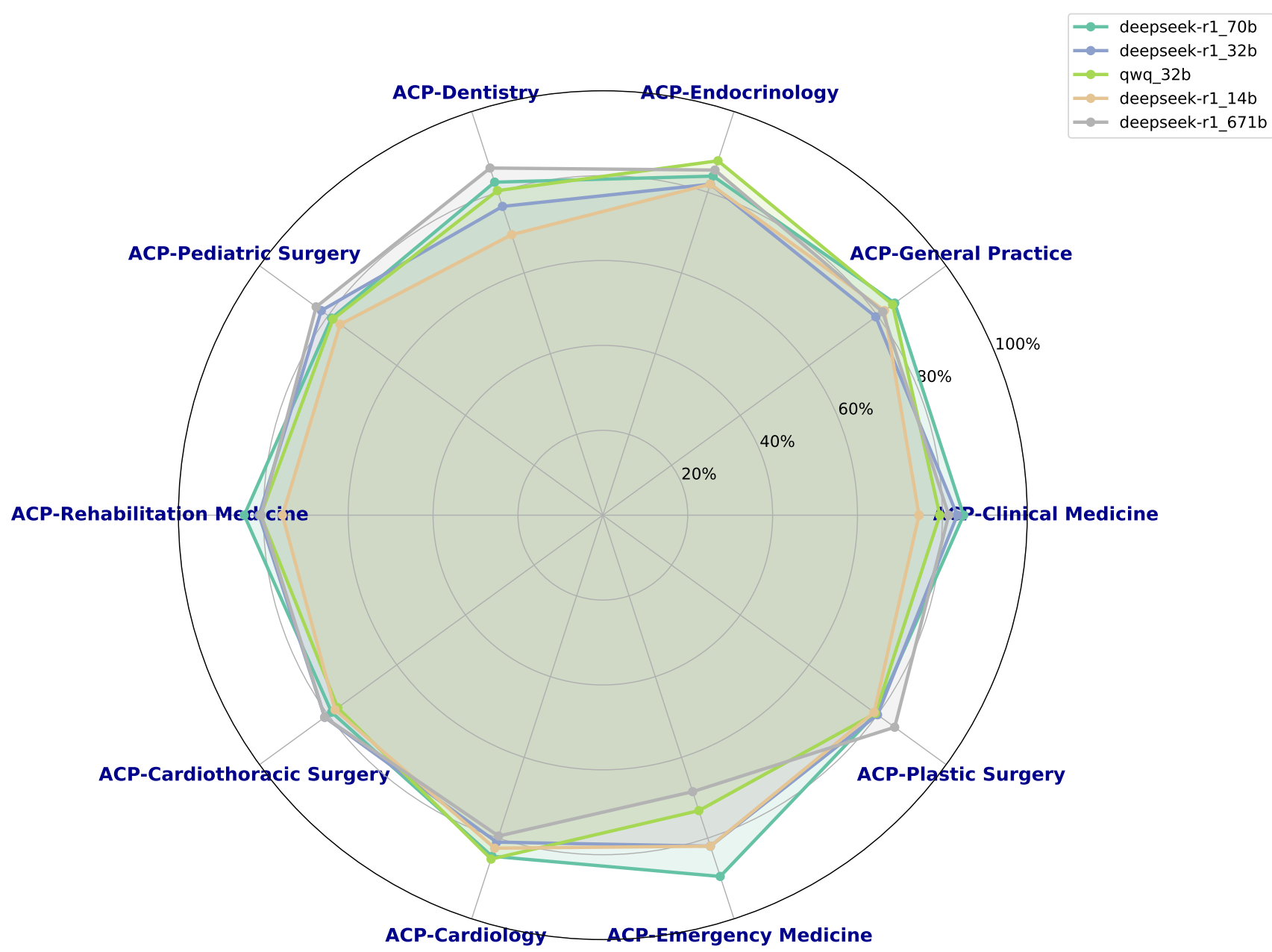

Figure S14: Radar chart displaying comparative performance of the top 5 models across 28 Associate Chief Physician (ACP) specialty examination datasets, representing senior-level medical expertise. The expanded number of axes compared to AP examinations reflects the finer subspecialty granularity at this professional level. Performance profiles at the ACP level provide insight into models' ability to handle advanced clinical scenarios requiring integration of specialist knowledge with complex multi-step diagnostic reasoning chains.

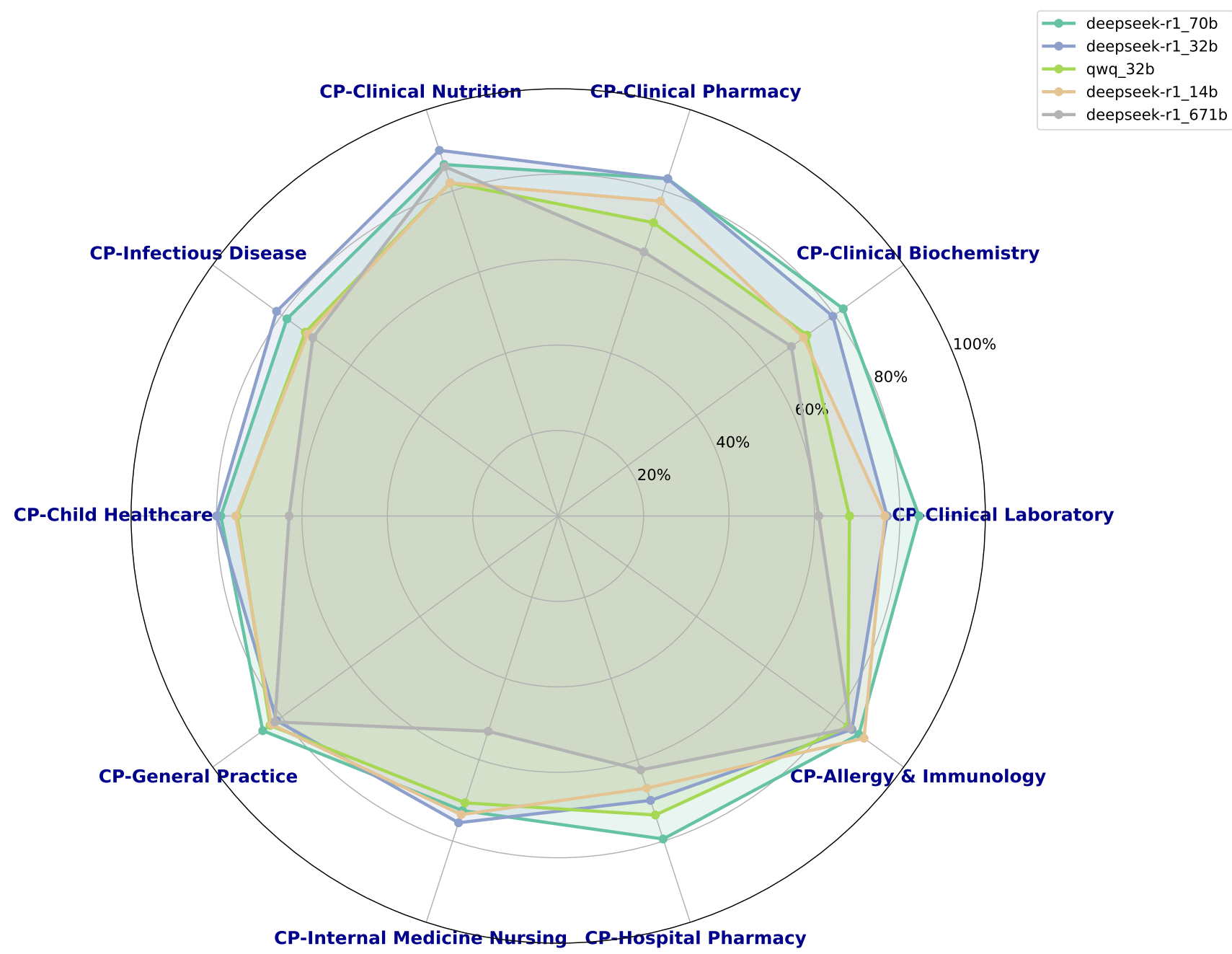

Figure S15: Radar chart presenting the performance profiles of top 5 models across 32 Chief Physician (CP) specialty examination datasets, representing the highest tier of medical professional qualification. The CP-level radar patterns reveal whether models maintaining strong Attending and Associate Chief Physician performance sustain their advantage at the most demanding professional level, or whether the increased complexity exposes limitations in certain specialty domains.

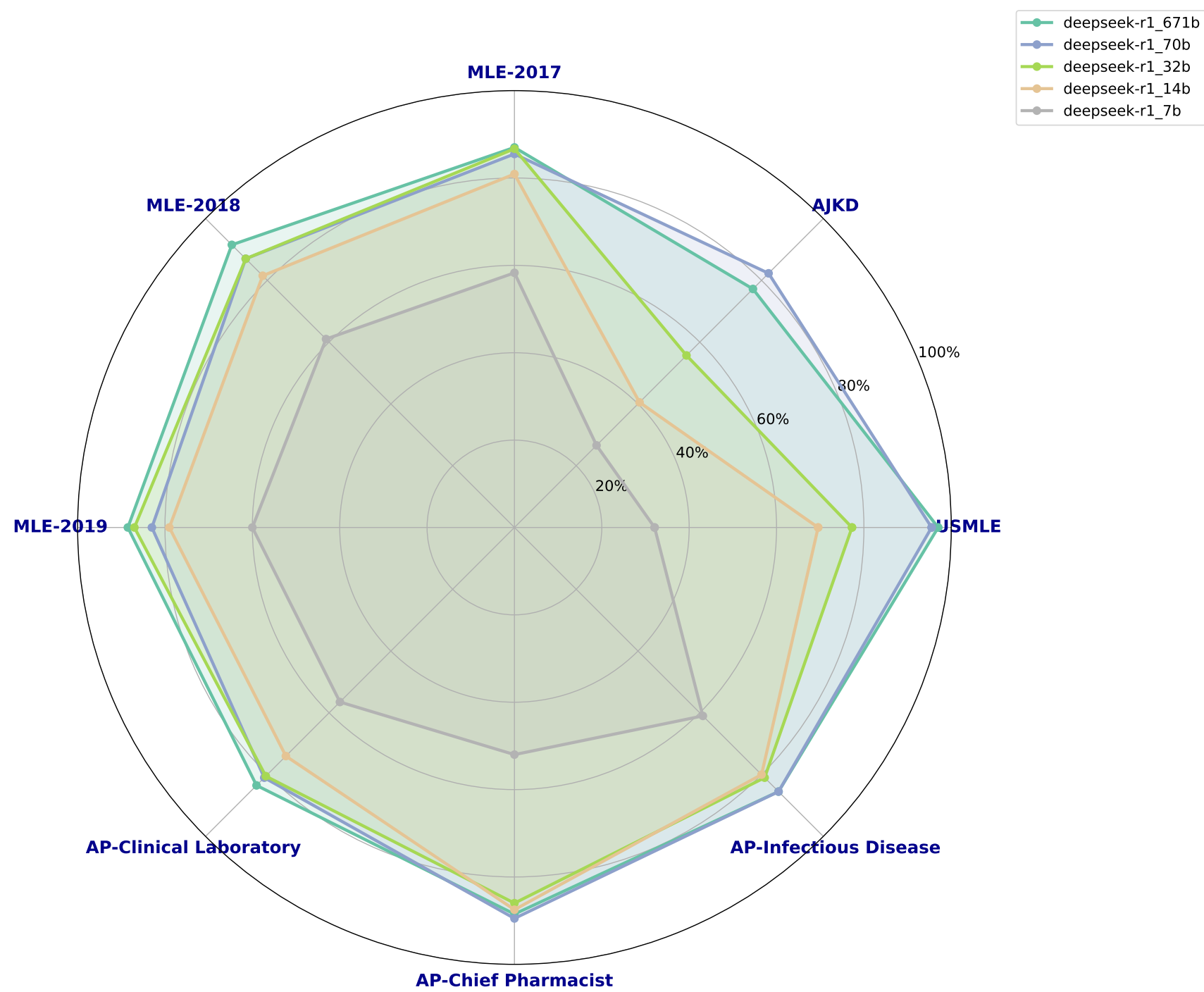

Figure S16: Intra-family scaling analysis radar chart comparing DeepSeek-R1 model variants across parameter scales (1.5B, 7B, 8B, 14B, 32B, 70B, 671B) on representative datasets spanning different examination categories and difficulty levels. This visualization enables direct assessment of scaling behavior within the reasoning-enhanced architecture family, revealing the non-monotonic relationship where the 70B variant often outperforms both smaller and larger variants, suggesting an optimal scale for current reasoning training methodologies in the medical domain.

Distribution Analysis

Figure S17 compares model accuracy distributions across examination categories; Figure S18 shows the full density distributions for the top 10 models.

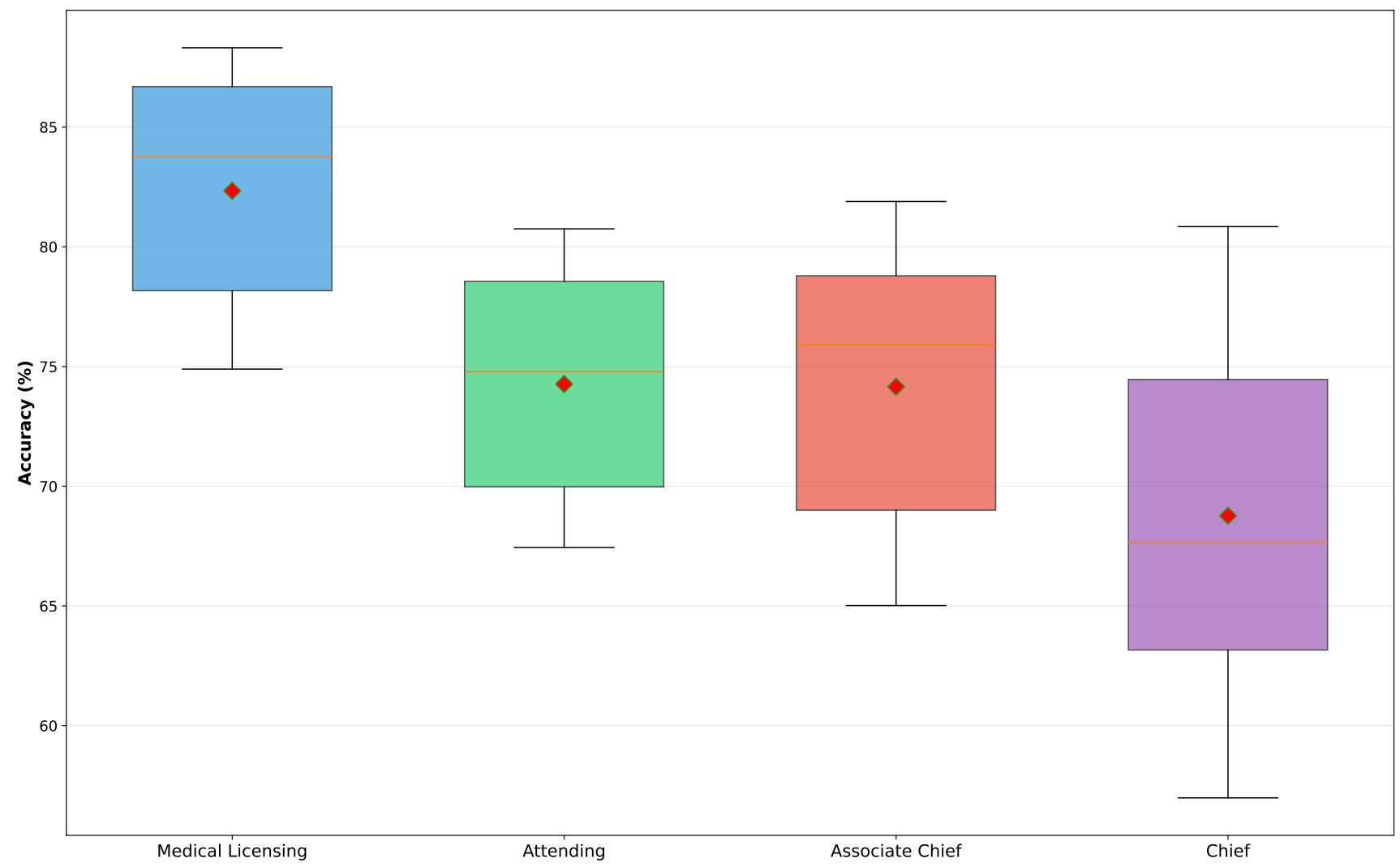

Figure S17: Categorical box plot comparing the distribution of model accuracies across five examination categories: Medical Licensing Examination (MLE), Attending Physician (AP), Associate Chief Physician (ACP), Chief Physician (CP), and International benchmarks (USMLE, AJKD). Each box summarizes the accuracy distribution across all evaluated models for that category, enabling assessment of relative difficulty levels and identification of categories where model performance shows greatest variance, indicating opportunities for targeted improvement in specific professional domains.

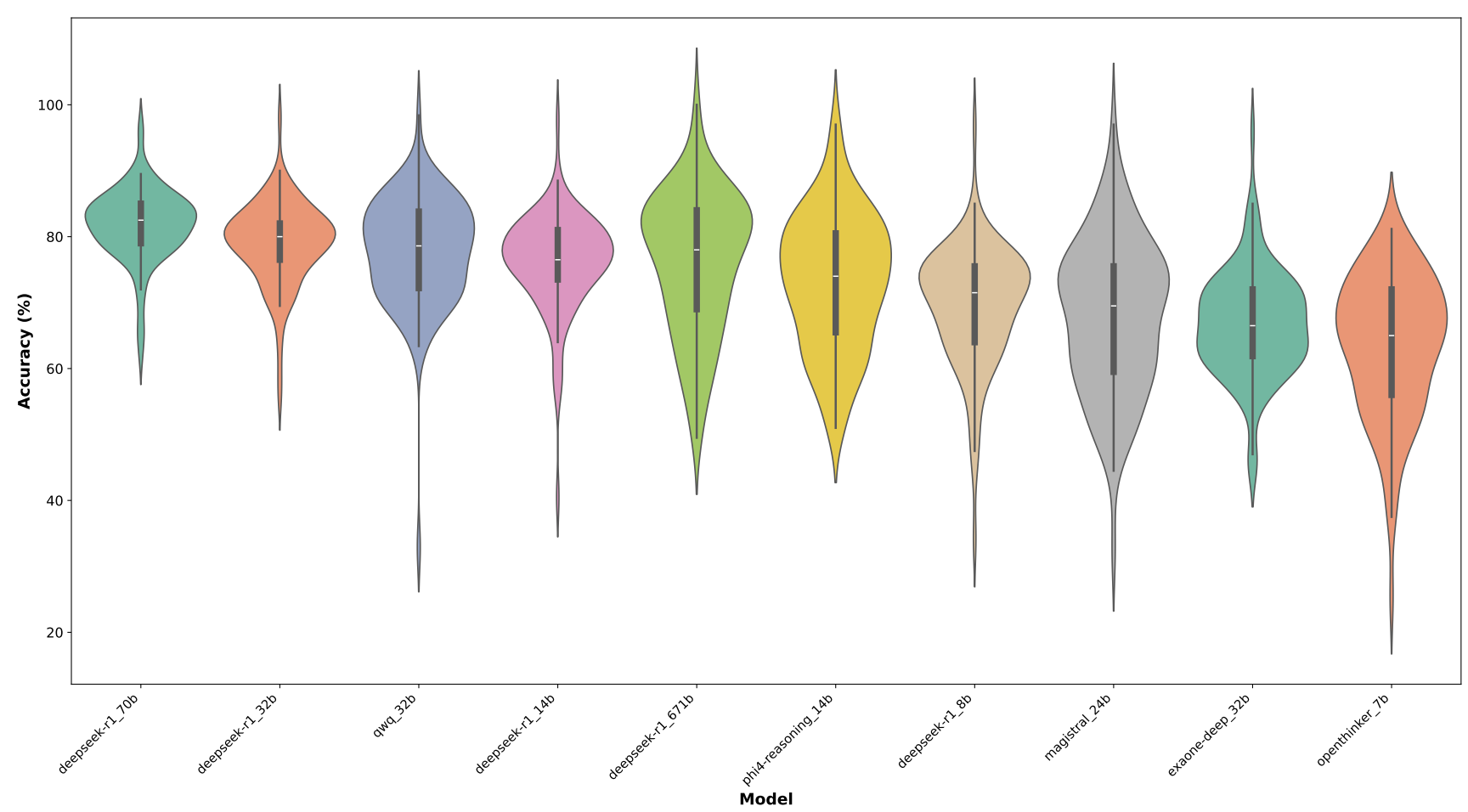

Figure S18: Violin plot visualization displaying the complete accuracy distribution shape for each of the top 10 performing models across all 93 datasets. Unlike box plots that summarize distributions with quartiles, violin plots reveal the full probability density, exposing bimodality (suggesting distinct performance regimes across dataset types), skewness (indicating systematic difficulty patterns), and the presence of outlier datasets where individual models underperform relative to their overall standing. The width at each accuracy level represents the proportion of datasets achieving that performance.

Trend Analysis

Figure S19 tracks model performance across Medical Licensing Examination years (2017–2024). Figure S20 plots parameter count against accuracy across all 172 models. Figure S21 ranks all 93 datasets by difficulty based on average model accuracy.

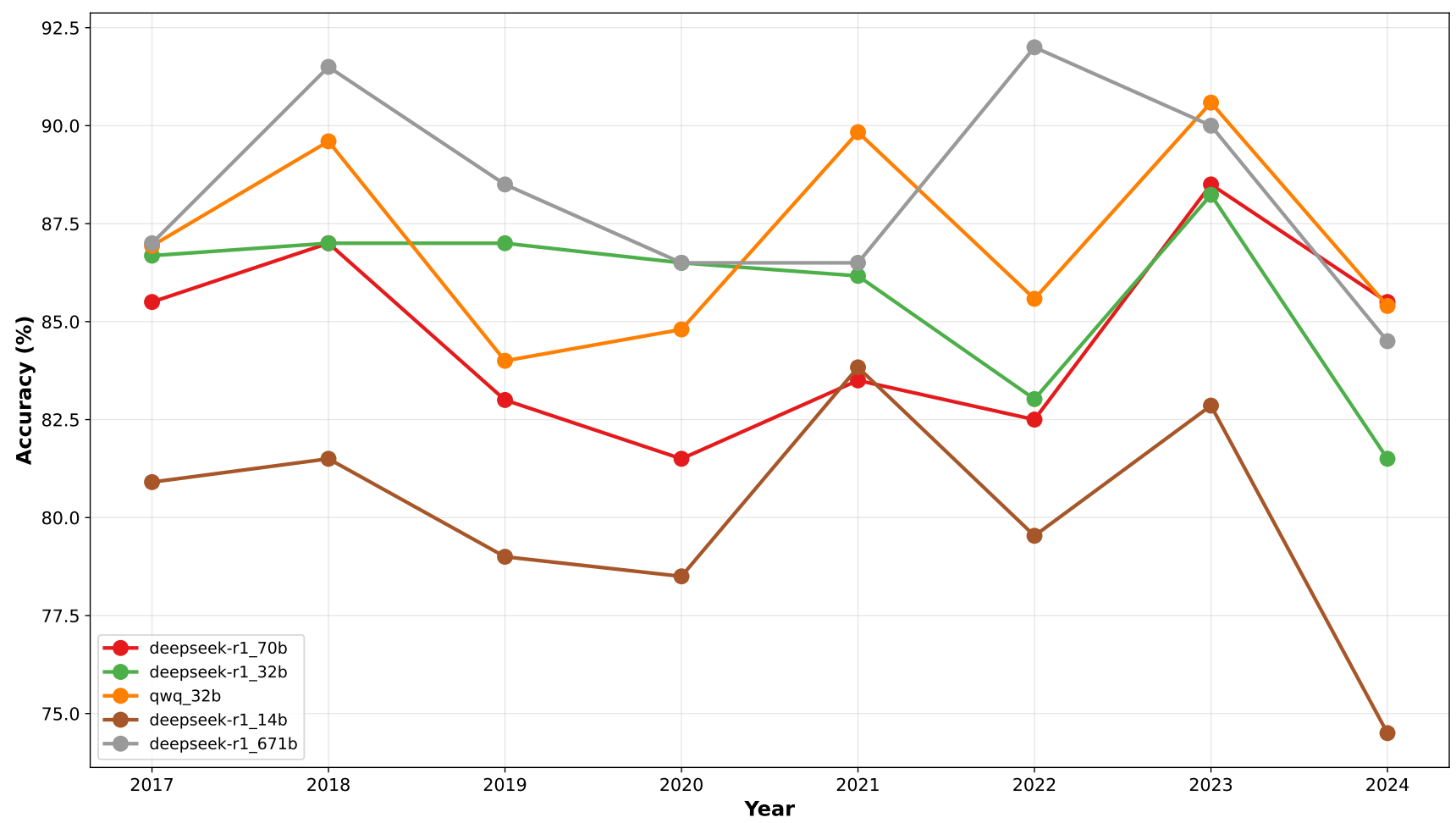

Figure S19: Temporal trend analysis line chart tracking the performance of top 5 models across Chinese Medical Licensing Examination (MLE) editions from 2017 to 2024. Connected data points reveal whether model performance remains stable across examination years or exhibits systematic trends potentially reflecting evolving examination difficulty, changing content emphases, or differential model sensitivity to temporal knowledge distribution shifts in training data. Parallel trajectories across models suggest examination-driven effects, while divergent patterns indicate model-specific temporal sensitivities.

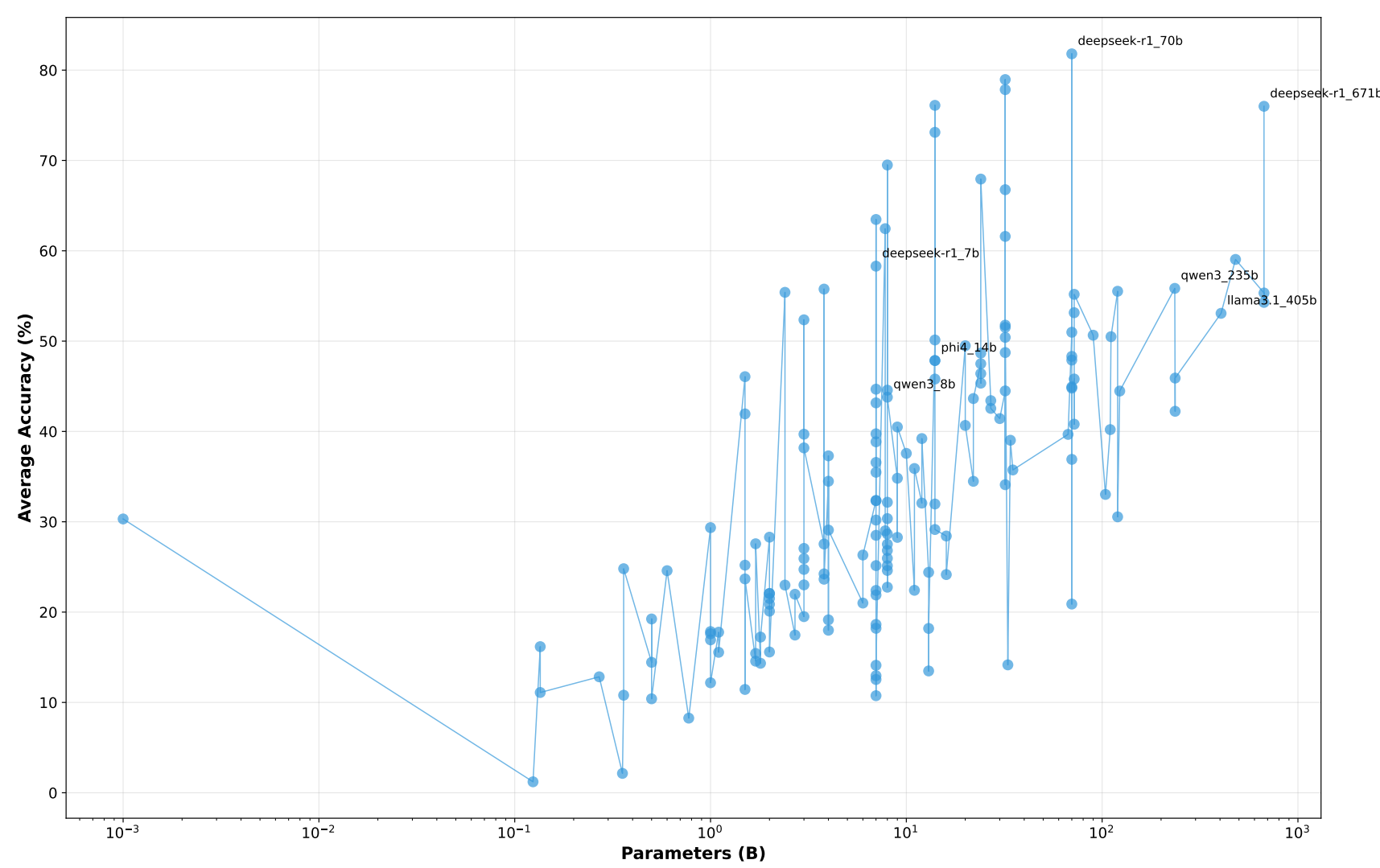

Figure S20: Scatter plot with logarithmic x-axis displaying the relationship between model parameter count and average accuracy across all 93 datasets, with key high-performing models individually annotated. The visualization enables identification of Pareto-optimal models that maximize accuracy for their parameter budget, as well as detection of the parameter threshold beyond which additional scale yields diminishing returns. Annotations highlight reasoning-enhanced models that achieve accuracy levels substantially above the general-purpose trendline at equivalent parameter counts.

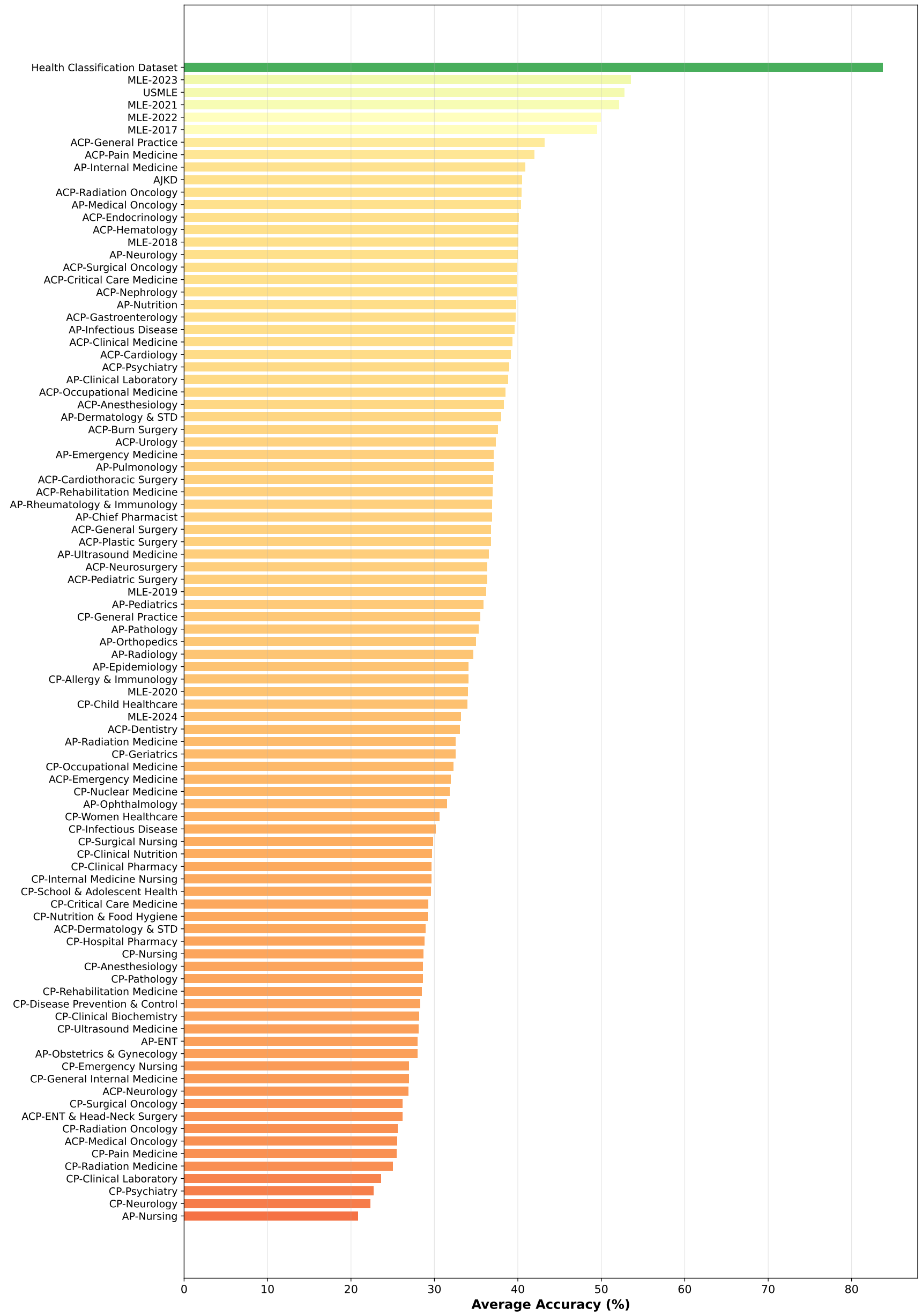

Figure S21: Horizontal bar chart ranking all 93 benchmark datasets by difficulty, operationalized as average accuracy across all evaluated models (lower average accuracy indicates higher difficulty). This visualization identifies the most challenging datasets in the benchmark, which may represent subspecialties requiring rare expertise, questions with high reasoning complexity, or domains where current language models exhibit systematic knowledge gaps. The ranking provides guidance for targeted model improvement and helps calibrate expectations for clinical deployment across different medical specialties.

Correlation Analysis

Figure S22 examines USMLE vs. MLE cross-benchmark correlation. Figure S23 shows the correlation between Attending Physician and Associate Chief Physician performance. Figure S24 visualizes model families across parameter count, accuracy, and variant breadth simultaneously.

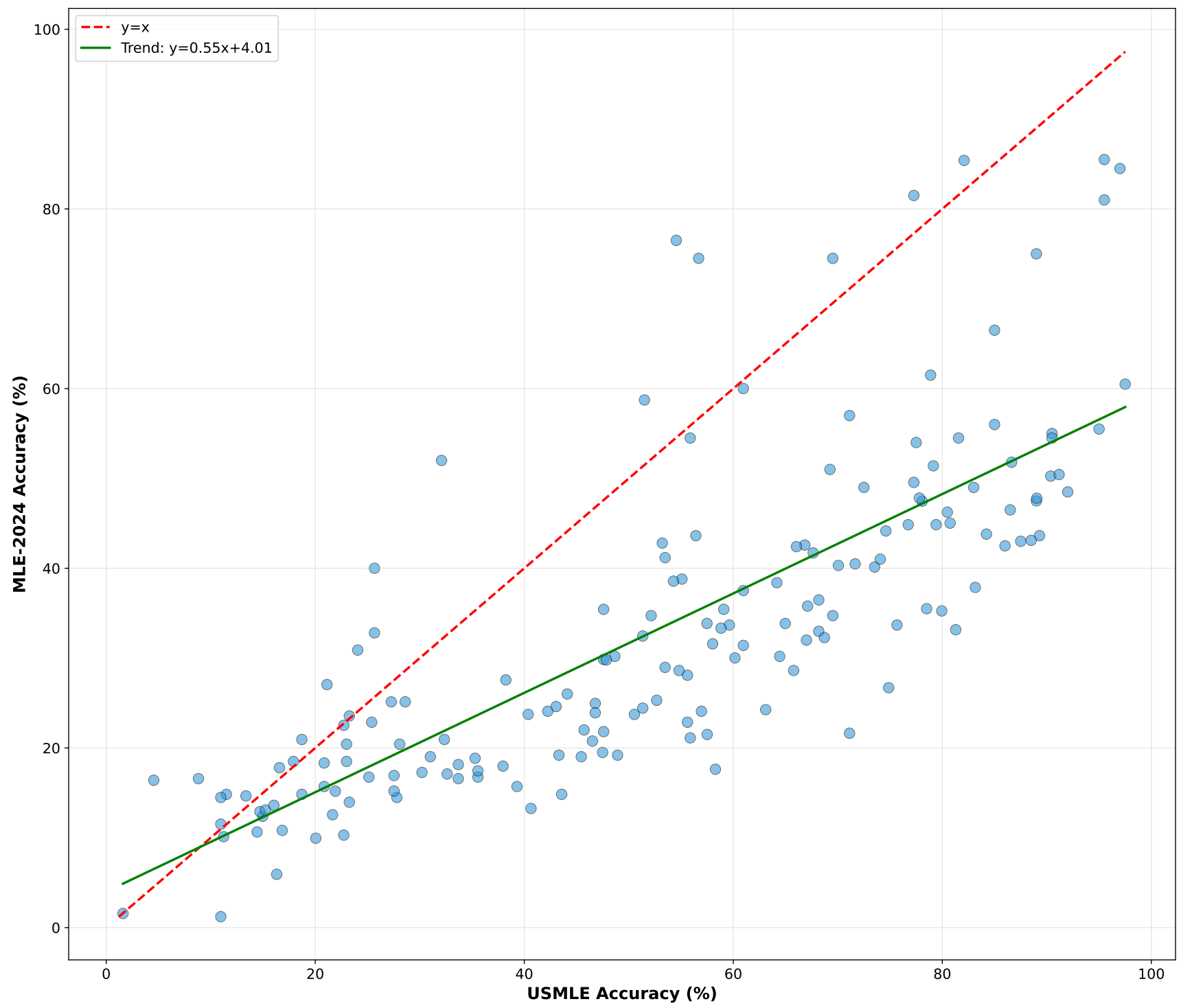

Figure S22: Cross-benchmark scatter plot examining the correlation between model performance on the United States Medical Licensing Examination (USMLE, x-axis) and Chinese Medical Licensing Examination 2024 edition (MLE-2024, y-axis). Strong positive correlation would suggest that medical reasoning capabilities transfer across linguistic and cultural contexts, while deviations from the diagonal identify models with language-specific advantages or disadvantages. This cross-cultural comparison has direct implications for the global deployment potential of medical AI systems.

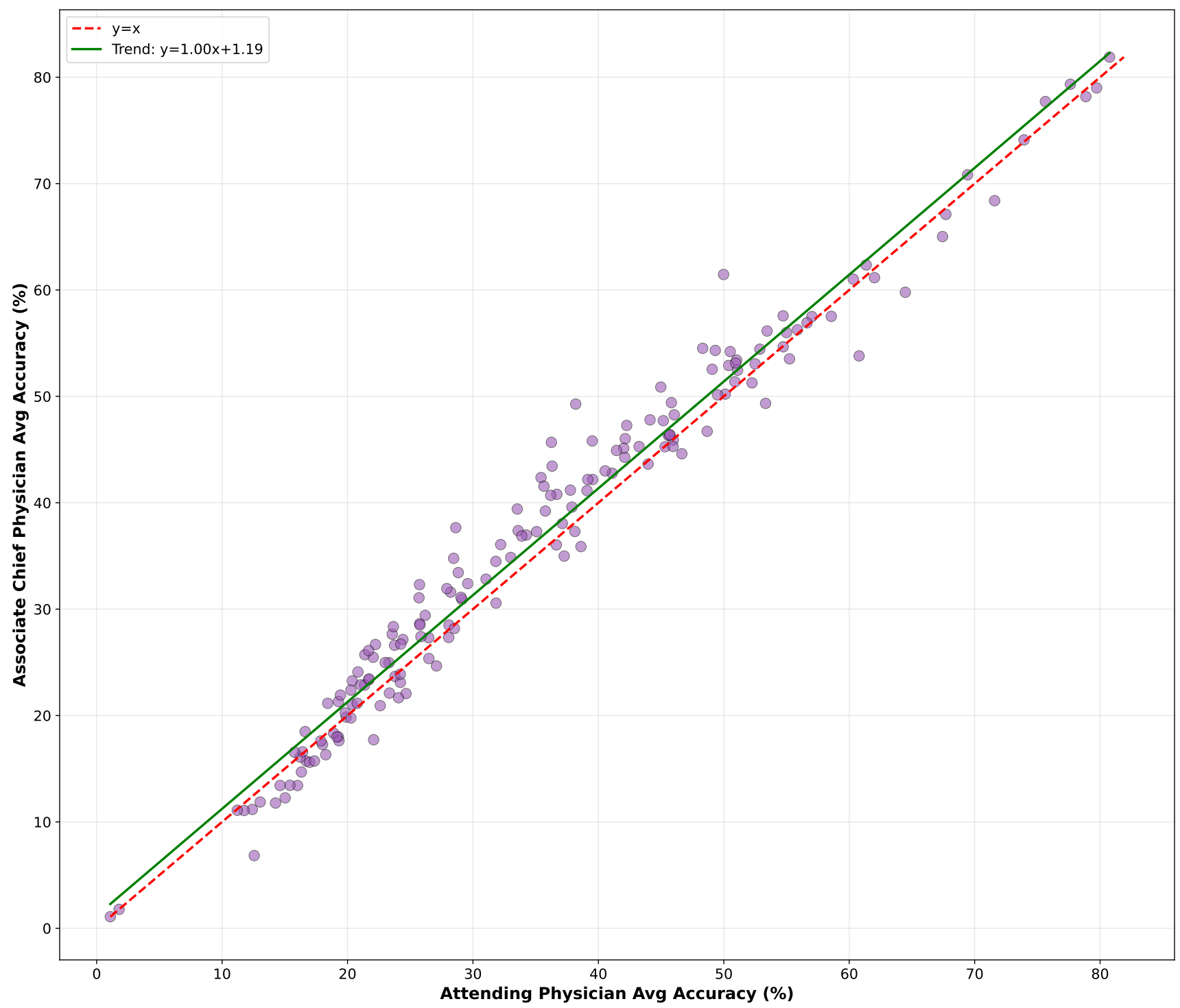

Figure S23: Professional level correlation scatter plot examining the relationship between model performance on Attending Physician (AP, intermediate level) and Associate Chief Physician (ACP, senior level) examinations. High correlation indicates that models maintain relative rankings across professional difficulty tiers, while low correlation would suggest that advanced clinical reasoning requirements at the ACP level differentially challenge certain architectures. The scatter pattern reveals whether success at intermediate professional levels reliably predicts performance on more demanding senior-level assessments.

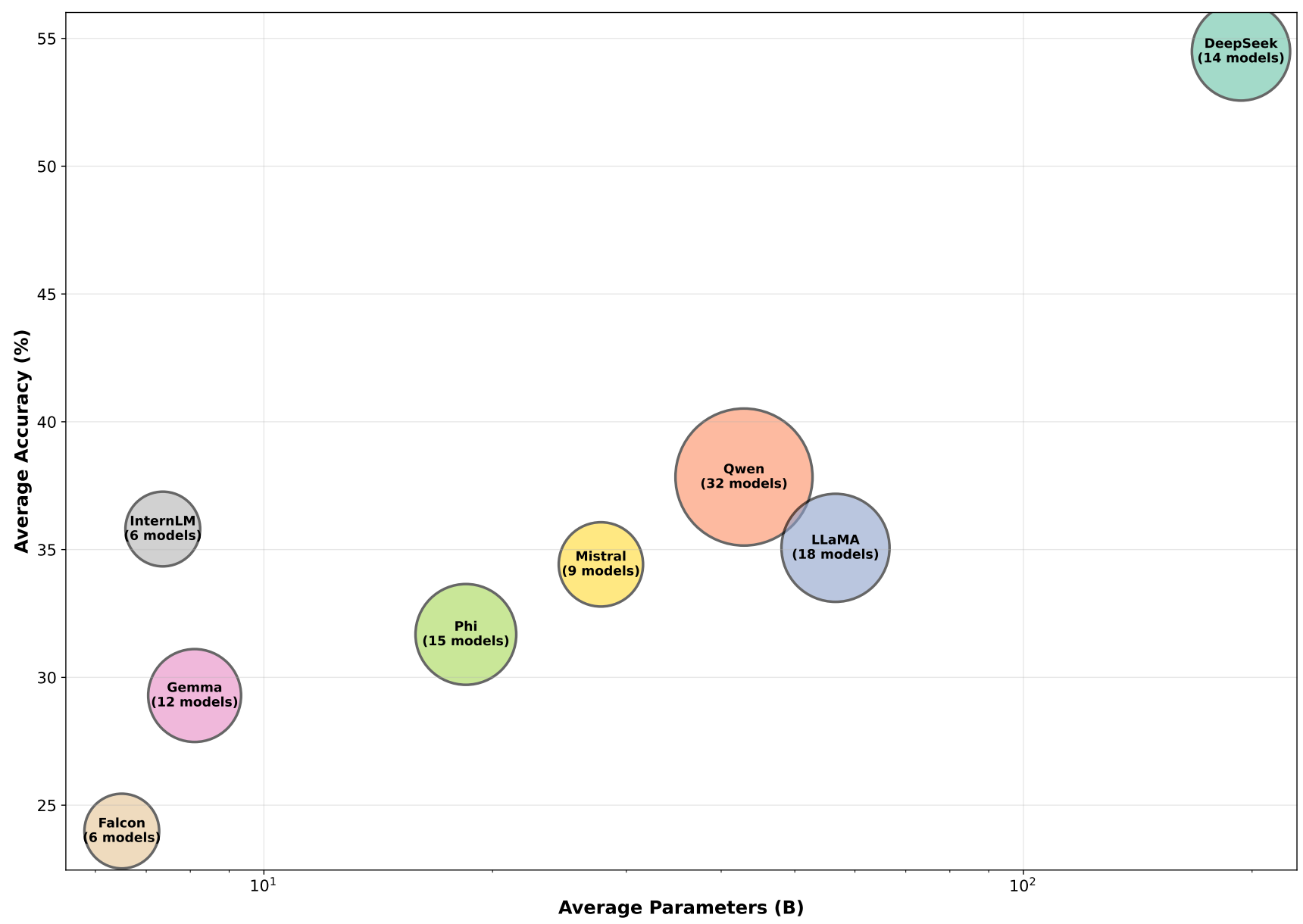

Figure S24: Multi-dimensional bubble chart visualizing model families across three dimensions: average parameter count (x-axis, logarithmic scale), average accuracy across all datasets (y-axis), and number of model variants evaluated within each family (bubble size). This representation enables simultaneous assessment of each family's parameter efficiency (accuracy relative to size), breadth of scale coverage (bubble size indicating variant diversity), and absolute performance level. Families positioned in the upper-left region achieve high accuracy with efficient parameter utilization, representing optimal targets for resource-constrained deployment scenarios.

Per-Dataset Performance Results

Figures S25–S117 show detailed model performance on each of the 93 individual benchmark datasets, with models ranked by descending accuracy and bar colors distinguishing reasoning-enhanced architectures from general-purpose models.

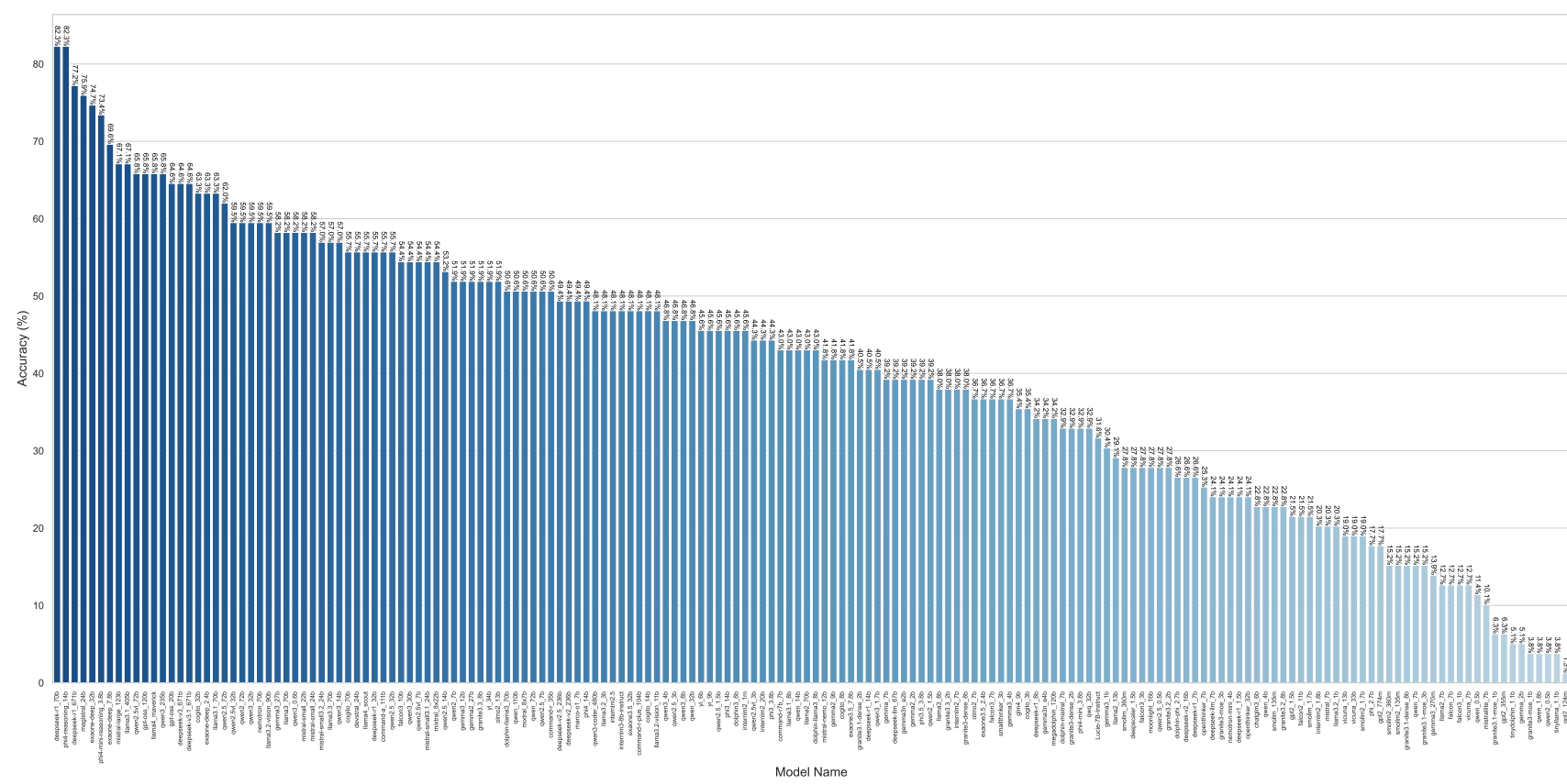

Figure S25: Ranked horizontal bar chart displaying accuracy of all 172 evaluated models on the American Journal of Kidney Diseases (AJKD) nephrology dataset, a specialized assessment requiring deep subspecialty expertise in kidney diseases, dialysis management, and renal pathophysiology. Models are sorted by descending accuracy, with bar colors distinguishing reasoning-enhanced architectures (achieving top performance around 82%) from general-purpose models. The substantial performance gap between top and bottom performers (approximately 60 percentage points) highlights the discriminative value of this challenging specialty benchmark. Top performers: DeepSeek-R1-70b (82.3%), Phi4-Reasoning-14b (82.3%), DeepSeek-R1-671b (77.2%).

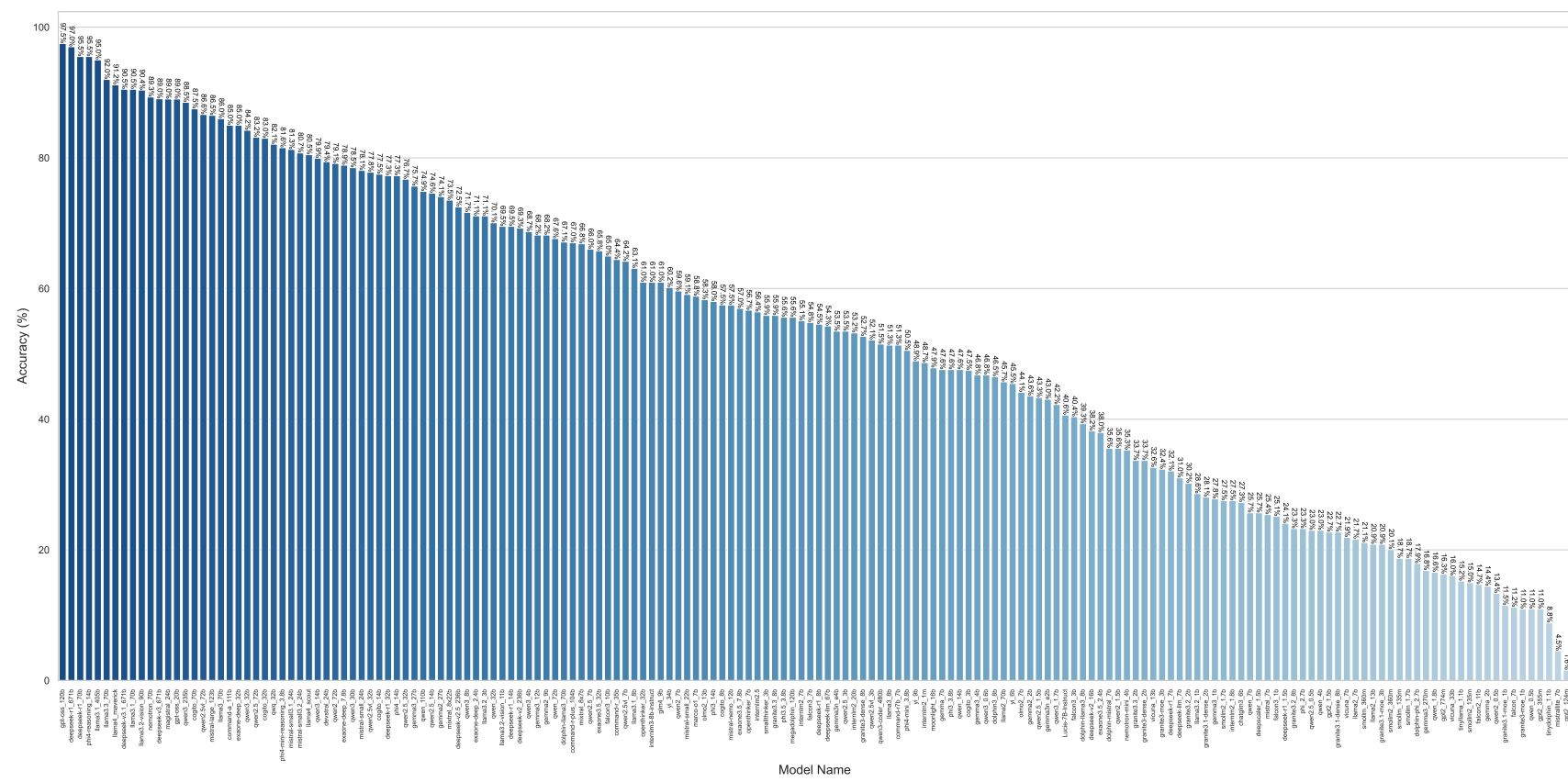

Figure S26: Comprehensive bar chart presenting model accuracy rankings on the United States Medical Licensing Examination (USMLE), the standardized assessment required for physician licensure in the United States. Top reasoning-enhanced models achieve remarkable accuracies approaching 97%, substantially exceeding the human passing threshold of approximately 60%. This near-ceiling performance by leading open-source models demonstrates that state-of-the-art AI systems can match or exceed proprietary alternatives on this gold-standard medical benchmark, with important implications for accessible healthcare AI deployment. Top performers: GPT-OSS-120b (97.5%), DeepSeek-R1-671b (97.0%), DeepSeek-R1-70b (95.5%).

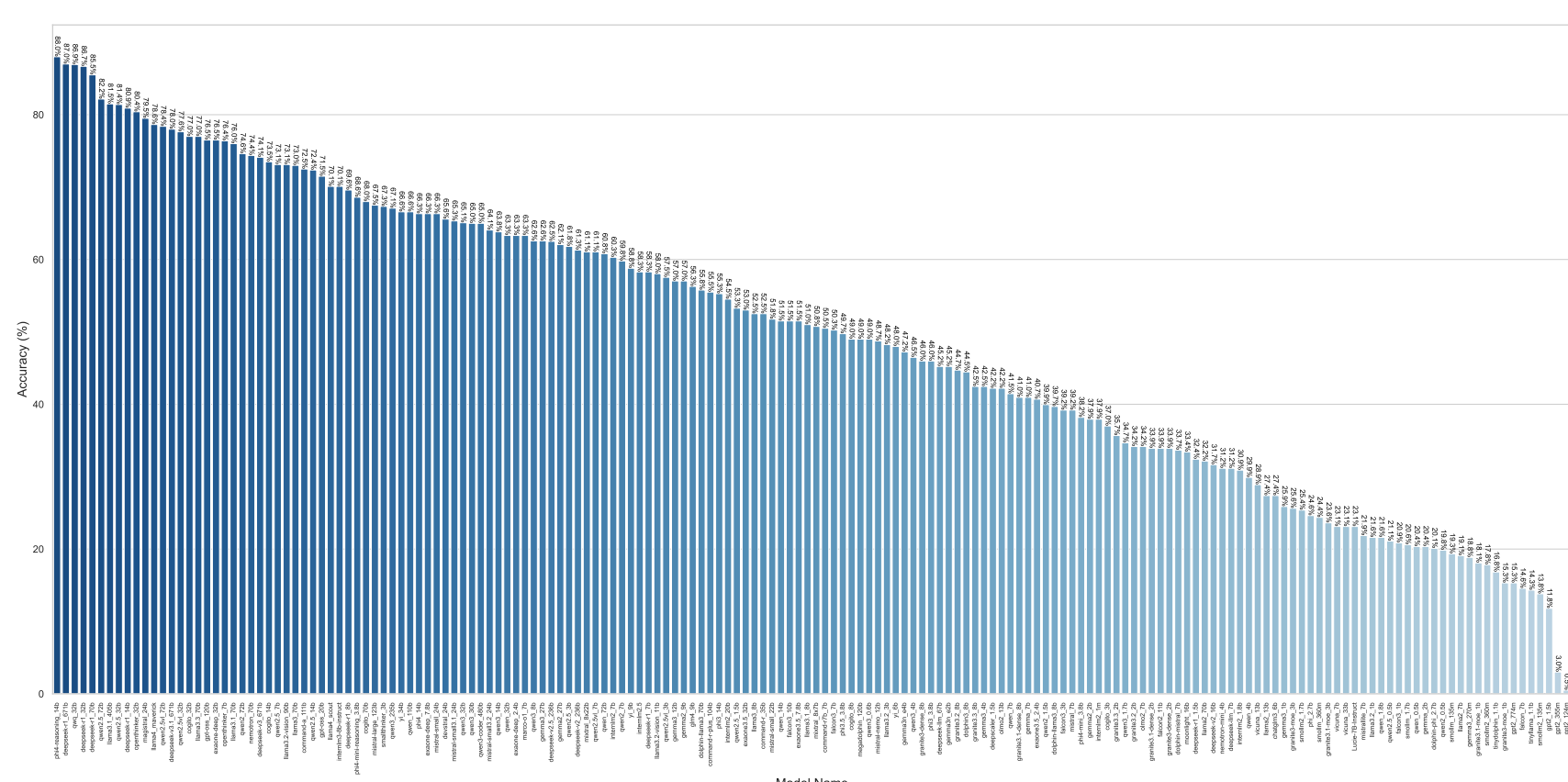

Figure S27: Bar chart of model accuracy rankings on the 2017 Chinese Medical Licensing Examination (MLE-2017), representing entry-level physician certification requirements. This examination tests fundamental clinical knowledge across major medical disciplines, establishing baseline competency for medical practice. Performance comparison across annual MLE editions enables assessment of temporal stability in model capabilities and identification of examination-year-specific difficulty variations. Top performers: Phi4-Reasoning-14b (88.0%), DeepSeek-R1-671b (87.0%), QWQ-32b (86.9%).

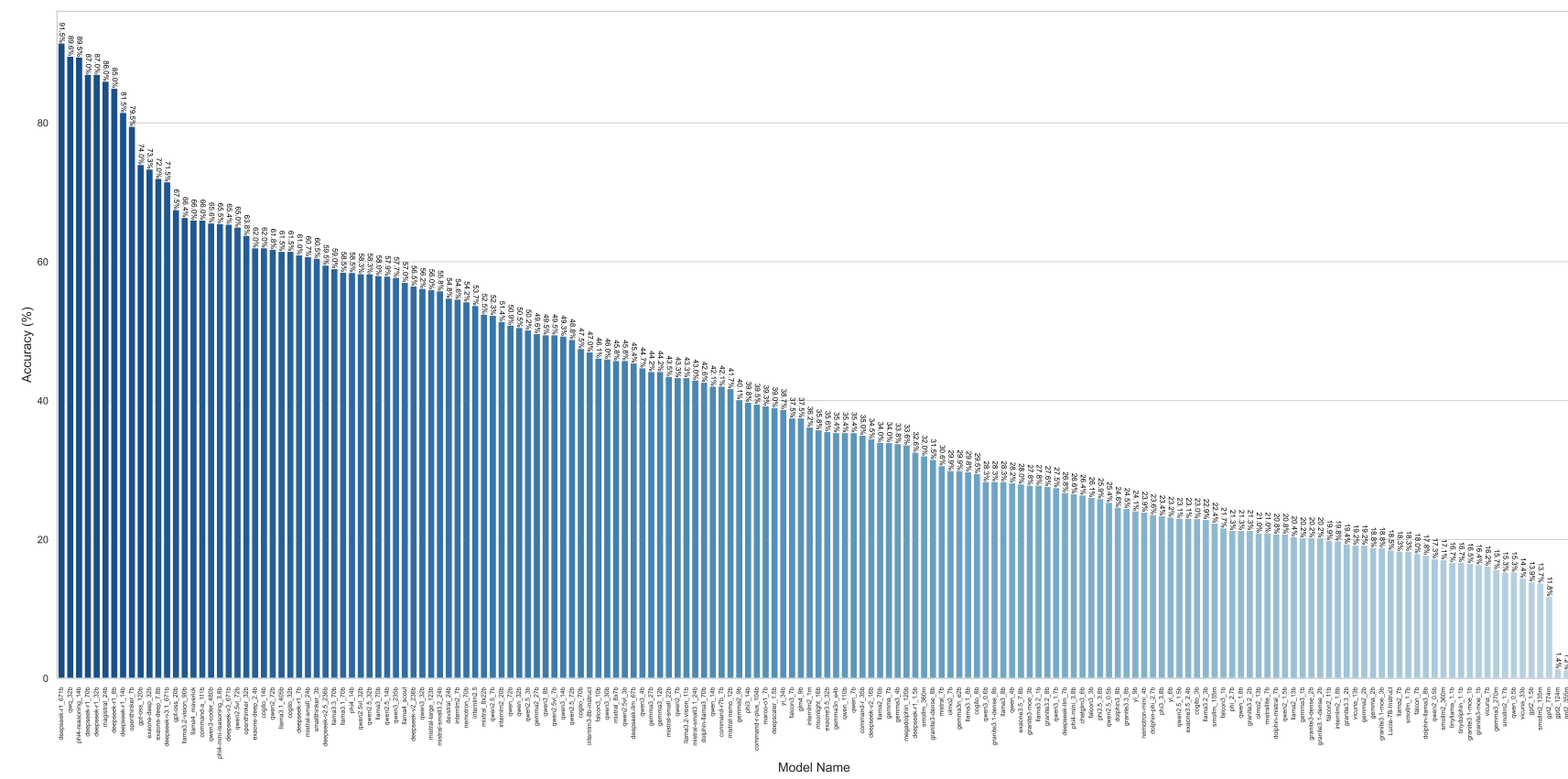

Figure S28: Bar chart of model accuracy rankings on the 2018 Chinese Medical Licensing Examination (MLE-2018). Comparison with adjacent examination years reveals consistency in model relative rankings while highlighting any year-specific content emphases or difficulty calibrations that may differentially affect certain model architectures. Top performers: DeepSeek-R1-671b (91.5%), QWQ-32b (89.6%), Phi4-Reasoning-14b (89.5%).

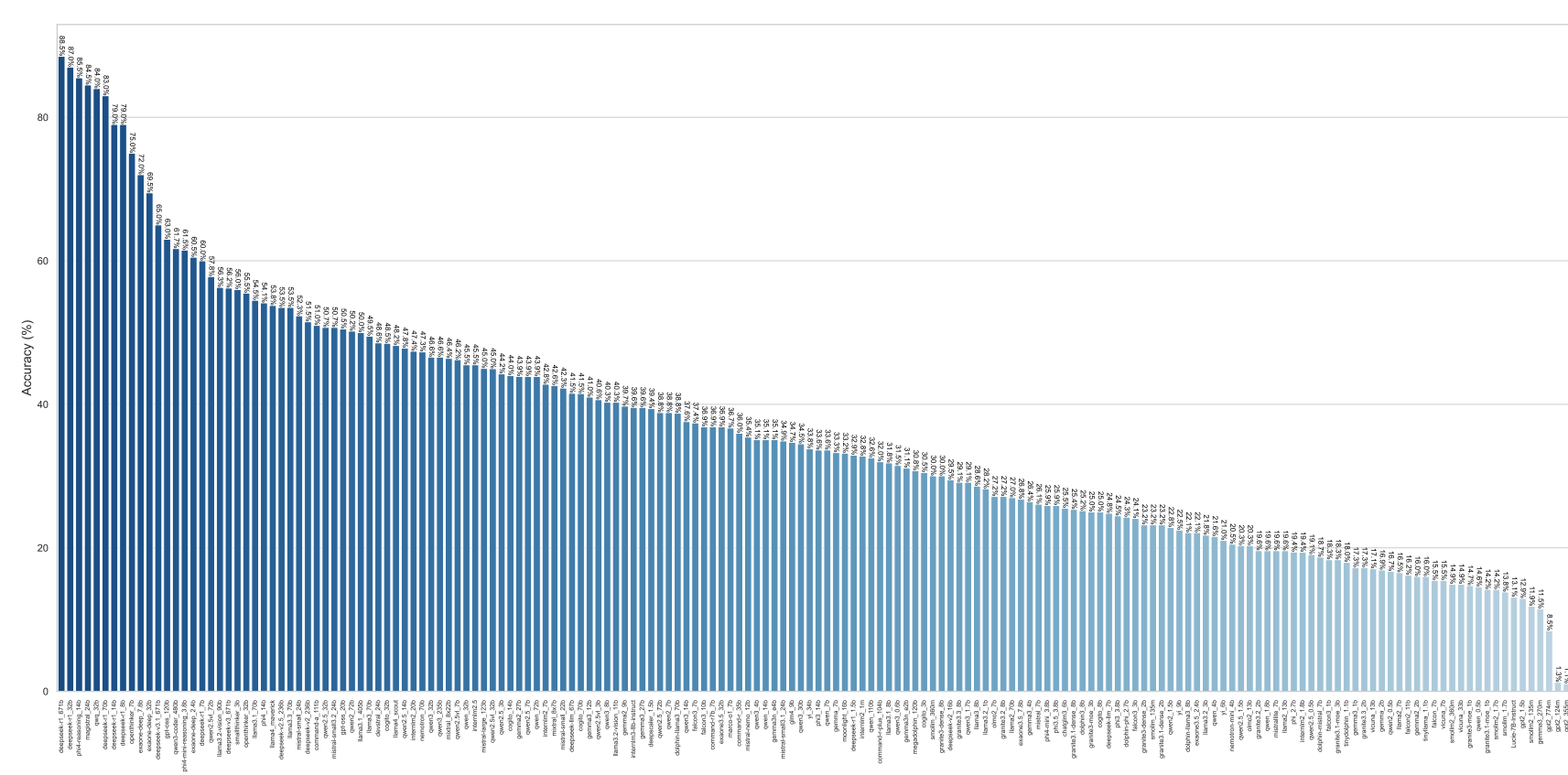

Figure S29: Bar chart of model accuracy rankings on the 2019 Chinese Medical Licensing Examination (MLE-2019). The temporal sequence of MLE examinations provides longitudinal insight into how examination content evolution interacts with model knowledge distributions derived from training data with varying temporal coverage. Top performers: DeepSeek-R1-671b (88.5%), DeepSeek-R1-32b (87.0%), Phi4-Reasoning-14b (85.5%).

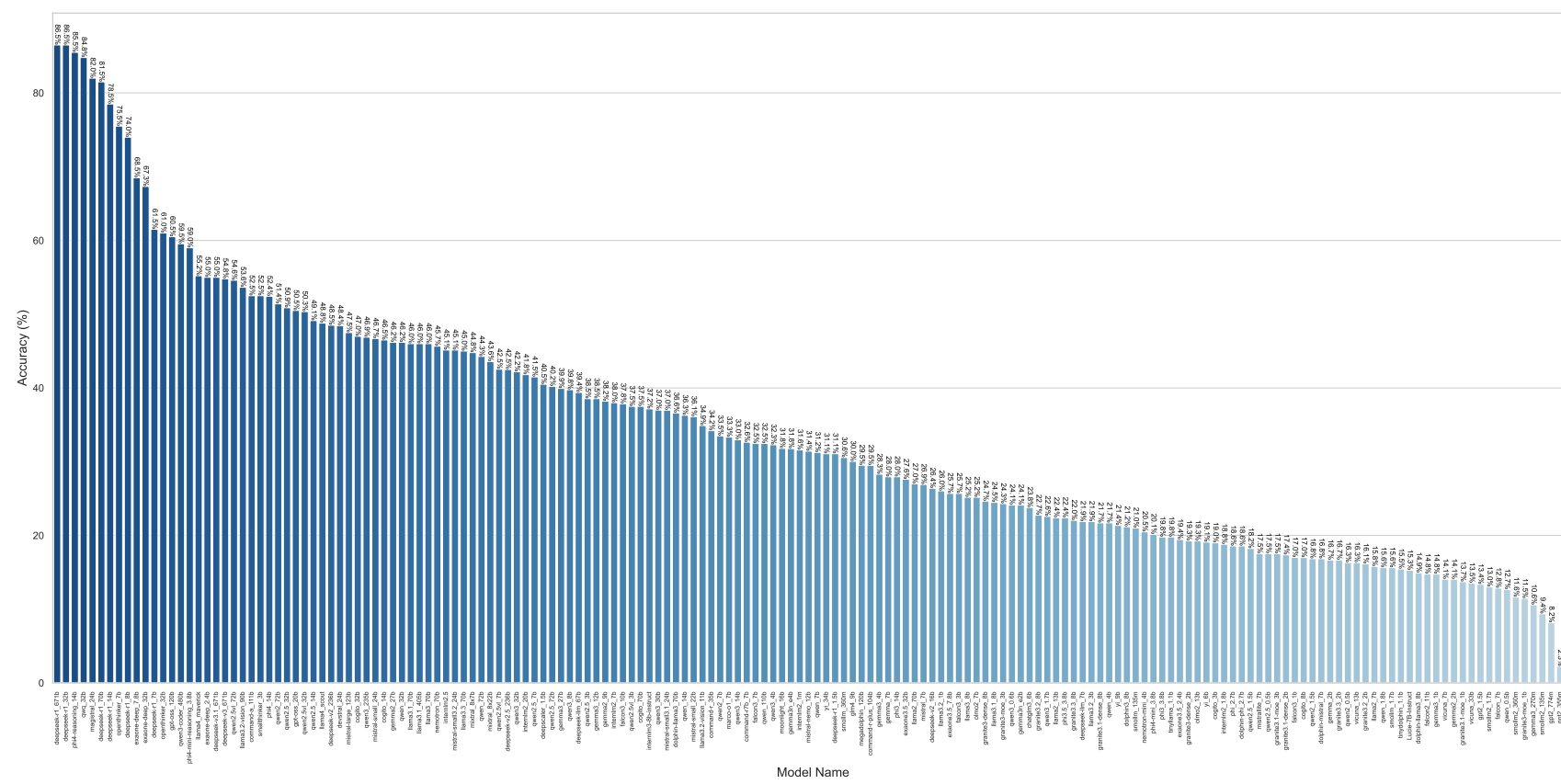

Figure S30: Bar chart of model accuracy rankings on the 2020 Chinese Medical Licensing Examination (MLE-2020). Examinations from this period may reflect emerging emphasis on pandemic-related clinical knowledge and public health competencies, providing insight into model performance on contemporary medical challenges. Top performers: DeepSeek-R1-671b (86.5%), DeepSeek-R1-32b (86.5%), Phi4-Reasoning-14b (85.5%).

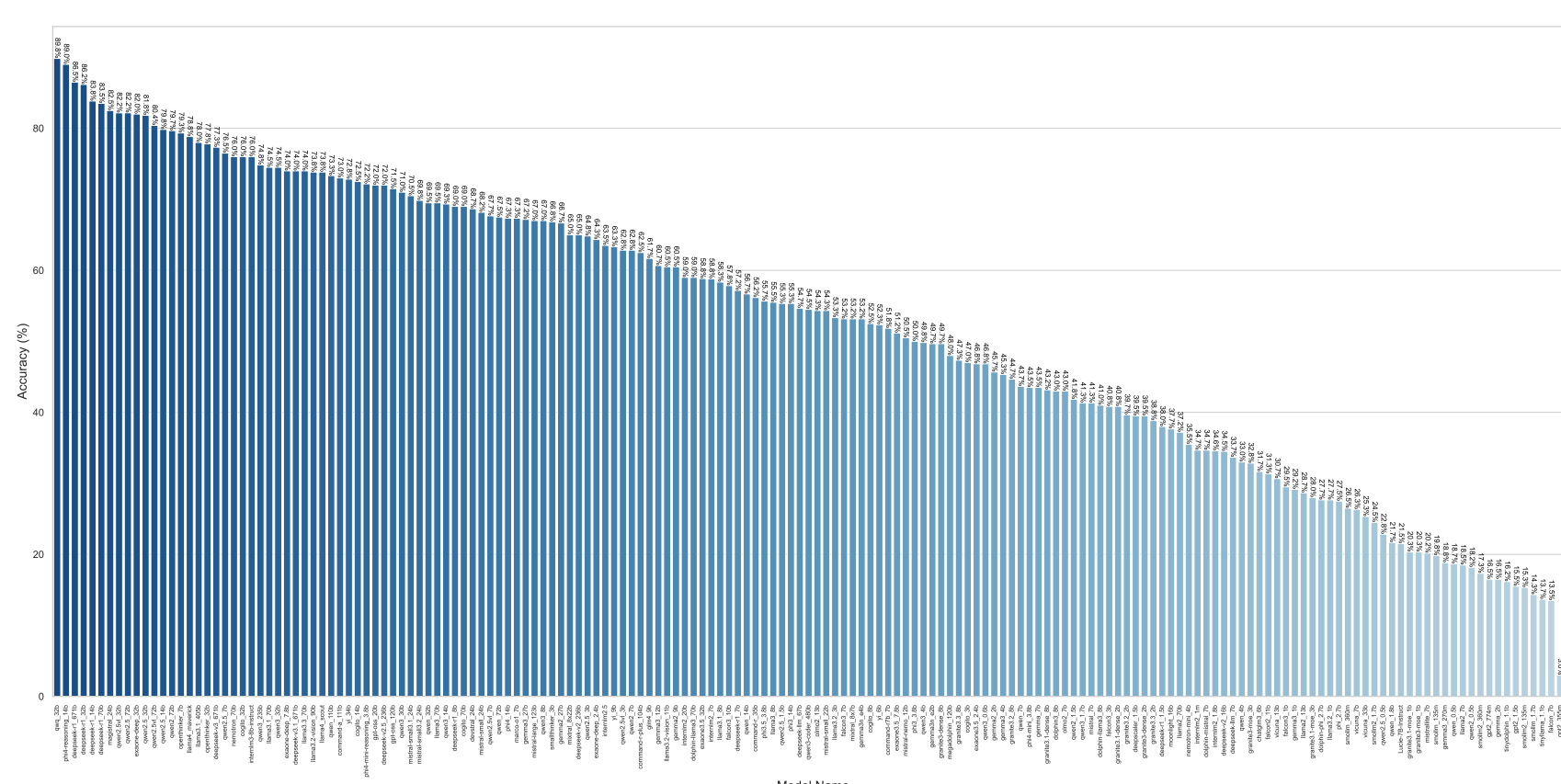

Figure S31: Bar chart of model accuracy rankings on the 2021 Chinese Medical Licensing Examination (MLE-2021). Performance patterns on recent examination years are particularly relevant for assessing model applicability to current clinical practice standards and medical knowledge expectations. Top performers: QWQ-32b (89.8%), Phi4-Reasoning-14b (89.0%), DeepSeek-R1-671b (86.5%).

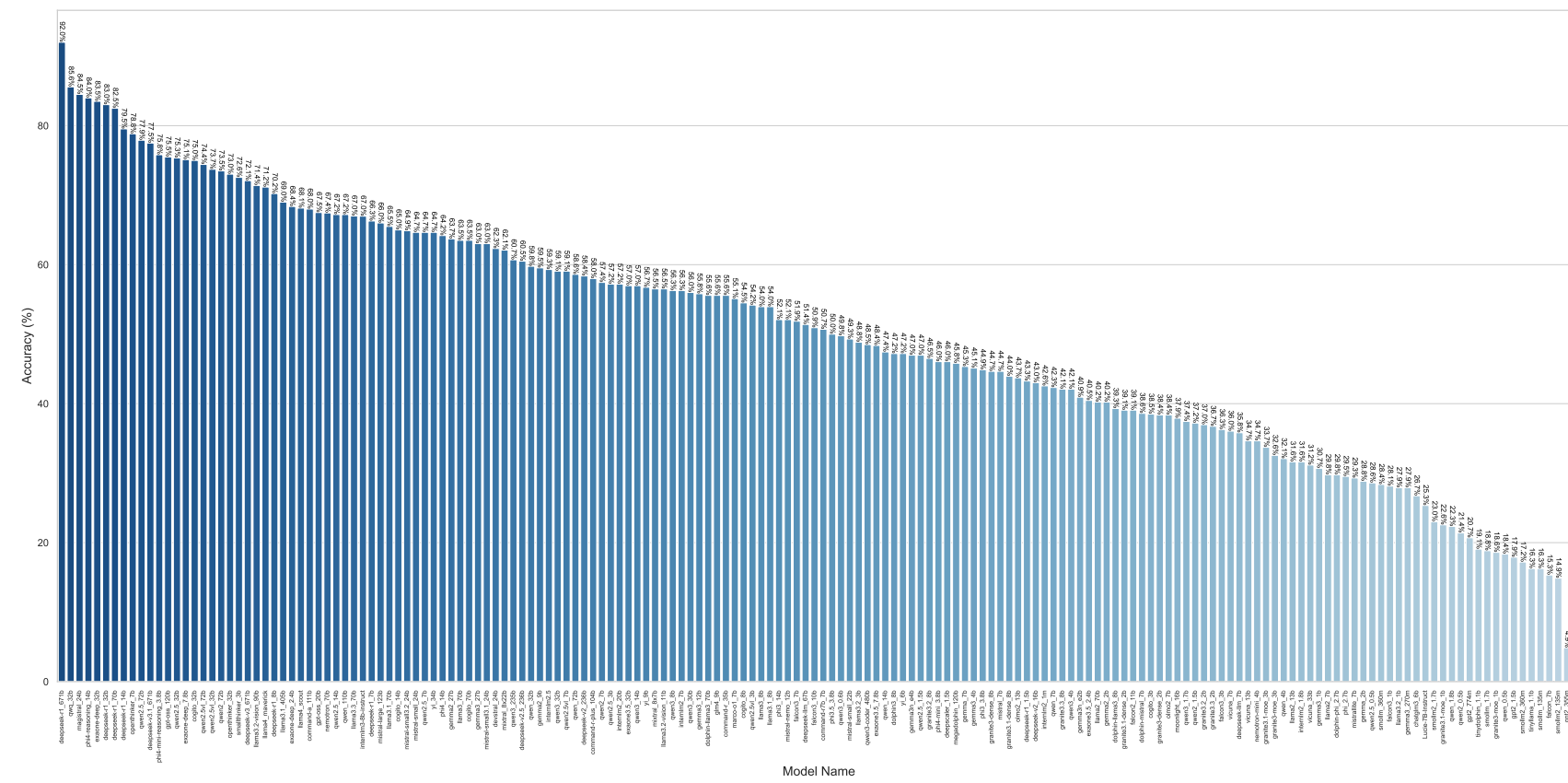

Figure S32: Bar chart of model accuracy rankings on the 2022 Chinese Medical Licensing Examination (MLE-2022). Recent examination editions provide the most relevant assessment of model suitability for contemporary clinical decision support applications. Top performers: DeepSeek-R1-671b (92.0%), QWQ-32b (85.6%), Magistral-24b (84.5%).

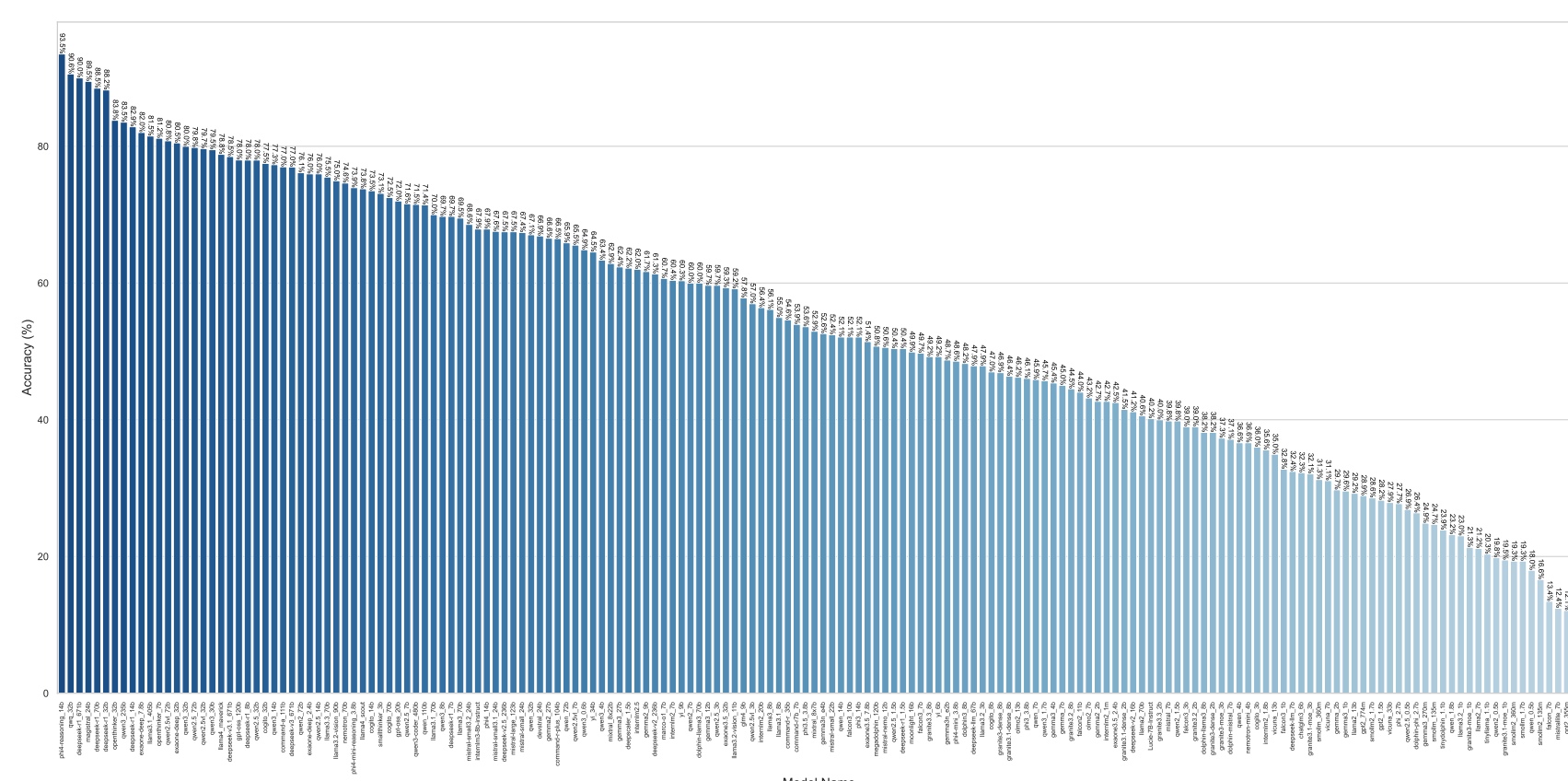

Figure S33: Bar chart of model accuracy rankings on the 2023 Chinese Medical Licensing Examination (MLE-2023). Performance on this recent examination reflects model competency on current medical knowledge standards, with top models achieving accuracies significantly exceeding human passing thresholds. Top performers: Phi4-Reasoning-14b (93.5%), QWQ-32b (90.6%), DeepSeek-R1-671b (90.0%).

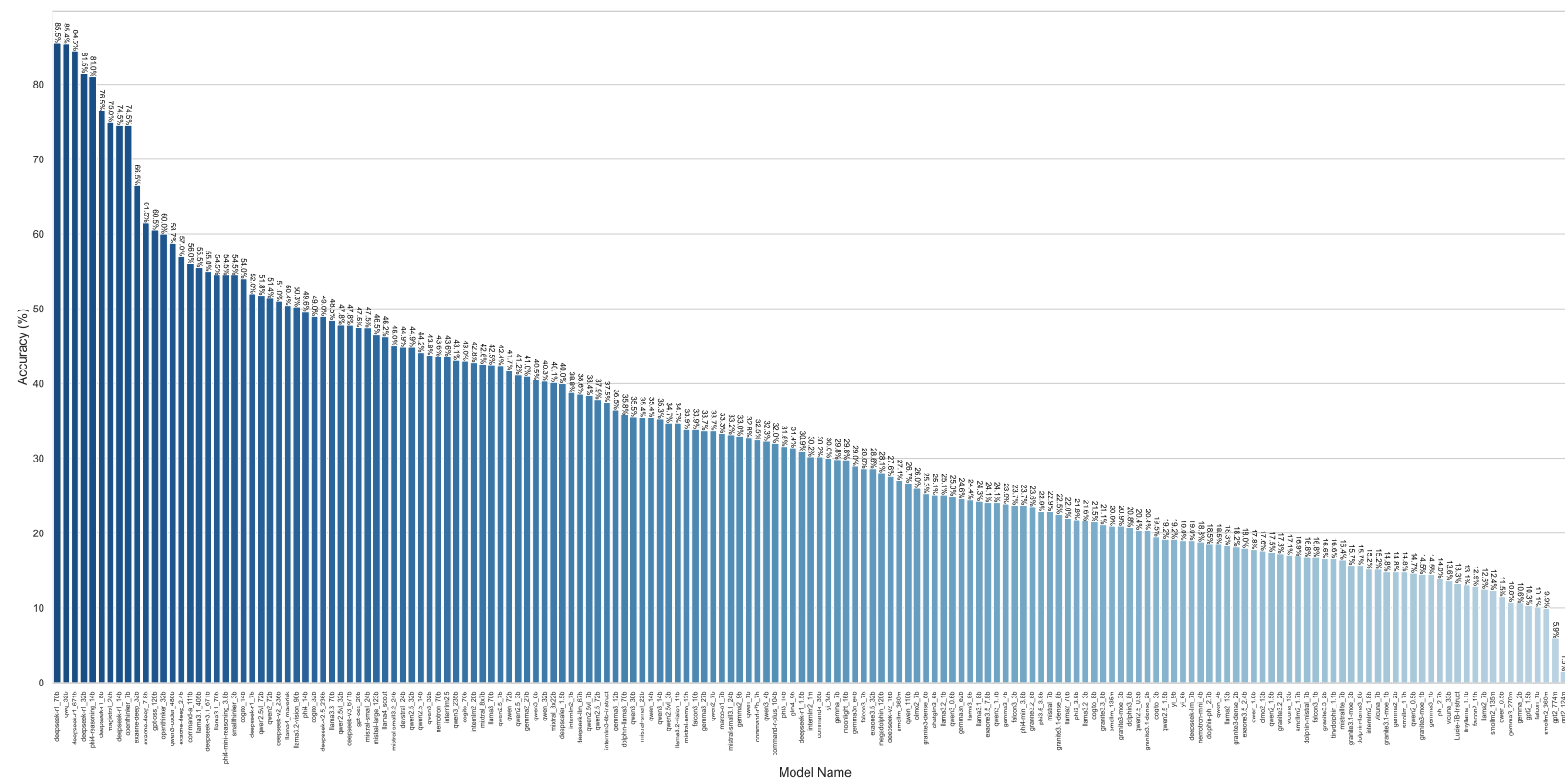

Figure S34: Bar chart of model accuracy rankings on the 2024 Chinese Medical Licensing Examination (MLE-2024), the most recent edition in our benchmark representing contemporary physician competency standards. Performance on this examination provides the most current assessment of model readiness for clinical deployment in Chinese healthcare settings. Top performers: DeepSeek-R1-70b (85.5%), QWQ-32b (85.4%), DeepSeek-R1-671b (84.5%).

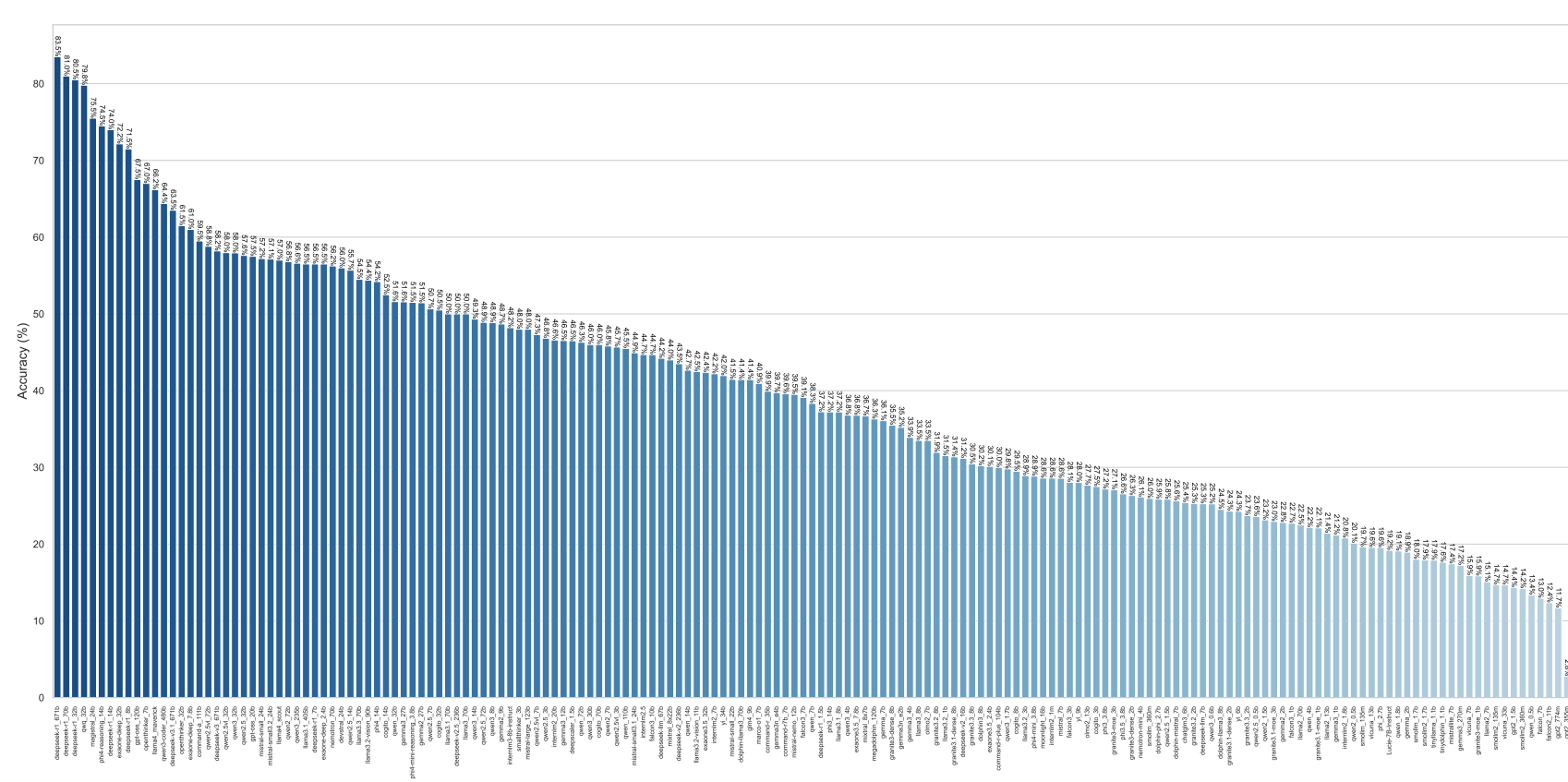

Figure S35: Bar chart of model accuracy rankings on the Attending Physician Clinical Laboratory examination, assessing intermediate-level expertise in laboratory medicine including test interpretation, quality control, and diagnostic applications of clinical biochemistry, hematology, microbiology, and immunology assays. Top performers: DeepSeek-R1-671b (83.5%), DeepSeek-R1-70b (81.0%), DeepSeek-R1-32b (80.5%).

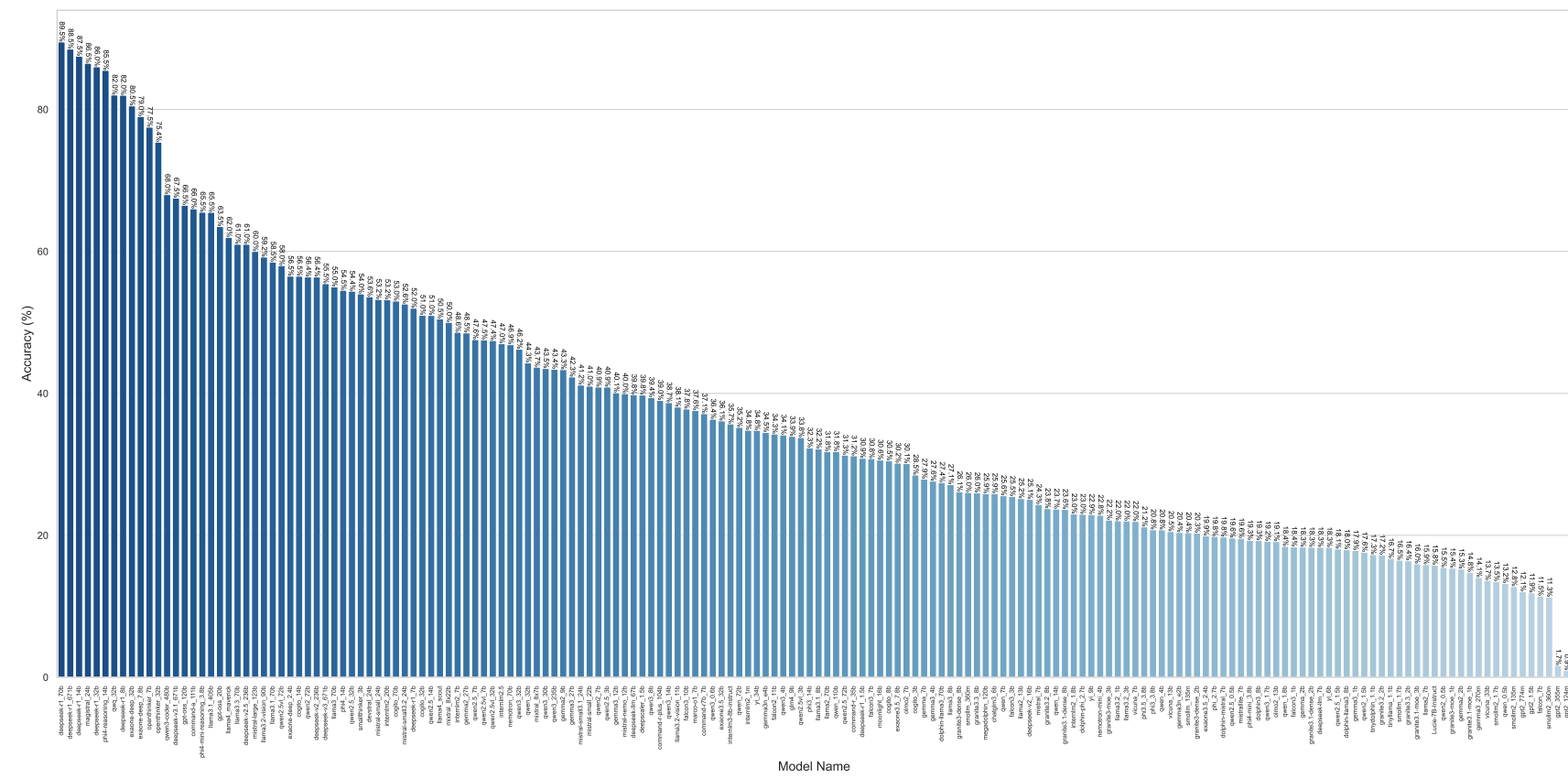

Figure S36: Bar chart of model accuracy rankings on the Attending Physician Chief Pharmacist examination, evaluating knowledge of clinical pharmacology, drug interactions, therapeutic drug monitoring, and pharmaceutical care principles essential for medication management. Top performers: DeepSeek-R1-70b (89.5%), DeepSeek-R1-671b (88.5%), DeepSeek-R1-14b (87.5%).

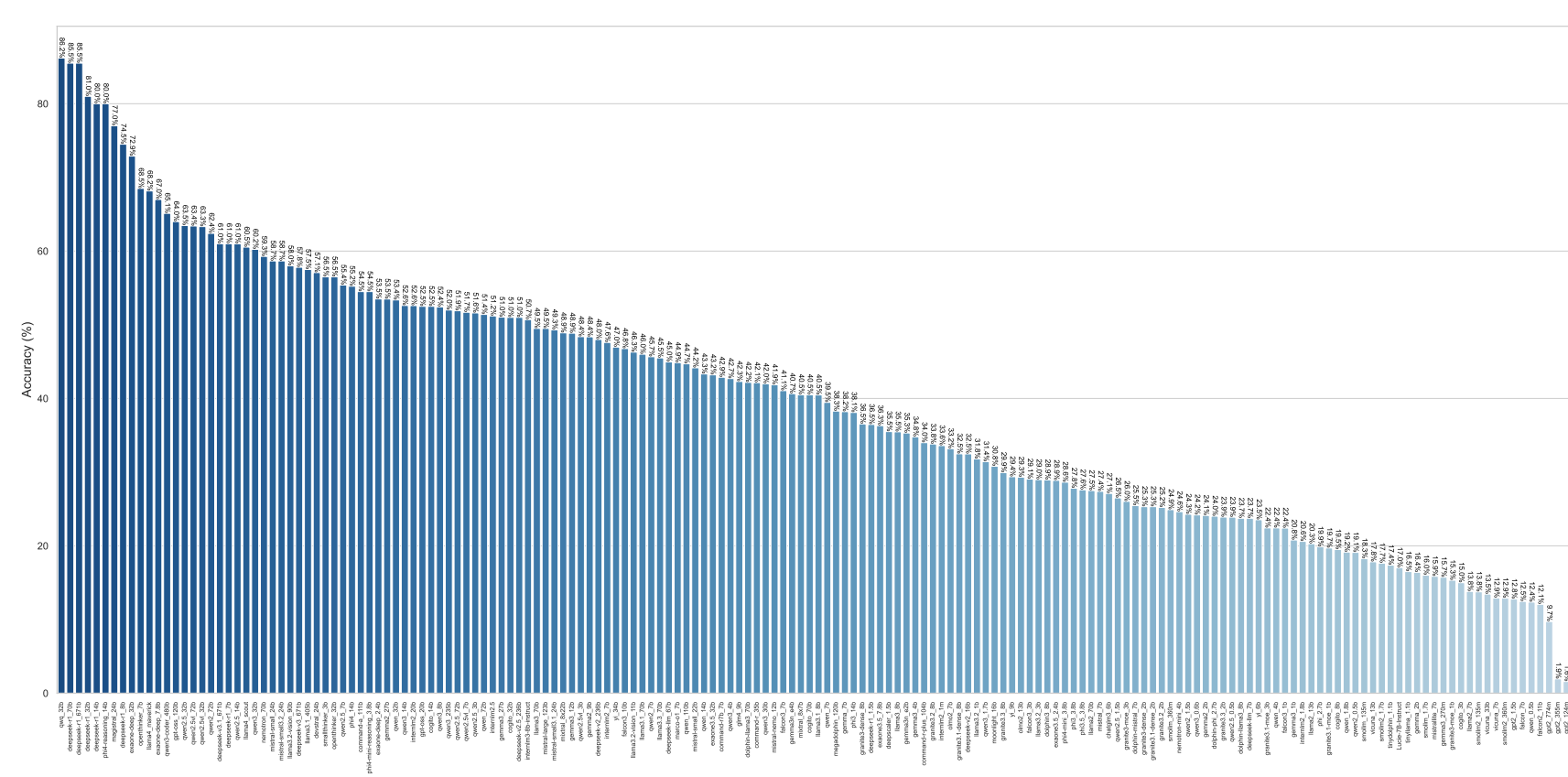

Figure S37: Bar chart of model accuracy rankings on the Attending Physician Infectious Disease examination, testing expertise in microbiology, antimicrobial therapy, infection control, and management of bacterial, viral, fungal, and parasitic infections across clinical settings. Top performers: QWQ-32b (86.2%), DeepSeek-R1-70b (85.5%), DeepSeek-R1-671b (85.5%).

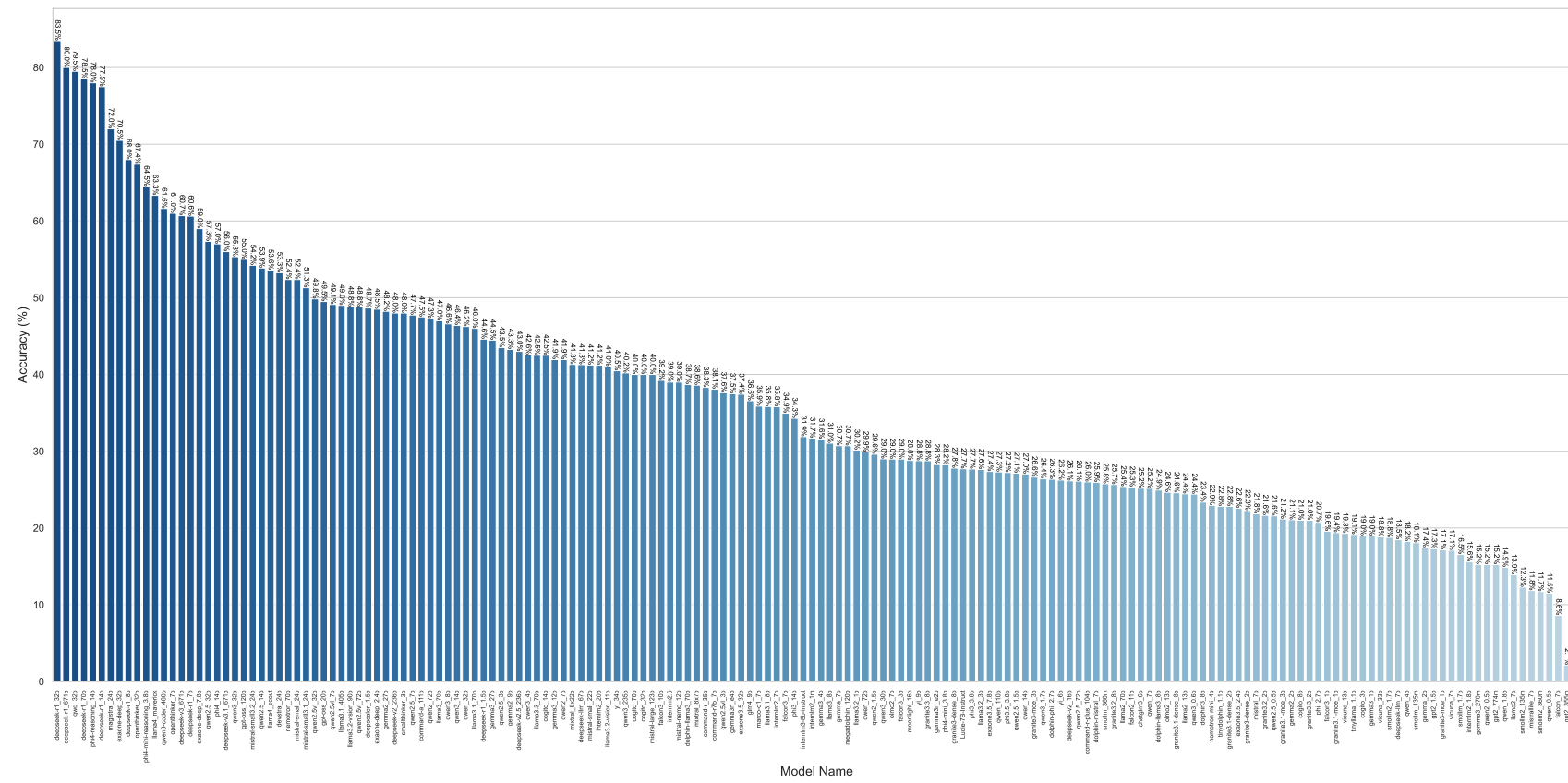

Figure S38: Bar chart of model accuracy rankings on the Attending Physician Pediatrics examination, assessing knowledge of child development, pediatric diseases, vaccination schedules, growth assessment, and age-specific treatment considerations across the neonatal-to-adolescent age spectrum. Top performers: DeepSeek-R1-32b (83.5%), DeepSeek-R1-671b (80.0%), QWQ-32b (79.5%).

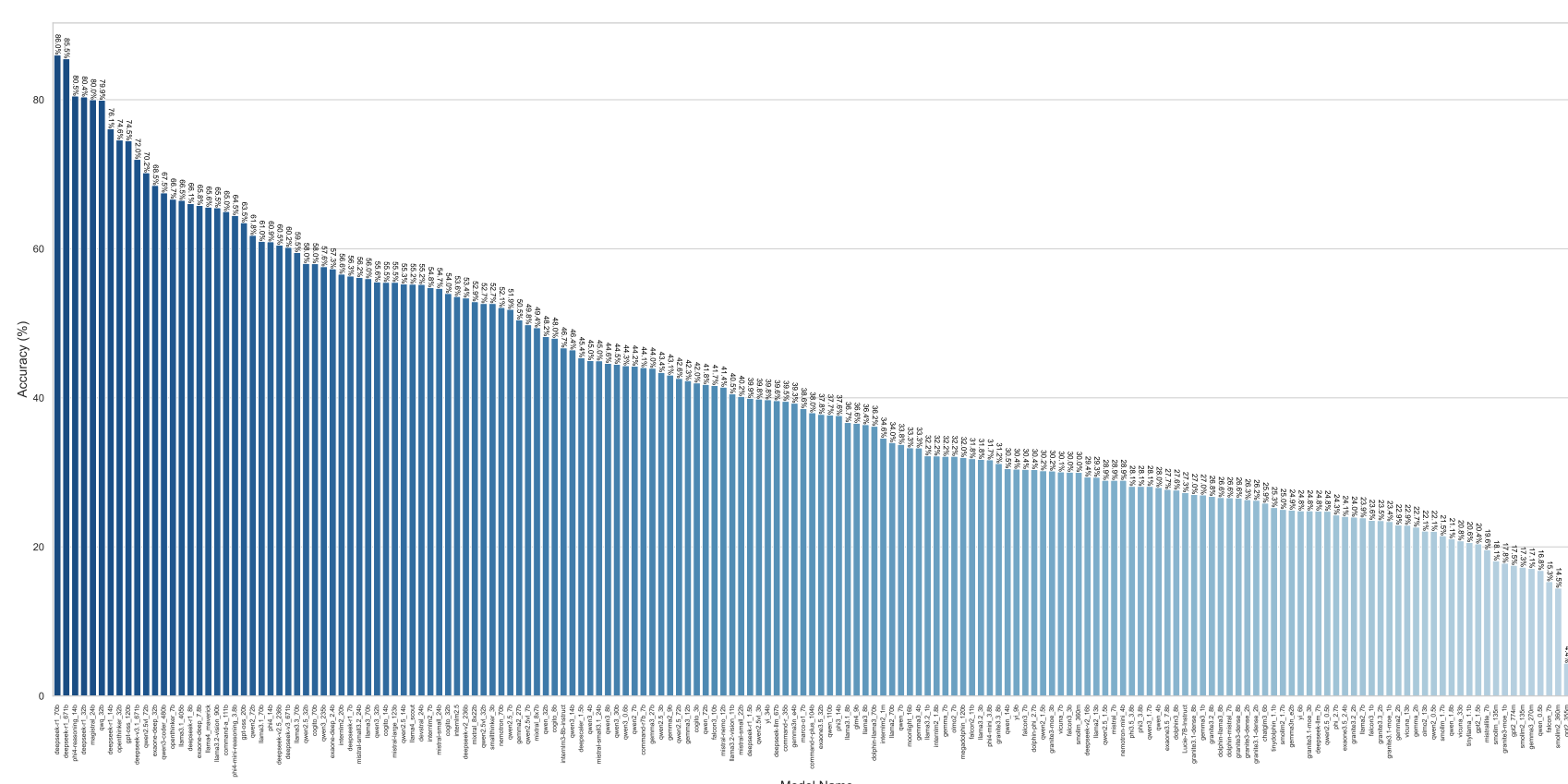

Figure S39: Bar chart of model accuracy rankings on the Attending Physician Internal Medicine examination, the broadest specialty assessment covering diagnosis and management of adult diseases across cardiovascular, pulmonary, gastrointestinal, endocrine, and other major organ systems. Top performers: DeepSeek-R1-70b (86.0%), DeepSeek-R1-671b (85.5%), Phi4-Reasoning-14b (80.5%).

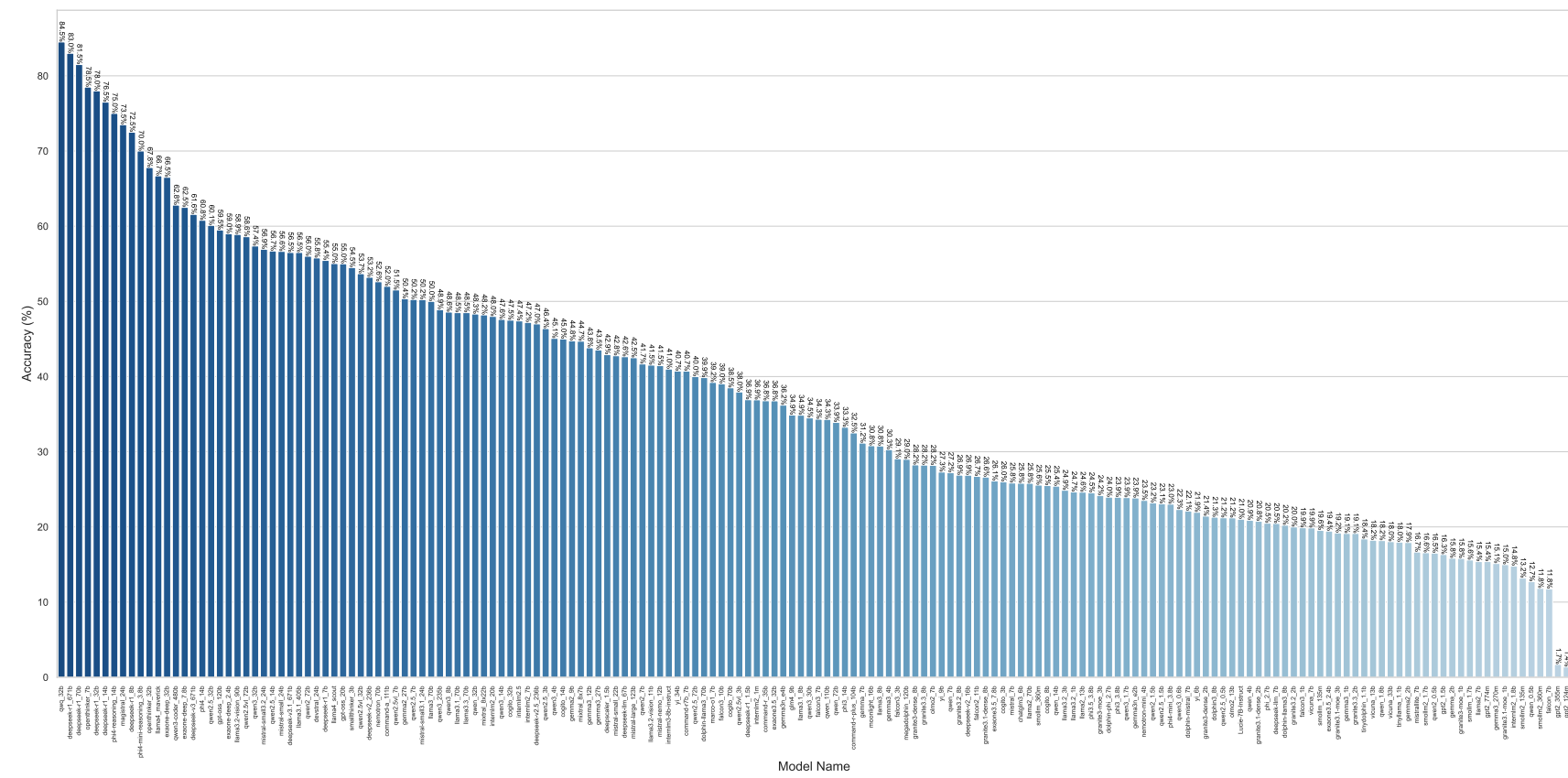

Figure S40: Bar chart of model accuracy rankings on the Attending Physician Pulmonology (Respiratory Medicine) examination, evaluating expertise in respiratory physiology, pulmonary function testing, and management of conditions including asthma, COPD, pneumonia, lung cancer, and sleep disorders. Top performers: QWQ-32b (84.5%), DeepSeek-R1-671b (83.0%), DeepSeek-R1-70b (81.5%).

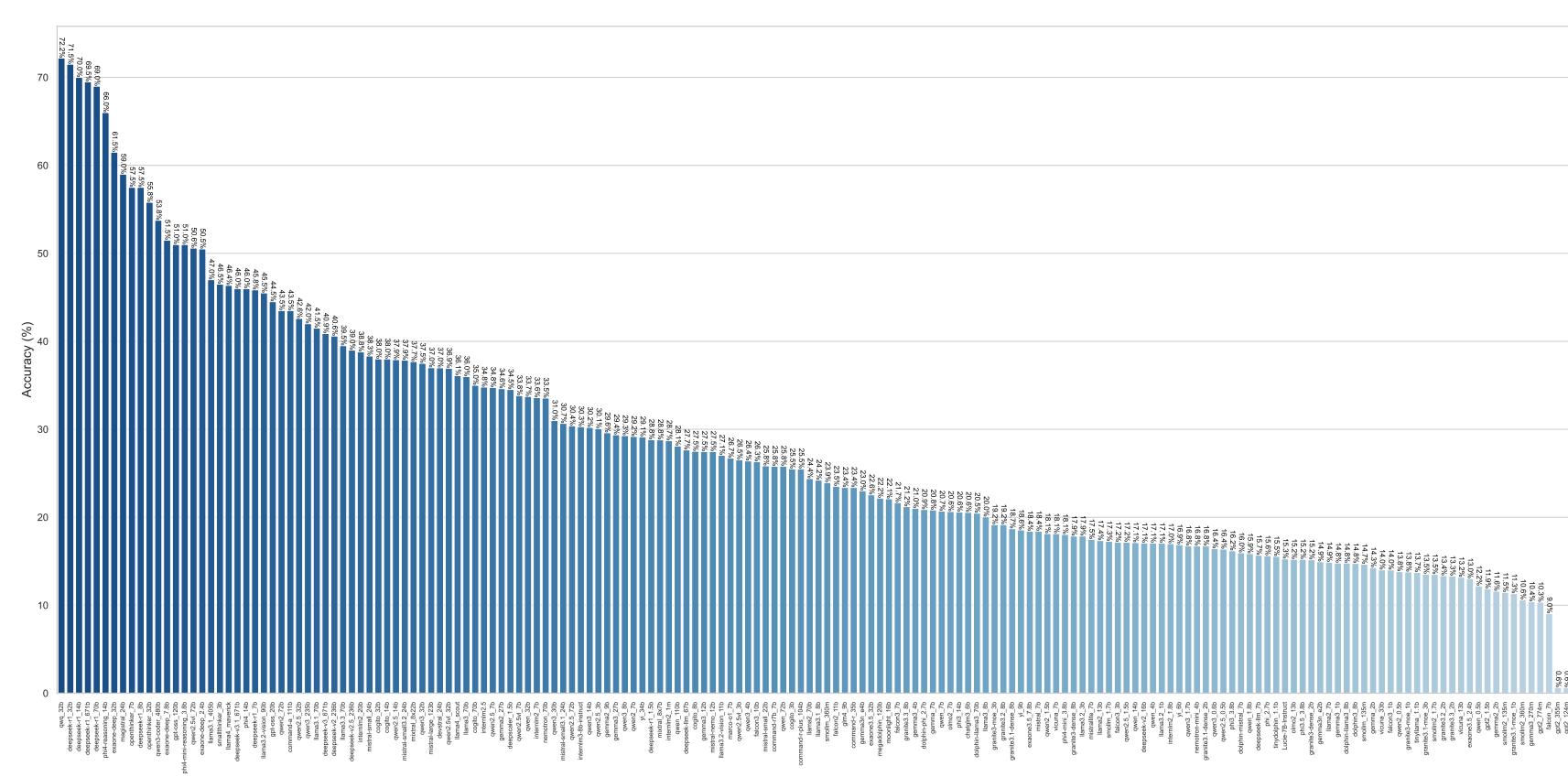

Figure S41: Bar chart of model accuracy rankings on the Attending Physician Obstetrics and Gynecology examination, assessing knowledge of pregnancy management, labor and delivery, gynecological oncology, reproductive endocrinology, and women's health across the lifespan. Top performers: QWQ-32b (72.2%), DeepSeek-R1-32b (71.5%), DeepSeek-R1-14b (70.0%).

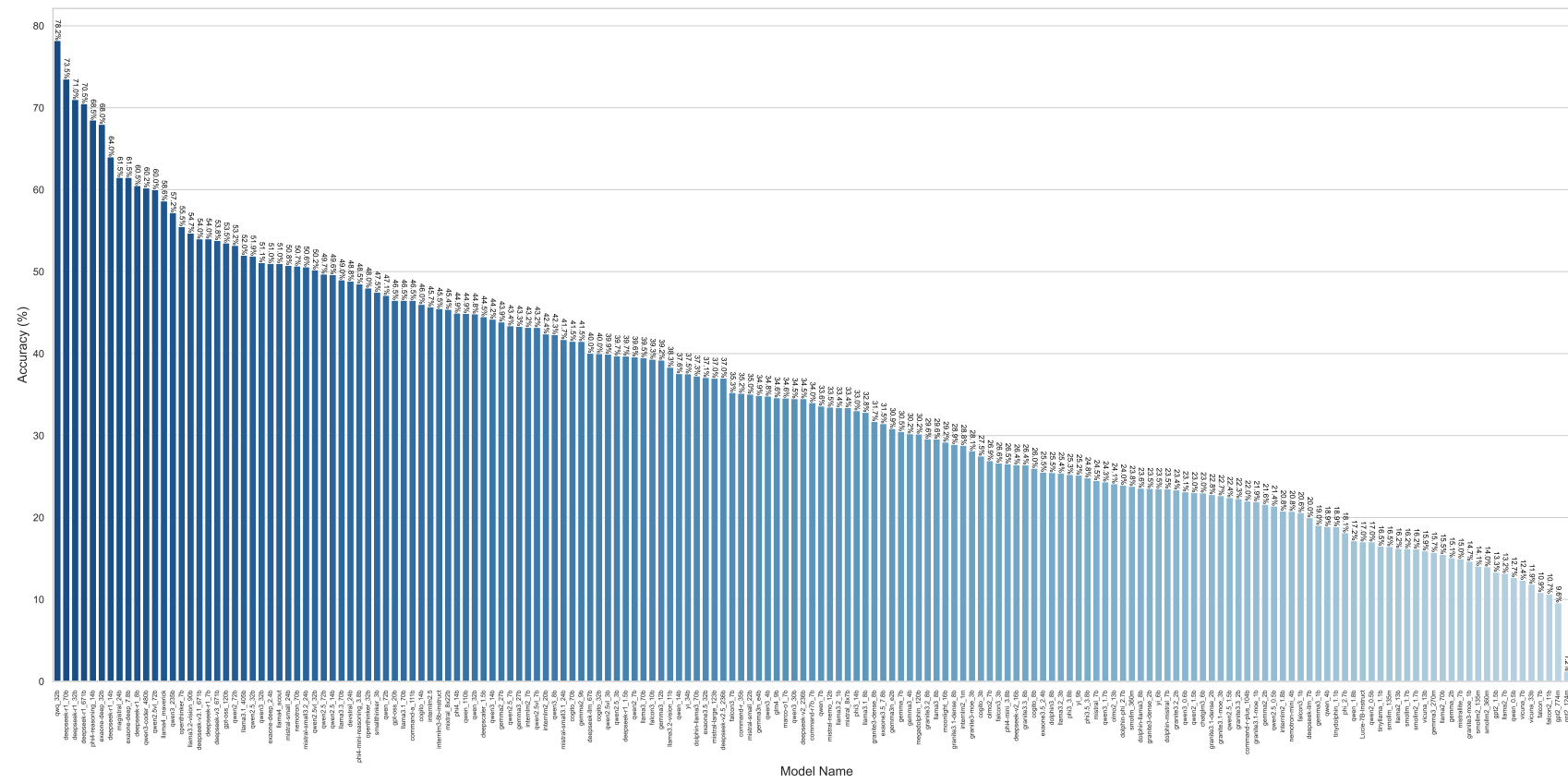

Figure S42: Bar chart of model accuracy rankings on the Attending Physician Radiology (Medical Imaging) examination, testing expertise in radiographic anatomy, imaging modality selection, pattern recognition across X-ray, CT, MRI, and ultrasound, and image-guided interventional procedures. Top performers: QWQ-32b (78.2%), DeepSeek-R1-70b (73.5%), DeepSeek-R1-32b (71.0%).

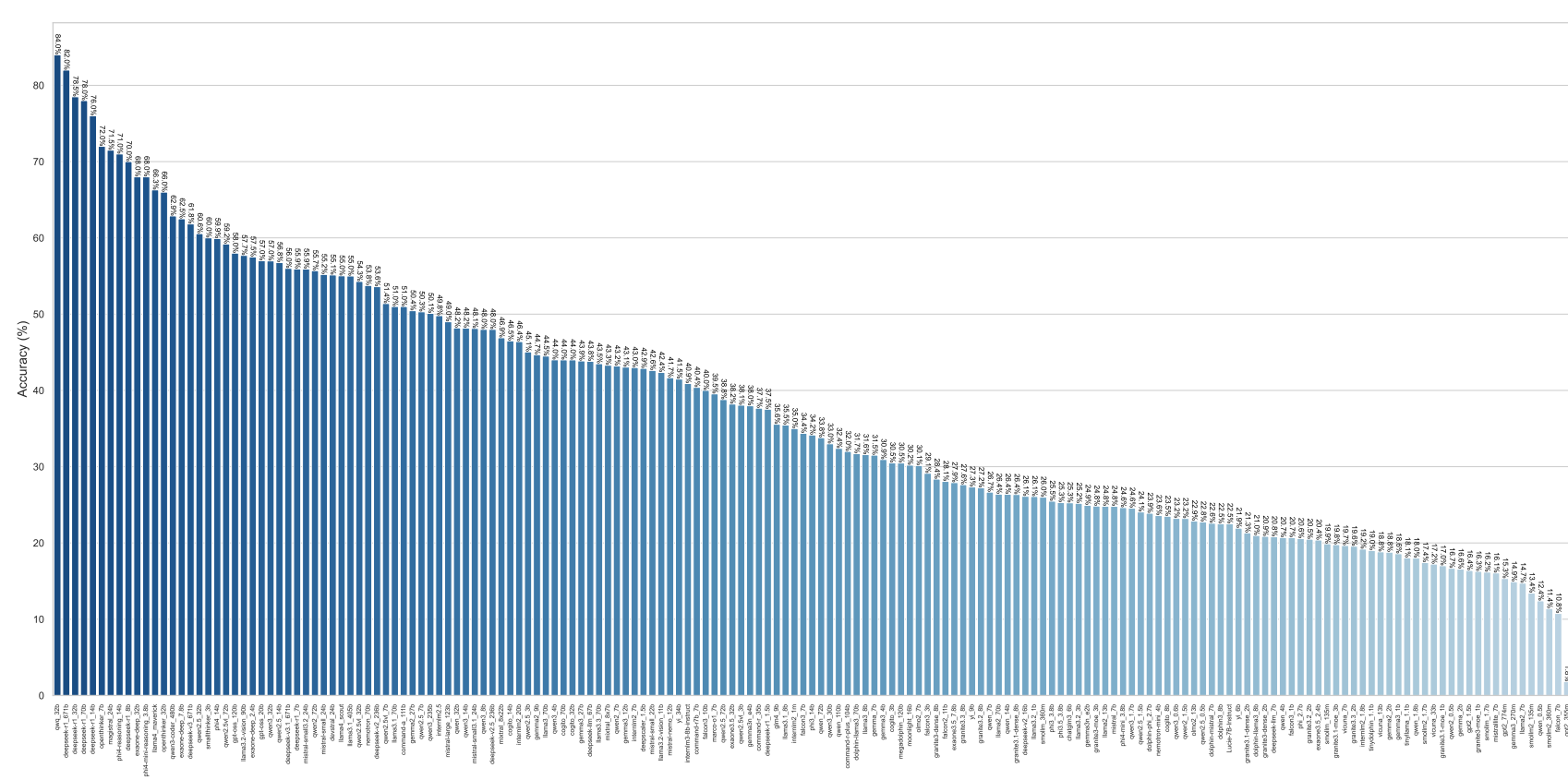

Figure S43: Bar chart of model accuracy rankings on the Attending Physician Emergency Medicine examination, evaluating rapid assessment and management of acute presentations including trauma, cardiac emergencies, toxicologic emergencies, and critical care stabilization. Top performers: QWQ-32b (84.0%), DeepSeek-R1-671b (82.0%), DeepSeek-R1-32b (78.5%).

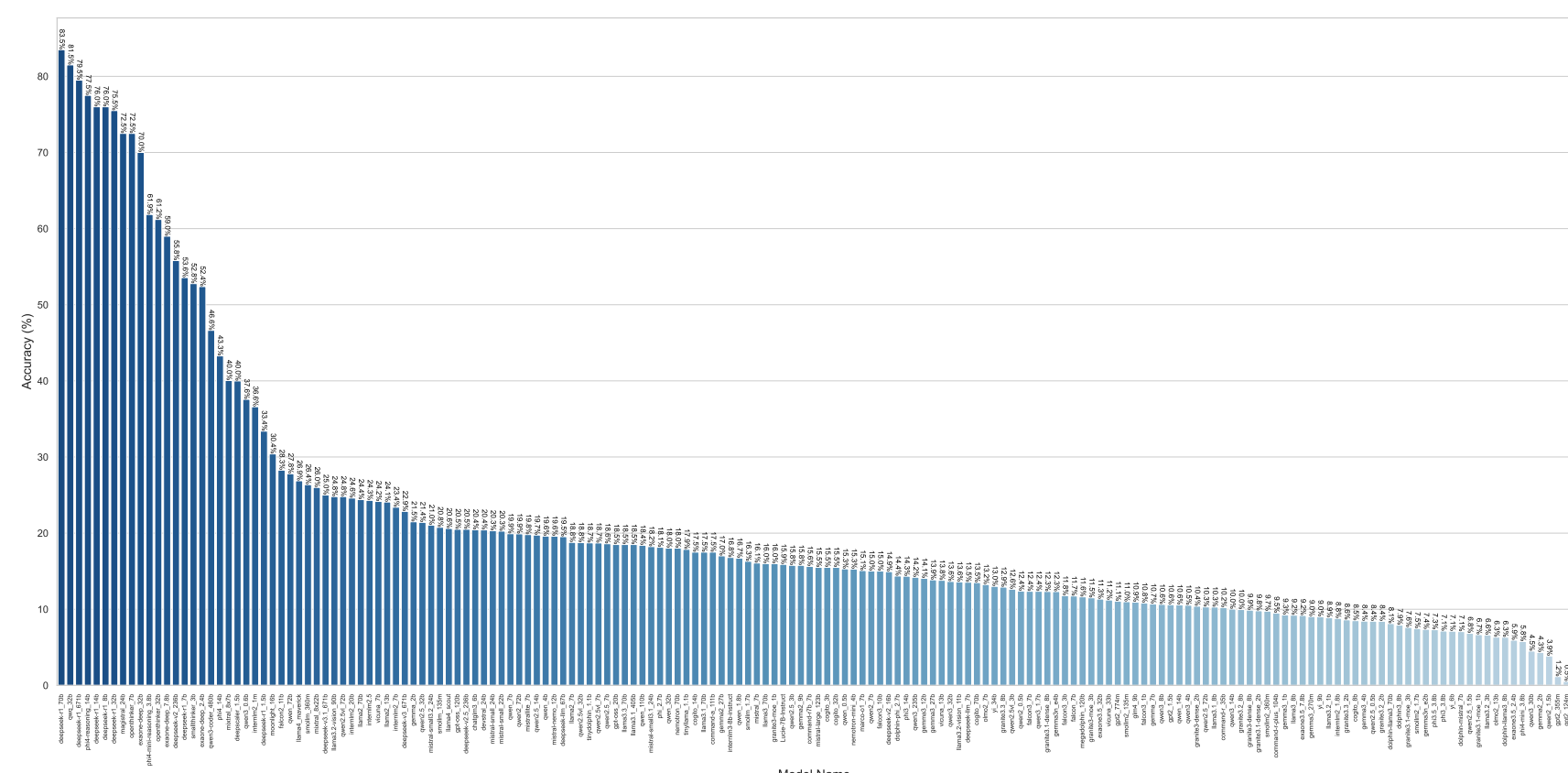

Figure S44: Bar chart of model accuracy rankings on the Attending Physician Nursing examination, assessing clinical nursing knowledge including patient assessment, care planning, medication administration, wound care, and nursing interventions across medical and surgical specialties. Top performers: DeepSeek-R1-70b (83.5%), QWQ-32b (81.5%), DeepSeek-R1-671b (79.5%).

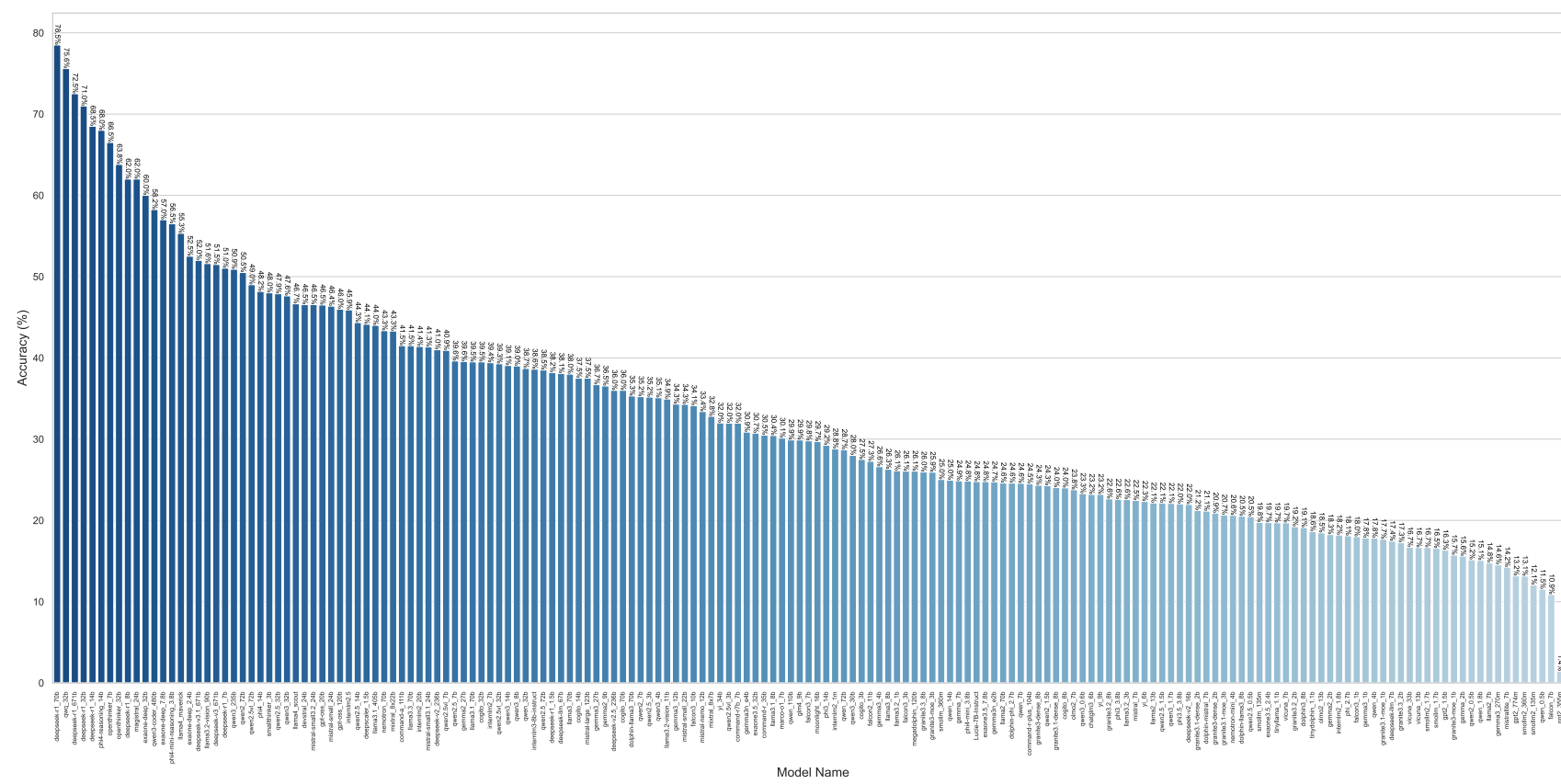

Figure S45: Bar chart of model accuracy rankings on the Attending Physician Radiation Medicine examination, testing knowledge of radiation physics, radiobiology, radiation safety, and diagnostic applications of ionizing radiation in medical imaging. Top performers: DeepSeek-R1-70b (78.5%), QWQ-32b (75.6%), DeepSeek-R1-671b (72.5%).

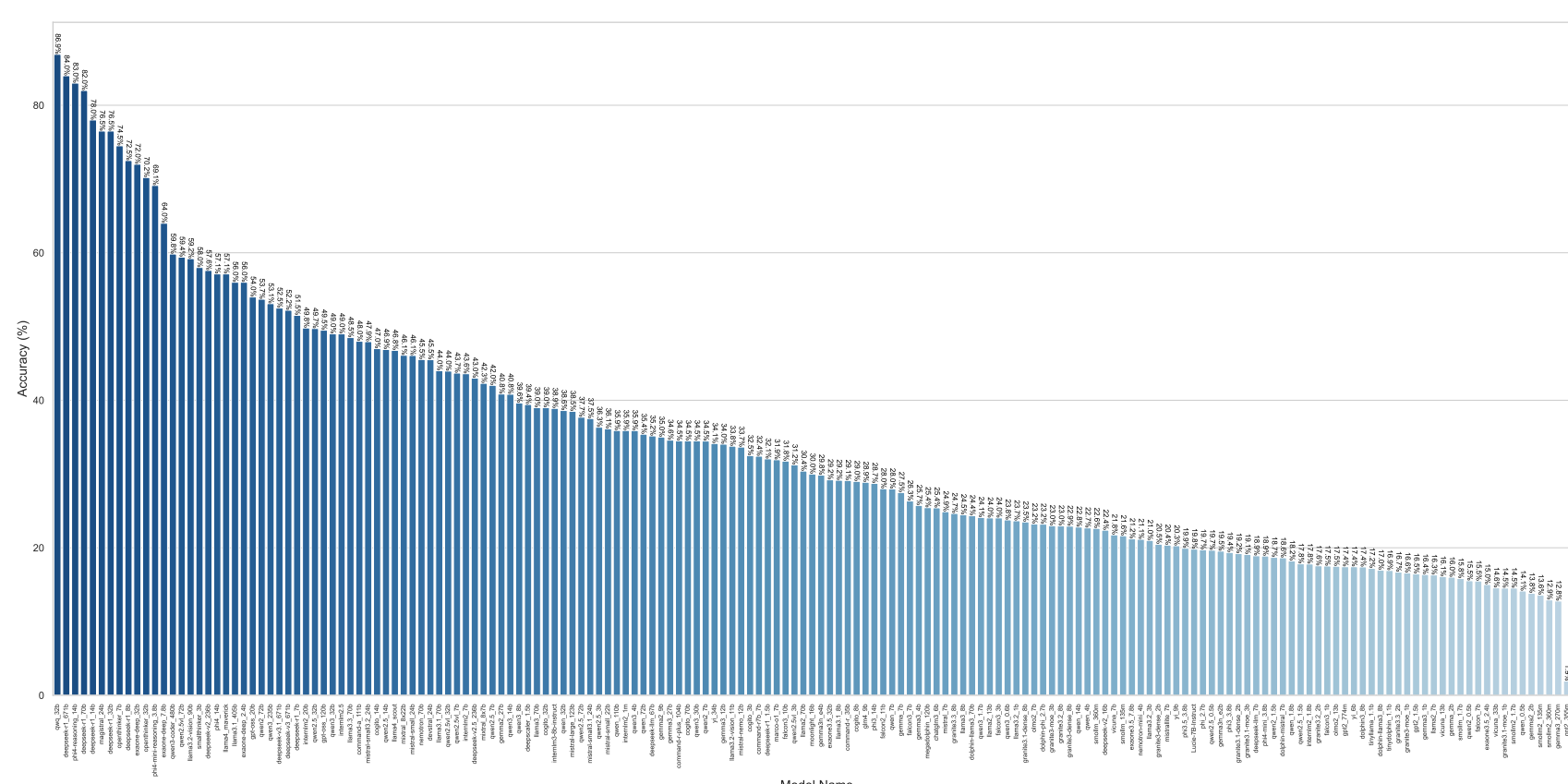

Figure S46: Bar chart of model accuracy rankings on the Attending Physician Epidemiology examination, evaluating understanding of disease surveillance, outbreak investigation, biostatistics, study design, and public health interventions for disease prevention and control. Top performers: QWQ-32b (86.9%), DeepSeek-R1-671b (84.0%), Phi4-Reasoning-14b (83.0%).

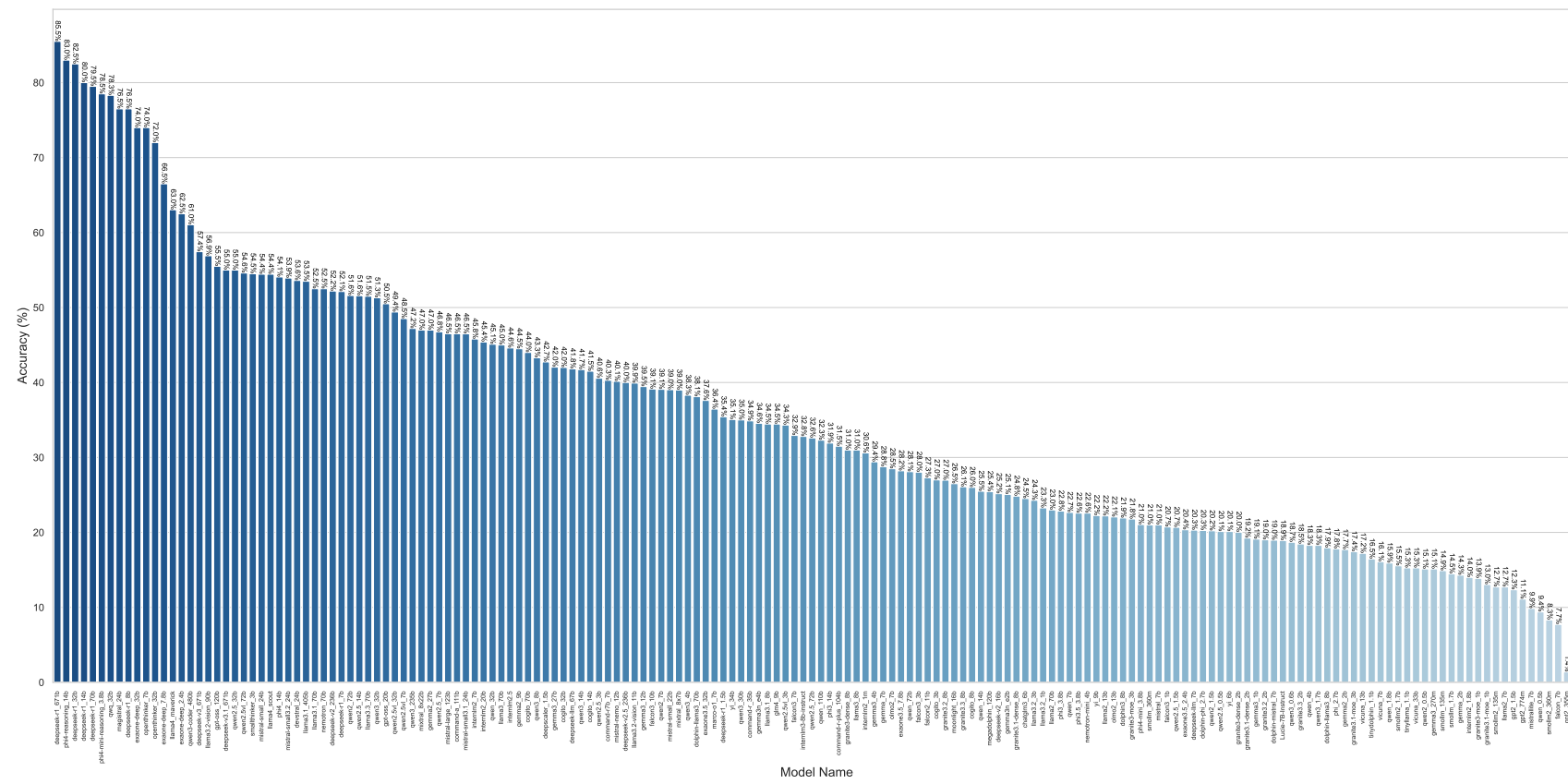

Figure S47: Bar chart of model accuracy rankings on the Attending Physician Pathology examination, assessing expertise in histopathology, cytopathology, autopsy procedures, and molecular pathology for disease diagnosis and classification. Top performers: DeepSeek-R1-671b (85.5%), Phi4-Reasoning-14b (83.0%), DeepSeek-R1-32b (82.5%).

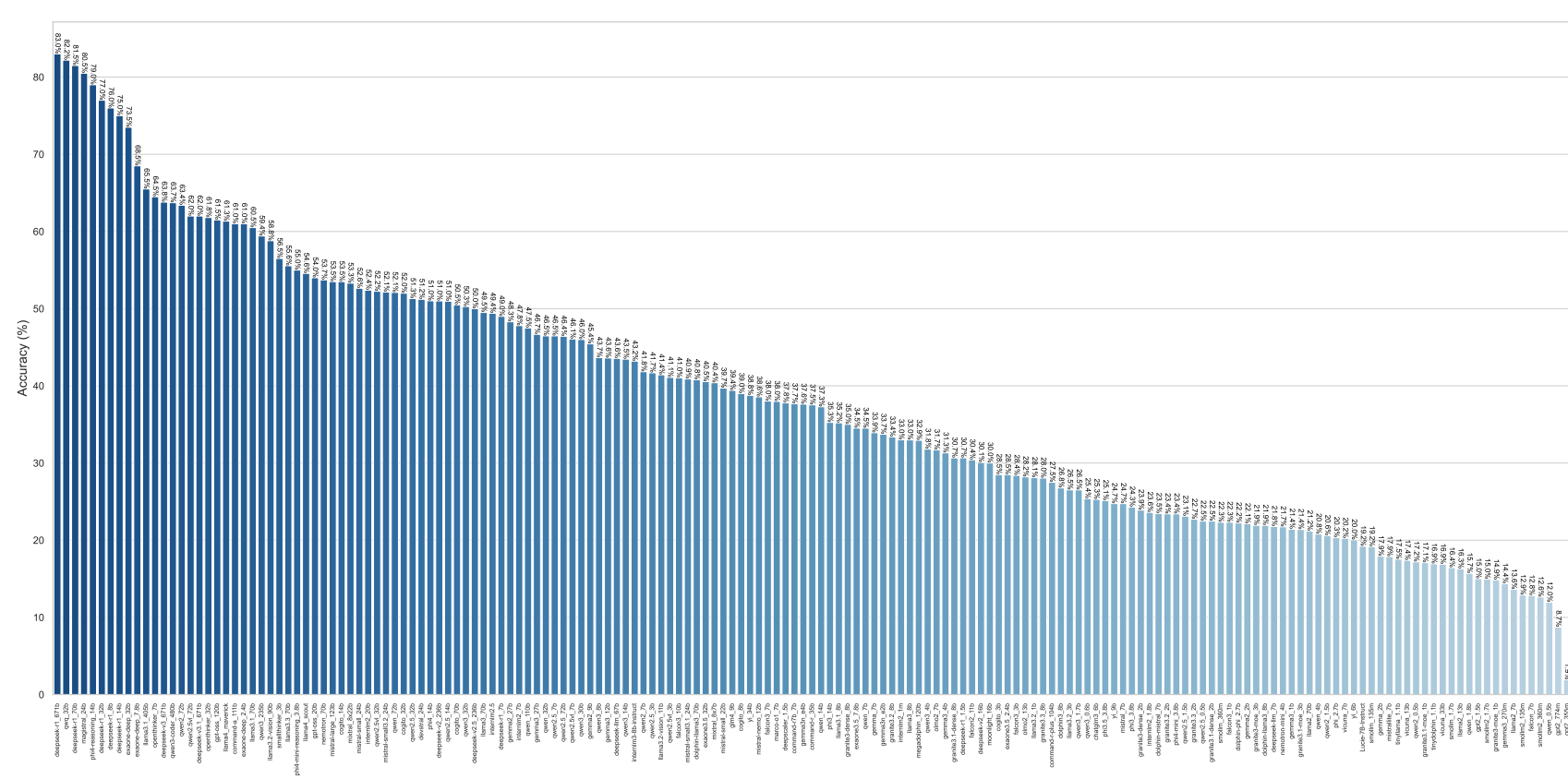

Figure S48: Bar chart of model accuracy rankings on the Attending Physician Dermatology and Venereology examination, testing knowledge of skin diseases, sexually transmitted infections, skin cancer diagnosis, and dermatologic therapeutics. Top performers: DeepSeek-R1-671b (83.0%), QWQ-32b (82.2%), DeepSeek-R1-70b (81.5%).

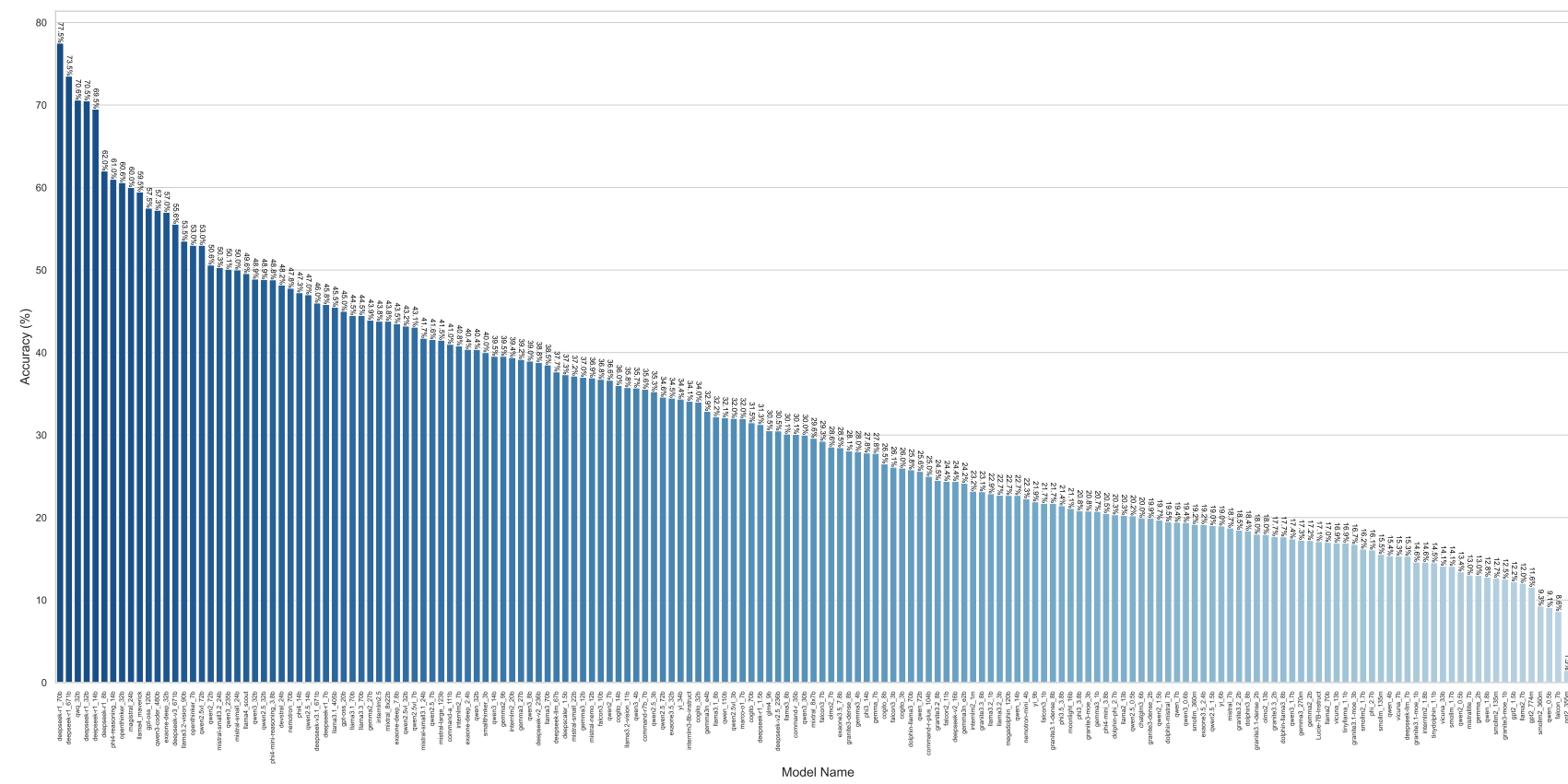

Figure S49: Bar chart of model accuracy rankings on the Attending Physician Ophthalmology examination, evaluating knowledge of ocular anatomy, refractive disorders, glaucoma, retinal diseases, and ophthalmic surgical procedures. Top performers: DeepSeek-R1-70b (77.5%), DeepSeek-R1-671b (73.5%), QWQ-32b (70.6%).

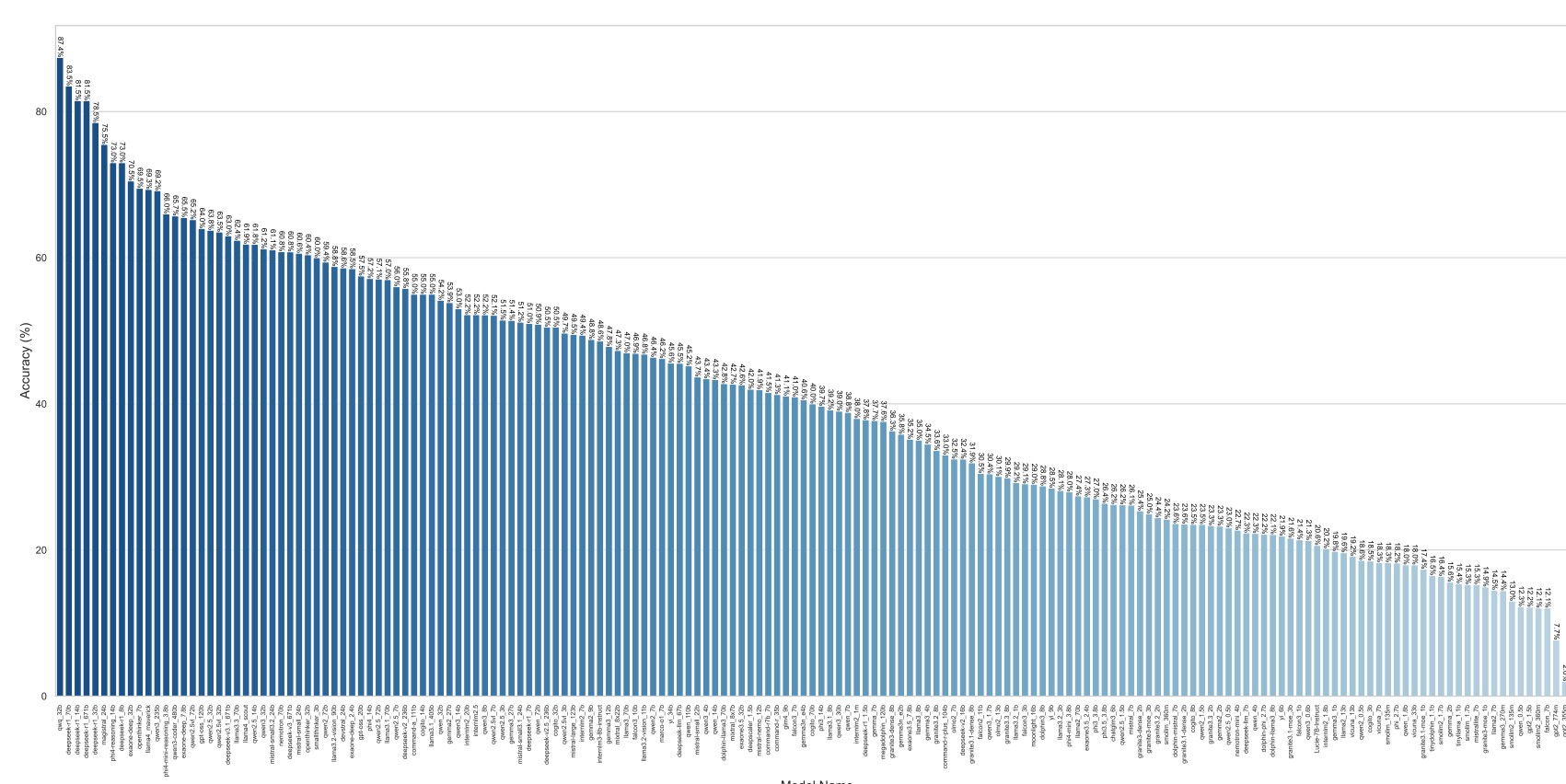

Figure S50: Bar chart of model accuracy rankings on the Attending Physician Neurology examination, assessing expertise in neurological examination, stroke management, epilepsy, movement disorders, neurodegenerative diseases, and neuromuscular conditions. Top performers: QWQ-32b (87.4%), DeepSeek-R1-70b (83.5%), DeepSeek-R1-14b (81.5%).

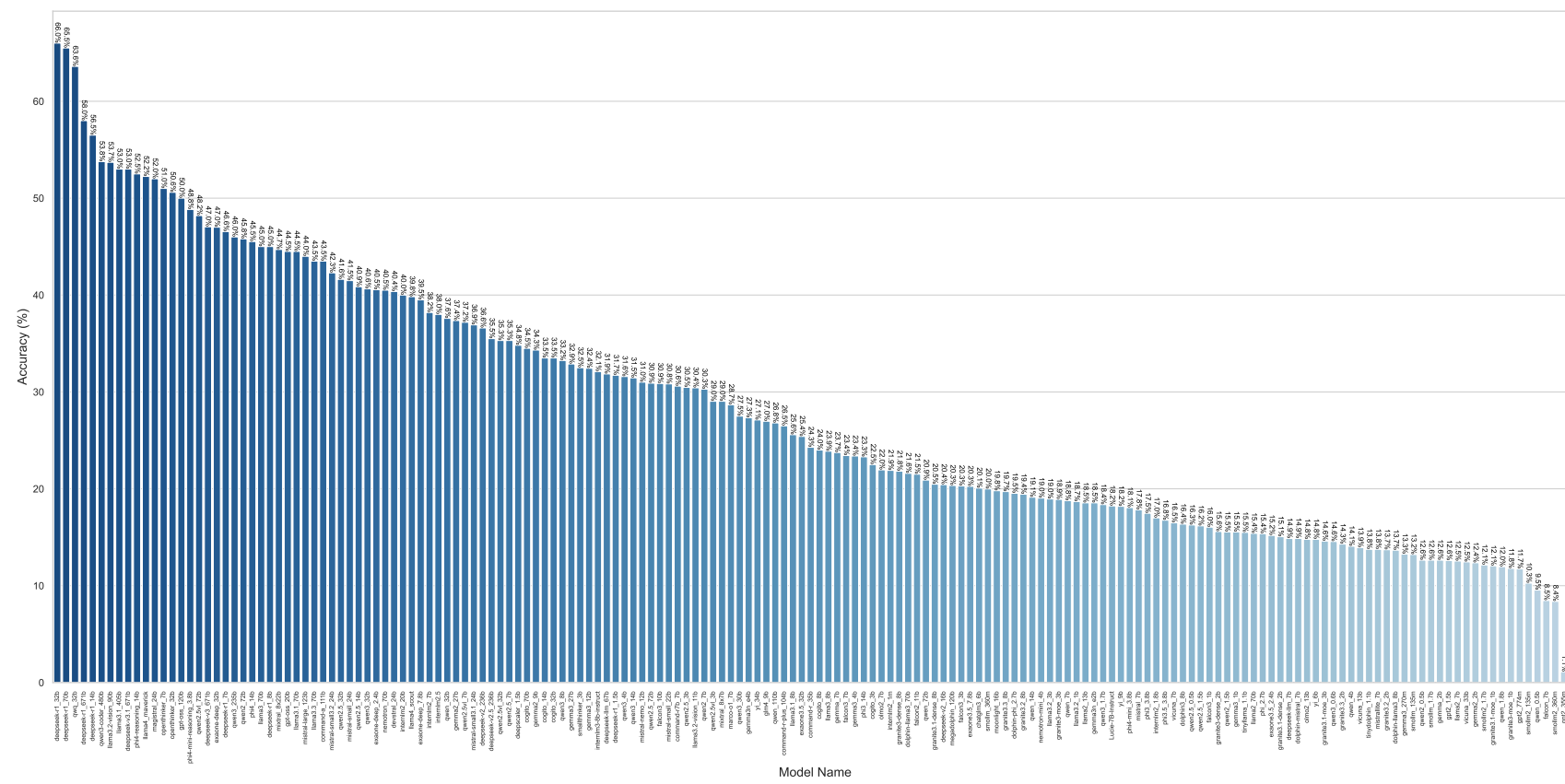

Figure S51: Bar chart of model accuracy rankings on the Attending Physician Otolaryngology (ENT) examination, testing knowledge of ear, nose, throat, and head-neck pathology including hearing disorders, sinusitis, laryngeal diseases, and head-neck oncology. Top performers: DeepSeek-R1-32b (66.0%), DeepSeek-R1-70b (65.5%), QWQ-32b (63.6%).

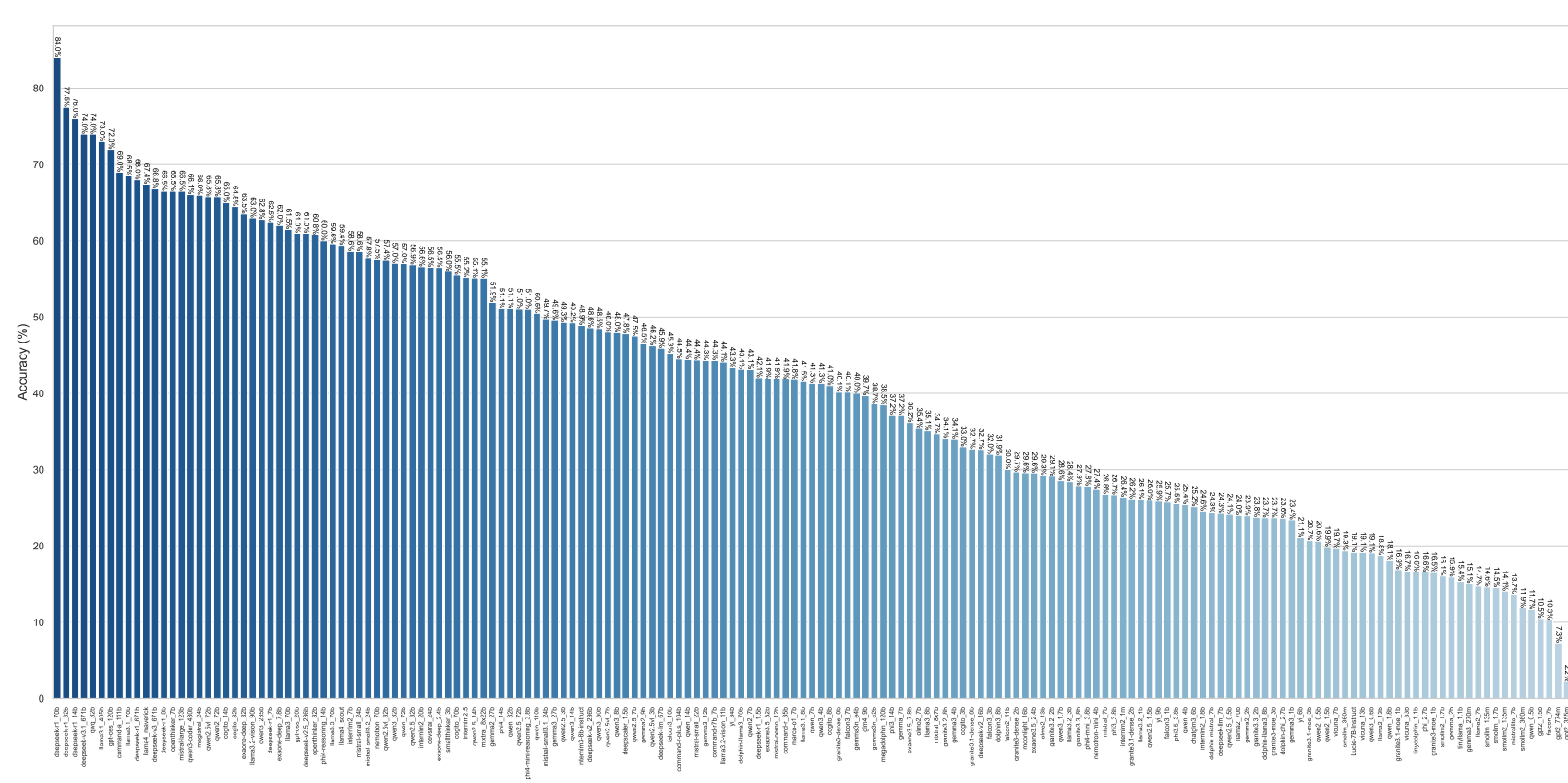

Figure S52: Bar chart of model accuracy rankings on the Attending Physician Medical Oncology examination, evaluating knowledge of cancer biology, chemotherapy protocols, targeted therapies, immunotherapy, and supportive care in oncology. Top performers: DeepSeek-R1-70b (84.0%), DeepSeek-R1-32b (77.5%), DeepSeek-R1-14b (76.0%).

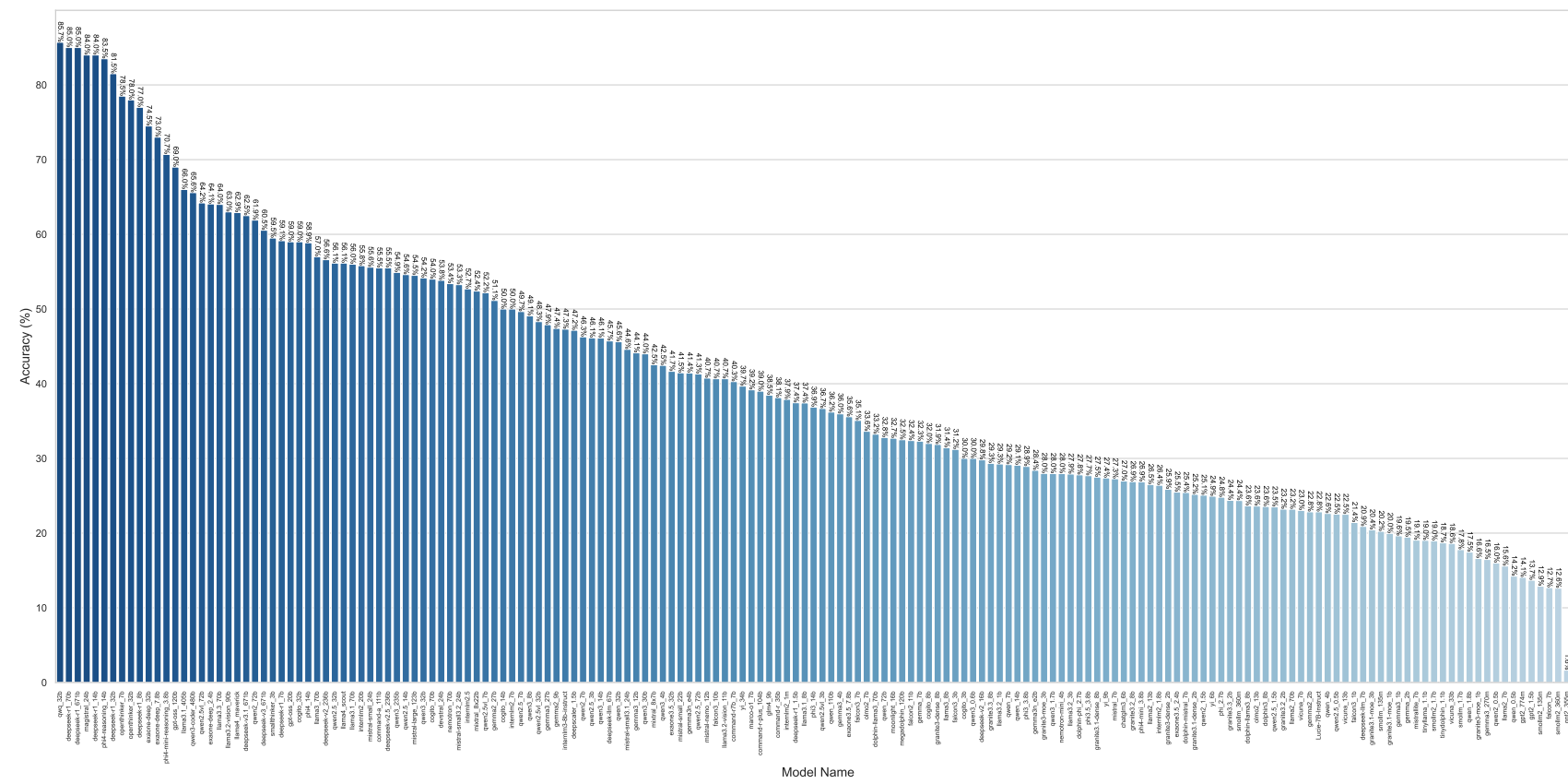

Figure S53: Bar chart of model accuracy rankings on the Attending Physician Nutrition examination, assessing expertise in nutritional assessment, therapeutic diets, enteral and parenteral nutrition, and management of nutritional disorders across clinical settings. Top performers: QWQ-32b (85.7%), DeepSeek-R1-70b (85.0%), DeepSeek-R1-671b (85.0%).

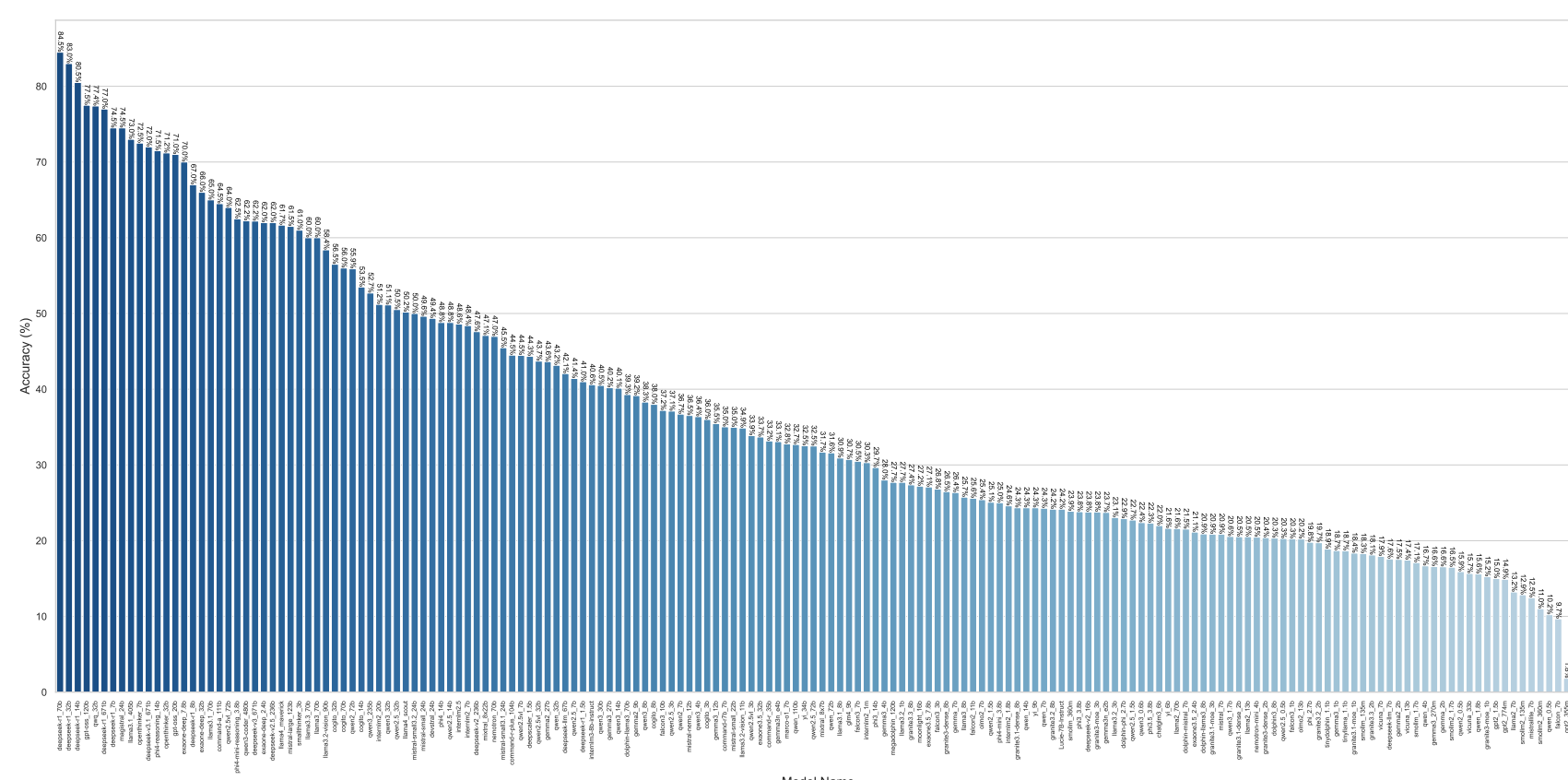

Figure S54: Bar chart of model accuracy rankings on the Attending Physician Ultrasound Medicine examination, testing knowledge of ultrasound physics, sonographic anatomy, and diagnostic applications in obstetrics, cardiology, abdominal imaging, and vascular assessment. Top performers: DeepSeek-R1-70b (84.5%), DeepSeek-R1-32b (83.0%), DeepSeek-R1-14b (80.5%).

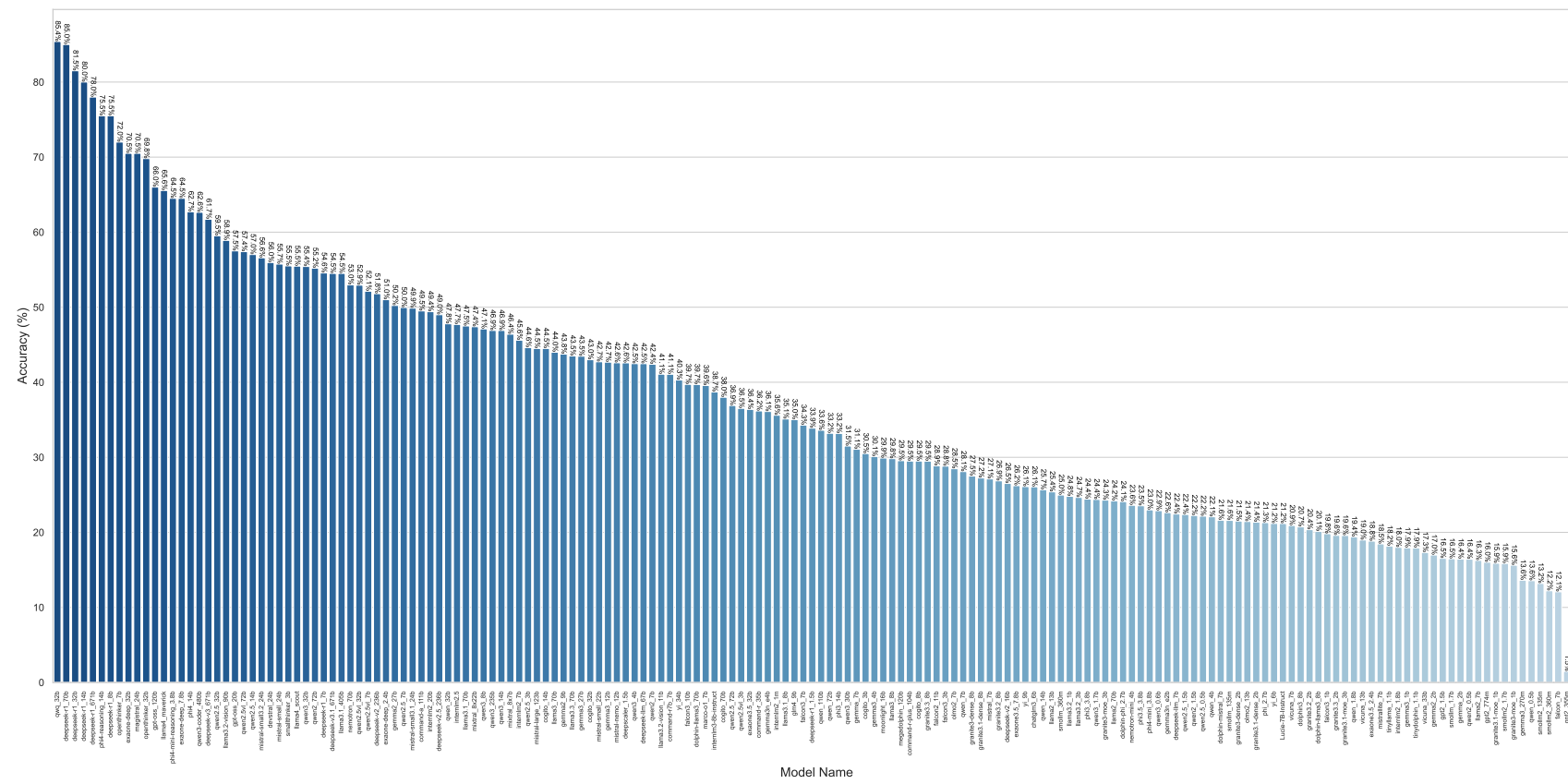

Figure S55: Bar chart of model accuracy rankings on the Attending Physician Rheumatology and Immunology examination, evaluating expertise in autoimmune diseases, inflammatory arthritis, connective tissue disorders, and immunomodulatory therapies. Top performers: QWQ-32b (85.4%), DeepSeek-R1-70b (85.0%), DeepSeek-R1-32b (81.5%).

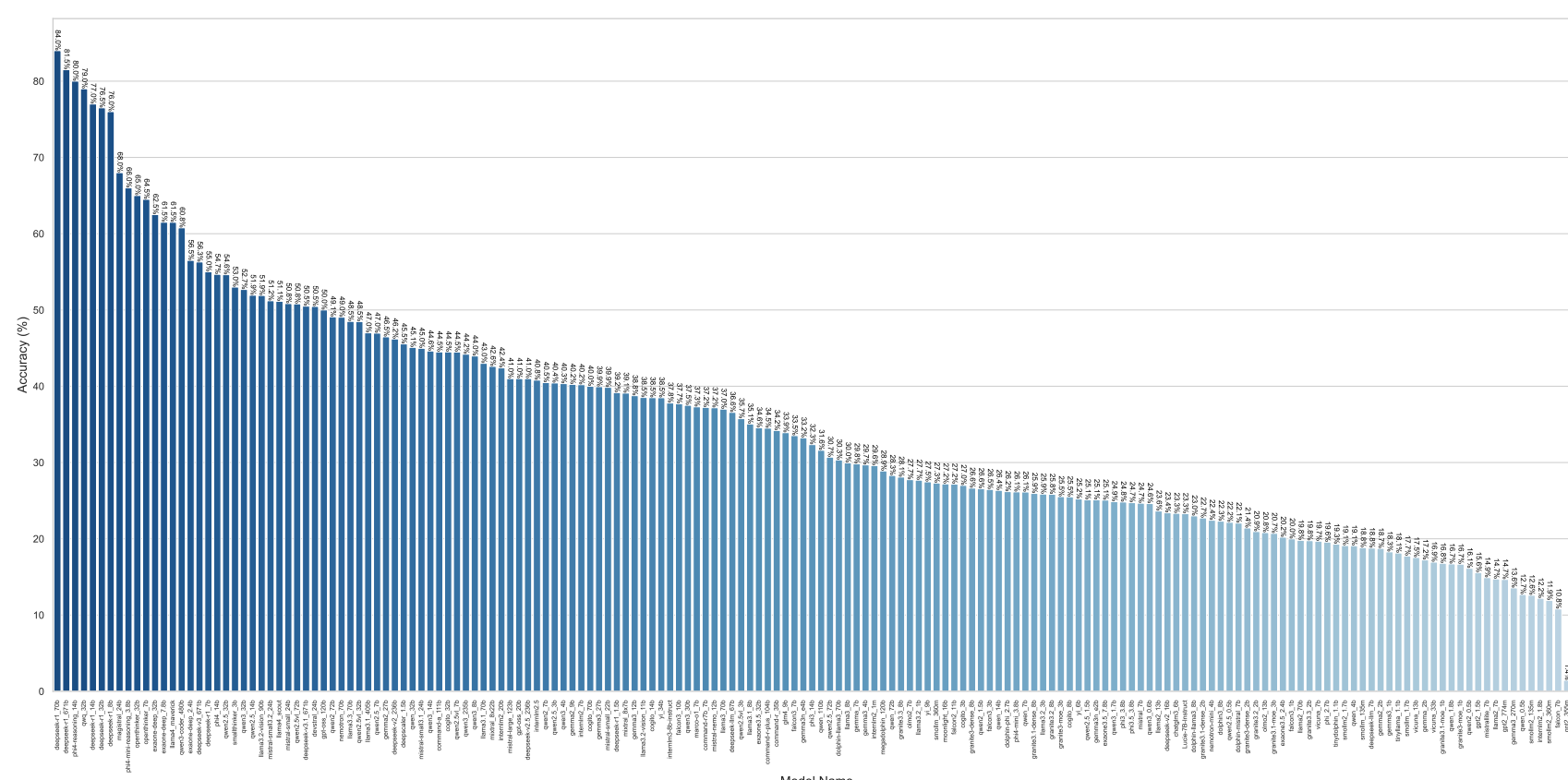

Figure S56: Bar chart of model accuracy rankings on the Attending Physician Orthopedics examination, assessing knowledge of musculoskeletal anatomy, fracture management, joint disorders, spine pathology, and orthopedic surgical principles. Top performers: DeepSeek-R1-70b (84.0%), DeepSeek-R1-671b (81.5%), Phi4-Reasoning-14b (80.0%).

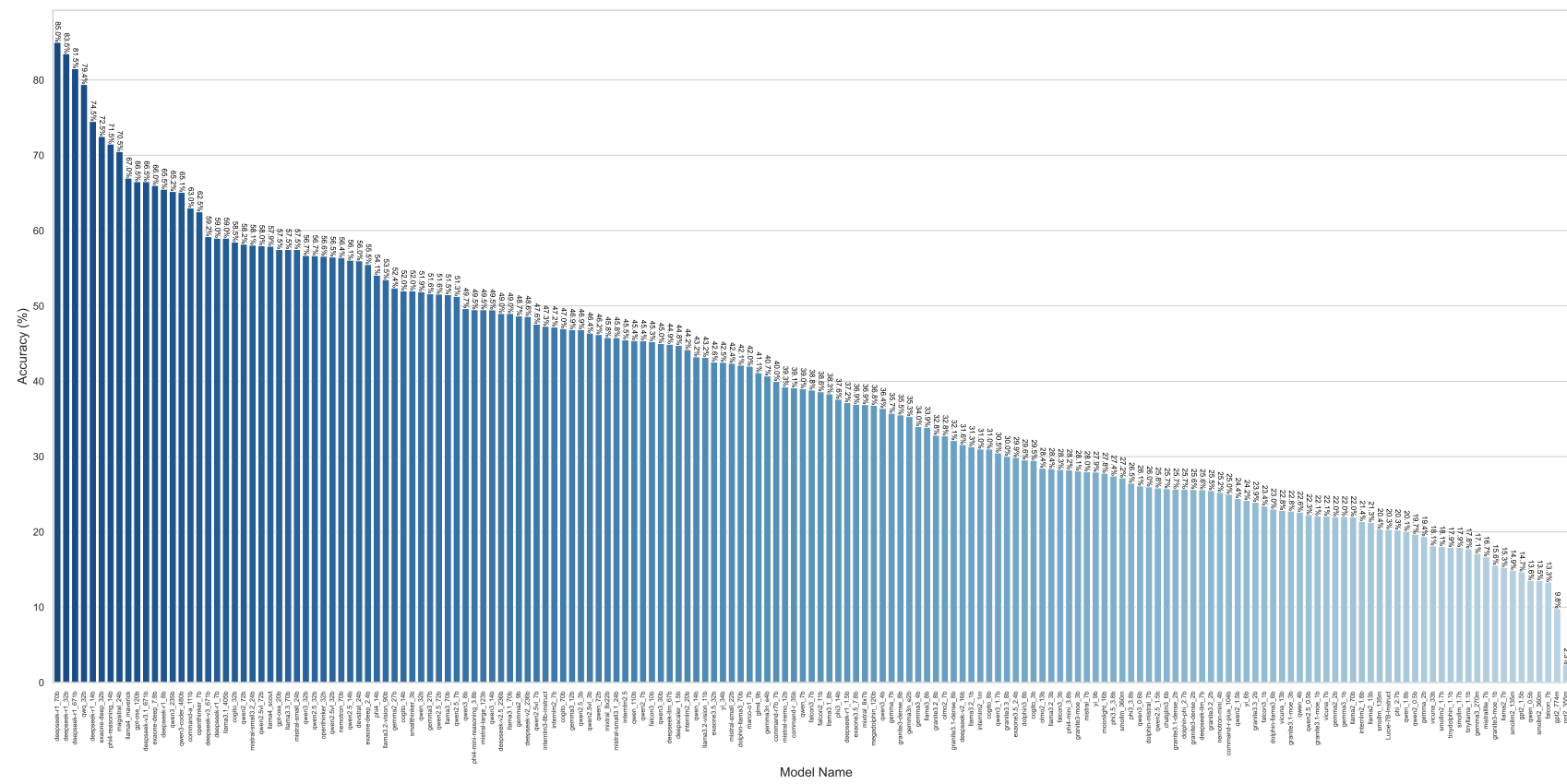

Figure S57: Bar chart of model accuracy rankings on the Associate Chief Physician Clinical Medicine examination, representing senior-level comprehensive assessment of internal medicine knowledge including complex differential diagnosis and advanced therapeutic decision-making. Top performers: DeepSeek-R1-70b (85.0%), DeepSeek-R1-32b (83.5%), DeepSeek-R1-671b (81.5%).

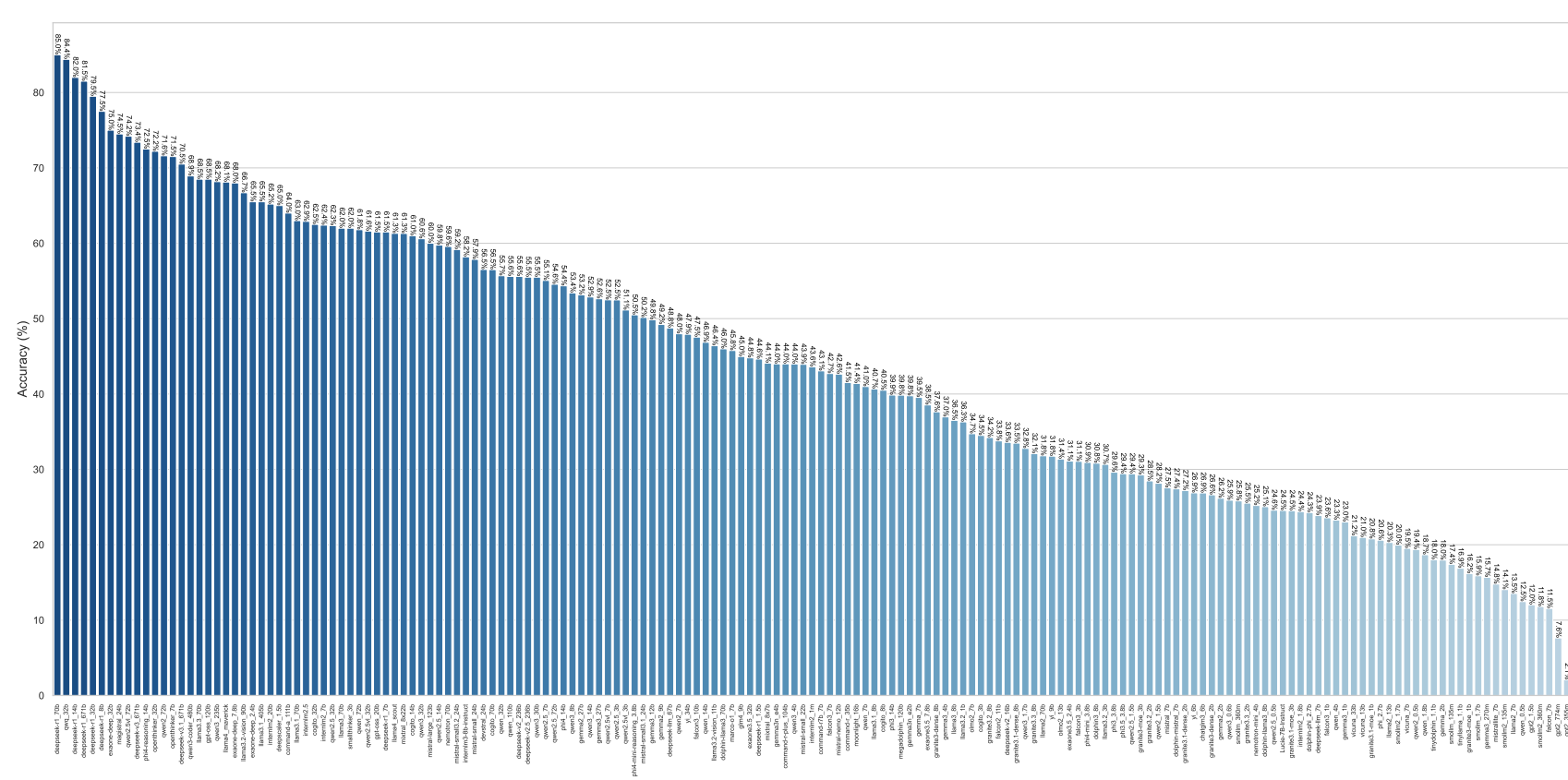

Figure S58: Bar chart of model accuracy rankings on the Associate Chief Physician General Practice examination, assessing senior-level primary care expertise including chronic disease management, preventive medicine, and coordination of multidisciplinary care. Top performers: DeepSeek-R1-70b (85.0%), QWQ-32b (84.4%), DeepSeek-R1-14b (82.0%).

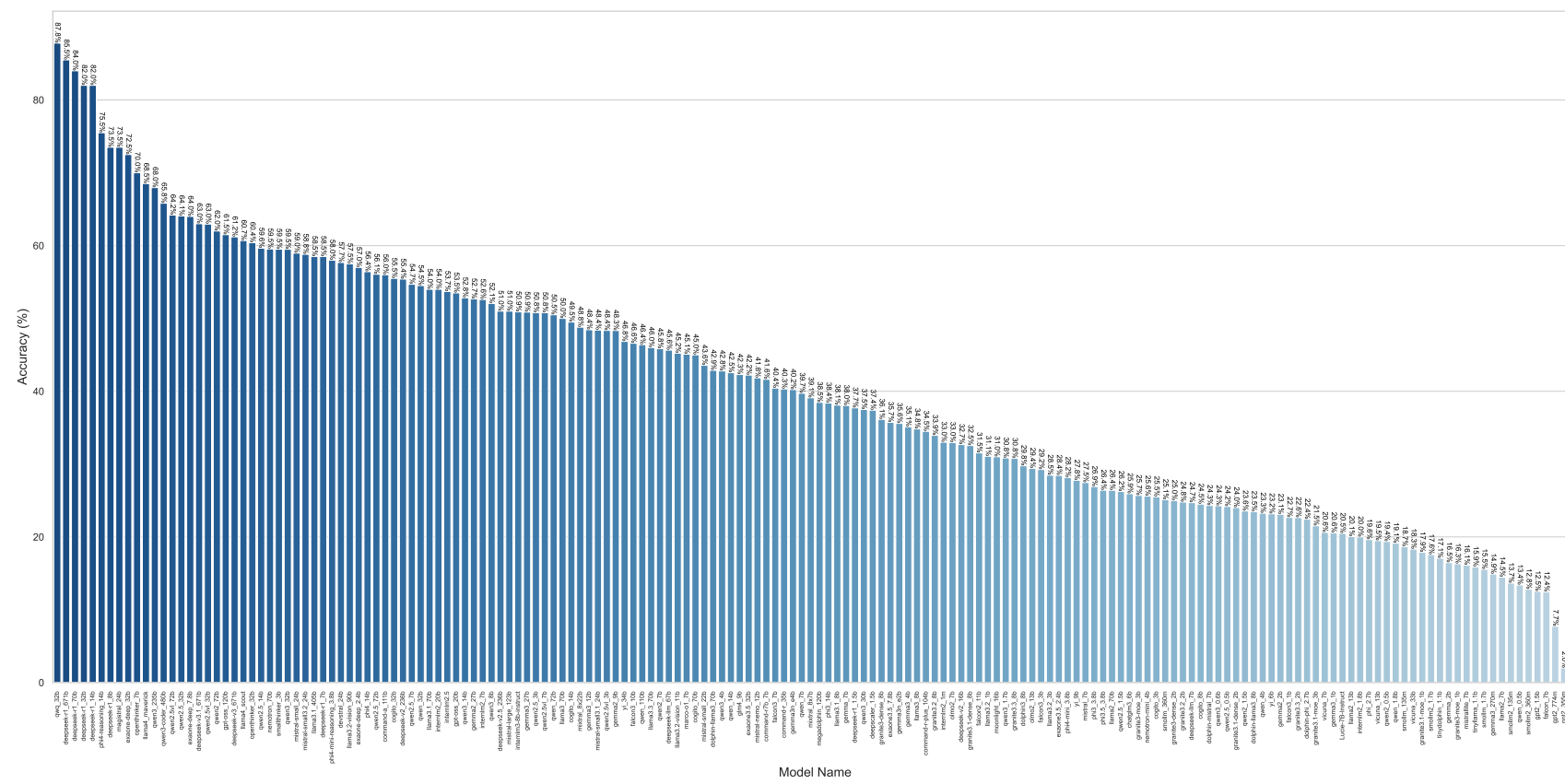

Figure S59: Bar chart of model accuracy rankings on the Associate Chief Physician Endocrinology examination, evaluating advanced knowledge of hormonal disorders, diabetes management, thyroid diseases, metabolic bone disease, and pituitary-adrenal pathology. Top performers: QWQ-32b (87.8%), DeepSeek-R1-671b (85.5%), DeepSeek-R1-70b (84.0%).

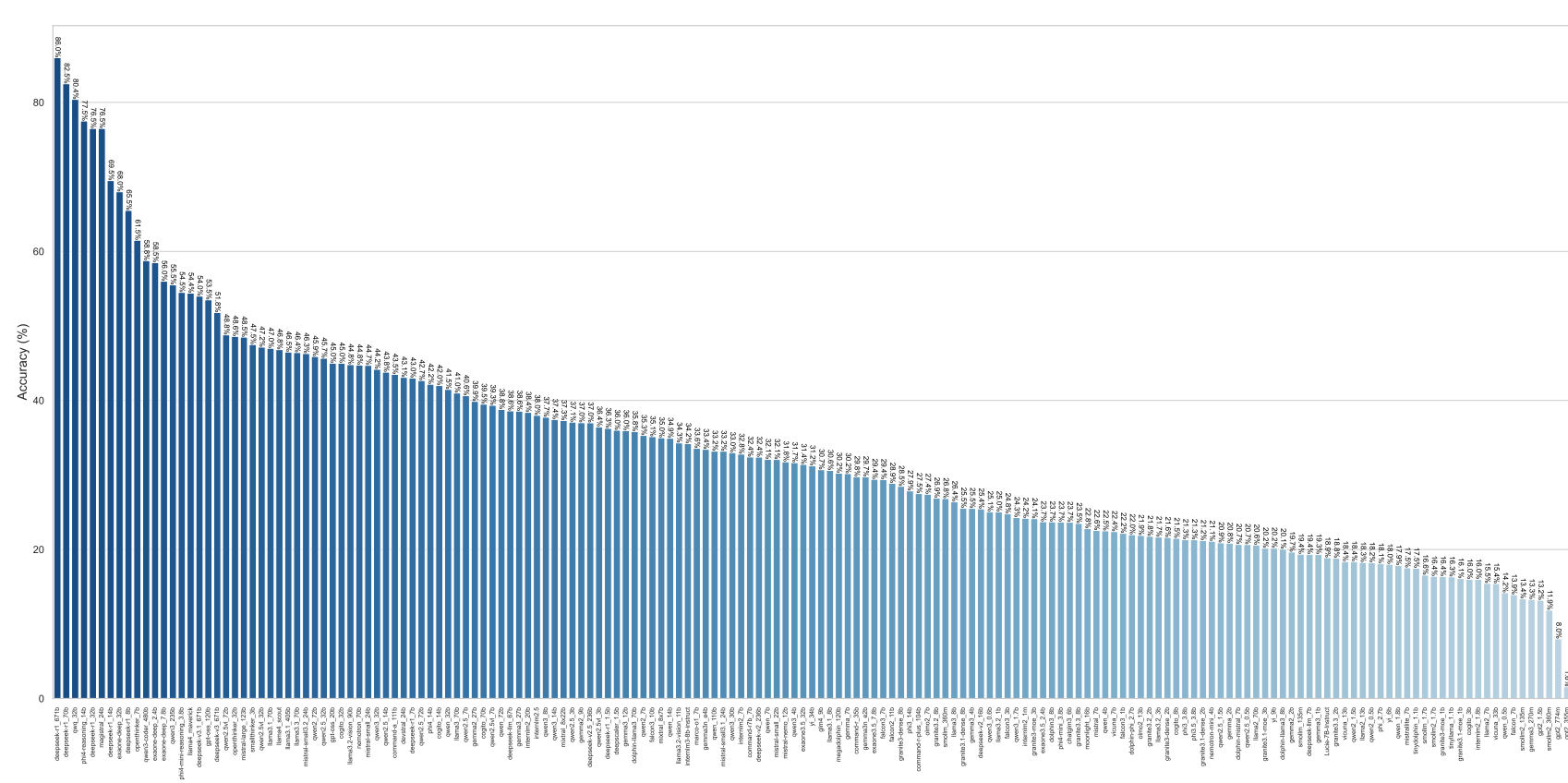

Figure S60: Bar chart of model accuracy rankings on the Associate Chief Physician Dentistry (Oral Medicine) examination, testing senior-level expertise in oral pathology, maxillofacial surgery, periodontics, and dental therapeutics. Top performers: DeepSeek-R1-671b (86.0%), DeepSeek-R1-70b (82.5%), QWQ-32b (80.4%).

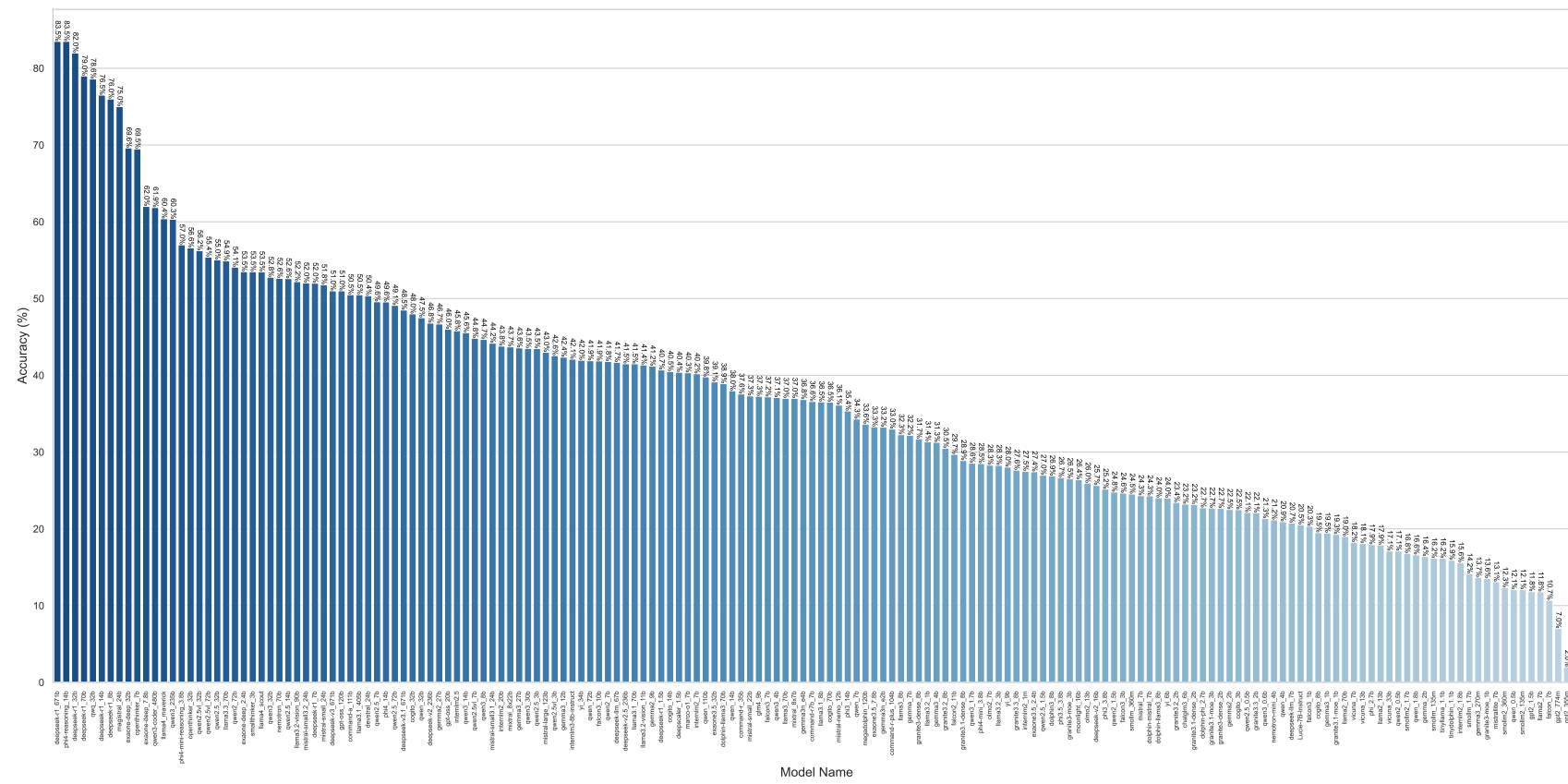

Figure S61: Bar chart of model accuracy rankings on the Associate Chief Physician Pediatric Surgery examination, assessing expertise in congenital malformations, pediatric trauma, neonatal surgery, and surgical management of childhood conditions. Top performers: DeepSeek-R1-671b (83.5%), Phi4-Reasoning-14b (83.5%), DeepSeek-R1-32b (82.0%).

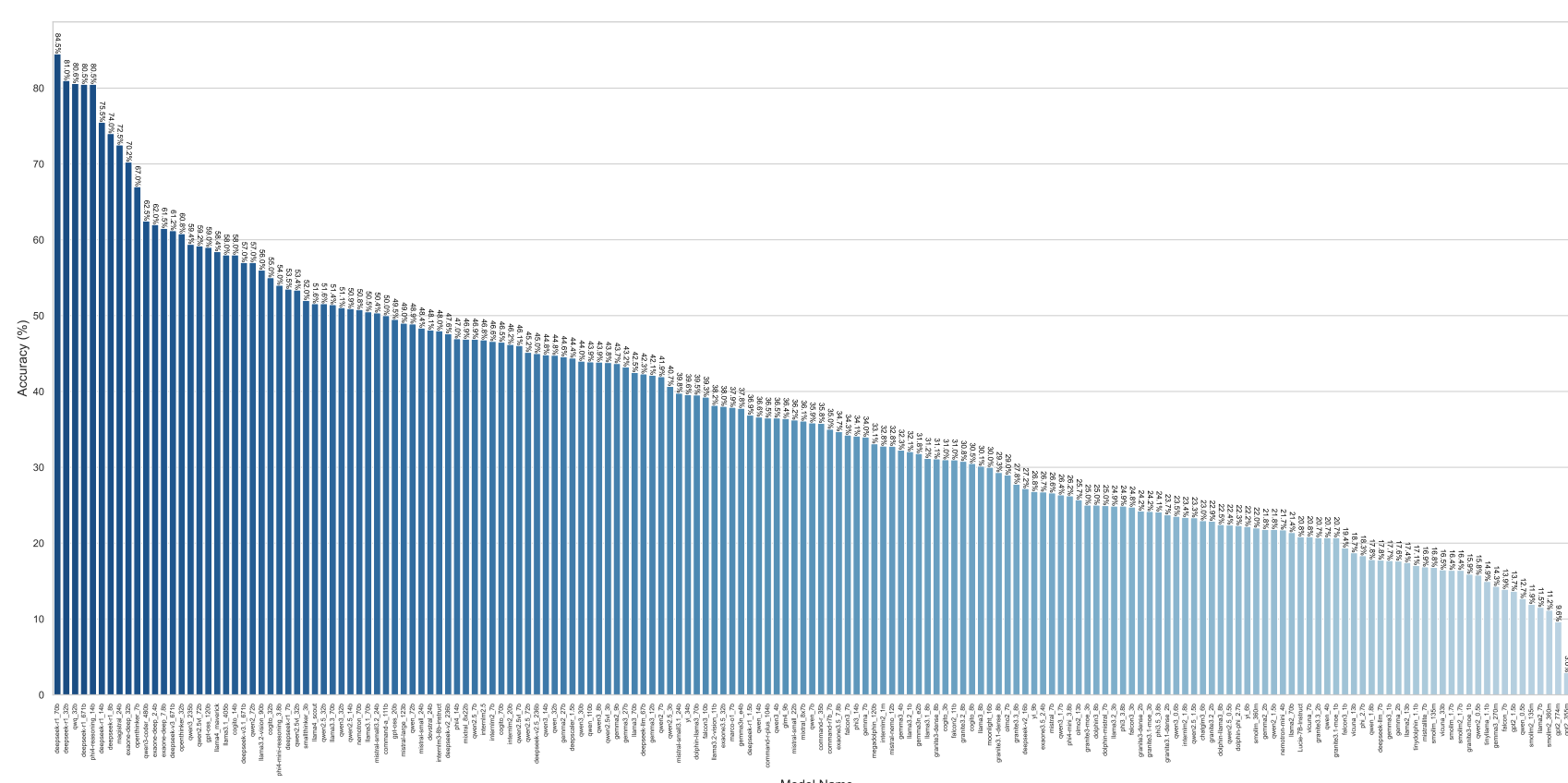

Figure S62: Bar chart of model accuracy rankings on the Associate Chief Physician Rehabilitation Medicine examination, evaluating knowledge of physical therapy, occupational therapy, stroke rehabilitation, and functional restoration techniques. Top performers: DeepSeek-R1-70b (84.5%), DeepSeek-R1-32b (81.0%), QWQ-32b (80.6%).

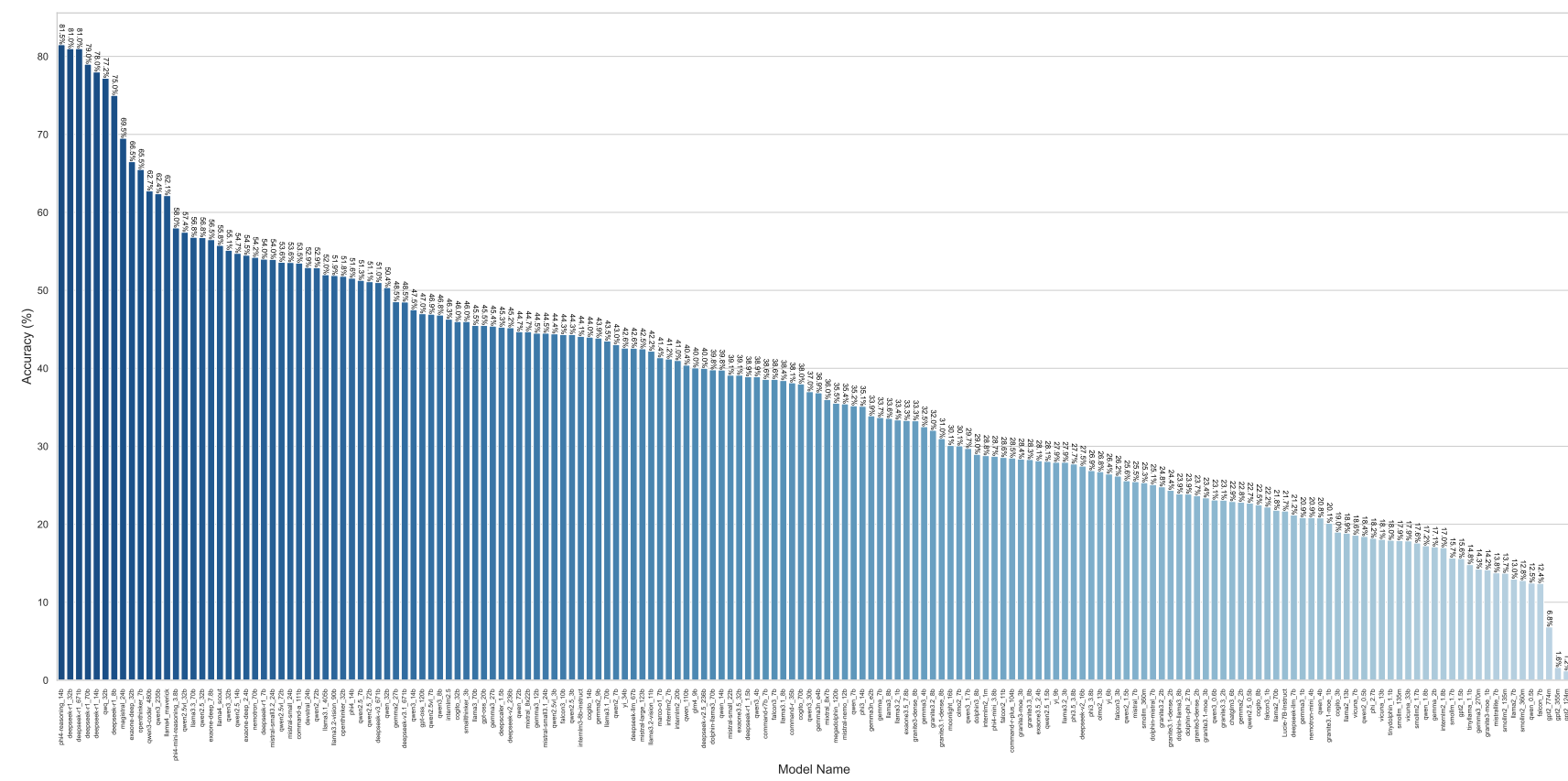

Figure S63: Bar chart of model accuracy rankings on the Associate Chief Physician Cardiothoracic Surgery examination, testing expertise in cardiac surgical procedures, thoracic surgery, congenital heart disease repair, and perioperative cardiac care. Top performers: Phi4-Reasoning-14b (81.5%), DeepSeek-R1-32b (81.0%), DeepSeek-R1-671b (81.0%).

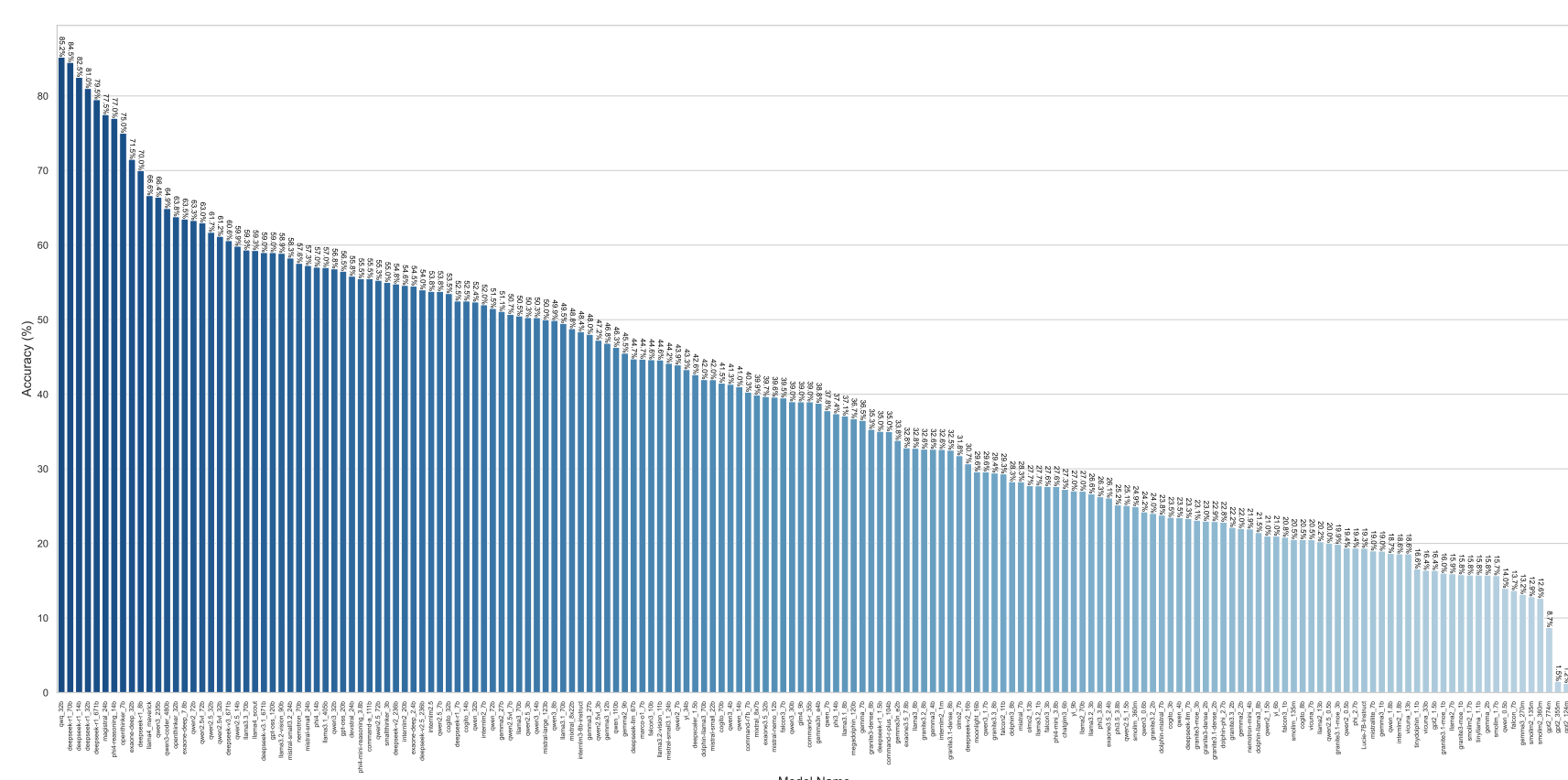

Figure S64: Bar chart of model accuracy rankings on the Associate Chief Physician Cardiology examination, assessing advanced knowledge of cardiac physiology, coronary artery disease, heart failure, arrhythmias, and interventional cardiology. Top performers: QWQ-32b (85.2%), DeepSeek-R1-70b (84.5%), DeepSeek-R1-14b (82.5%).

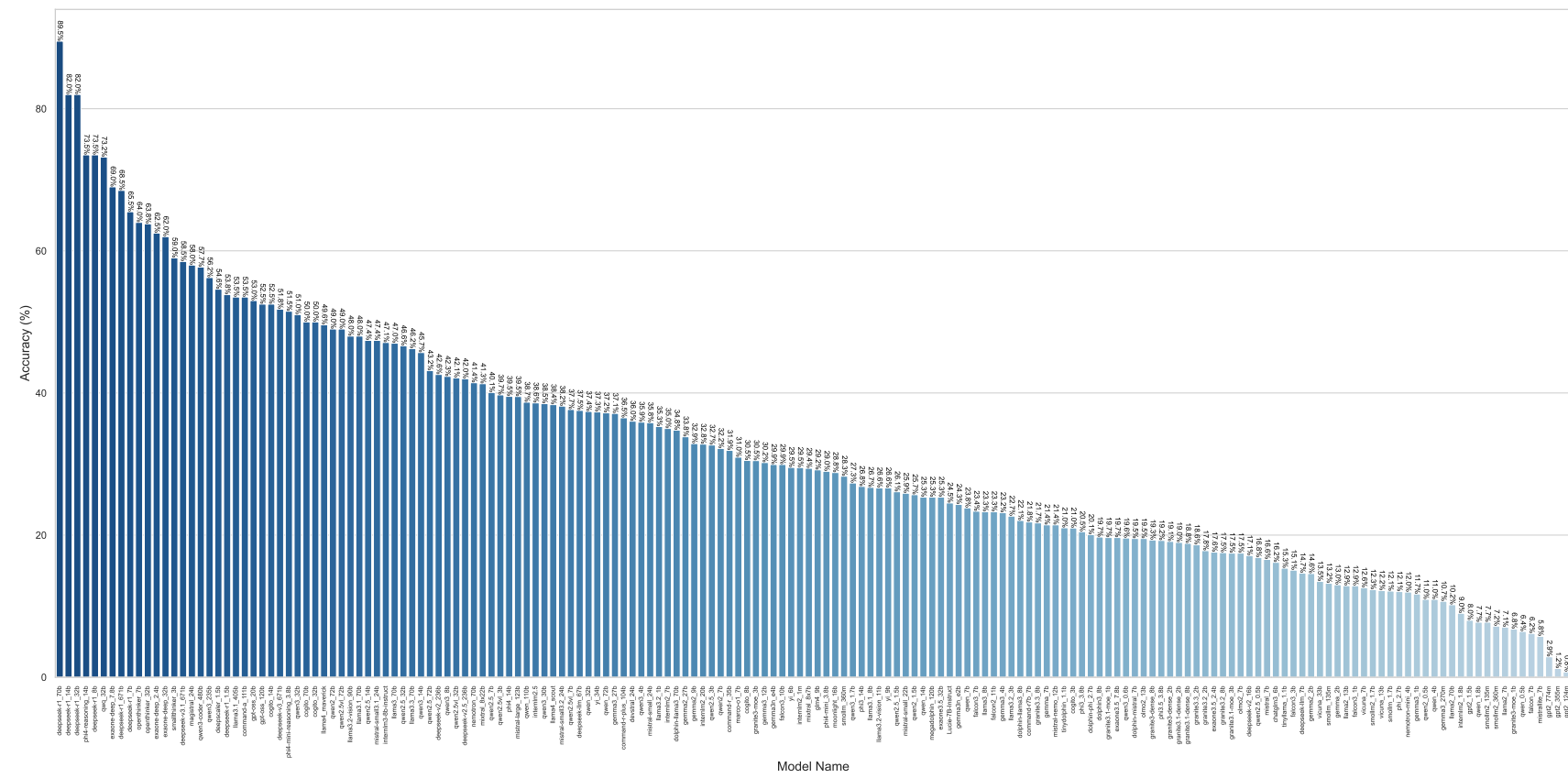

Figure S65: Bar chart of model accuracy rankings on the Associate Chief Physician Emergency Medicine examination, evaluating senior-level expertise in critical care, trauma management, and complex acute presentations requiring rapid decision-making. Top performers: DeepSeek-R1-70b (89.5%), DeepSeek-R1-14b (82.0%), DeepSeek-R1-32b (82.0%).

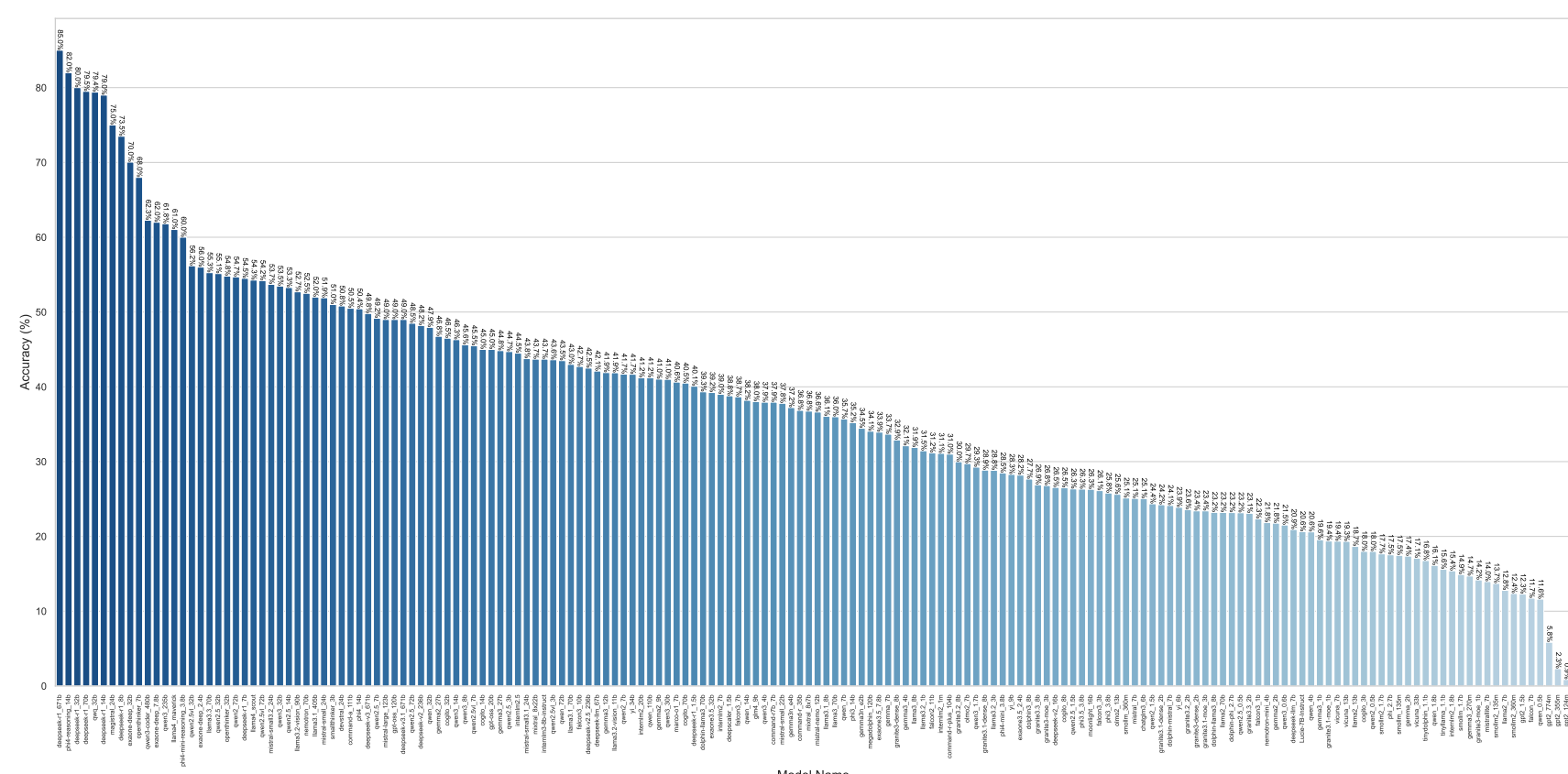

Figure S66: Bar chart of model accuracy rankings on the Associate Chief Physician Plastic Surgery examination, testing knowledge of reconstructive surgery, aesthetic procedures, microsurgery, and burn reconstruction. Top performers: DeepSeek-R1-671b (85.0%), Phi4-Reasoning-14b (82.0%), DeepSeek-R1-32b (80.0%).

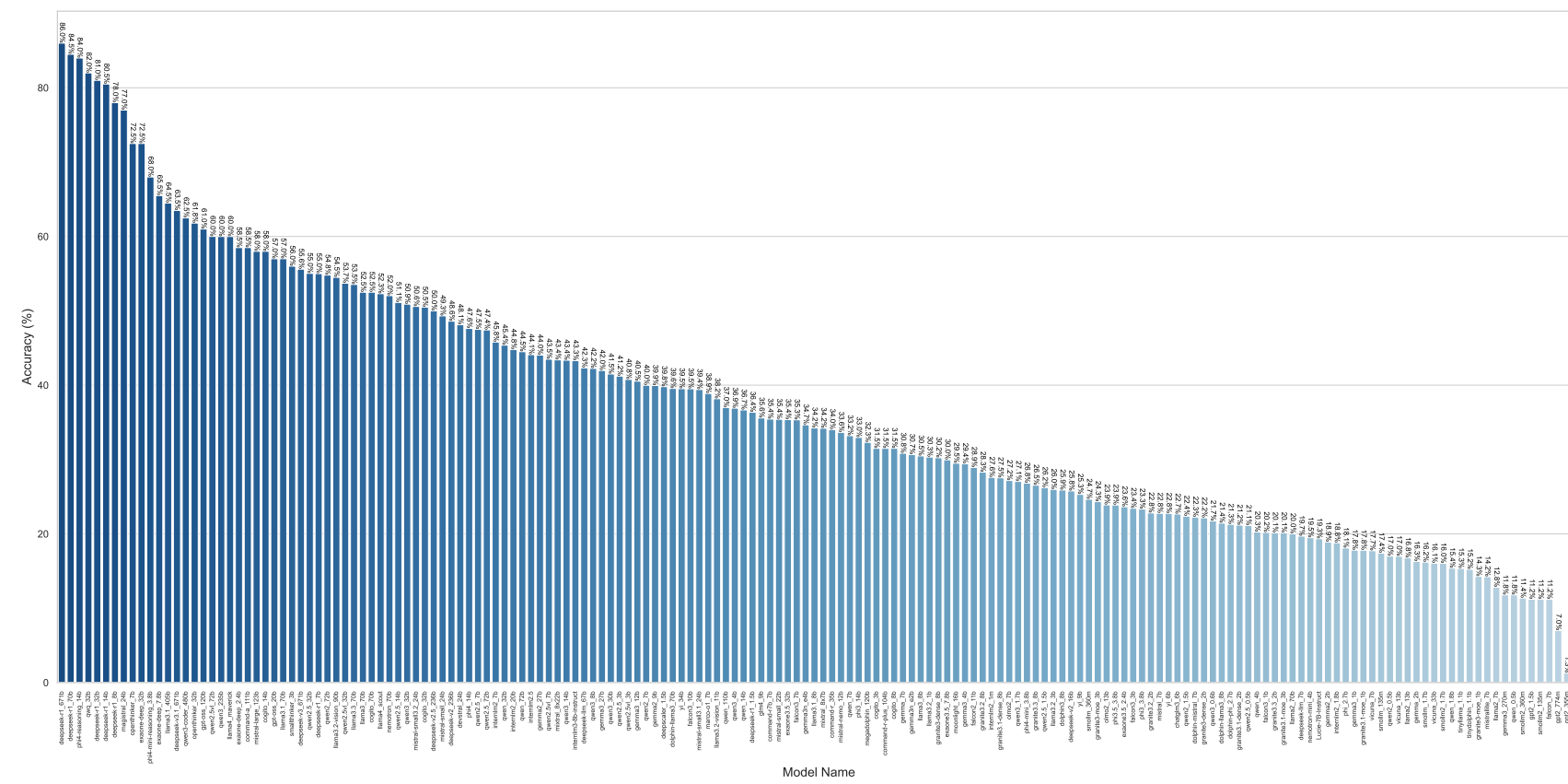

Figure S67: Bar chart of model accuracy rankings on the Associate Chief Physician General Surgery examination, assessing expertise in abdominal surgery, surgical oncology, trauma surgery, and minimally invasive surgical techniques. Top performers: DeepSeek-R1-671b (86.0%), DeepSeek-R1-70b (84.5%), Phi4-Reasoning-14b (84.0%).

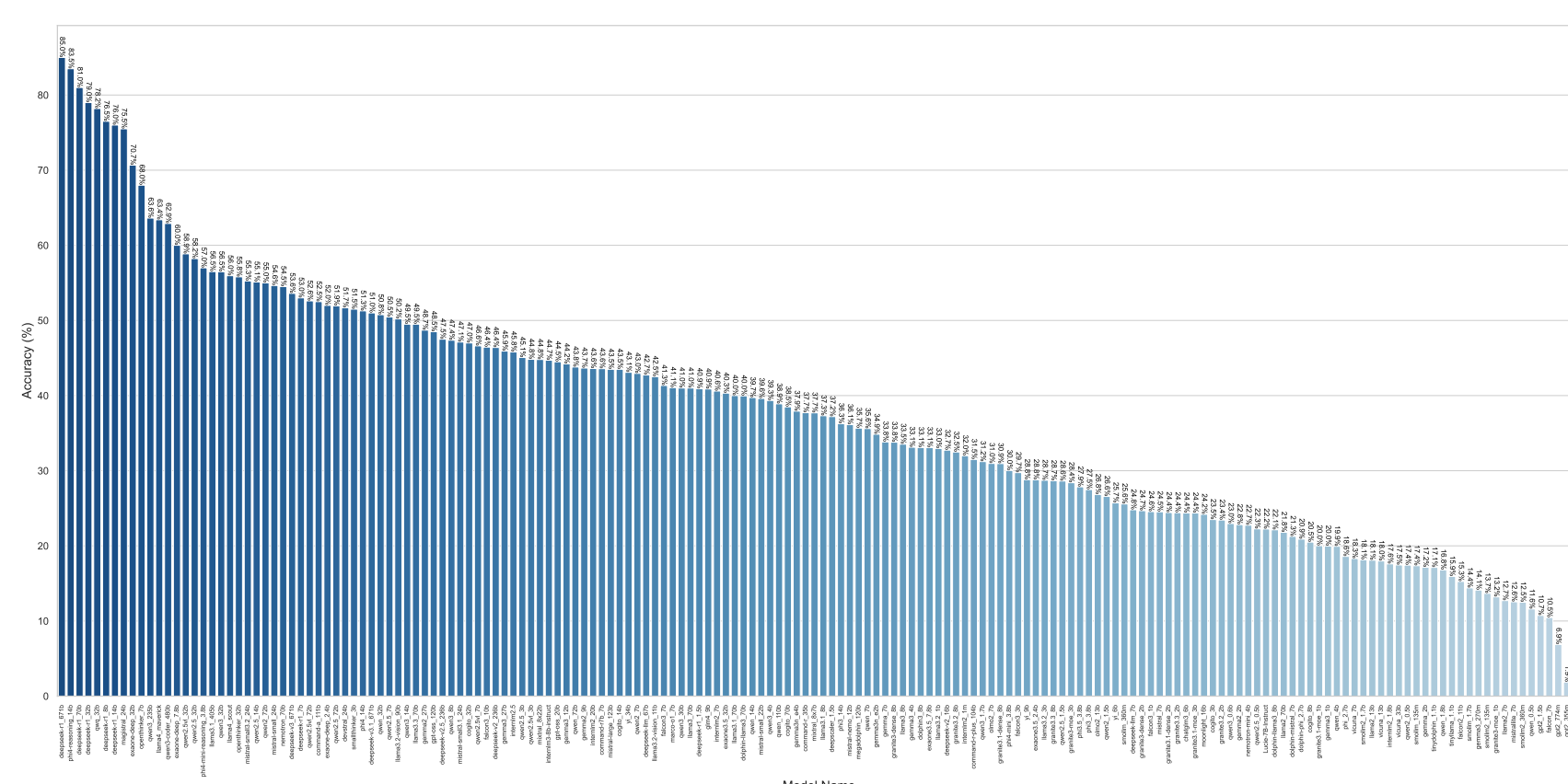

Figure S68: Bar chart of model accuracy rankings on the Associate Chief Physician Urology examination, evaluating knowledge of urinary tract disorders, prostate diseases, urological oncology, and reconstructive urology. Top performers: DeepSeek-R1-671b (85.0%), Phi4-Reasoning-14b (83.5%), DeepSeek-R1-70b (81.0%).

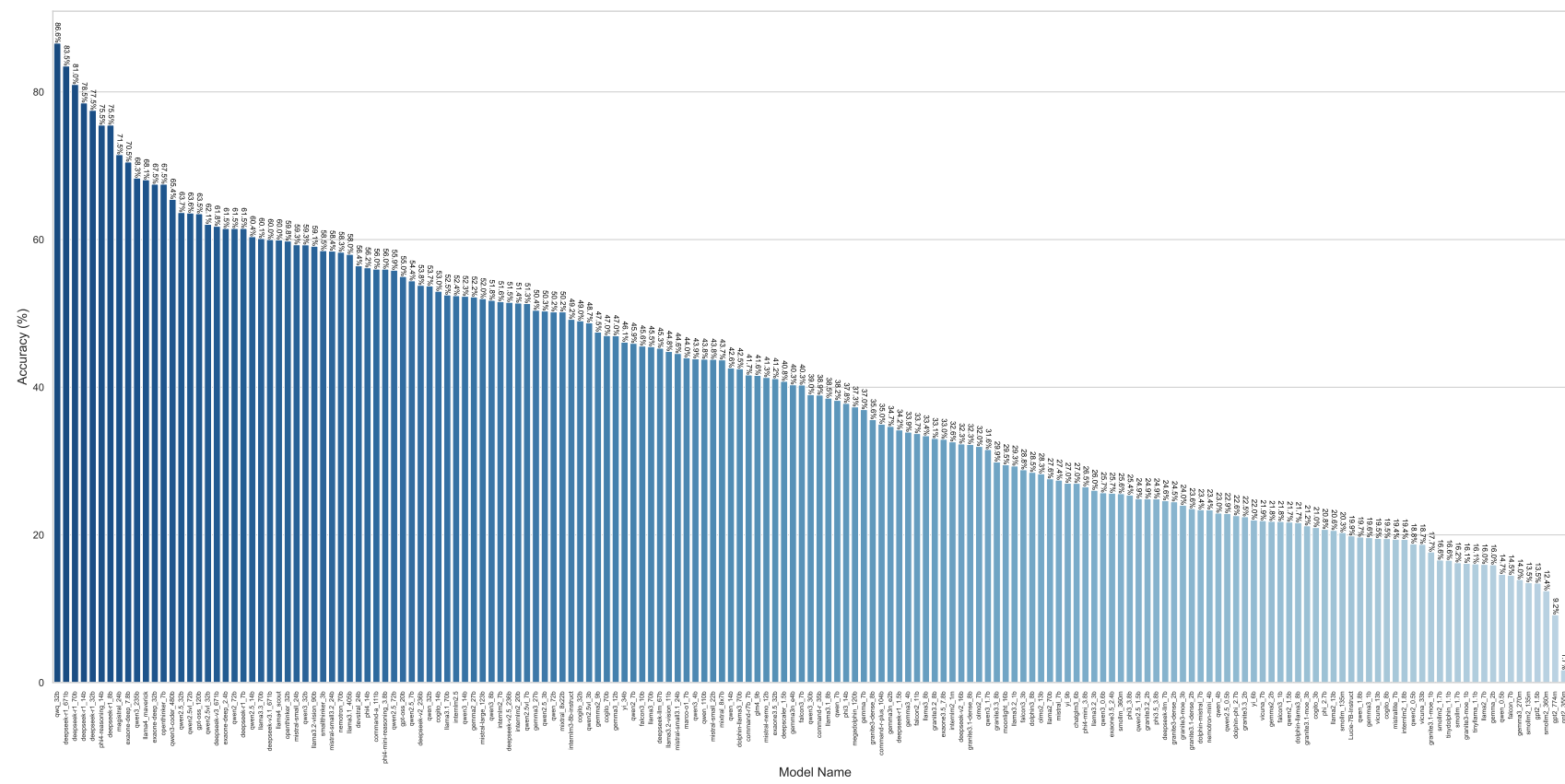

Figure S69: Bar chart of model accuracy rankings on the Associate Chief Physician Gastroenterology examination, assessing expertise in digestive tract diseases, hepatobiliary disorders, endoscopic procedures, and inflammatory bowel disease management. Top performers: QWQ-32b (86.6%), DeepSeek-R1-671b (83.5%), DeepSeek-R1-70b (81.0%).

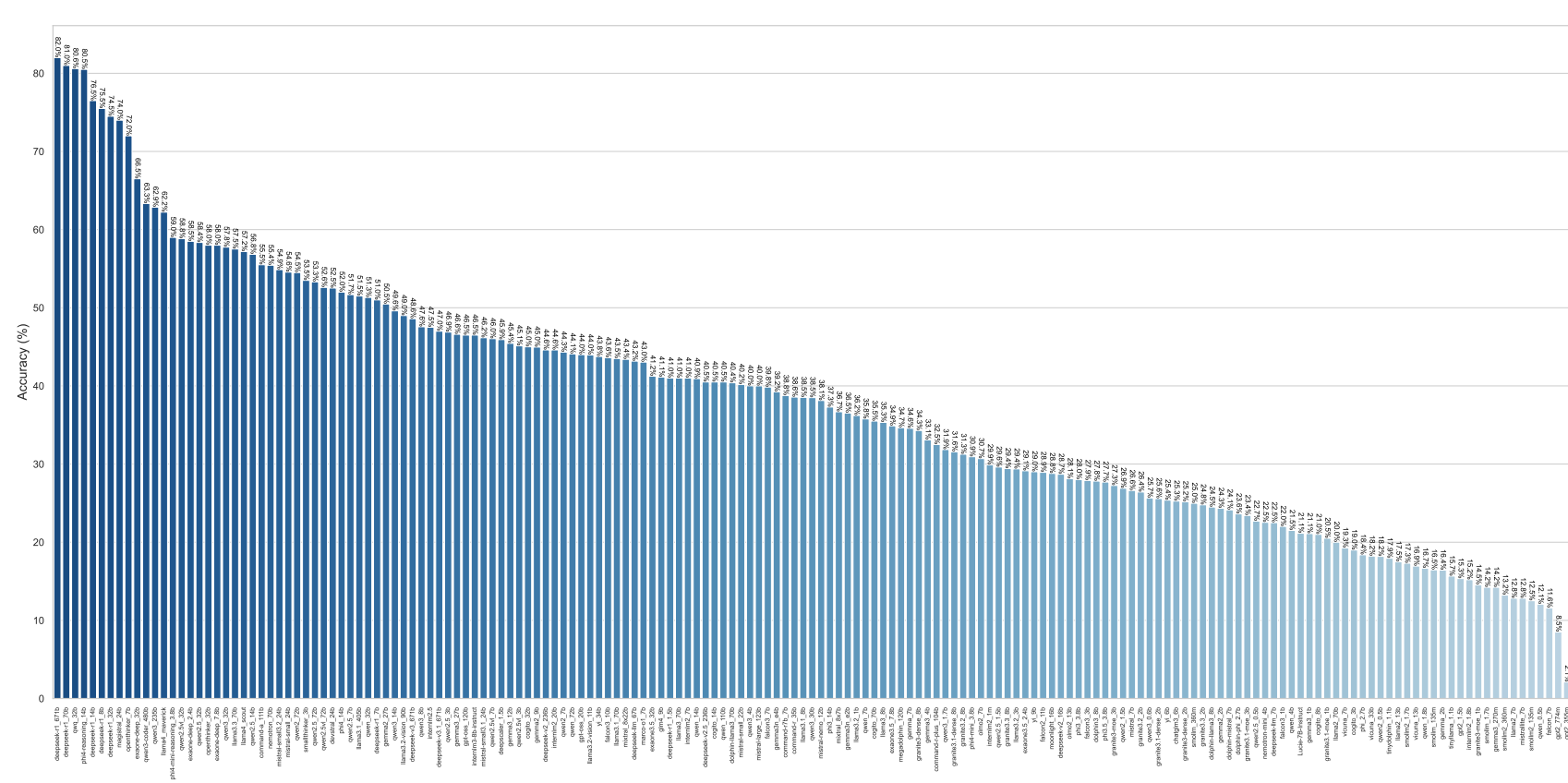

Figure S70: Bar chart of model accuracy rankings on the Associate Chief Physician Burn Surgery examination, testing expertise in burn wound management, fluid resuscitation, skin grafting, and burn rehabilitation. Top performers: DeepSeek-R1-671b (82.0%), DeepSeek-R1-70b (81.0%), QWQ-32b (80.6%).

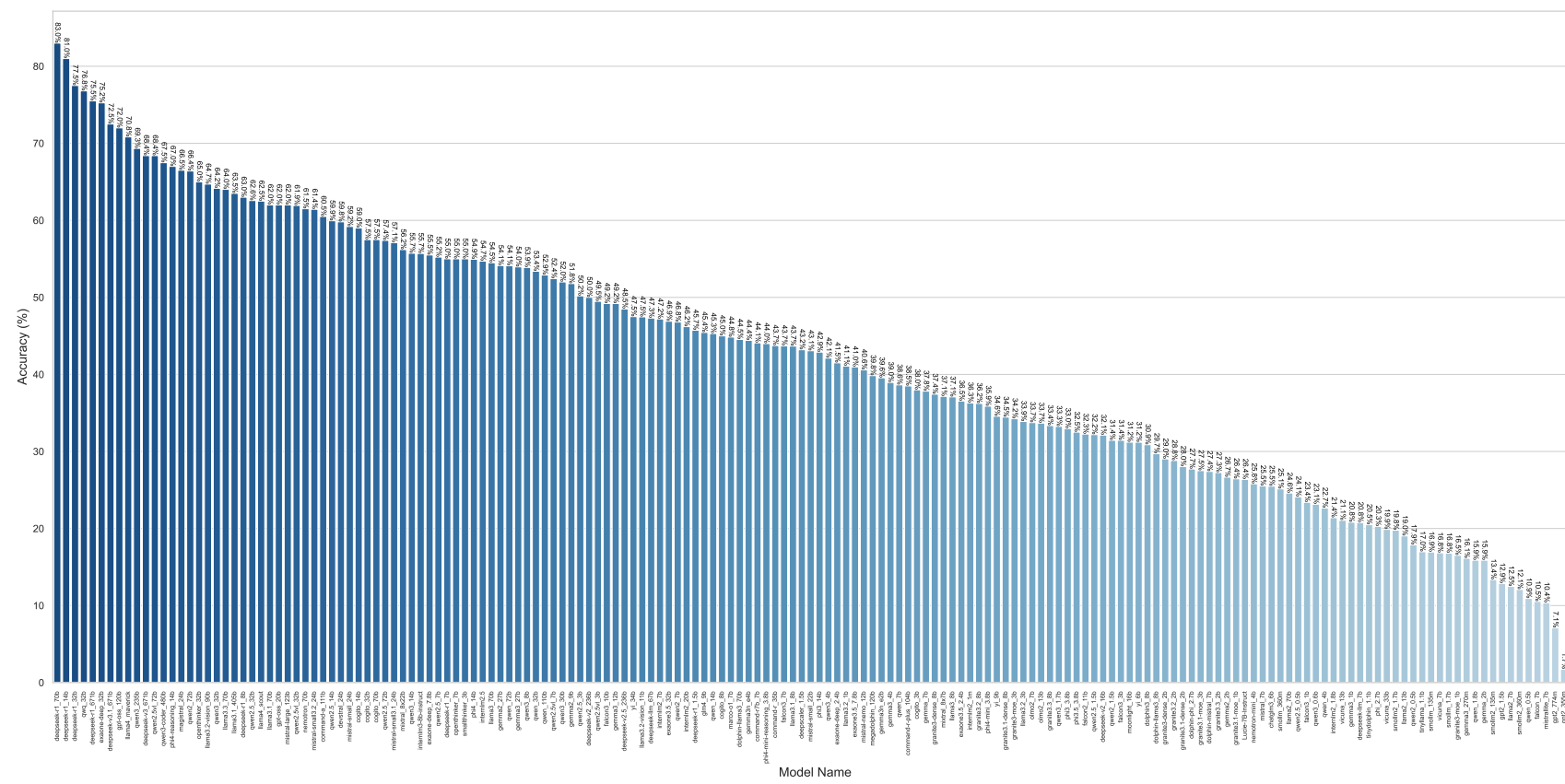

Figure S71: Bar chart of model accuracy rankings on the Associate Chief Physician Pain Medicine examination, evaluating knowledge of pain pathophysiology, chronic pain management, interventional pain procedures, and multimodal analgesia. Top performers: DeepSeek-R1-70b (83.0%), DeepSeek-R1-14b (81.0%), DeepSeek-R1-32b (77.5%).

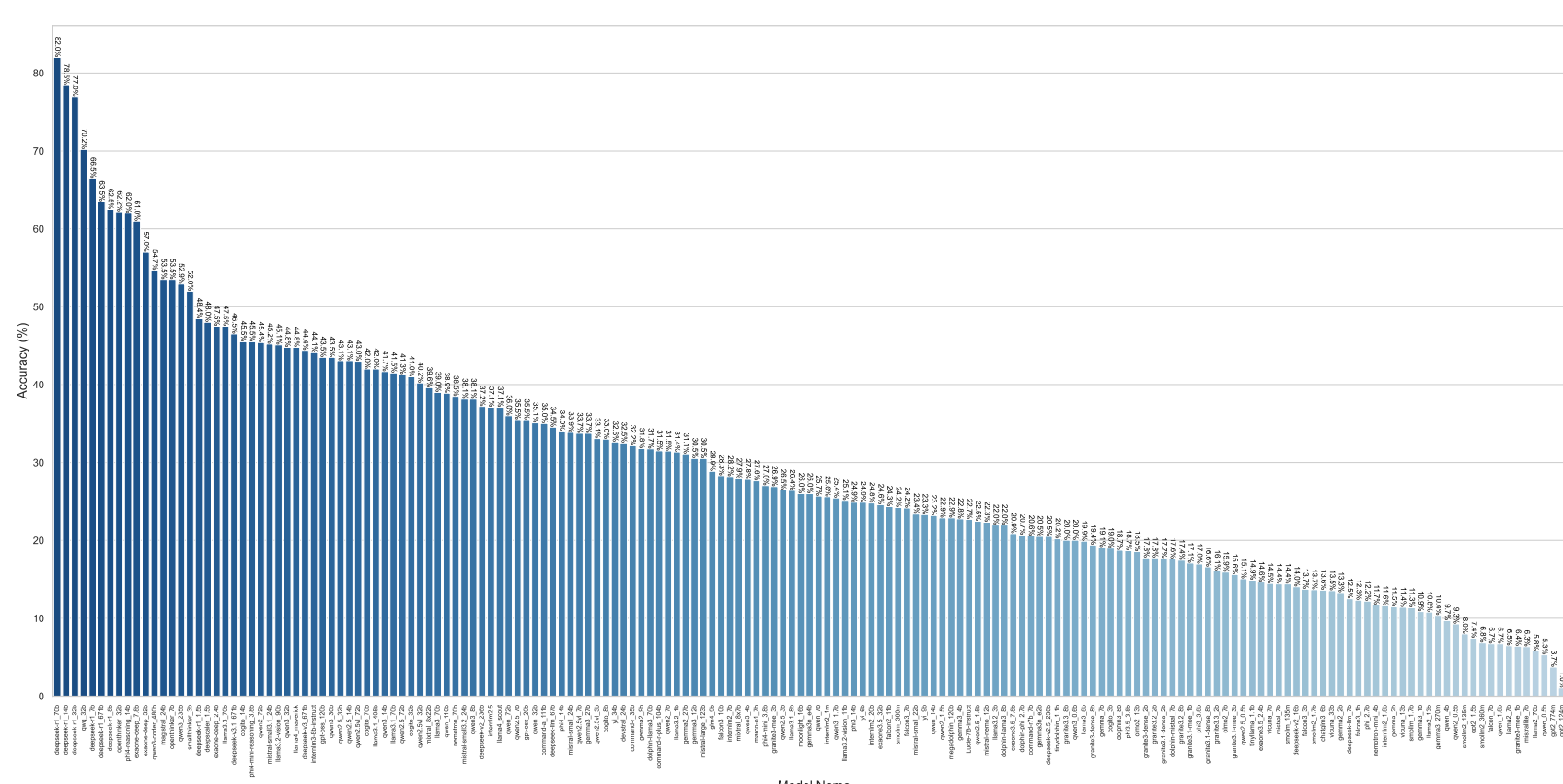

Figure S72: Bar chart of model accuracy rankings on the Associate Chief Physician Dermatology and Venereology examination, assessing senior-level expertise in complex dermatologic conditions and sexually transmitted disease management. Top performers: DeepSeek-R1-70b (82.0%), DeepSeek-R1-14b (78.5%), DeepSeek-R1-32b (77.0%).

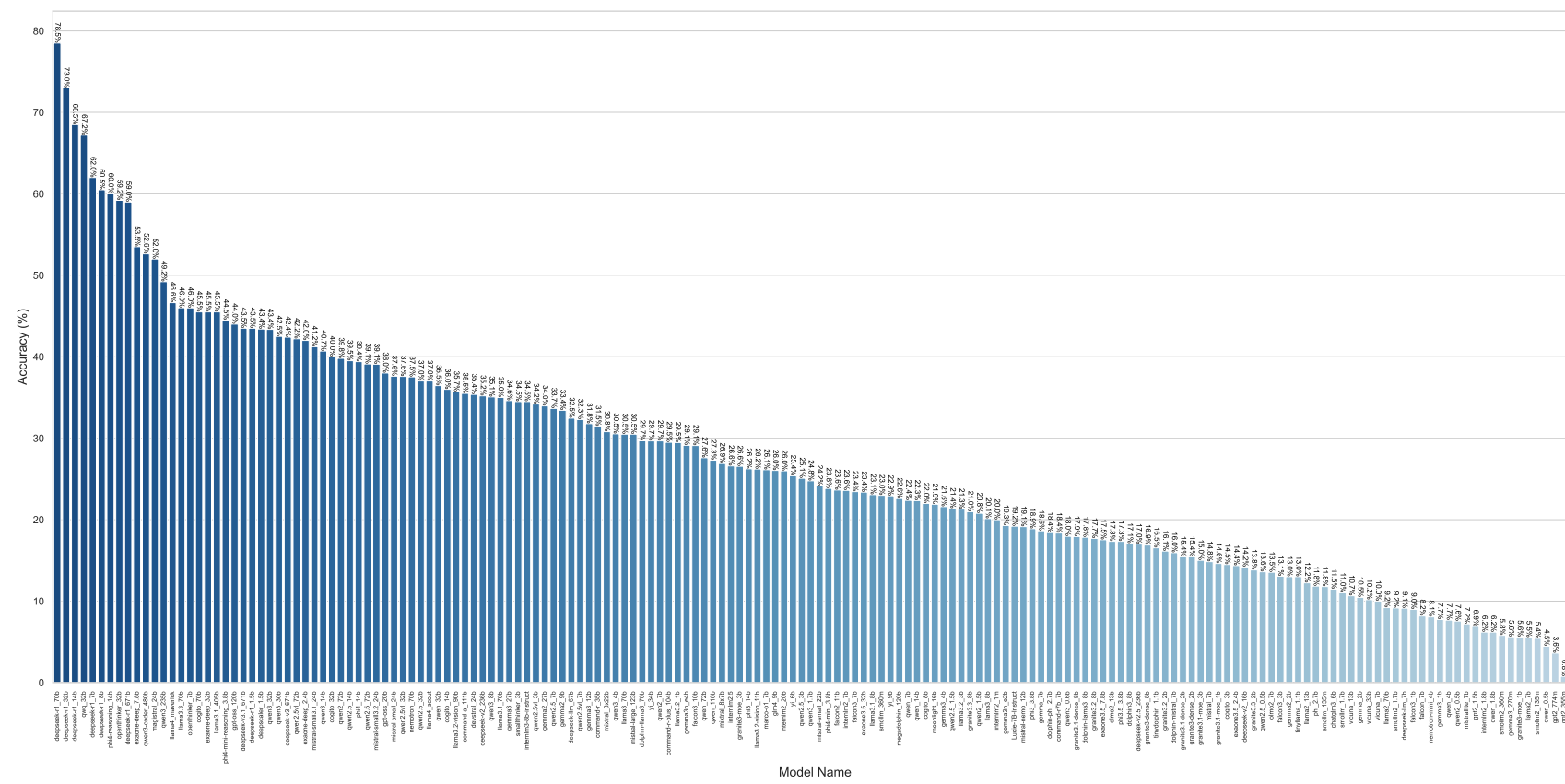

Figure S73: Bar chart of model accuracy rankings on the Associate Chief Physician Neurology examination, testing advanced knowledge of neurological disorders, stroke intervention, and complex neurodegenerative disease management. Top performers: DeepSeek-R1-70b (78.5%), DeepSeek-R1-32b (73.0%), DeepSeek-R1-14b (68.5%).

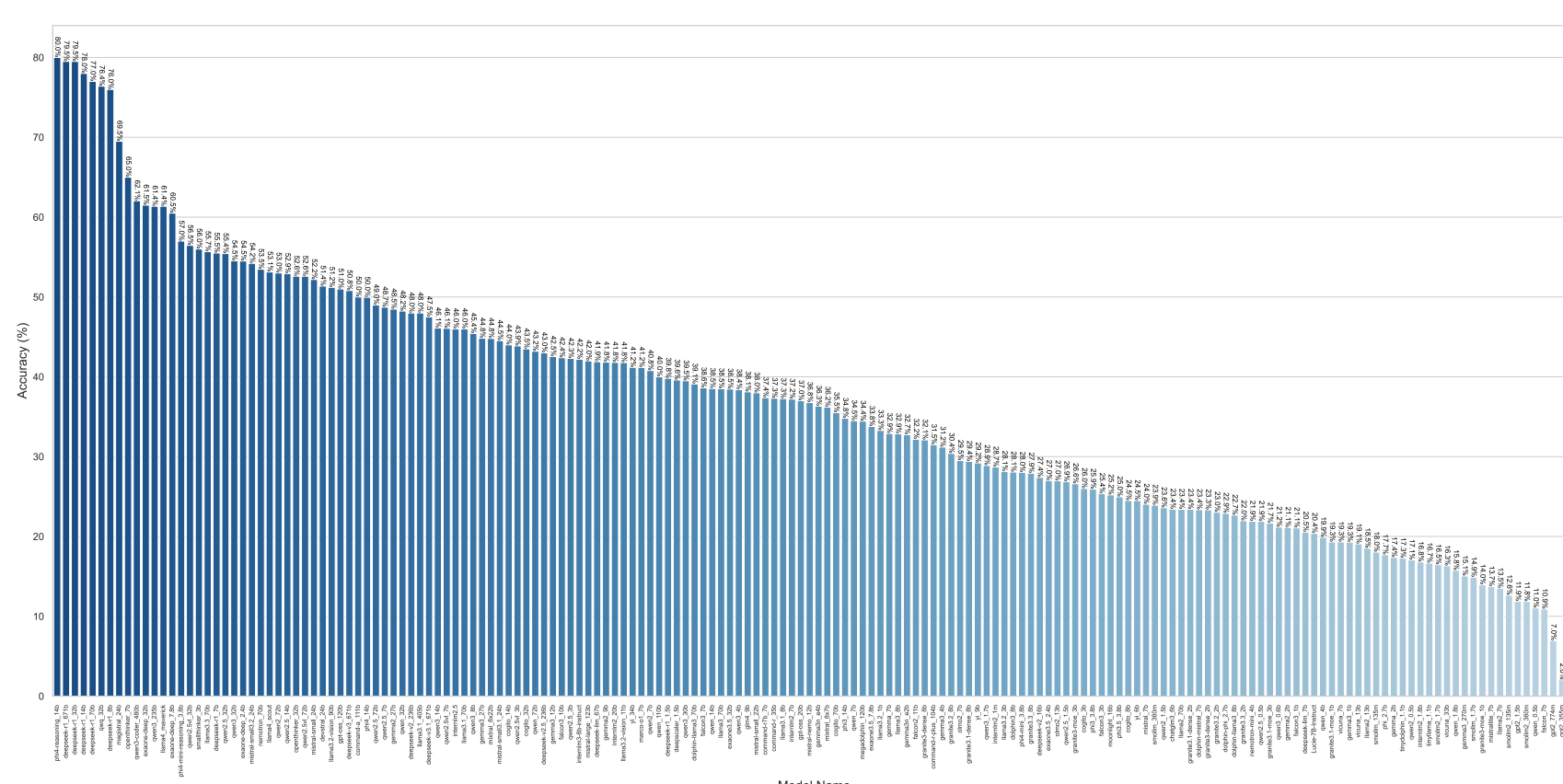

Figure S74: Bar chart of model accuracy rankings on the Associate Chief Physician Neurosurgery examination, assessing expertise in brain and spine surgery, neurovascular procedures, and surgical management of CNS tumors. Top performers: Phi4-Reasoning-14b (80.0%), DeepSeek-R1-671b (79.5%), DeepSeek-R1-32b (79.5%).

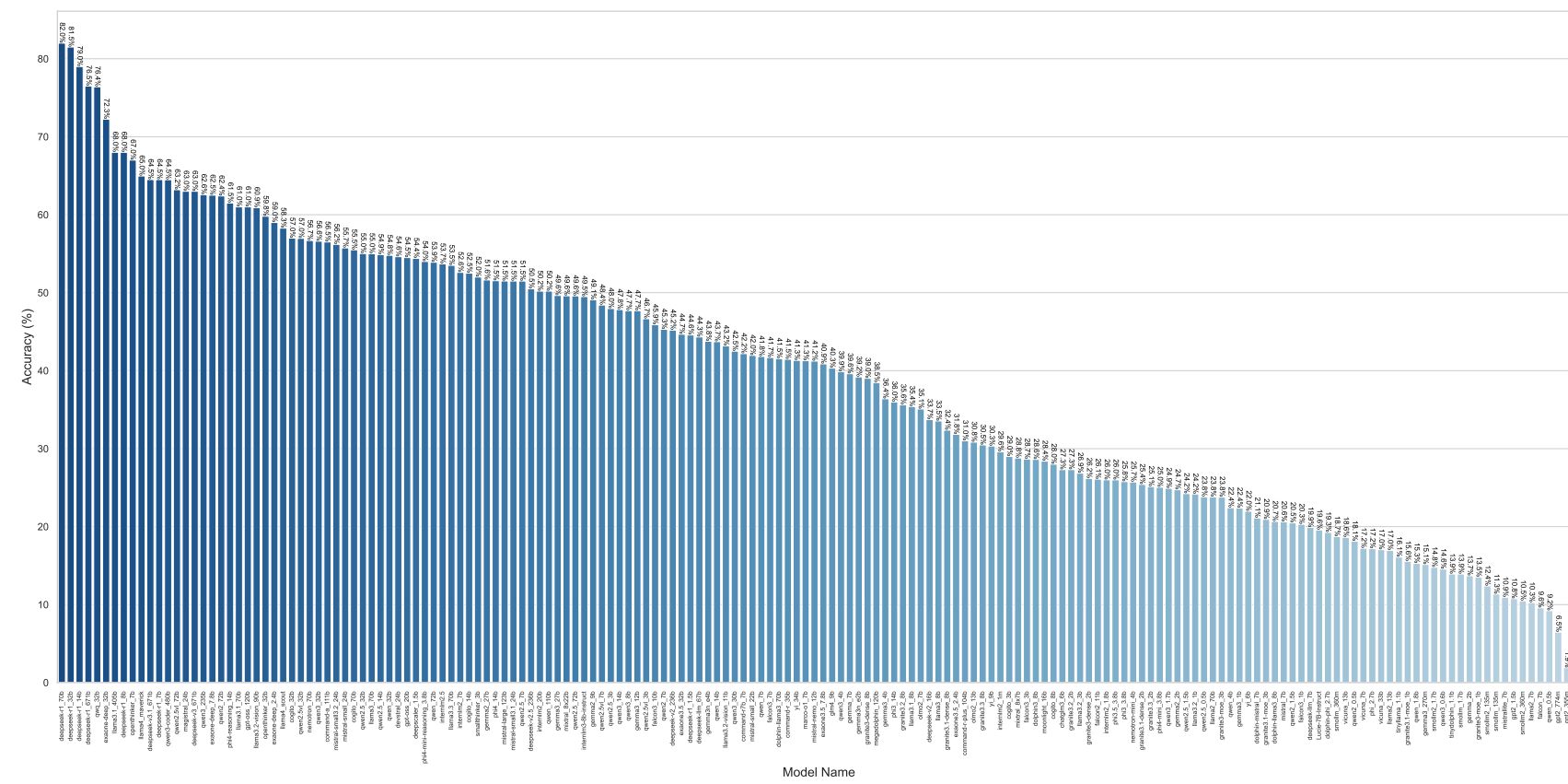

Figure S75: Bar chart of model accuracy rankings on the Associate Chief Physician Psychiatry examination, evaluating advanced knowledge of psychiatric disorders, psychopharmacology, and evidence-based psychotherapeutic interventions. Top performers: DeepSeek-R1-70b (82.0%), DeepSeek-R1-32b (81.5%), DeepSeek-R1-14b (79.0%).

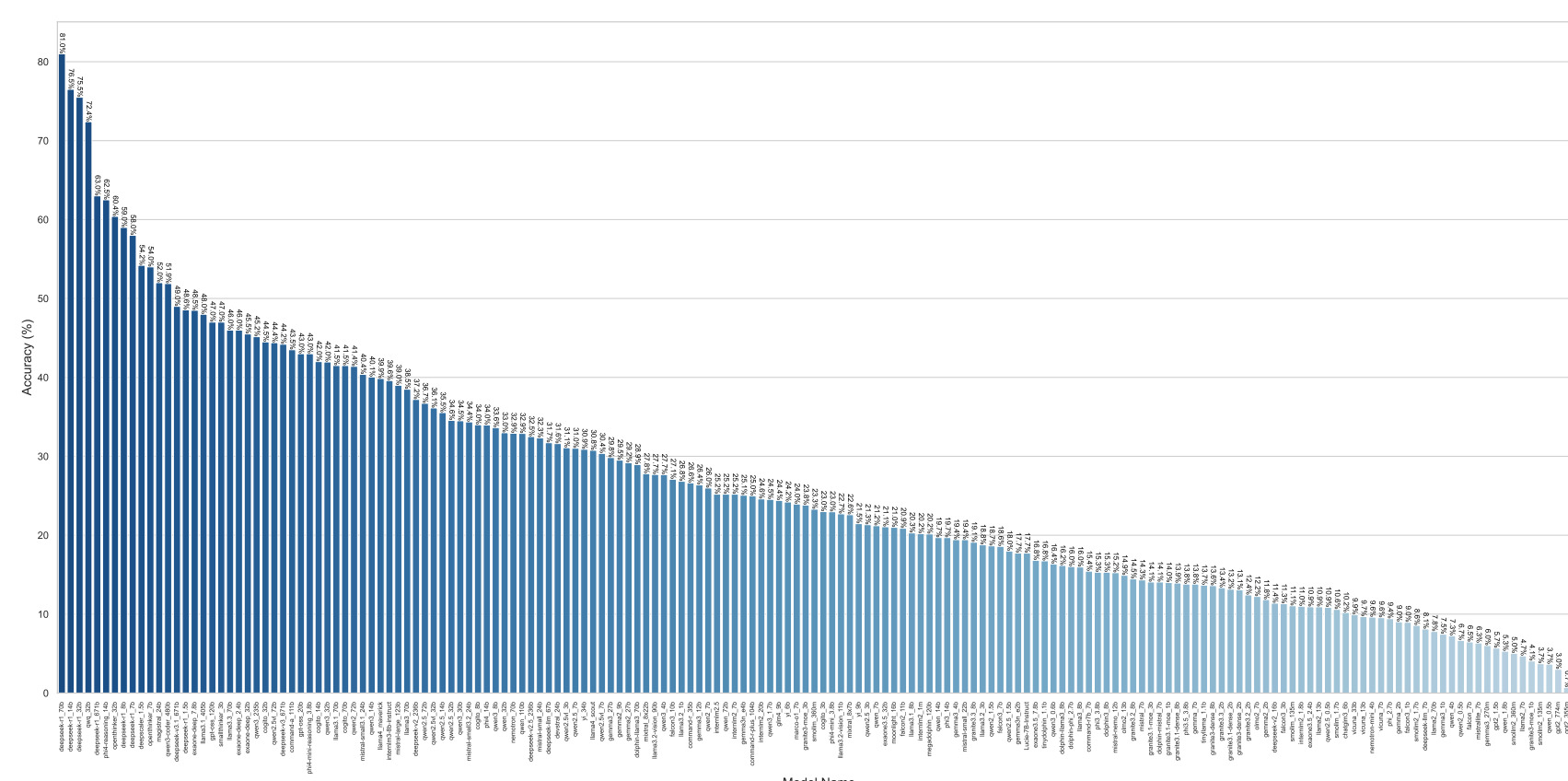

Figure S76: Bar chart of model accuracy rankings on the Associate Chief Physician Otolaryngology-Head and Neck Surgery examination, testing expertise in head-neck oncology, complex ENT surgery, and reconstructive procedures. Top performers: DeepSeek-R1-70b (81.0%), DeepSeek-R1-14b (76.5%), DeepSeek-R1-32b (75.5%).

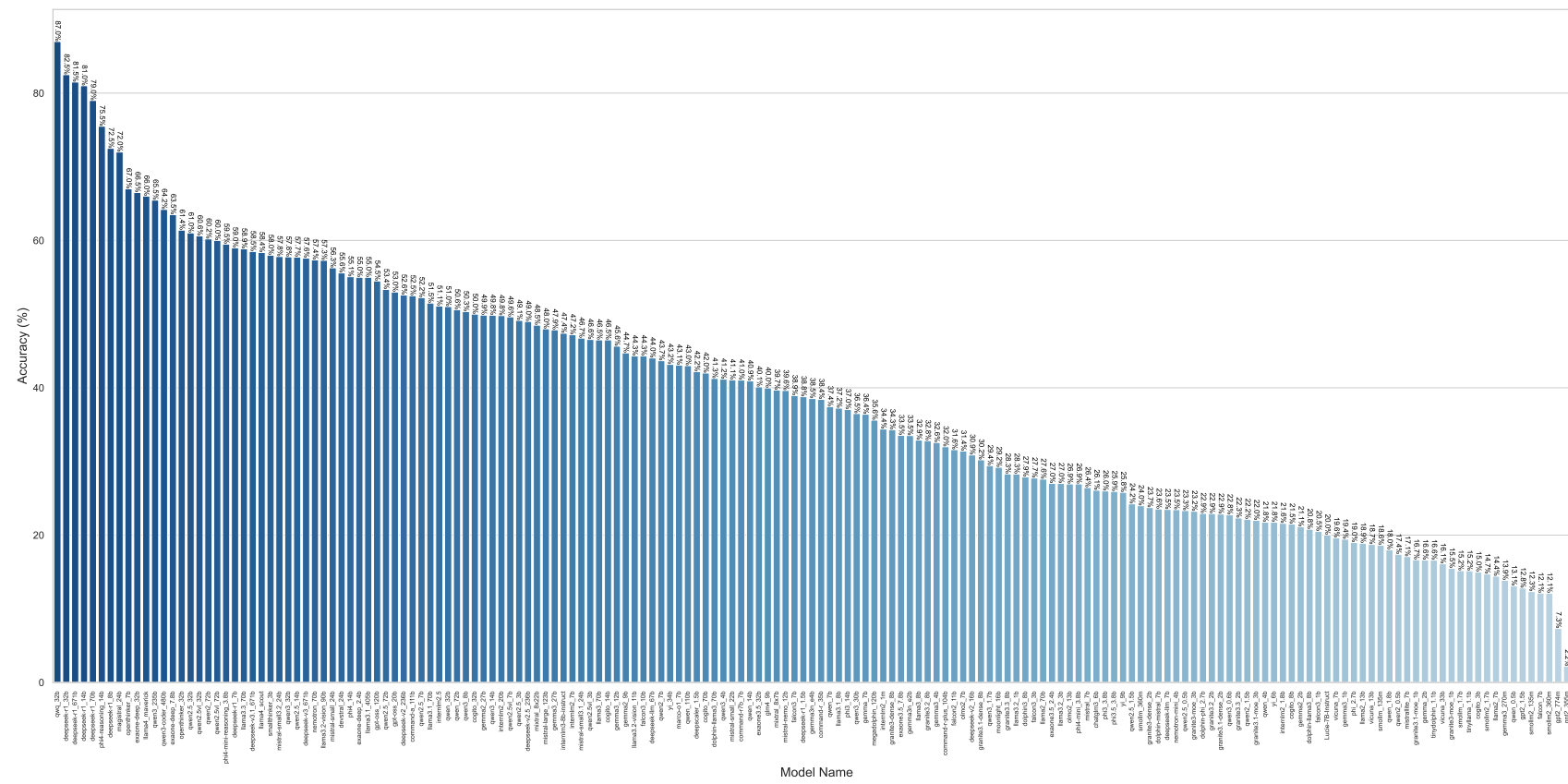

Figure S77: Bar chart of model accuracy rankings on the Associate Chief Physician Occupational Medicine examination, assessing knowledge of occupational diseases, workplace hazards, and occupational health surveillance. Top performers: QWQ-32b (87.0%), DeepSeek-R1-32b (82.5%), DeepSeek-R1-671b (81.5%).

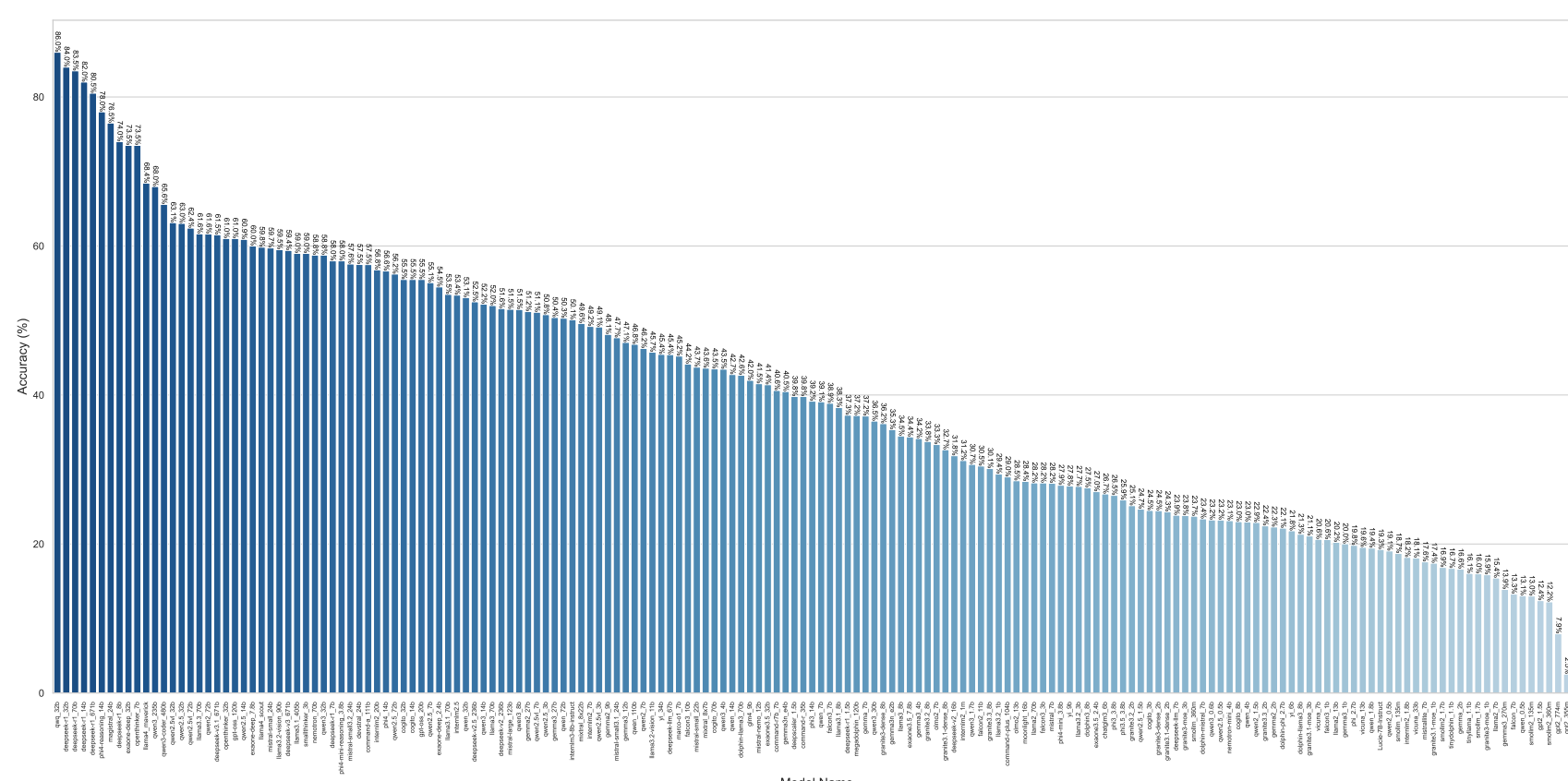

Figure S78: Bar chart of model accuracy rankings on the Associate Chief Physician Nephrology examination, evaluating expertise in kidney diseases, dialysis management, kidney transplantation, and electrolyte disorders. Top performers: QWQ-32b (86.0%), DeepSeek-R1-32b (84.0%), DeepSeek-R1-70b (83.5%).

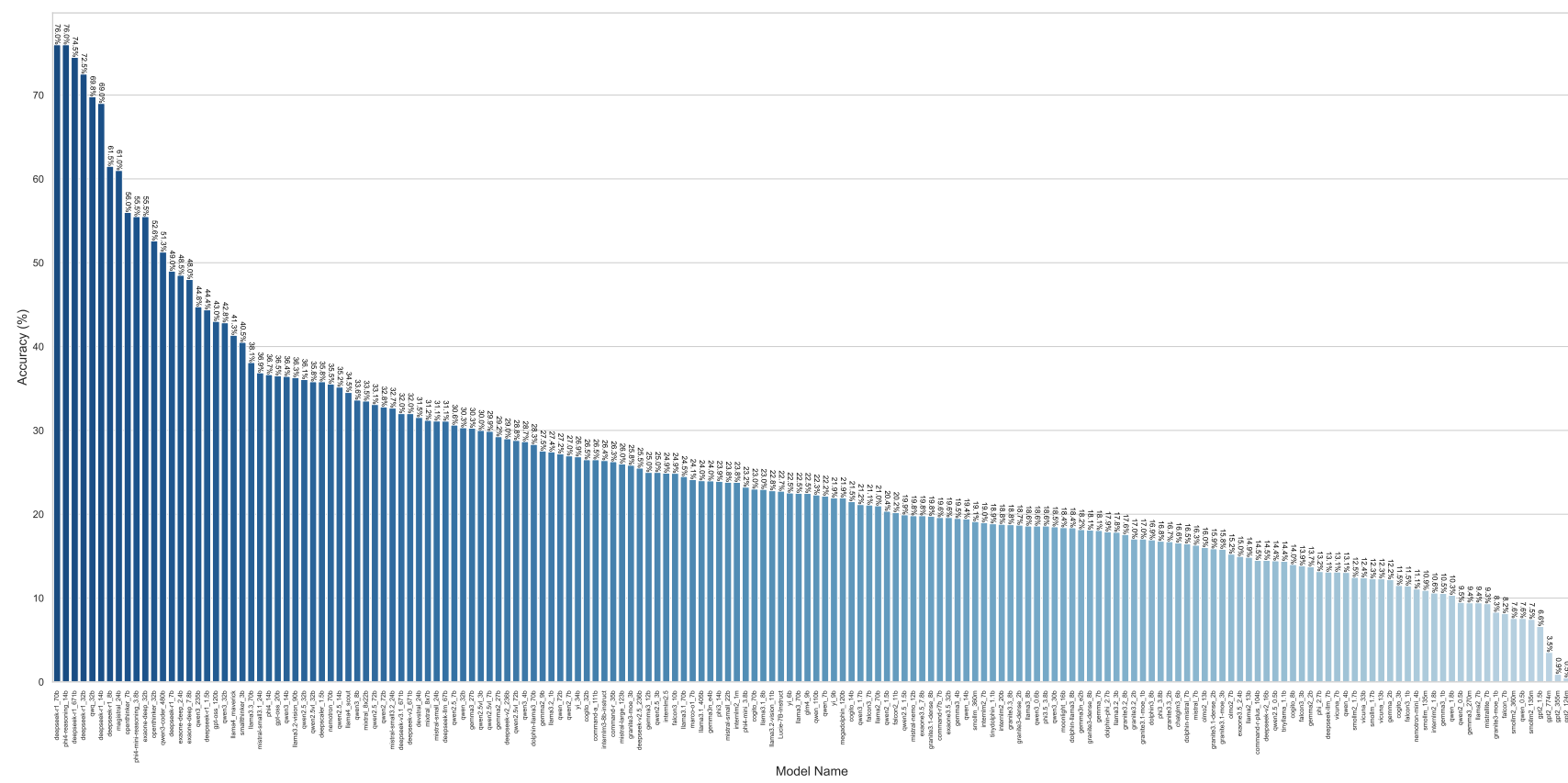

Figure S79: Bar chart of model accuracy rankings on the Associate Chief Physician Medical Oncology examination, testing advanced knowledge of cancer treatment protocols, targeted therapies, and oncologic emergencies. Top performers: DeepSeek-R1-70b (76.0%), Phi4-Reasoning-14b (76.0%), DeepSeek-R1-671b (74.5%).

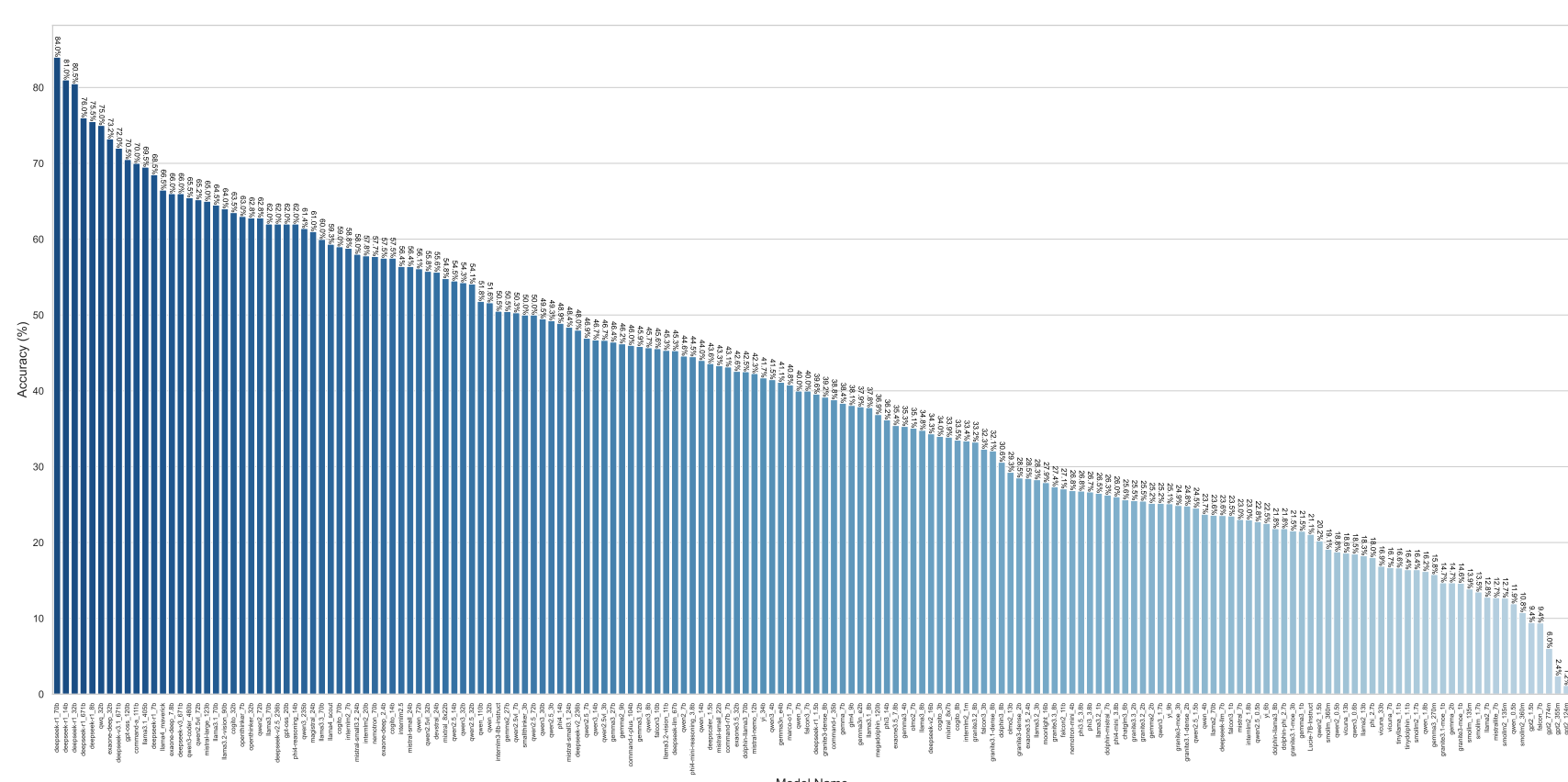

Figure S80: Bar chart of model accuracy rankings on the Associate Chief Physician Surgical Oncology examination, assessing expertise in surgical treatment of malignancies across organ systems. Top performers: DeepSeek-R1-70b (84.0%), DeepSeek-R1-14b (81.0%), DeepSeek-R1-32b (80.5%).

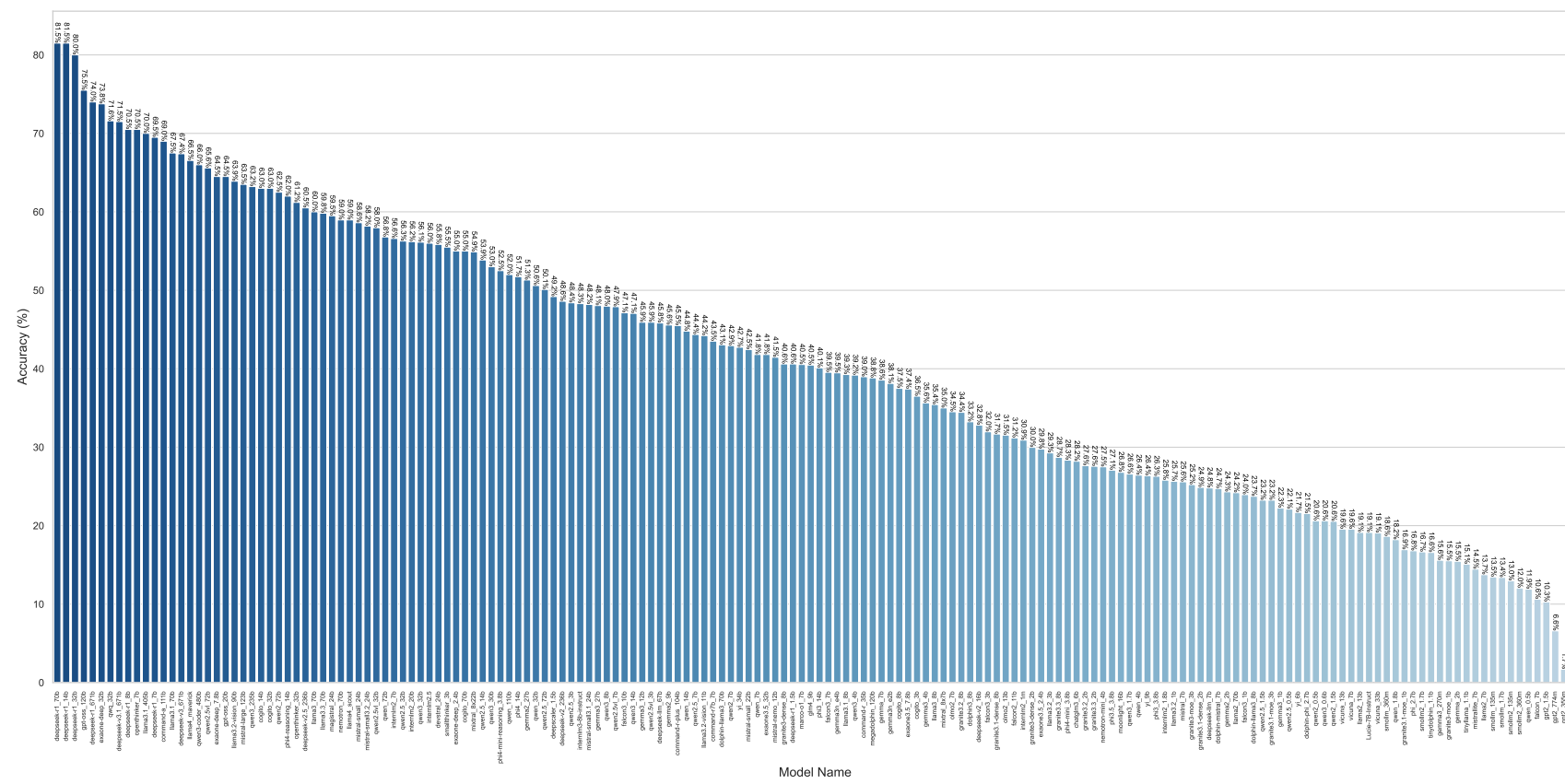

Figure S81: Bar chart of model accuracy rankings on the Associate Chief Physician Radiation Oncology examination, evaluating expertise in radiotherapy planning, brachytherapy, and management of radiation-related toxicities. Top performers: DeepSeek-R1-70b (81.5%), DeepSeek-R1-14b (81.5%), DeepSeek-R1-32b (80.0%).

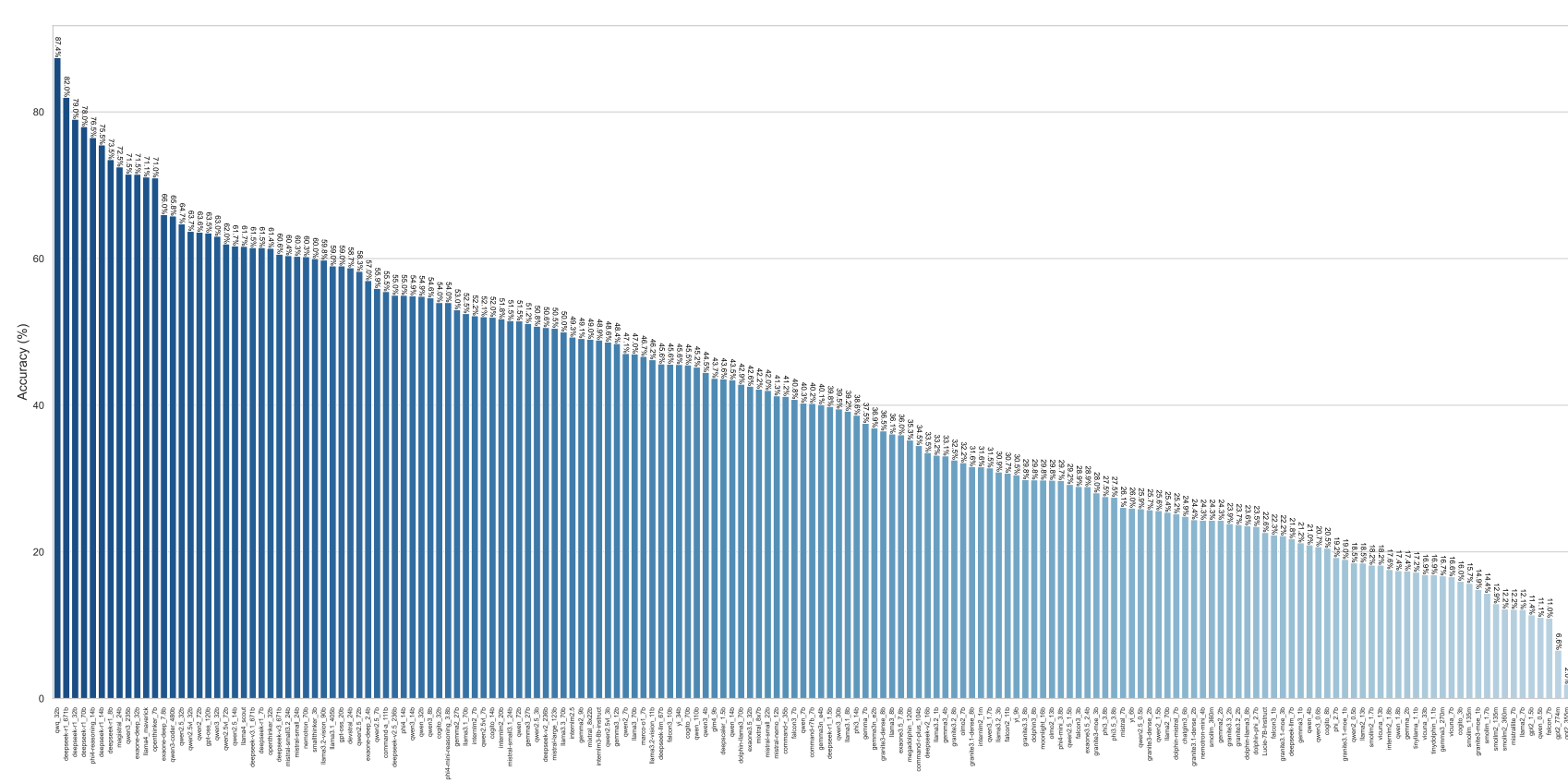

Figure S82: Bar chart of model accuracy rankings on the Associate Chief Physician Hematology examination, testing knowledge of blood disorders, coagulation abnormalities, hematologic malignancies, and transfusion medicine. Top performers: QWQ-32b (87.4%), DeepSeek-R1-671b (82.0%), DeepSeek-R1-32b (79.0%).

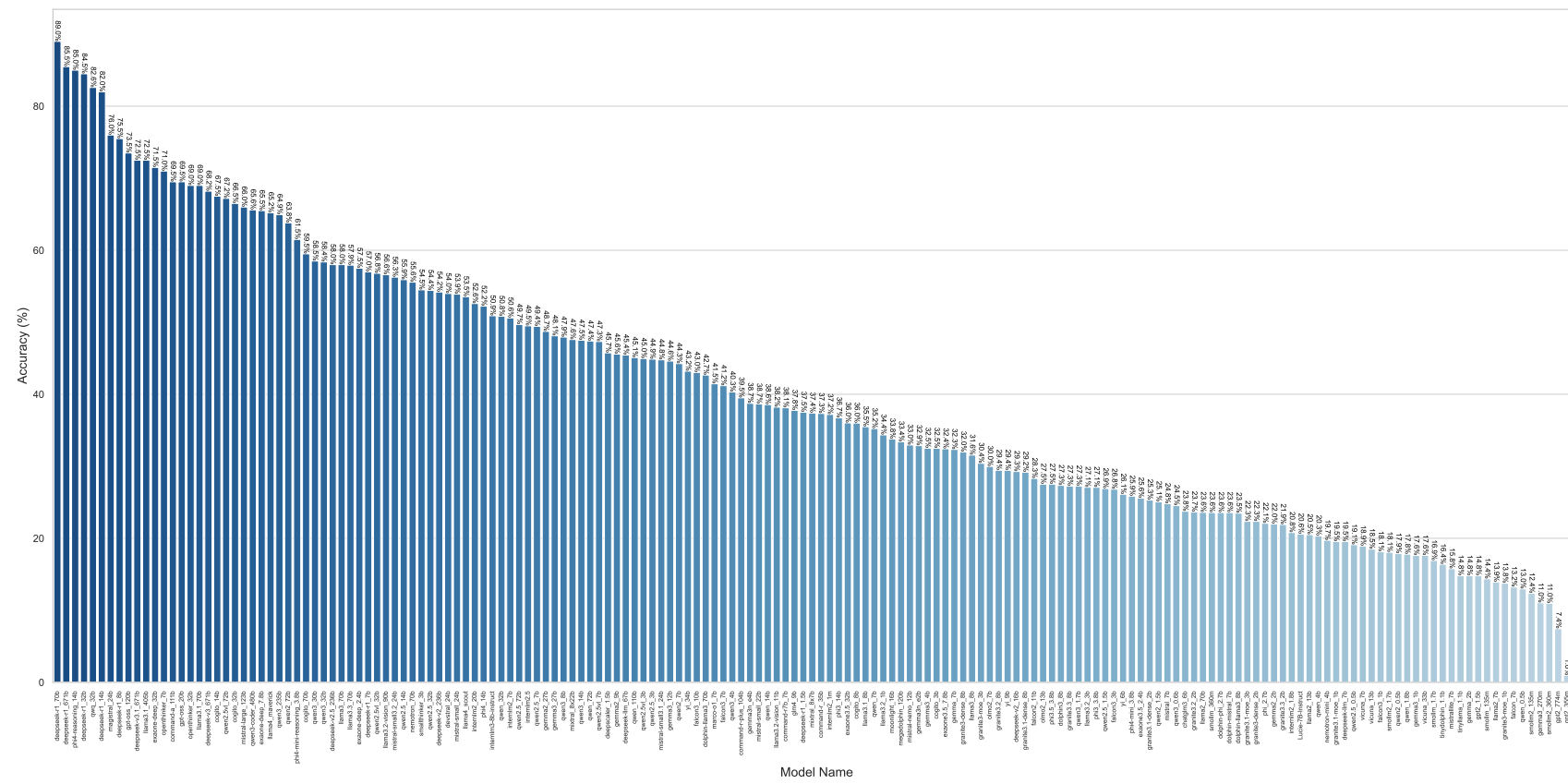

Figure S83: Bar chart of model accuracy rankings on the Associate Chief Physician Critical Care Medicine examination, assessing expertise in ICU management, mechanical ventilation, hemodynamic monitoring, and sepsis treatment. Top performers: DeepSeek-R1-70b (89.0%), DeepSeek-R1-671b (85.5%), Phi4-Reasoning-14b (85.0%).

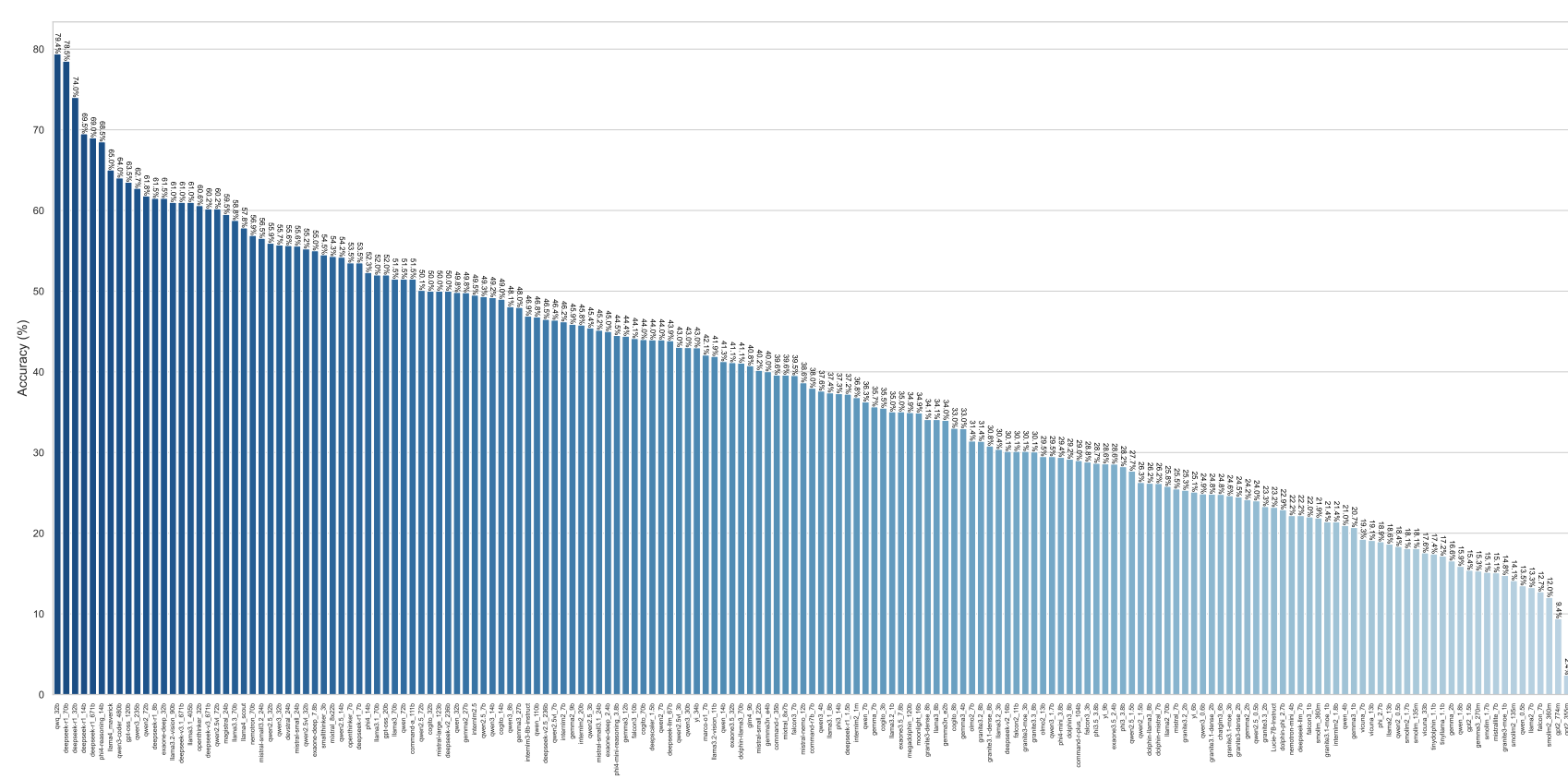

Figure S84: Bar chart of model accuracy rankings on the Associate Chief Physician Anesthesiology examination, evaluating knowledge of anesthetic pharmacology, airway management, regional anesthesia, and perioperative care. Top performers: QWQ-32b (79.4%), DeepSeek-R1-70b (78.5%), DeepSeek-R1-32b (74.0%).

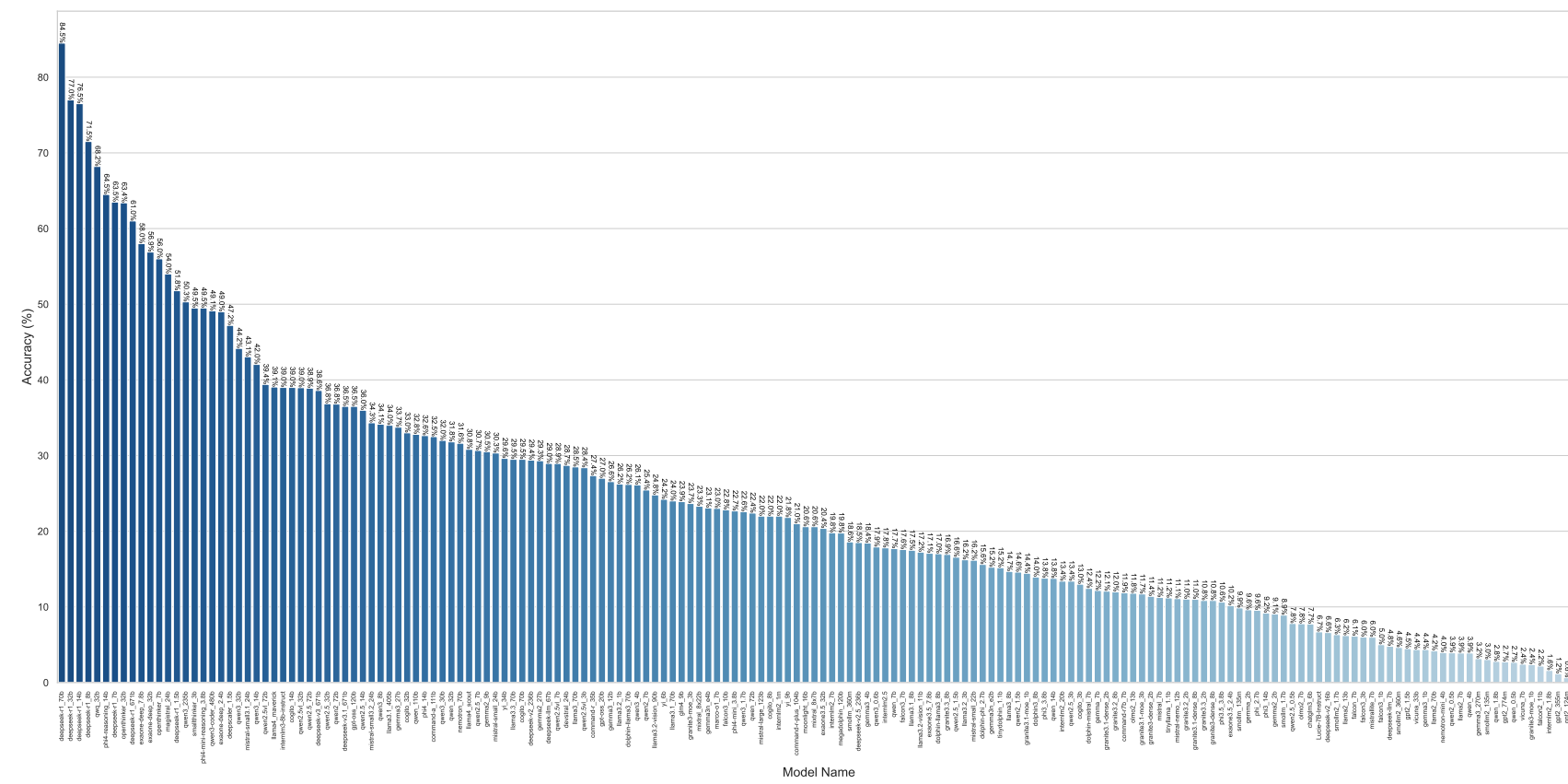

Figure S85: Bar chart of model accuracy rankings on the Chief Physician Clinical Laboratory examination, representing the highest professional level for laboratory medicine expertise including advanced diagnostic interpretation and quality management. Top performers: DeepSeek-R1-70b (84.5%), DeepSeek-R1-32b (77.0%), DeepSeek-R1-14b (76.5%).

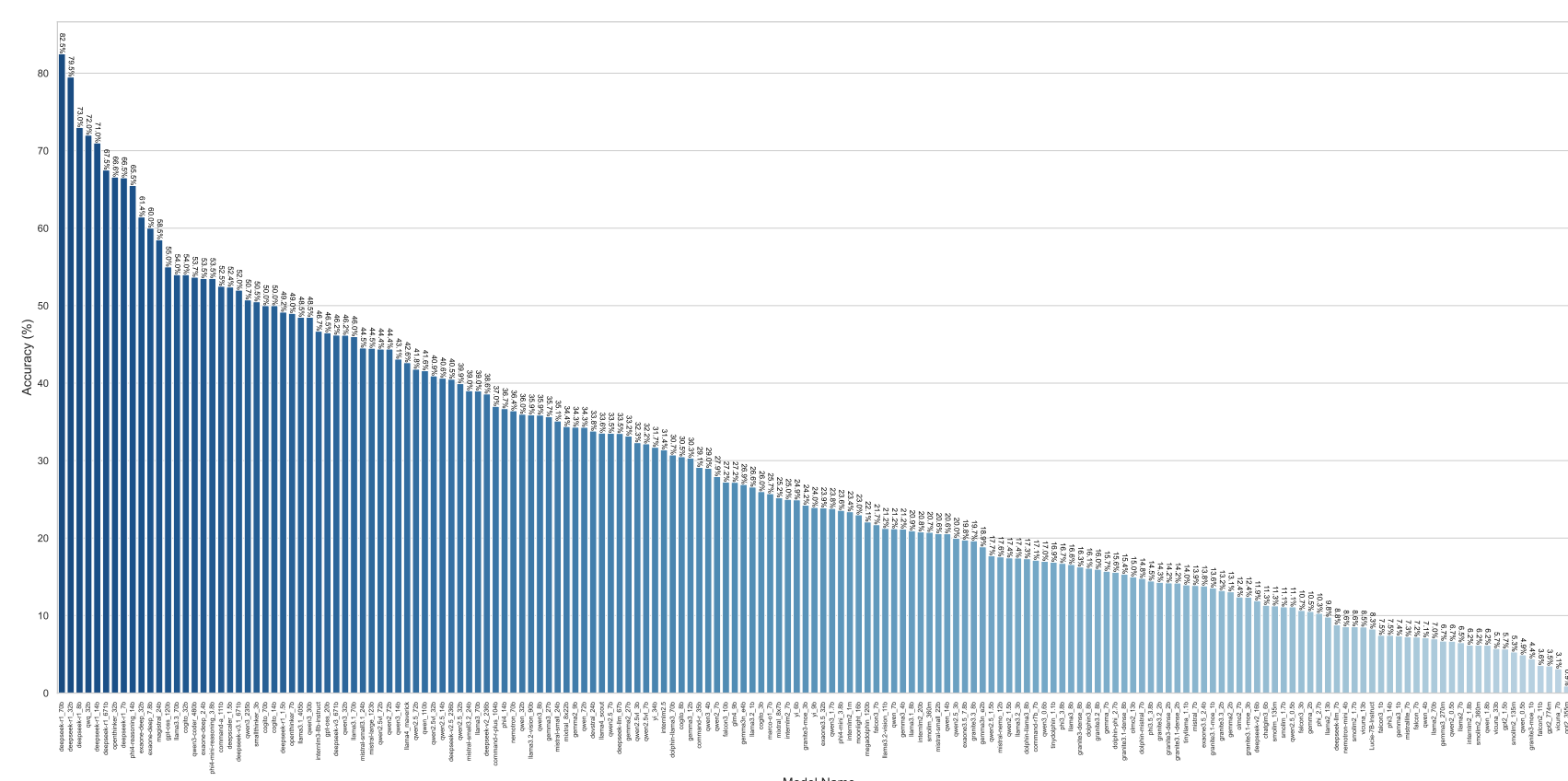

Figure S86: Bar chart of model accuracy rankings on the Chief Physician Clinical Biochemistry examination, testing highest-level expertise in biochemical testing, metabolic disorders, and laboratory method validation. Top performers: DeepSeek-R1-70b (82.5%), DeepSeek-R1-32b (79.5%), DeepSeek-R1-8b (73.0%).

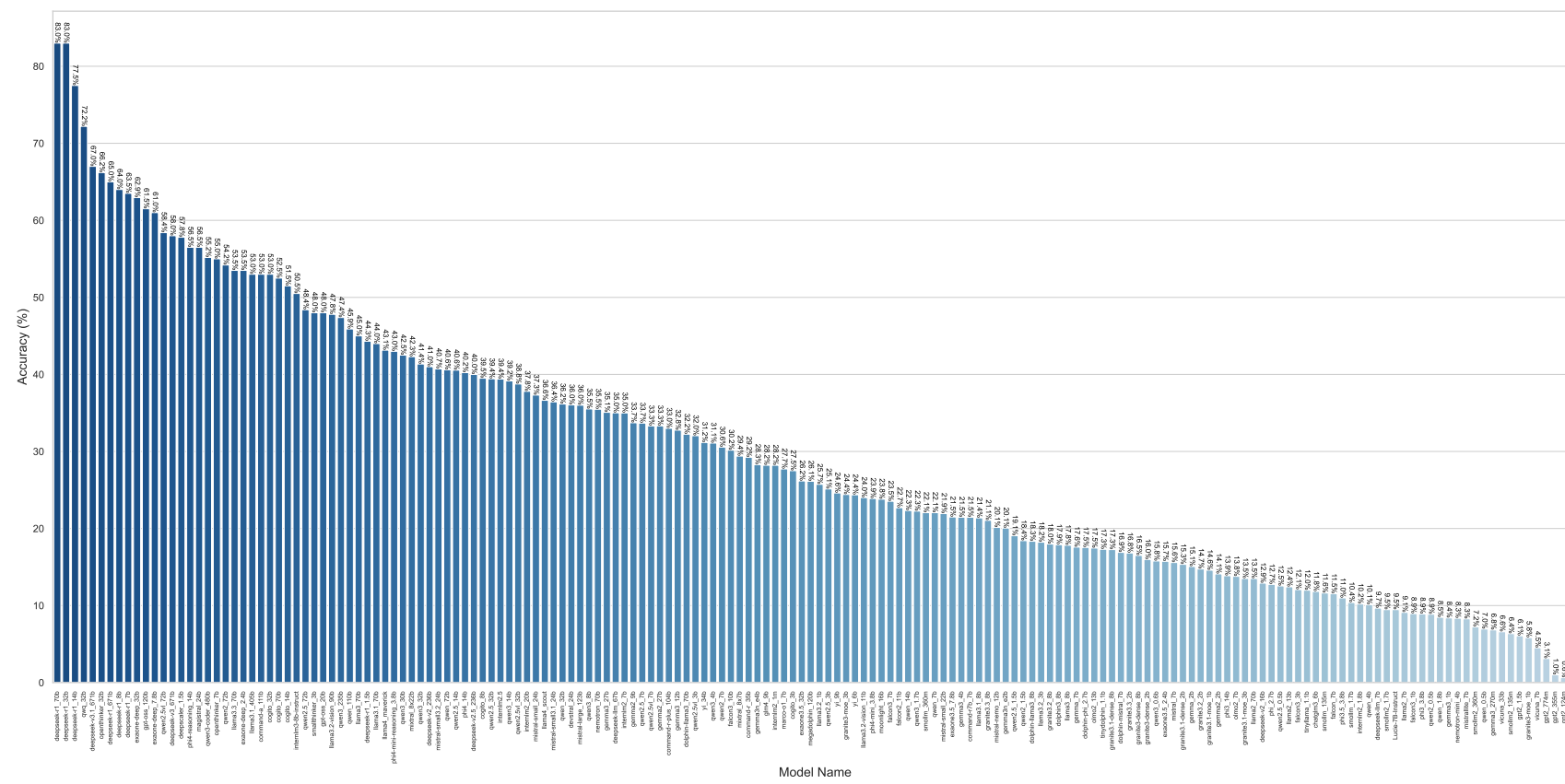

Figure S87: Bar chart of model accuracy rankings on the Chief Physician Clinical Pharmacy examination, evaluating expertise in advanced pharmacotherapy, drug policy, and pharmaceutical care leadership. Top performers: DeepSeek-R1-70b (83.0%), DeepSeek-R1-32b (83.0%), DeepSeek-R1-14b (77.5%).

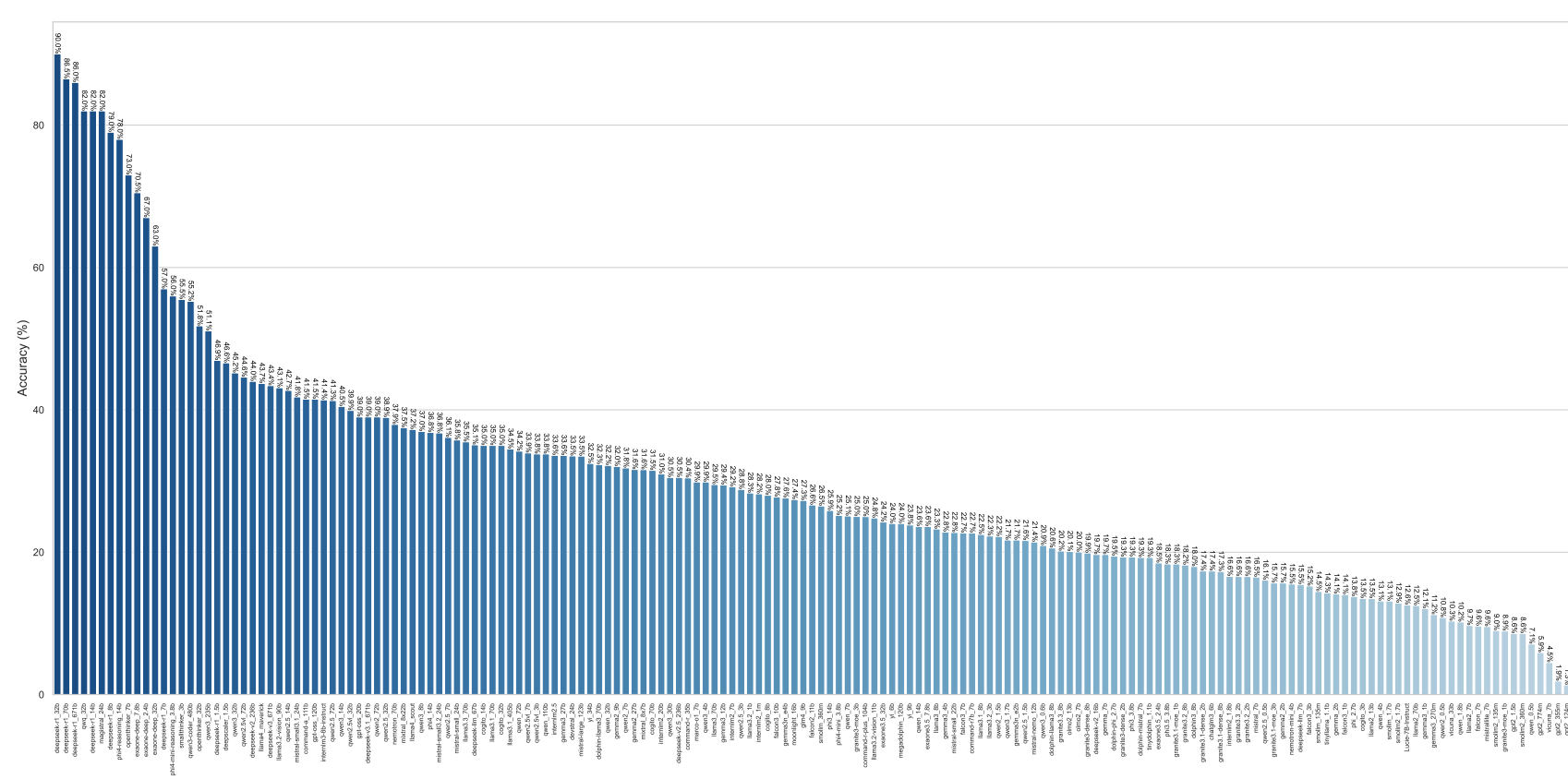

Figure S88: Bar chart of model accuracy rankings on the Chief Physician Clinical Nutrition examination, assessing advanced knowledge of nutritional therapy, metabolic support, and nutritional care program development. Top performers: DeepSeek-R1-32b (90.0%), DeepSeek-R1-70b (86.5%), DeepSeek-R1-671b (86.0%).

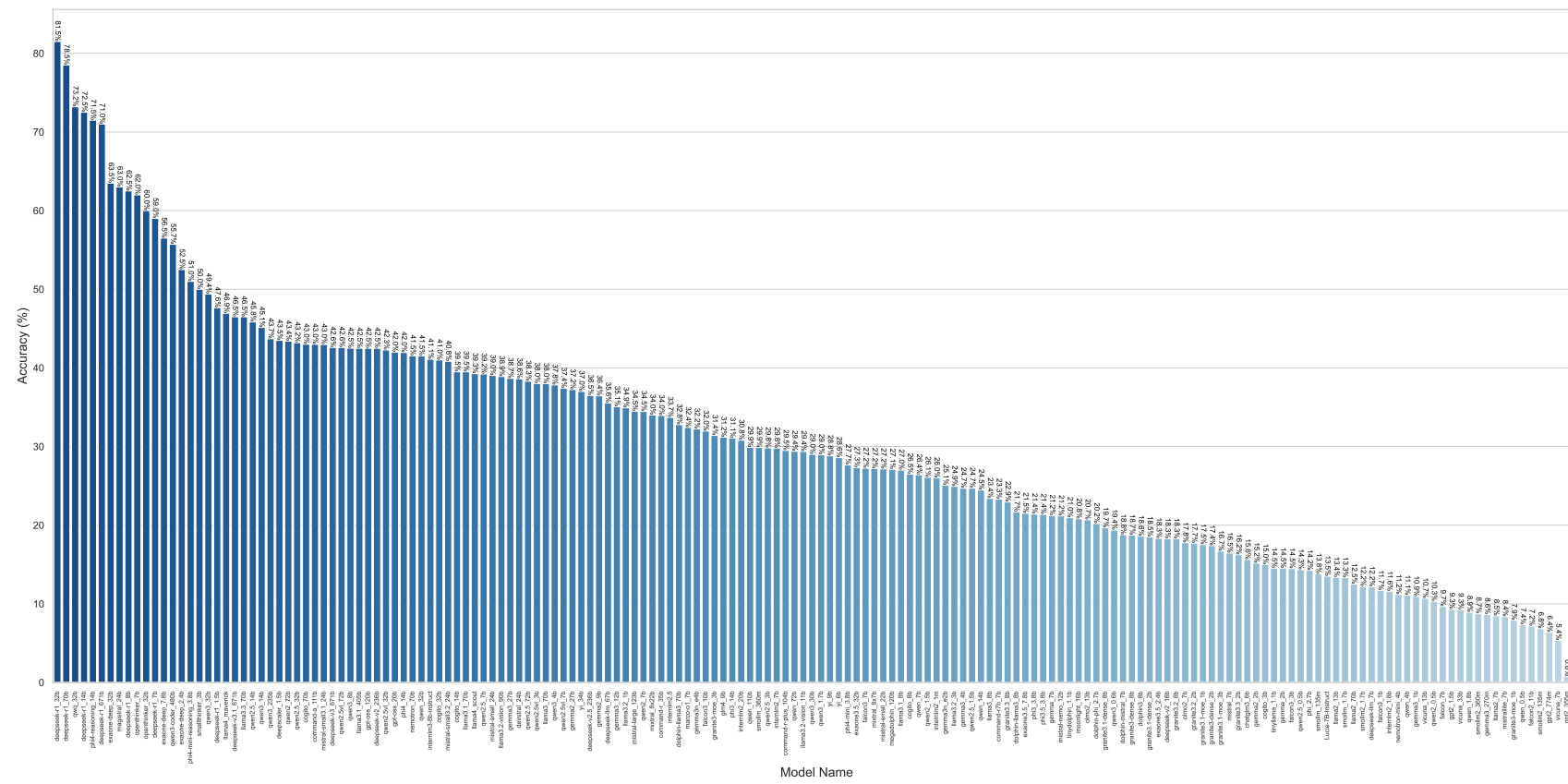

Figure S89: Bar chart of model accuracy rankings on the Chief Physician Infectious Disease examination, testing highest-level expertise in complex infections, antimicrobial stewardship, and emerging infectious disease management. Top performers: DeepSeek-R1-32b (81.5%), DeepSeek-R1-70b (78.5%), QWQ-32b (73.2%).

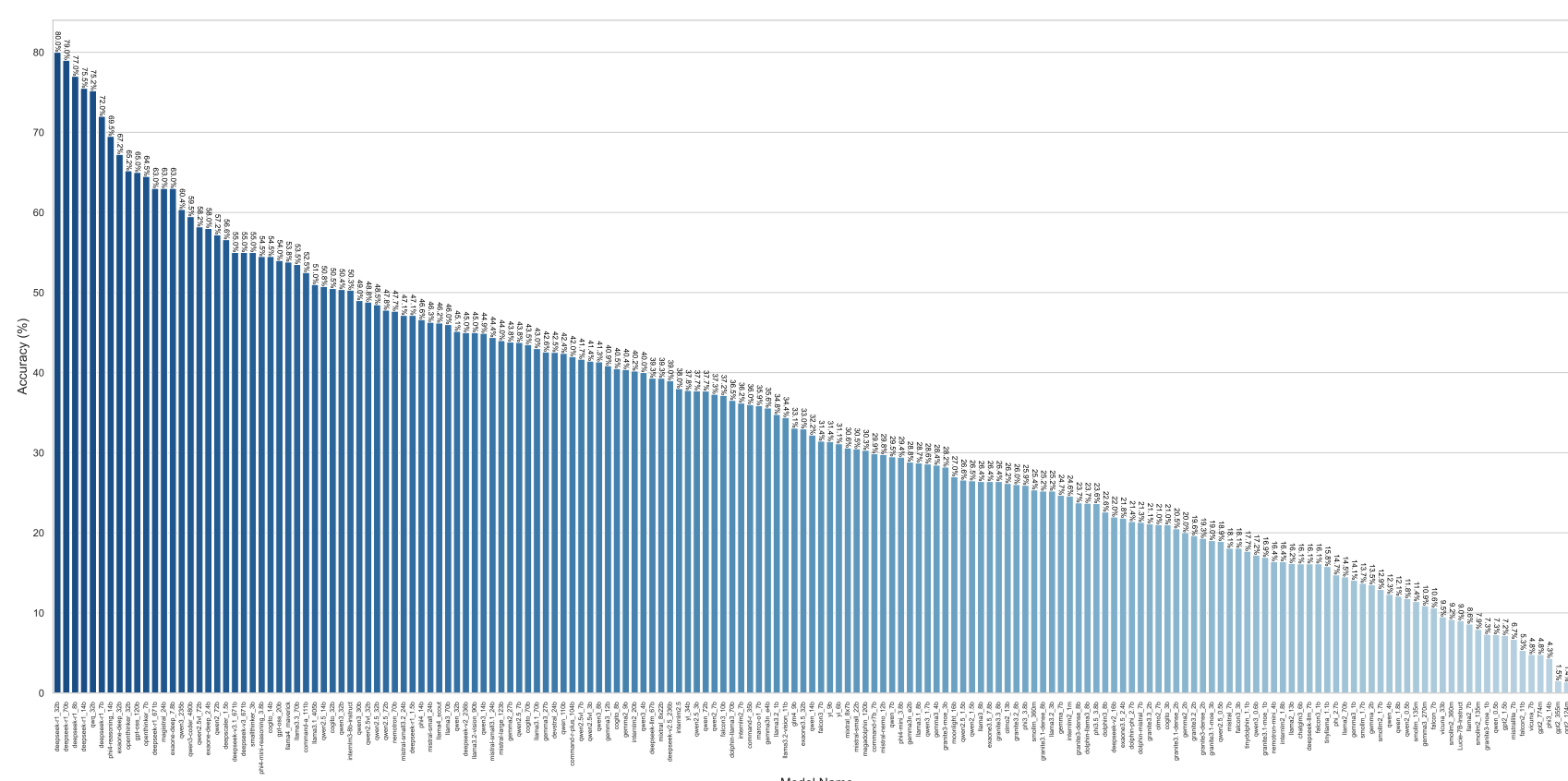

Figure S90: Bar chart of model accuracy rankings on the Chief Physician Child Healthcare examination, evaluating expertise in pediatric preventive care, developmental assessment, and child health program management. Top performers: DeepSeek-R1-32b (80.0%), DeepSeek-R1-70b (79.0%), DeepSeek-R1-8b (77.0%).

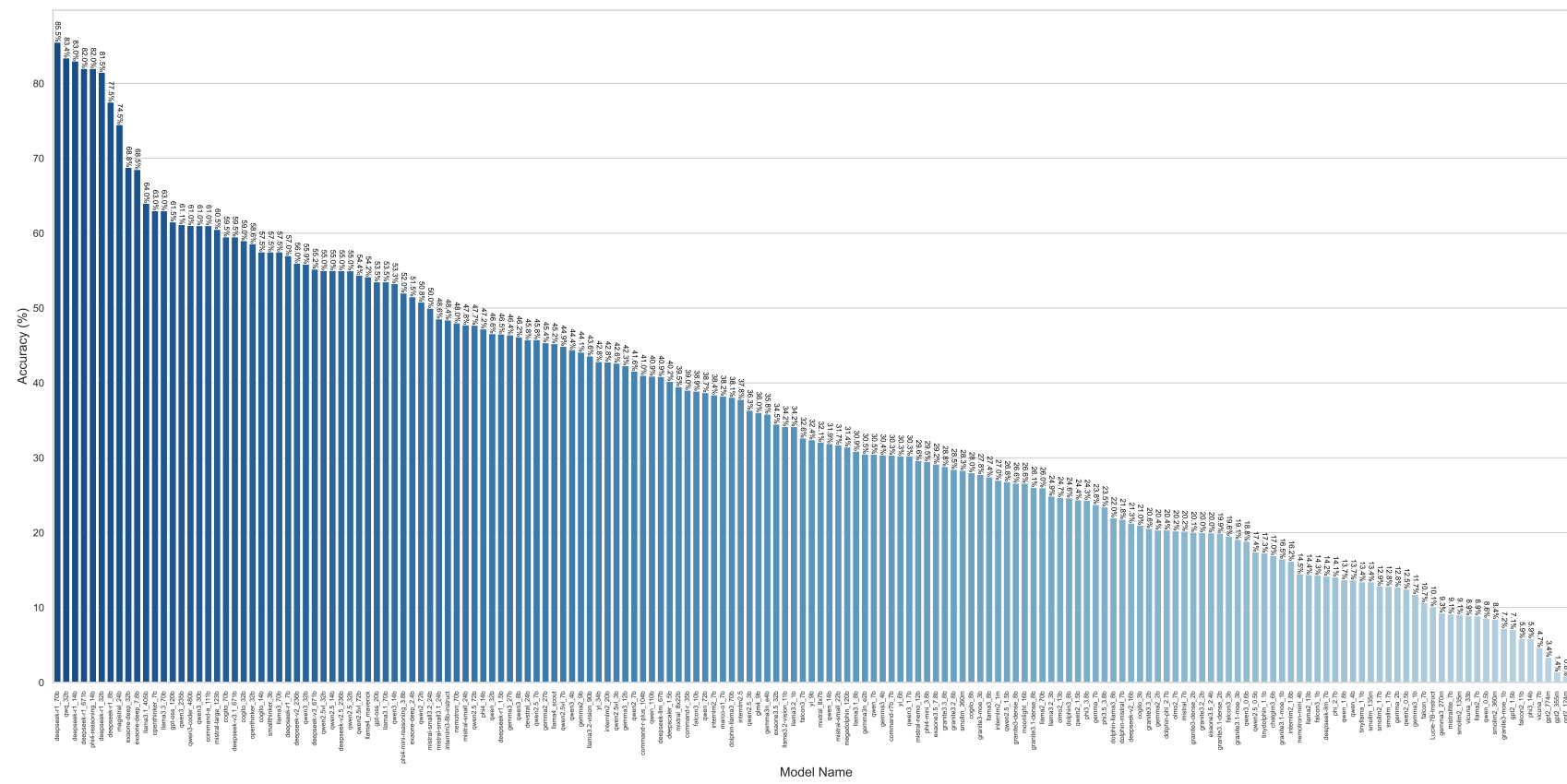

Figure S91: Bar chart of model accuracy rankings on the Chief Physician General Practice examination, assessing highest-level primary care expertise including health system leadership and community medicine. Top performers: DeepSeek-R1-70b (85.5%), QWQ-32b (83.4%), DeepSeek-R1-14b (83.0%).

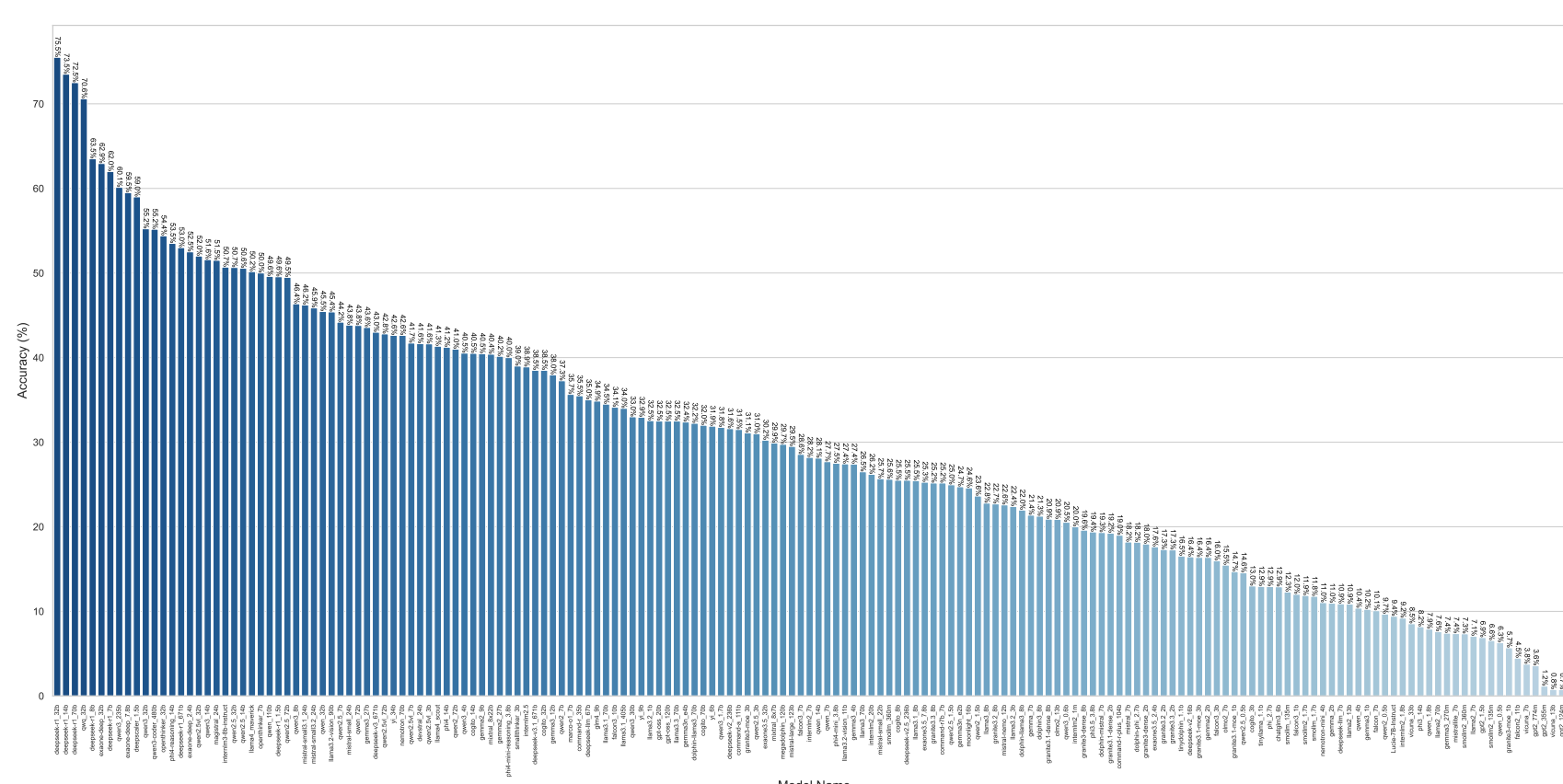

Figure S92: Bar chart of model accuracy rankings on the Chief Physician Internal Medicine Nursing examination, testing advanced nursing expertise in medical patient care and nursing leadership. Top performers: DeepSeek-R1-32b (75.5%), DeepSeek-R1-14b (73.5%), DeepSeek-R1-70b (72.5%).

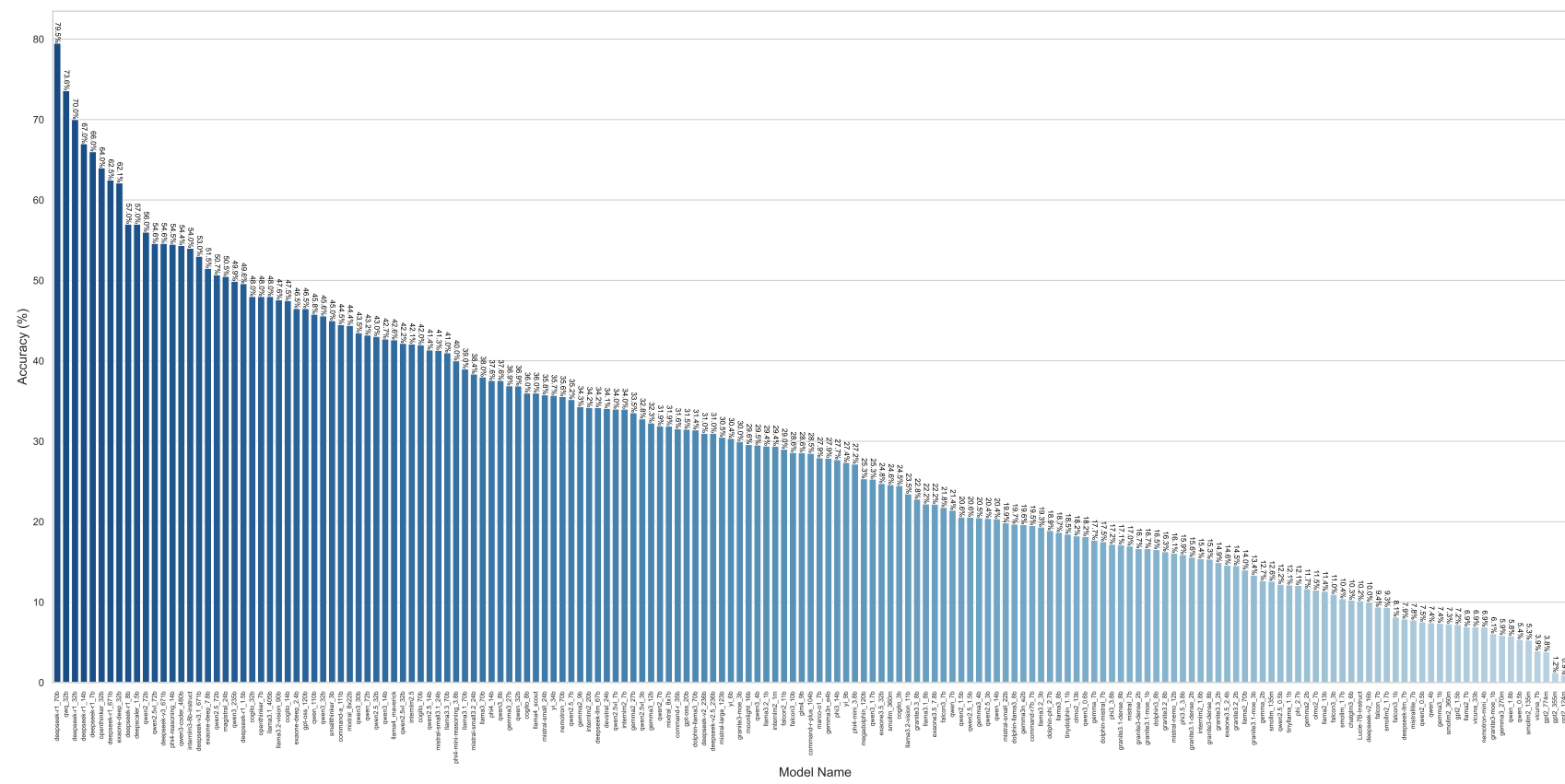

Figure S93: Bar chart of model accuracy rankings on the Chief Physician Hospital Pharmacy examination, evaluating expertise in pharmaceutical management, formulary development, and medication safety systems. Top performers: DeepSeek-R1-70b (79.5%), QWQ-32b (73.6%), DeepSeek-R1-32b (70.0%).

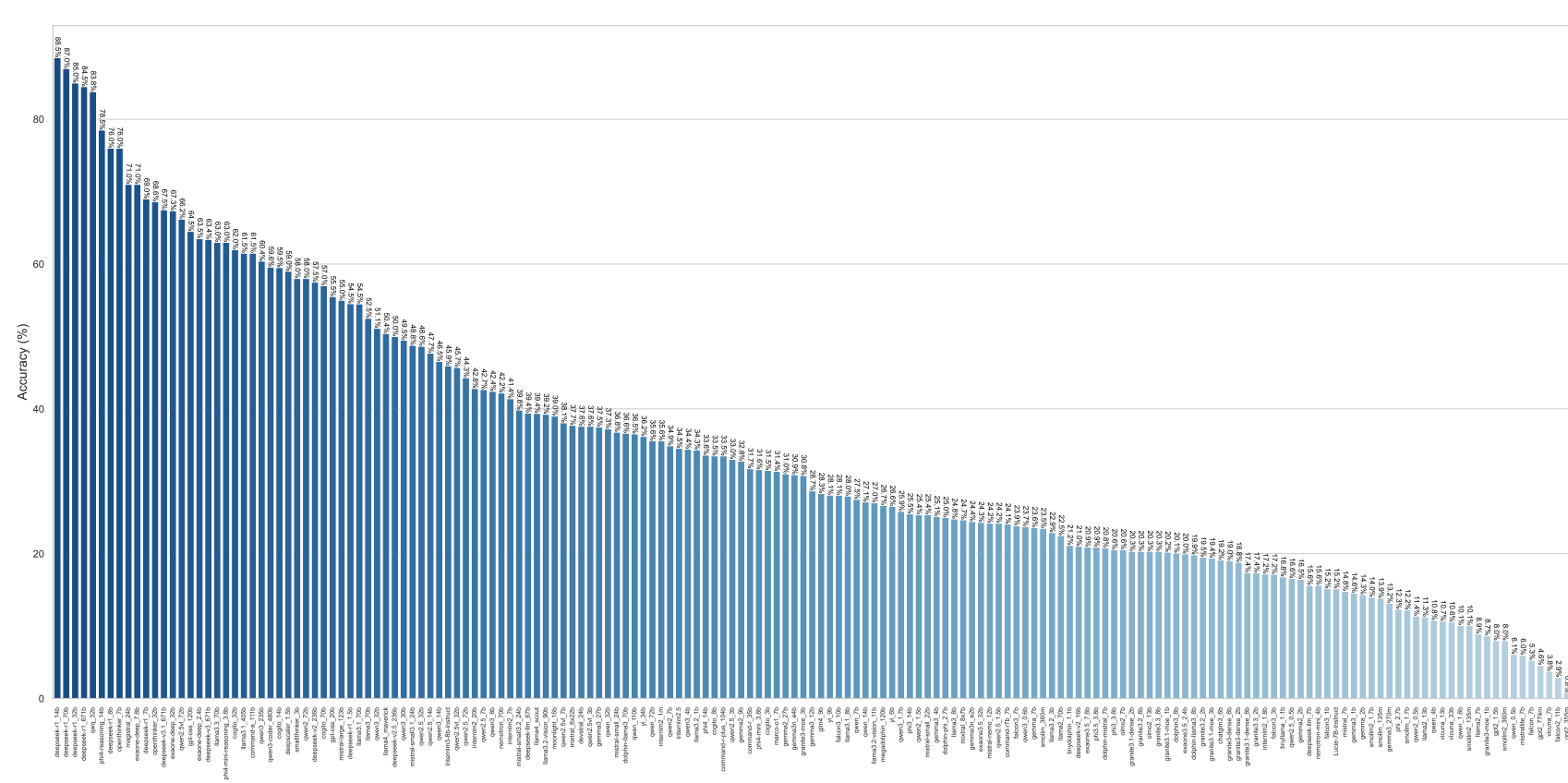

Figure S94: Bar chart of model accuracy rankings on the Chief Physician Allergy and Immunology examination, testing highest-level expertise in allergic diseases, immunodeficiencies, and immunotherapy protocols. Top performers: DeepSeek-R1-14b (88.5%), DeepSeek-R1-70b (87.0%), DeepSeek-R1-32b (85.0%).

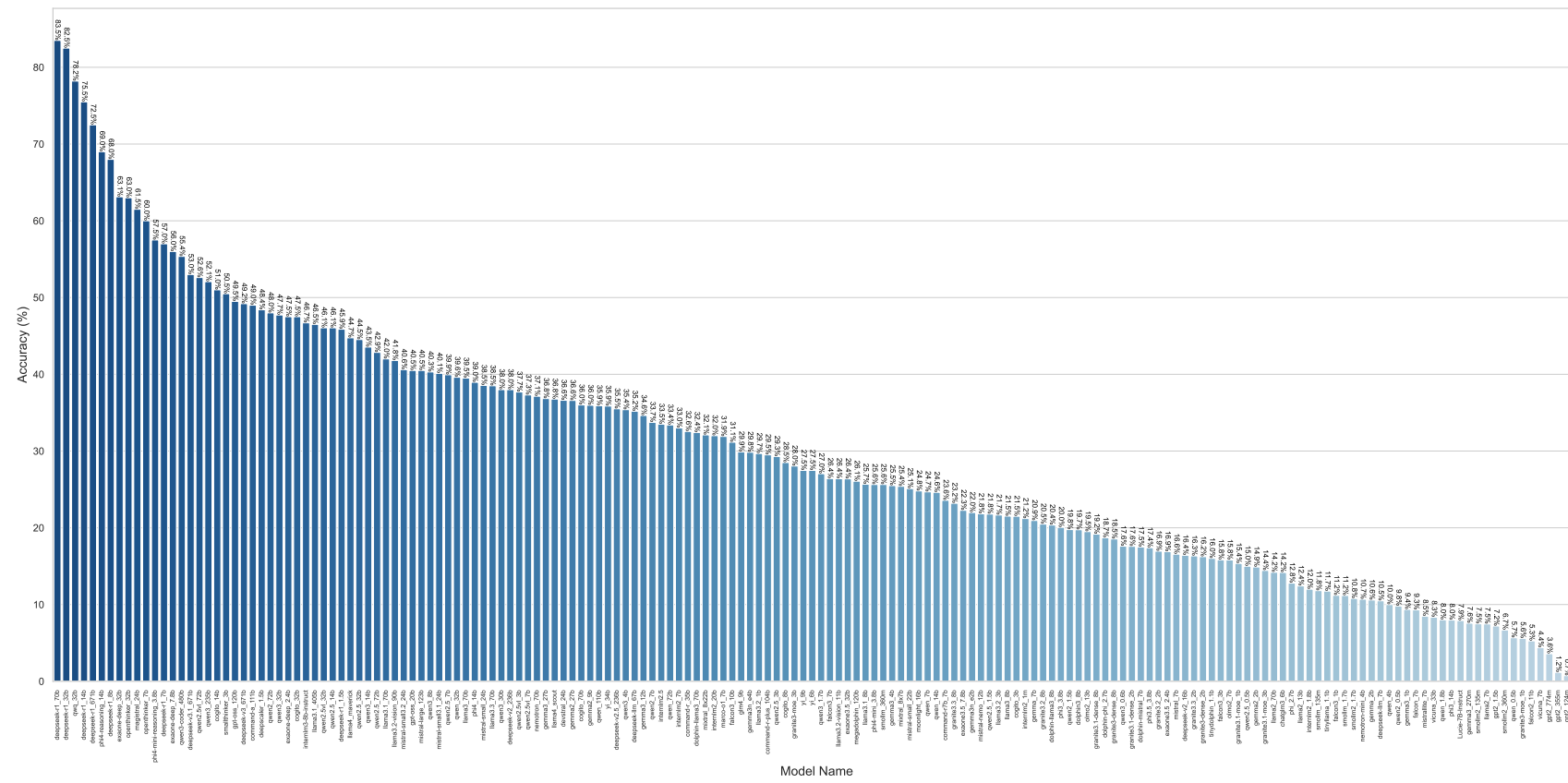

Figure S95: Bar chart of model accuracy rankings on the Chief Physician Surgical Nursing examination, assessing advanced surgical nursing expertise and perioperative care leadership. Top performers: DeepSeek-R1-70b (83.5%), DeepSeek-R1-32b (82.5%), QWQ-32b (78.2%).

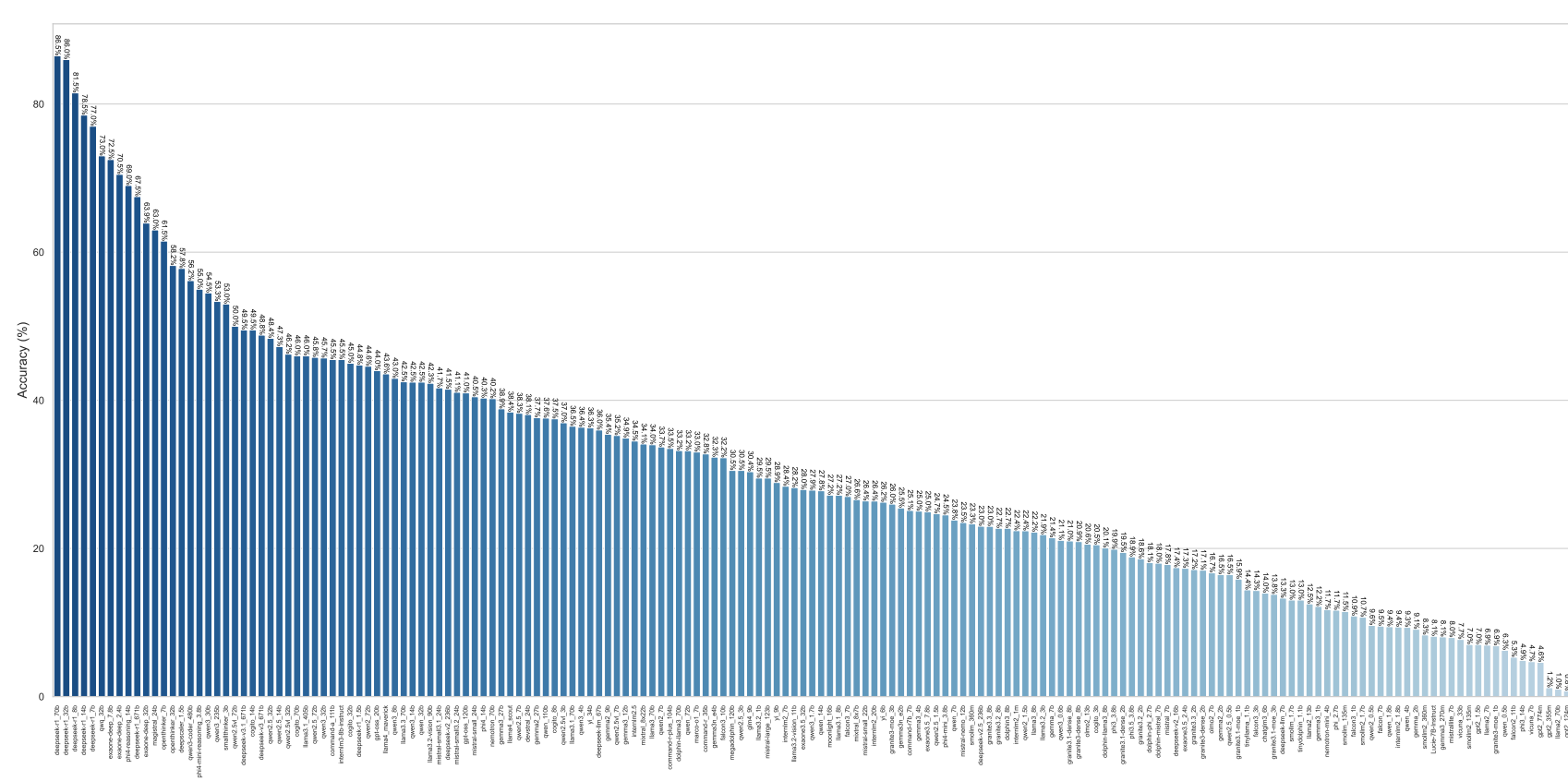

Figure S96: Bar chart of model accuracy rankings on the Chief Physician Women's Healthcare examination, evaluating expertise in maternal health, reproductive medicine, and women's preventive care programs. Top performers: DeepSeek-R1-70b (86.5%), DeepSeek-R1-32b (86.0%), DeepSeek-R1-8b (81.5%).

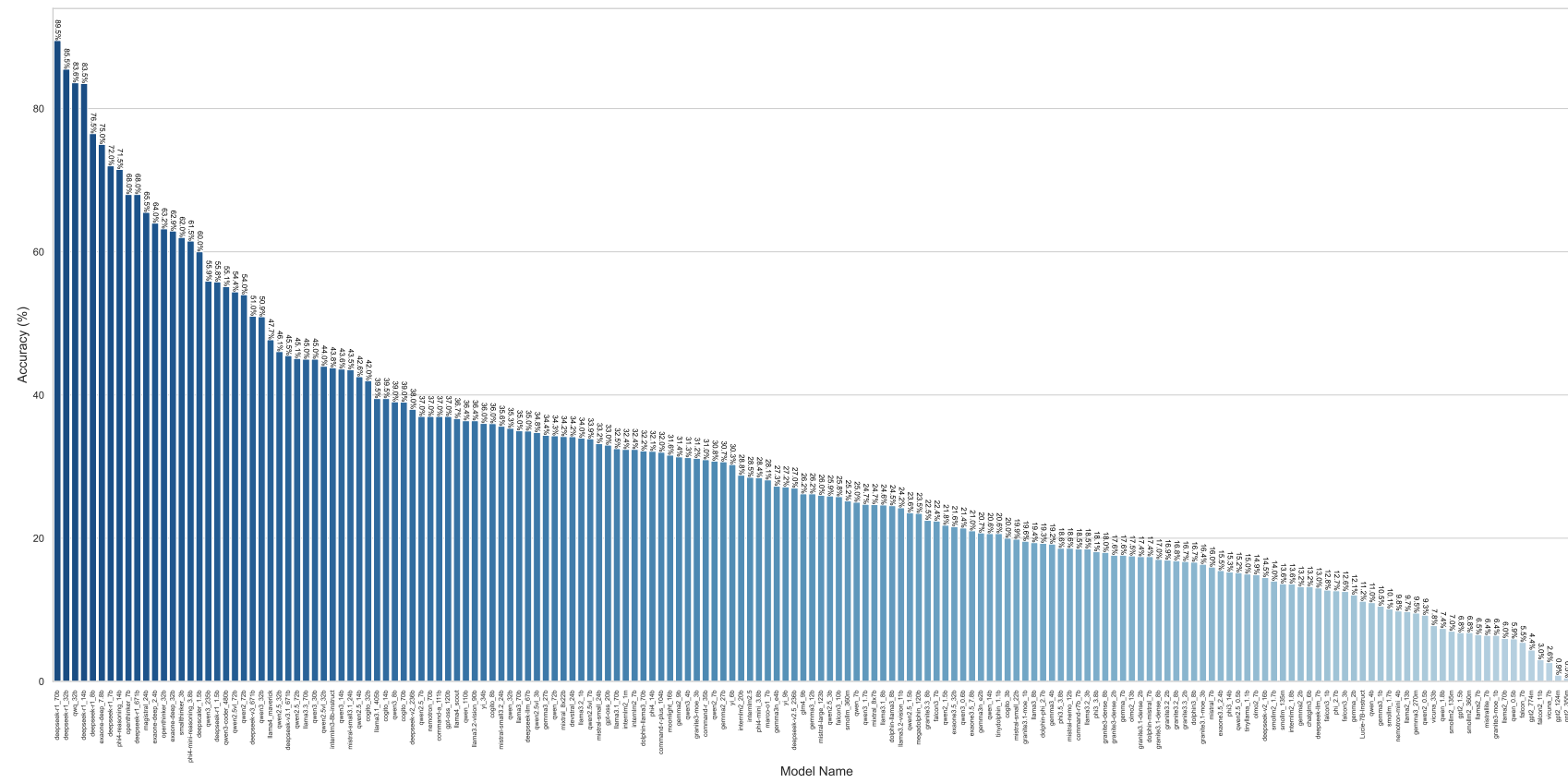

Figure S97: Bar chart of model accuracy rankings on the Chief Physician School Health and Pediatric/Adolescent Health examination, representing the highest professional level for expertise in school-based health services, adolescent development, and youth health program leadership. Top performers: DeepSeek-R1-70b (89.5%), DeepSeek-R1-32b (85.5%), QWQ-32b (83.6%).

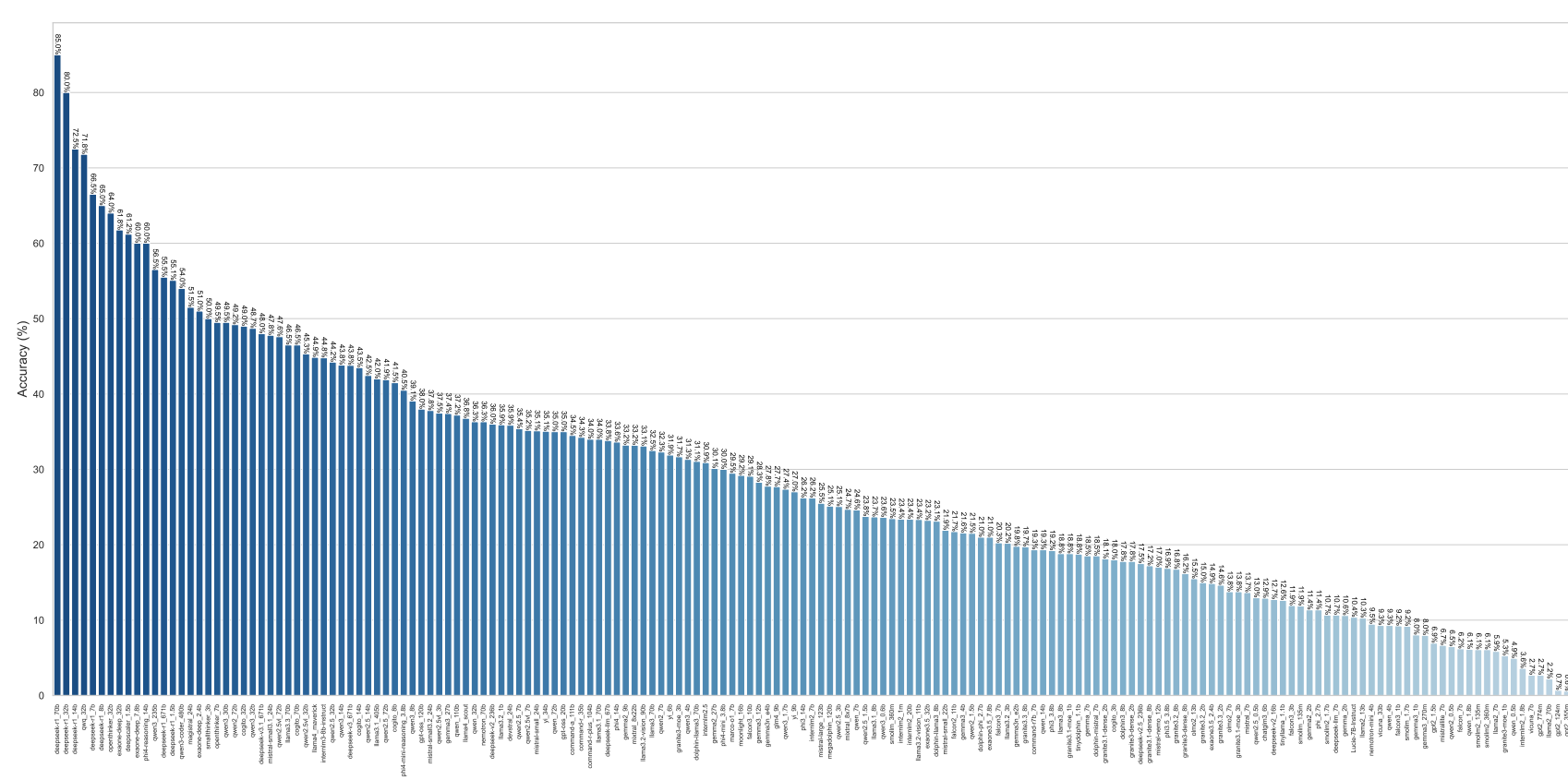

Figure S98: Bar chart of model accuracy rankings on the Chief Physician Rehabilitation Medicine examination, testing highest-level expertise in physical medicine, neurological rehabilitation, and comprehensive rehabilitation program development. Top performers: DeepSeek-R1-70b (85.0%), DeepSeek-R1-32b (80.0%), DeepSeek-R1-14b (72.5%).

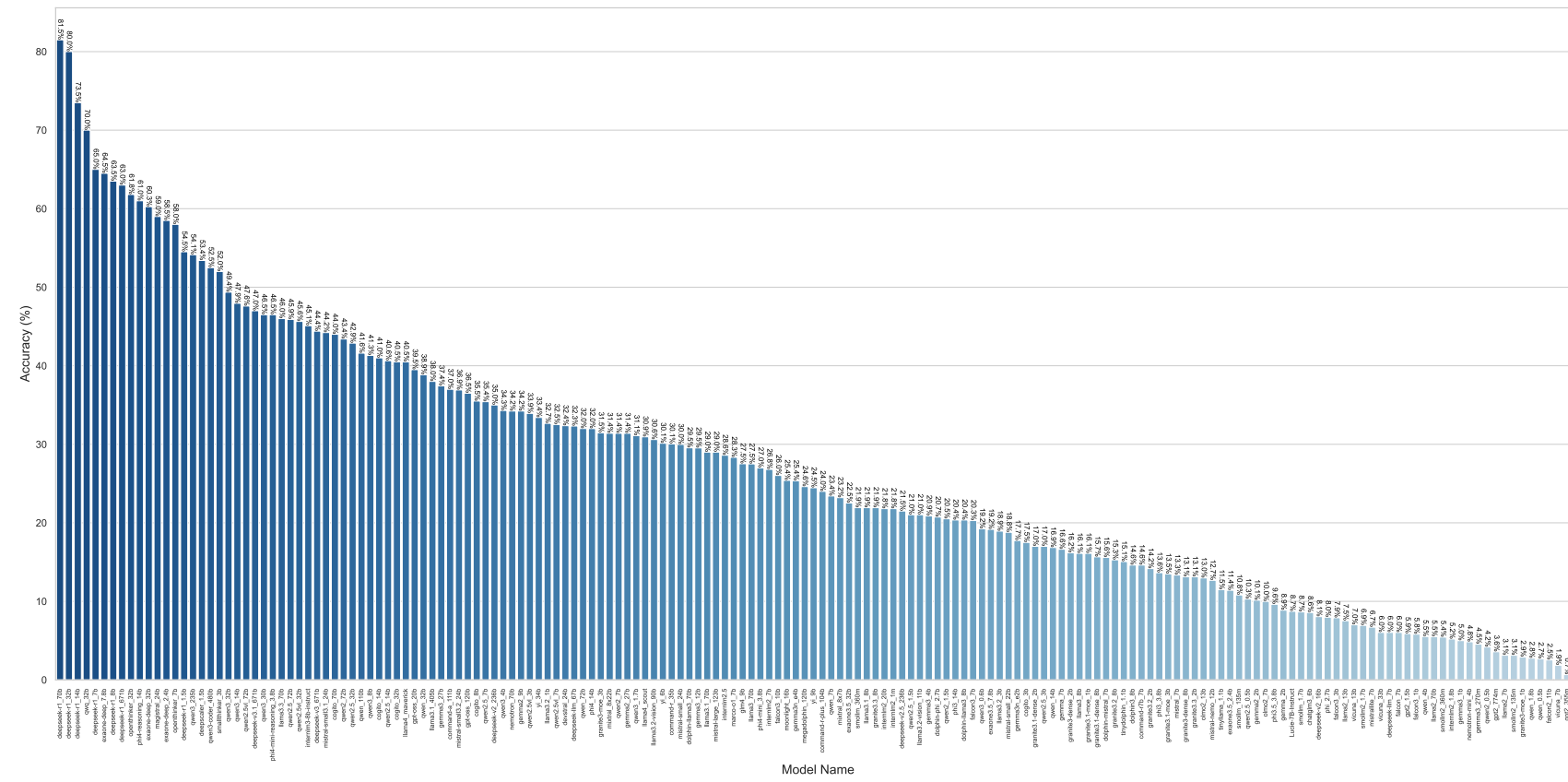

Figure S99: Bar chart of model accuracy rankings on the Chief Physician Emergency Nursing examination, representing the highest professional level for emergency nursing expertise including advanced triage, resuscitation protocols, and critical care nursing leadership. Reasoning-enhanced models (DeepSeek-R1, QWQ series) consistently achieve top performance, demonstrating superior capability in handling complex emergency clinical scenarios. Top performers: DeepSeek-R1-70b (81.5%), DeepSeek-R1-32b (80.0%), DeepSeek-R1-14b (73.5%).

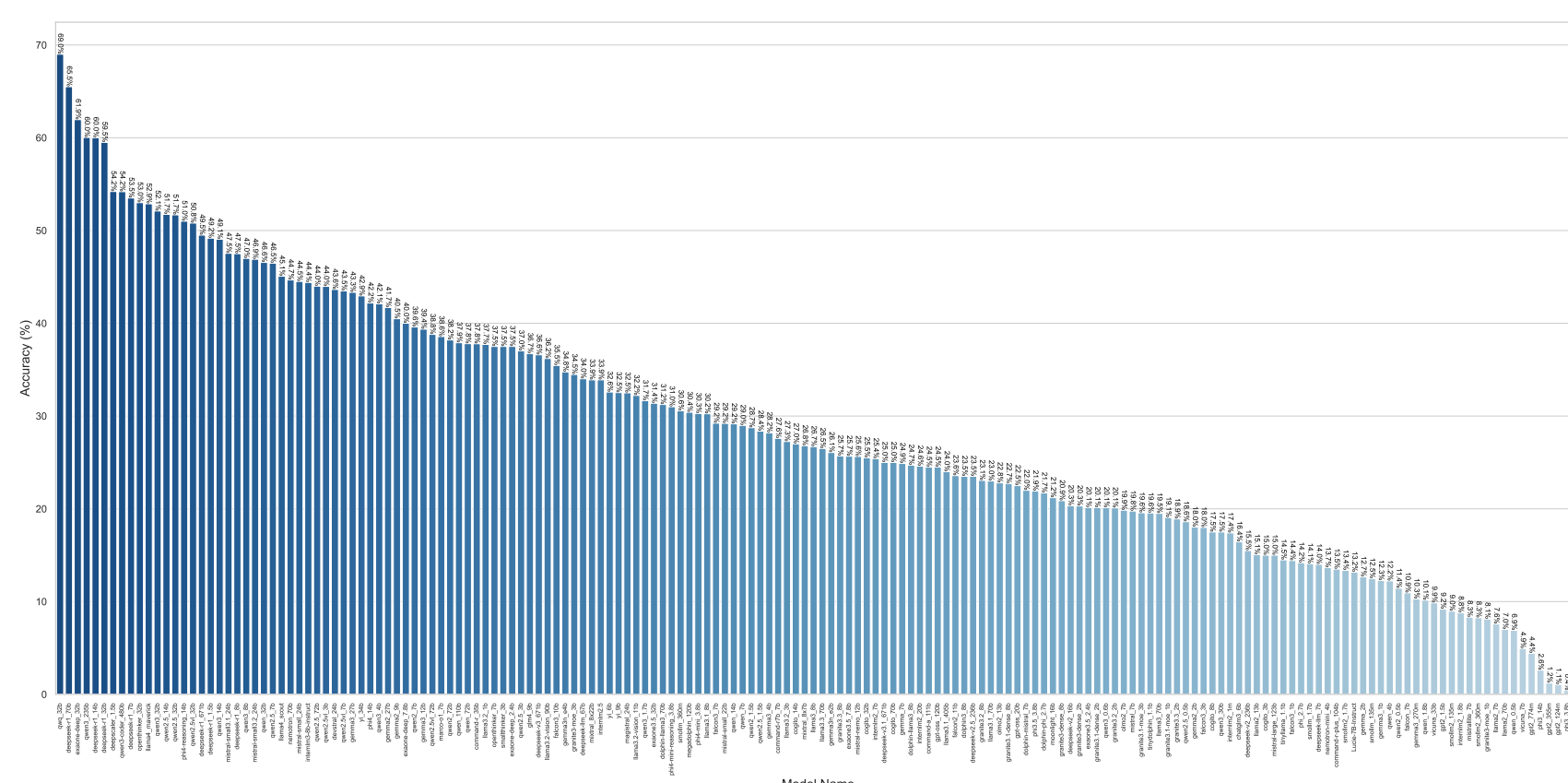

Figure S100: Bar chart of model accuracy rankings on the Chief Physician Nursing examination, assessing highest-level nursing expertise including advanced clinical nursing practice, nursing administration, and evidence-based nursing care. Top-performing reasoning models achieve accuracies significantly exceeding general-purpose models, reflecting the benefit of explicit reasoning training for complex nursing decision-making scenarios. Top performers: QWQ-32b (69.0%), DeepSeek-R1-70b (65.5%), ExaOne-Deep-32b (61.9%).

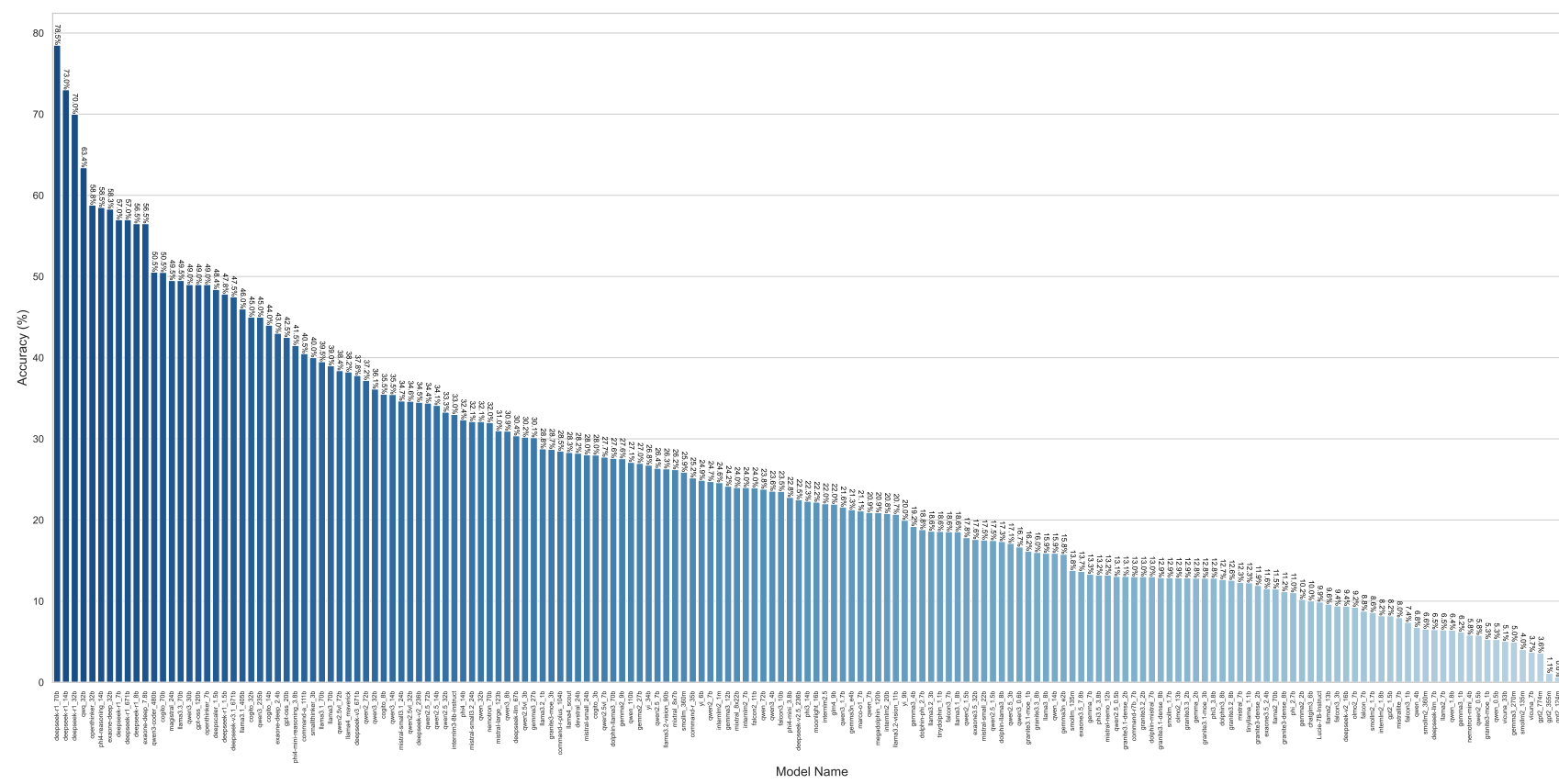

Figure S101: Bar chart of model accuracy rankings on the Chief Physician Radiation Medicine examination, testing highest-level expertise in radiation physics, radiation protection, diagnostic radiology, and radiation safety management. Leading reasoning-enhanced models demonstrate strong performance on this technically demanding specialty, with DeepSeek-R1 variants achieving top accuracy through sophisticated understanding of radiation dosimetry and clinical applications. Top performers: DeepSeek-R1-70b (78.5%), DeepSeek-R1-14b (73.0%), DeepSeek-R1-32b (70.0%).

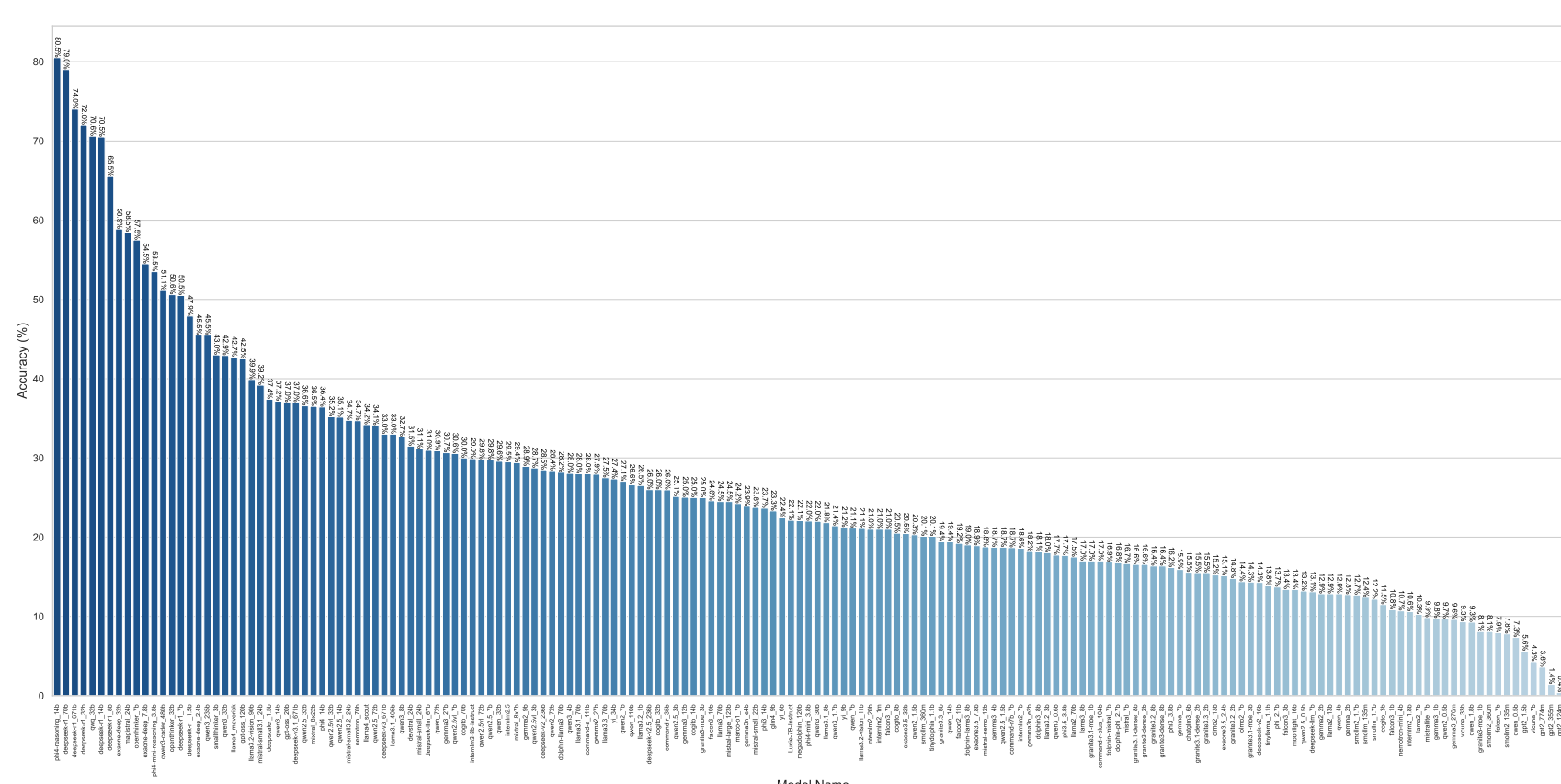

Figure S102: Bar chart of model accuracy rankings on the Chief Physician Radiation Oncology examination, evaluating expertise in radiotherapy treatment planning, brachytherapy, stereotactic radiosurgery, and management of radiation-induced toxicities. This challenging specialty requires integration of physics and clinical knowledge, where reasoning-enhanced models significantly outperform general-purpose alternatives. Top performers: Phi4-Reasoning-14b (80.5%), DeepSeek-R1-70b (79.0%), DeepSeek-R1-671b (74.0%).

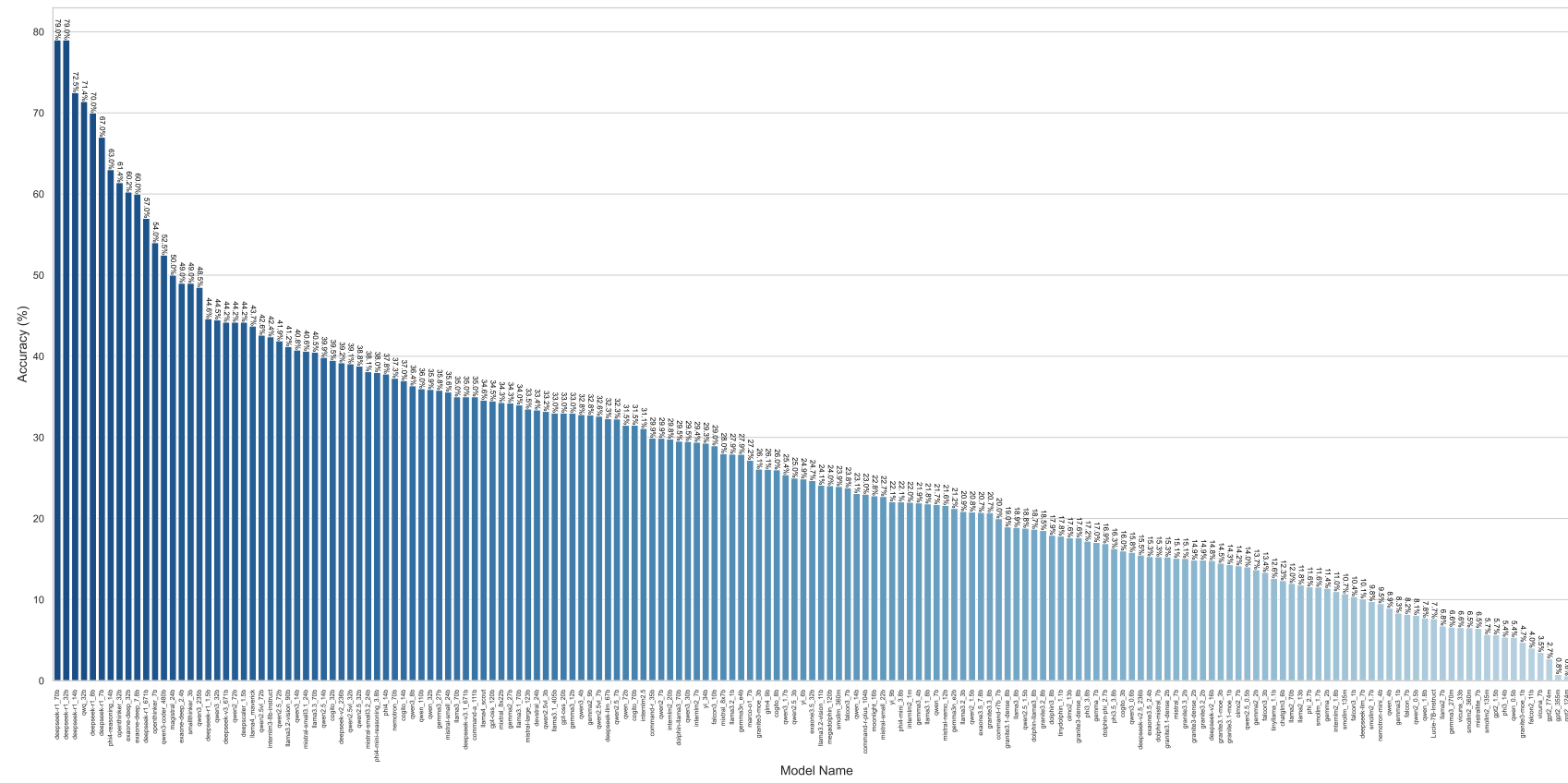

Figure S103: Bar chart of model accuracy rankings on the Chief Physician General Internal Medicine examination, representing the highest professional level for comprehensive internal medicine expertise including complex differential diagnosis, multi-system disease management, and clinical decision-making. Top reasoning models achieve exceptional accuracy, demonstrating robust generalization across the broad spectrum of internal medicine subspecialties. Top performers: DeepSeek-R1-70b (79.0%), DeepSeek-R1-32b (79.0%), DeepSeek-R1-14b (72.5%).

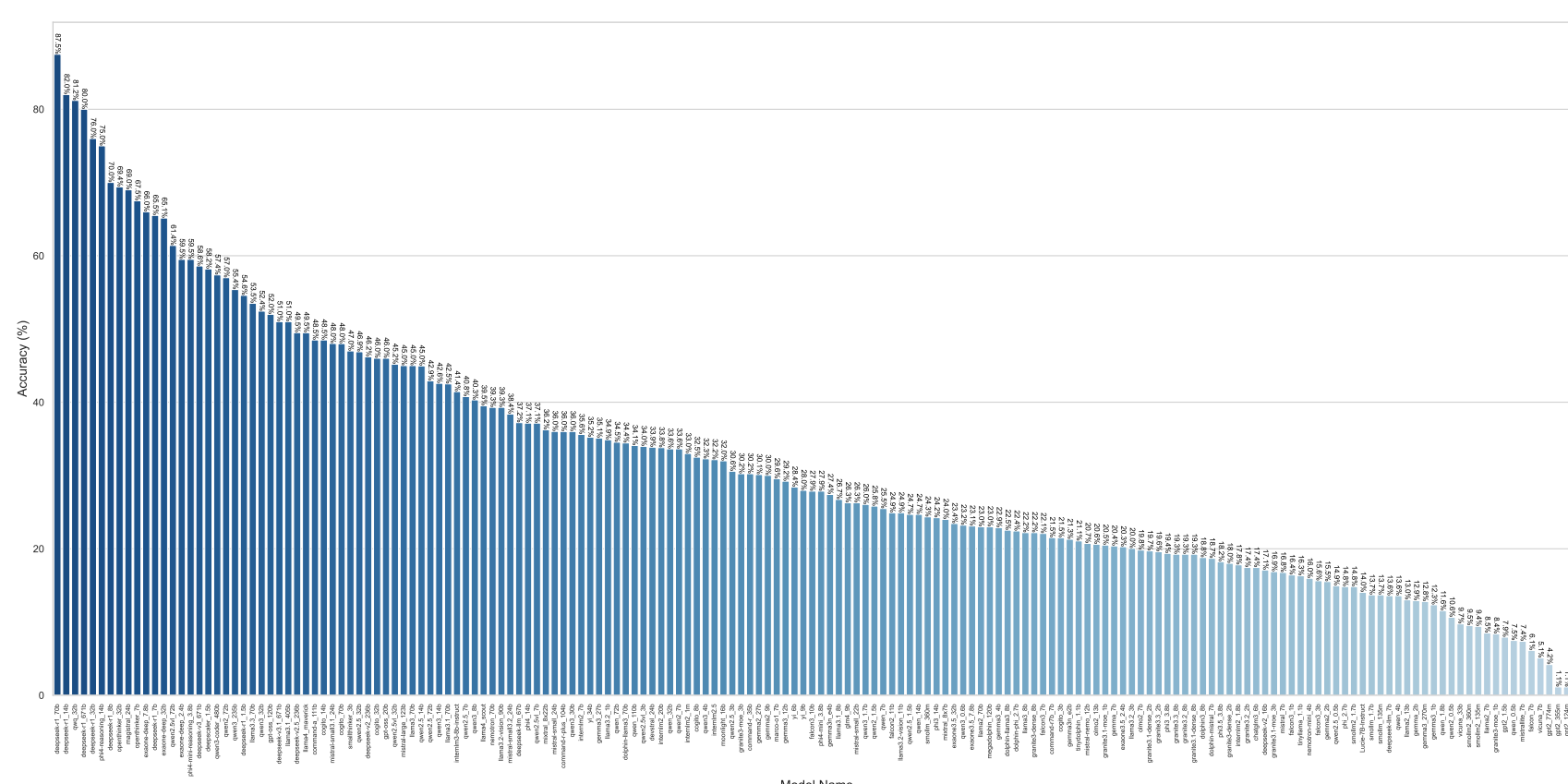

Figure S104: Bar chart of model accuracy rankings on the Chief Physician Nuclear Medicine examination, testing highest-level expertise in radiopharmaceuticals, nuclear imaging modalities (PET, SPECT), radionuclide therapy, and radiation safety. This specialized field requires integration of physics, pharmacology, and clinical medicine, where reasoning-enhanced architectures demonstrate clear advantages over general-purpose models. Top performers: DeepSeek-R1-70b (87.5%), DeepSeek-R1-14b (82.0%), QWQ-32b (81.2%).

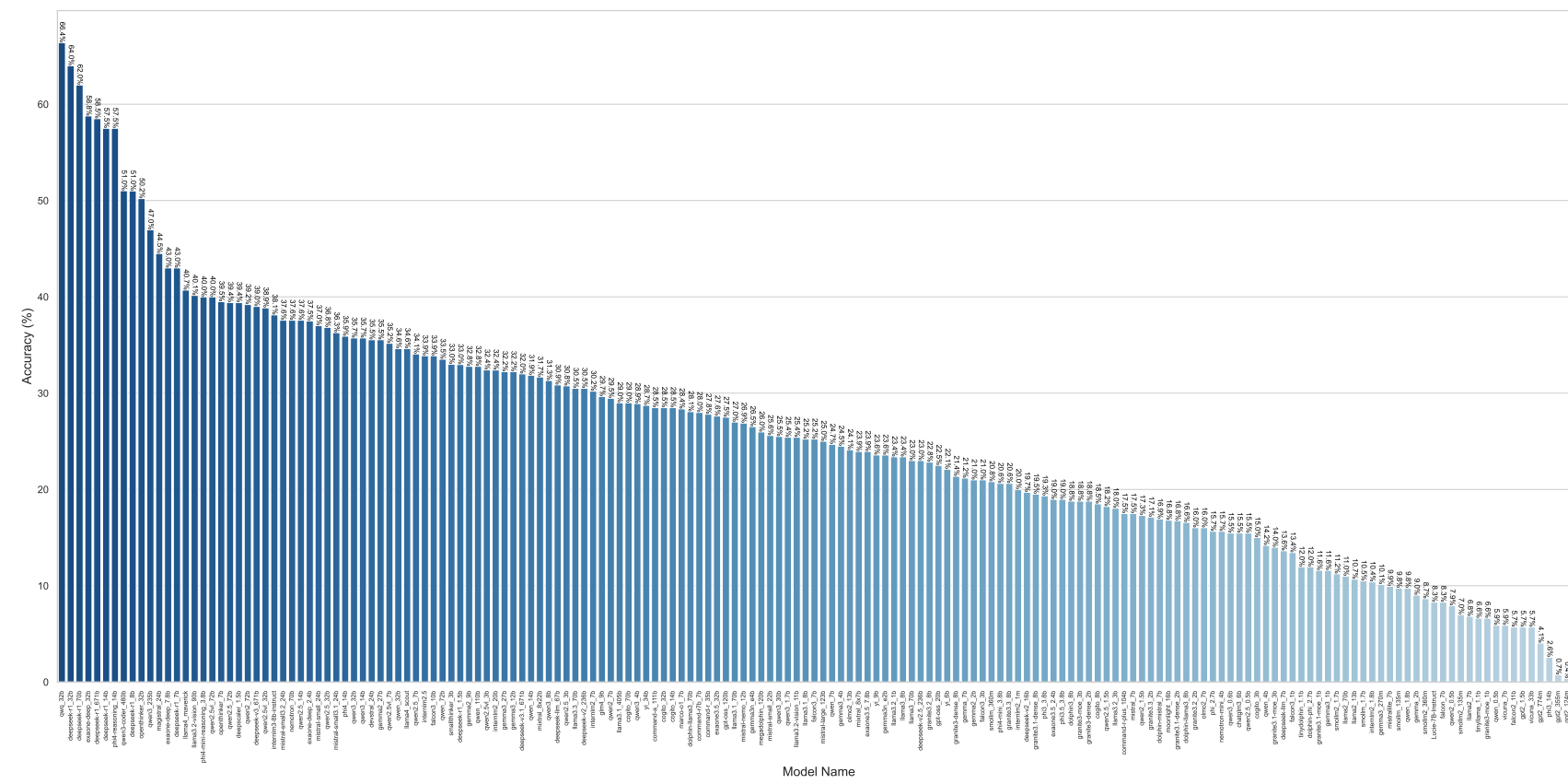

Figure S105: Bar chart of model accuracy rankings on the Chief Physician Pain Medicine examination, assessing highest-level expertise in pain pathophysiology, chronic pain syndromes, interventional pain management, and multimodal analgesia strategies. Reasoning-enhanced models excel at integrating the complex pharmacological and procedural knowledge required for comprehensive pain management, achieving top accuracy in this multidisciplinary specialty. Top performers: QWQ-32b (66.4%), DeepSeek-R1-32b (64.0%), DeepSeek-R1-70b (62.0%).

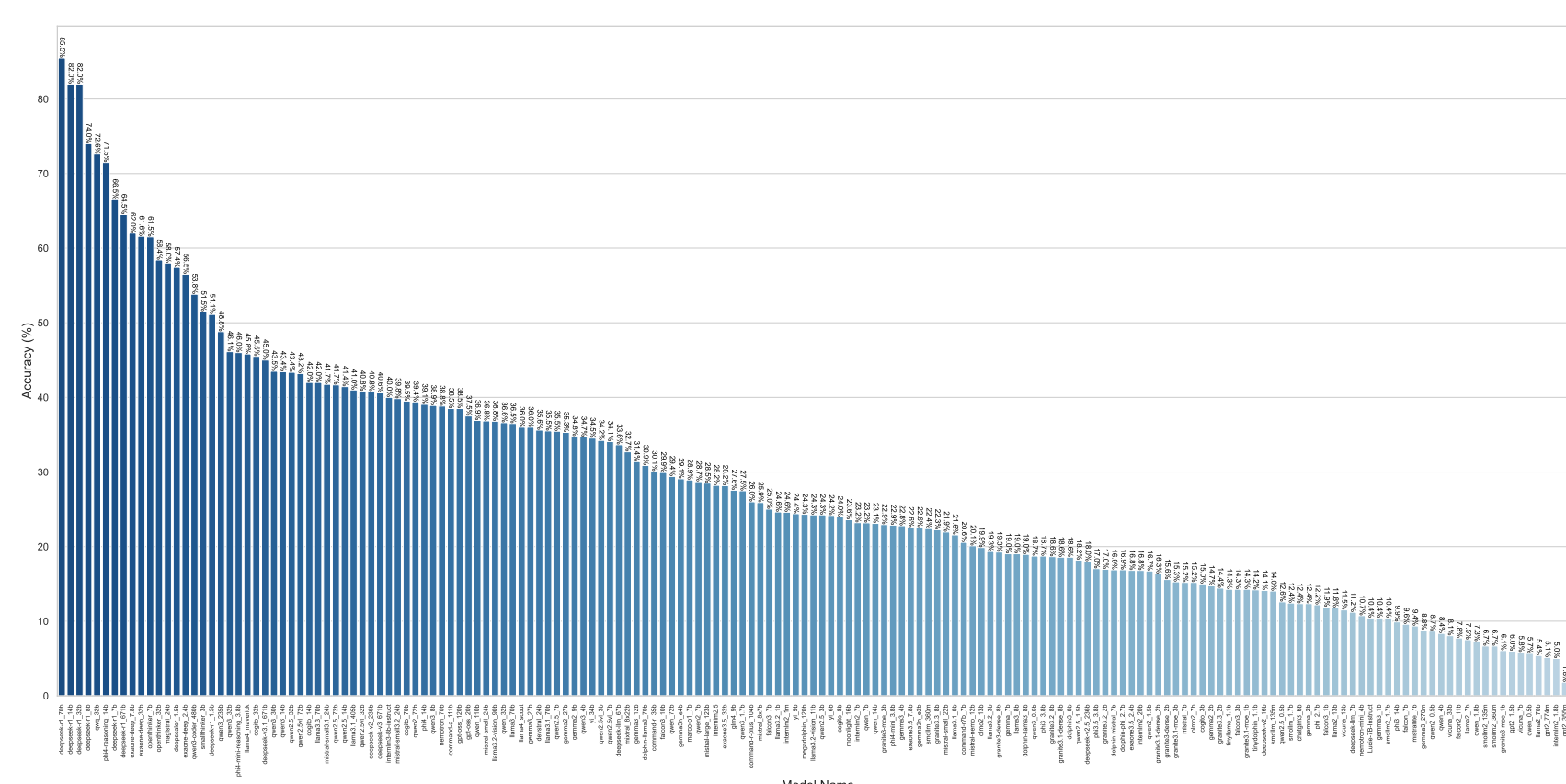

Figure S106: Bar chart of model accuracy rankings on the Chief Physician Disease Prevention and Control examination, testing highest-level expertise in epidemiology, public health surveillance, outbreak investigation, and population health management. This public health specialty requires integration of biostatistics, epidemiological methods, and health policy knowledge, where reasoning-enhanced models demonstrate robust performance through systematic analytical capabilities. Top performers: DeepSeek-R1-70b (85.5%), DeepSeek-R1-14b (82.0%), DeepSeek-R1-32b (82.0%).

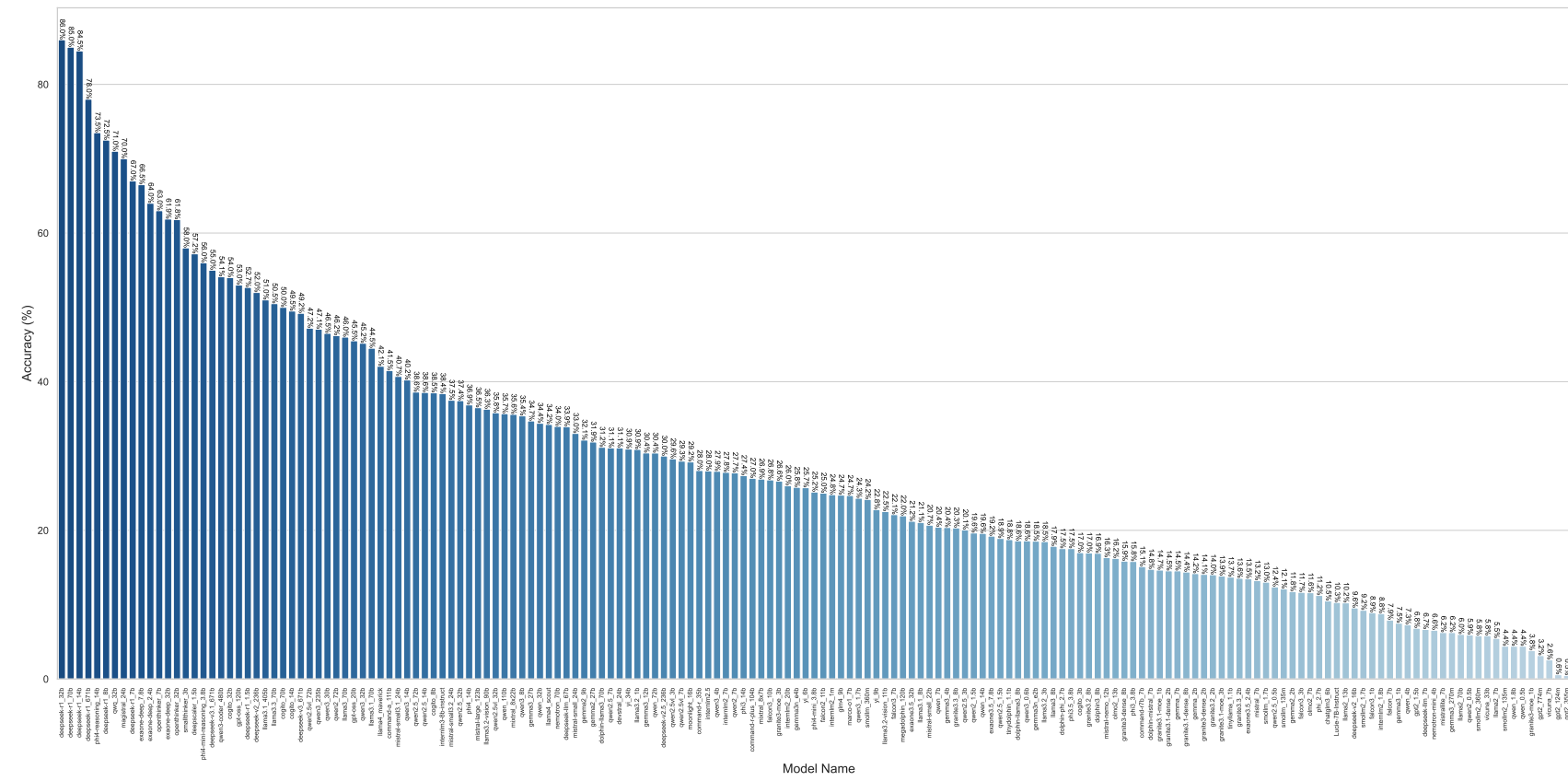

Figure S107: Bar chart of model accuracy rankings on the Chief Physician Pathology examination, representing the highest professional level for pathology expertise including advanced histopathology, molecular pathology, cytopathology, and autopsy medicine. Top reasoning models achieve exceptional accuracy through sophisticated pattern recognition and diagnostic reasoning capabilities essential for pathological diagnosis. Top performers: DeepSeek-R1-32b (86.0%), DeepSeek-R1-70b (85.0%), DeepSeek-R1-14b (84.5%).

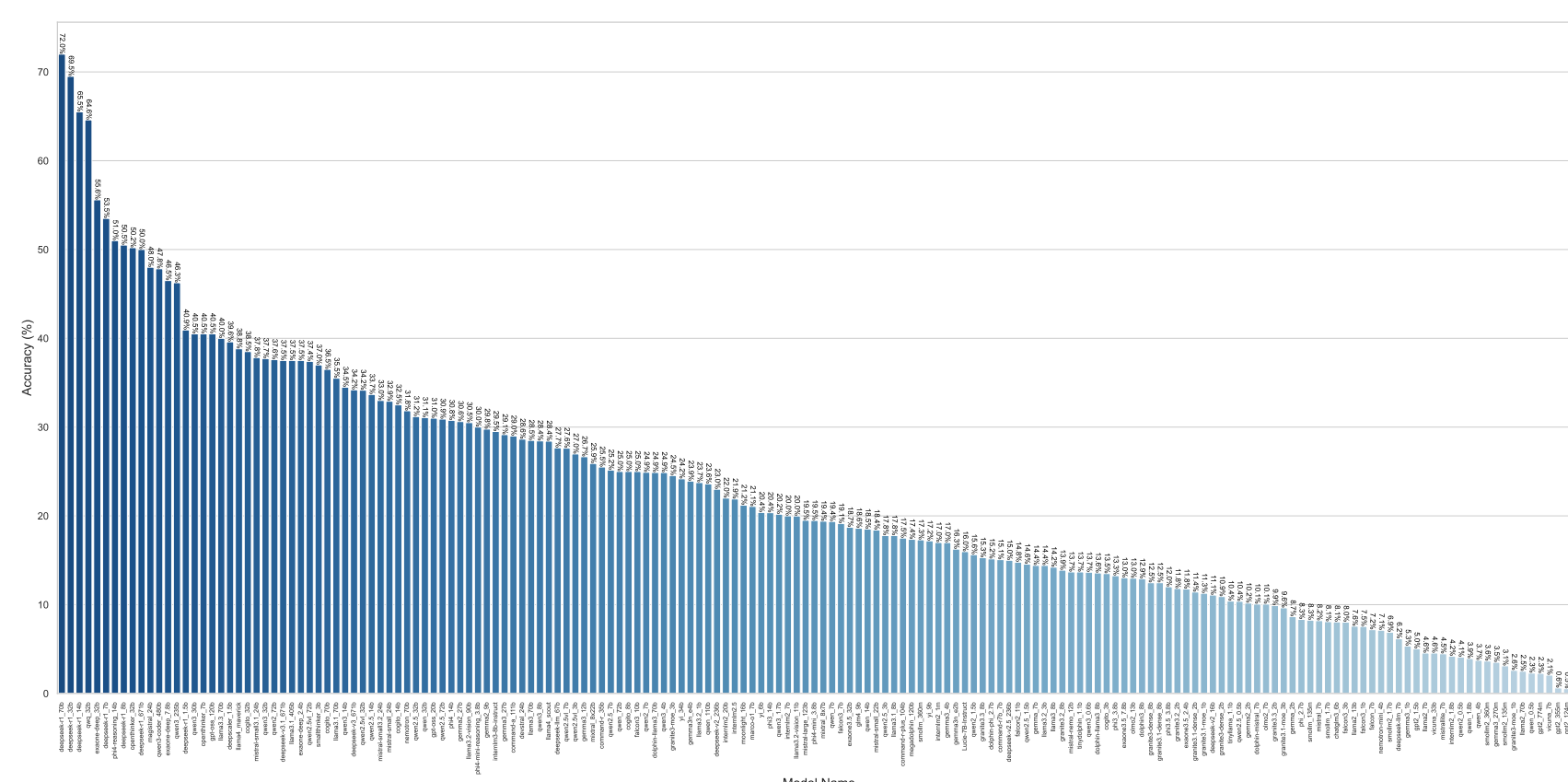

Figure S108: Bar chart of model accuracy rankings on the Chief Physician Neurology examination, testing highest-level expertise in neurological disorders including stroke, epilepsy, movement disorders, neurodegenerative diseases, and neuromuscular conditions. The complex diagnostic reasoning required in neurology favors reasoning-enhanced architectures, with DeepSeek-R1 and QWQ models achieving superior performance through explicit chain-of-thought processes. Top performers: DeepSeek-R1-70b (72.0%), DeepSeek-R1-32b (69.5%), DeepSeek-R1-14b (65.5%).

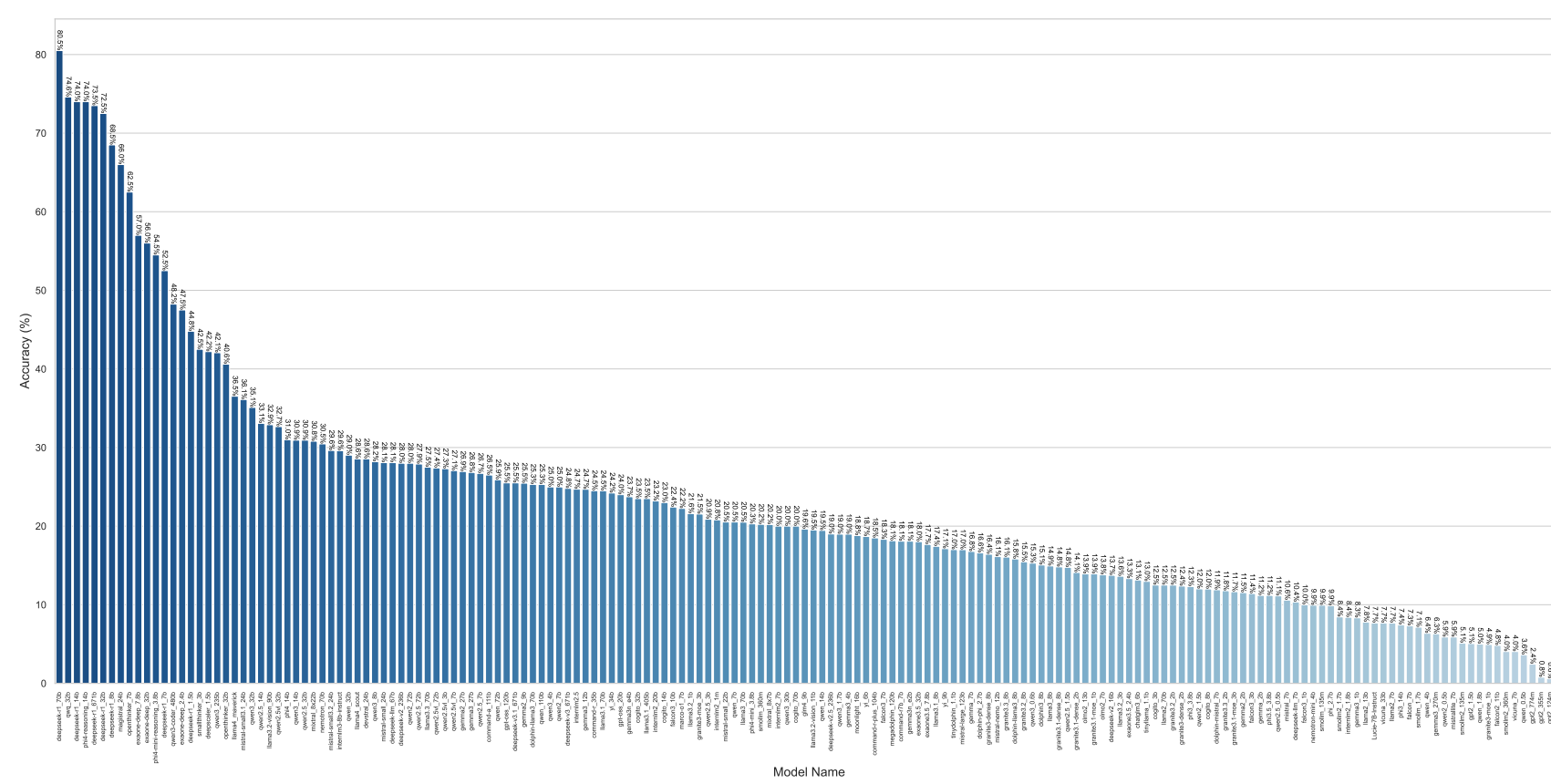

Figure S109: Bar chart of model accuracy rankings on the Chief Physician Psychiatry examination, assessing highest-level expertise in psychiatric disorders, psychopharmacology, psychotherapy, and forensic psychiatry. This specialty requires nuanced understanding of mental health presentations and treatment approaches, where top reasoning models demonstrate strong performance in navigating complex diagnostic criteria and therapeutic decision-making. Top performers: DeepSeek-R1-70b (80.5%), QWQ-32b (74.6%), DeepSeek-R1-14b (74.0%).

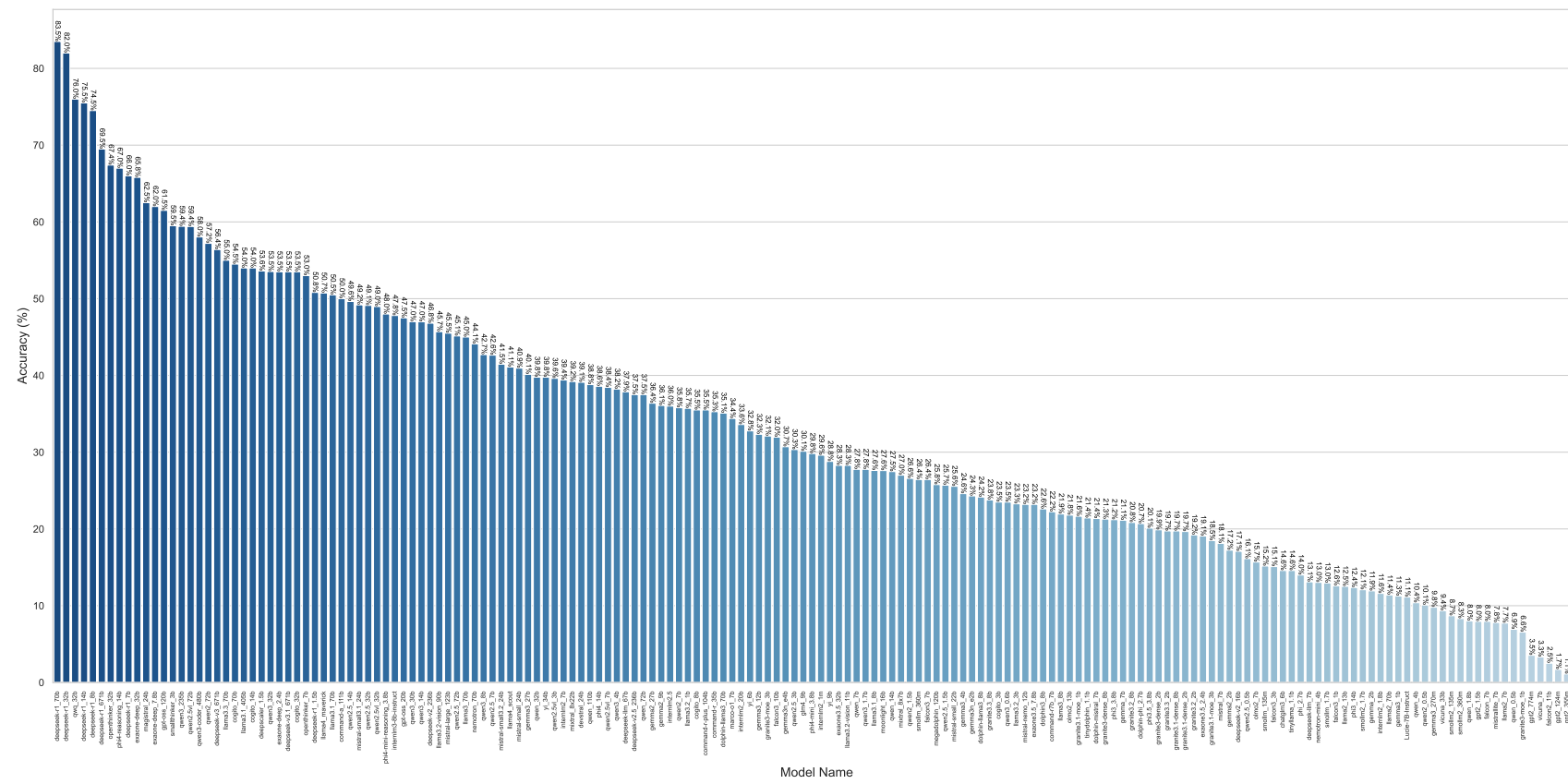

Figure S110: Bar chart of model accuracy rankings on the Chief Physician Geriatrics examination, evaluating expertise in elderly patient care including polypharmacy management, frailty assessment, geriatric syndromes, and end-of-life care. Reasoning-enhanced models excel at the multidimensional clinical reasoning required for geriatric medicine, achieving top accuracy through comprehensive integration of multiple comorbidities and treatment considerations. Top performers: DeepSeek-R1-70b (83.5%), DeepSeek-R1-32b (82.0%), QWQ-32b (76.0%).

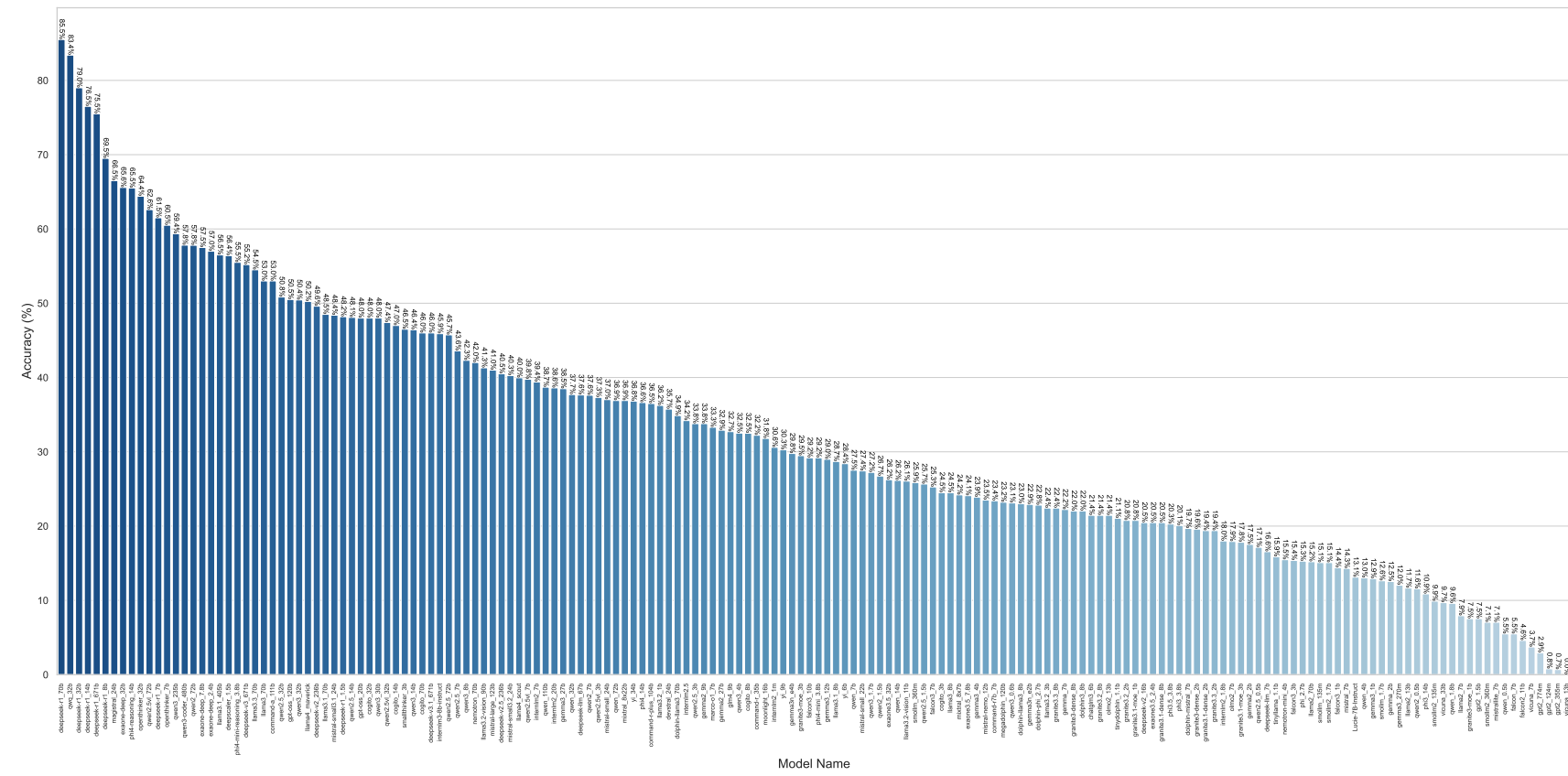

Figure S111: Bar chart of model accuracy rankings on the Chief Physician Occupational Medicine examination, testing highest-level expertise in occupational diseases, workplace hazard assessment, occupational toxicology, and occupational health program management. Reasoning-enhanced models achieve top performance through systematic analysis of exposure-disease relationships and evidence-based preventive medicine principles. Top performers: DeepSeek-R1-70b (85.5%), QWQ-32b (83.4%), DeepSeek-R1-32b (79.0%).

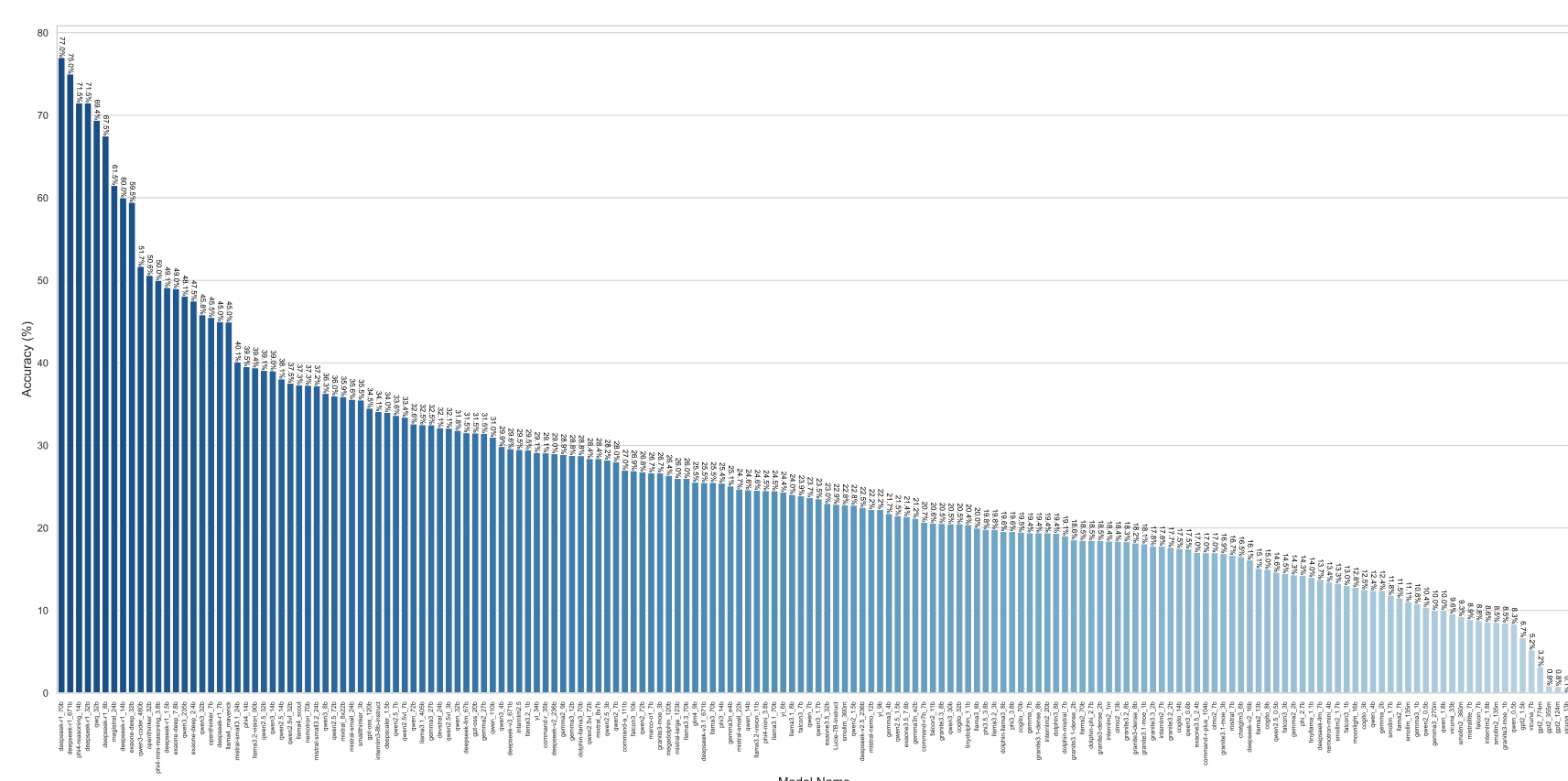

Figure S112: Bar chart of model accuracy rankings on the Chief Physician Surgical Oncology examination, assessing highest-level expertise in surgical management of malignancies, oncological resection techniques, multidisciplinary cancer care, and surgical decision-making for complex tumor presentations. Top reasoning models demonstrate exceptional accuracy in this demanding specialty requiring integration of surgical technique with oncological principles. Top performers: DeepSeek-R1-70b (77.0%), DeepSeek-R1-671b (75.0%), Phi4-Reasoning-14b (71.5%).

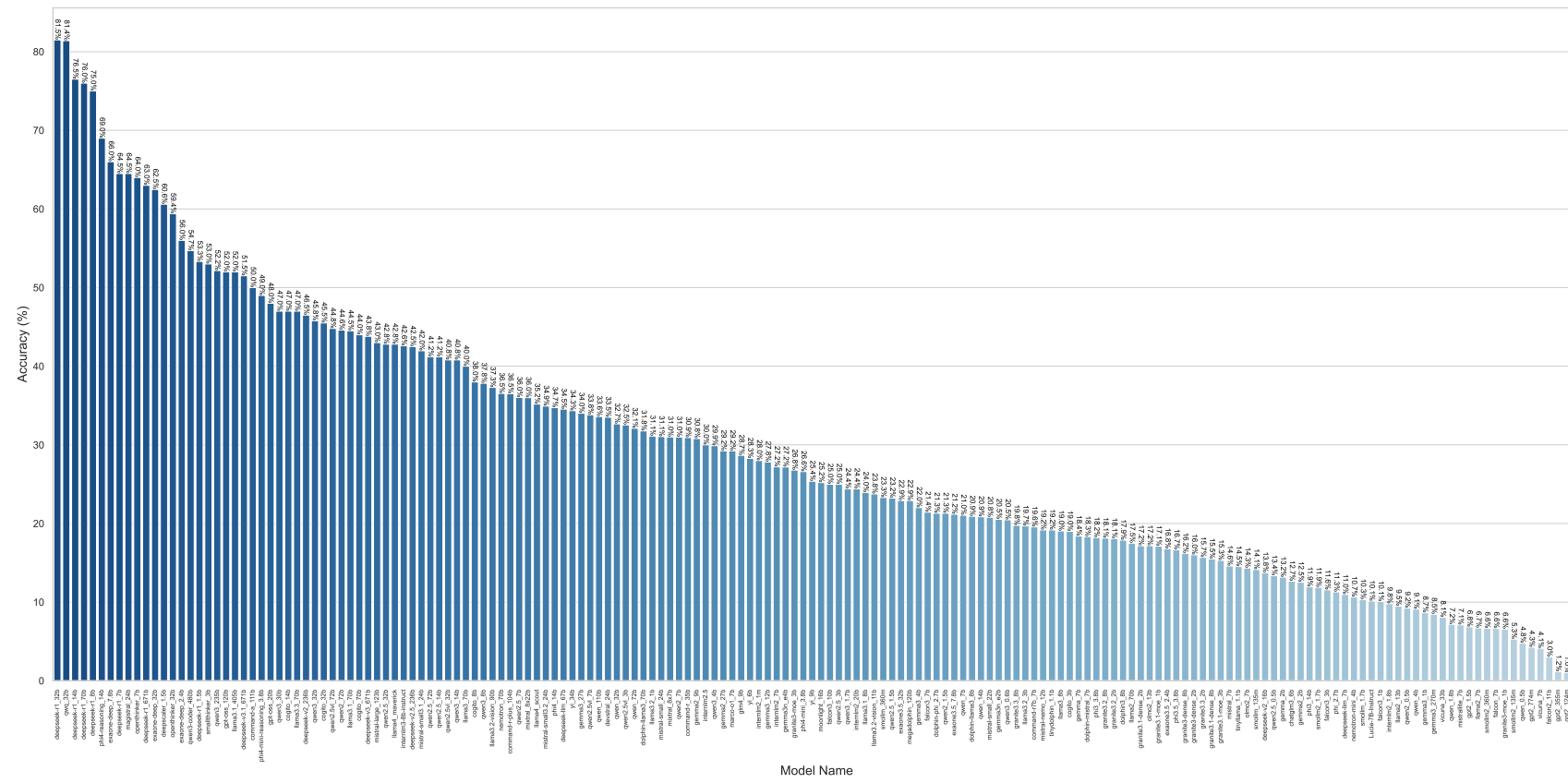

Figure S113: Bar chart of model accuracy rankings on the Chief Physician Nutrition and Food Hygiene examination, testing highest-level expertise in nutritional science, food safety, dietary intervention, and public health nutrition policy. This specialty requires integration of biochemistry, epidemiology, and regulatory knowledge, where reasoning-enhanced models demonstrate robust performance through comprehensive analytical capabilities. Top performers: DeepSeek-R1-32b (81.5%), QWQ-32b (81.4%), DeepSeek-R1-14b (76.5%).

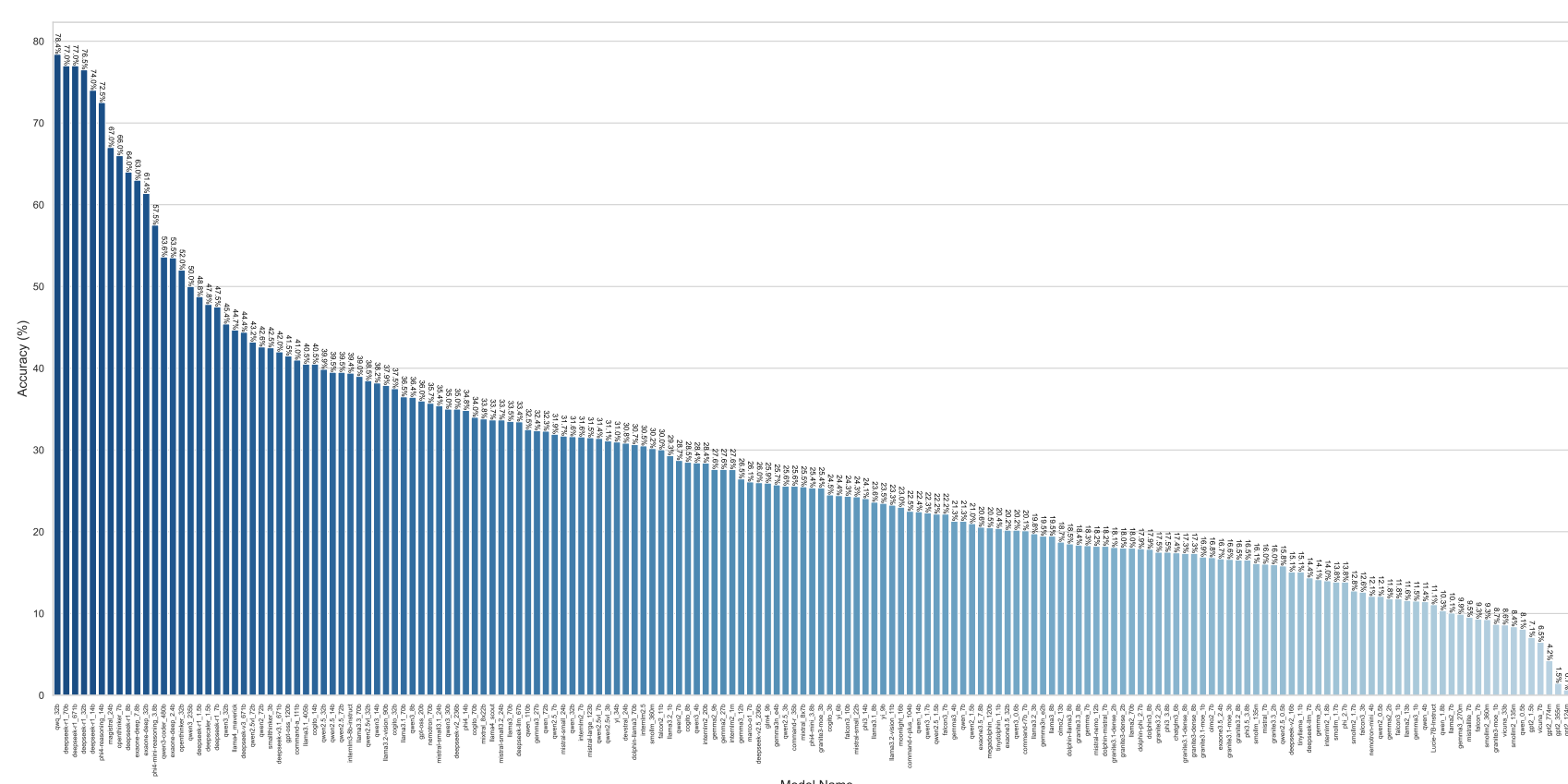

Figure S114: Bar chart of model accuracy rankings on the Chief Physician Ultrasound Medicine examination, evaluating highest-level expertise in ultrasound physics, sonographic anatomy, and diagnostic applications across obstetrics, cardiology, abdominal imaging, and vascular assessment. Leading reasoning-enhanced models achieve top accuracy through sophisticated integration of imaging principles and clinical correlation essential for ultrasound diagnosis. Top performers: QWQ-32b (78.4%), DeepSeek-R1-70b (77.0%), DeepSeek-R1-671b (77.0%).

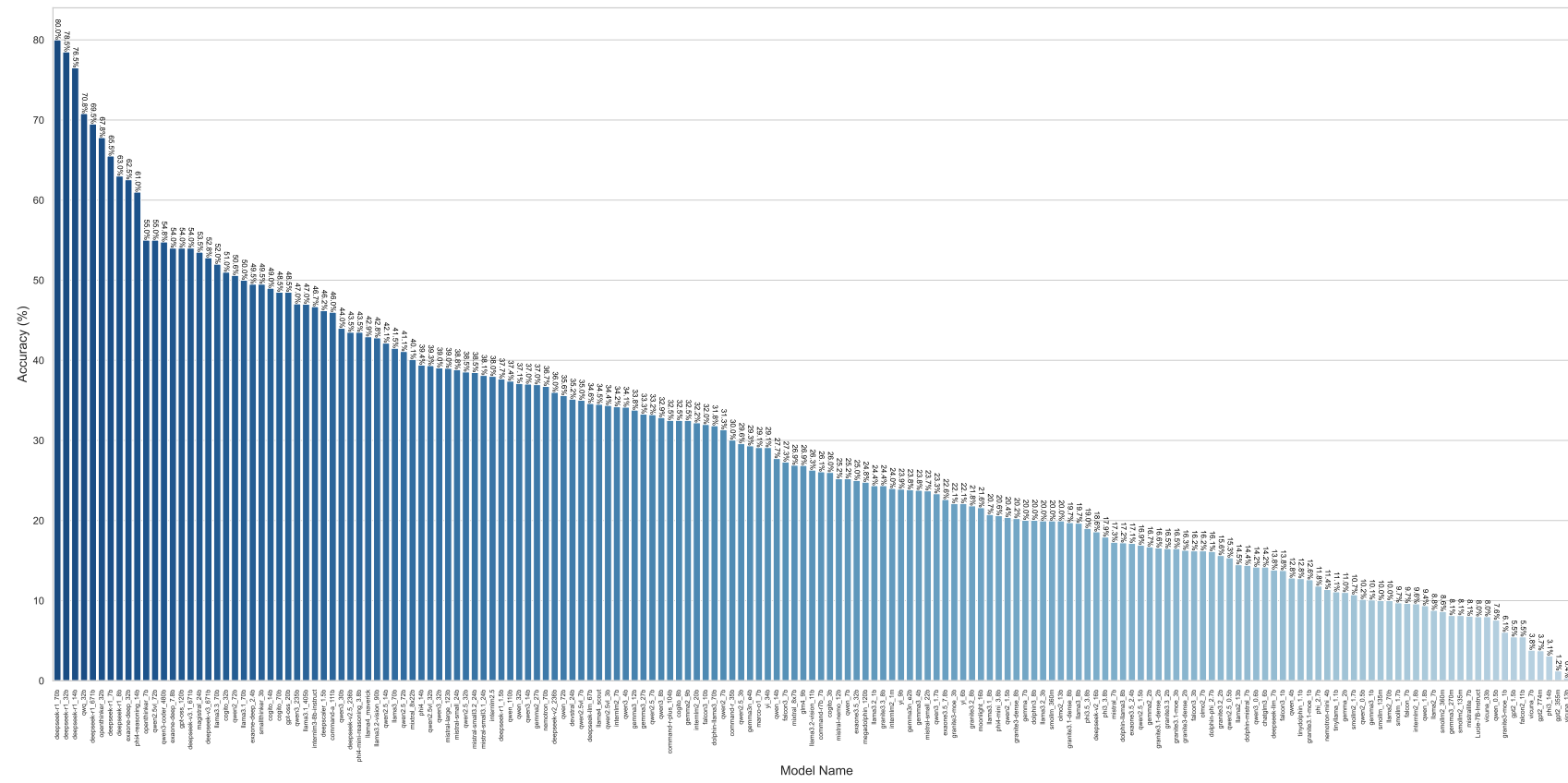

Figure S115: Bar chart of model accuracy rankings on the Chief Physician Critical Care Medicine examination, assessing highest-level expertise in ICU management, mechanical ventilation, hemodynamic monitoring, sepsis treatment, and multi-organ failure management. This high-acuity specialty demands rapid clinical decision-making under complex conditions, where reasoning-enhanced models (DeepSeek-R1, QWQ) demonstrate superior performance through explicit reasoning chains. Top performers: DeepSeek-R1-70b (80.0%), DeepSeek-R1-32b (78.5%), DeepSeek-R1-14b (76.5%).

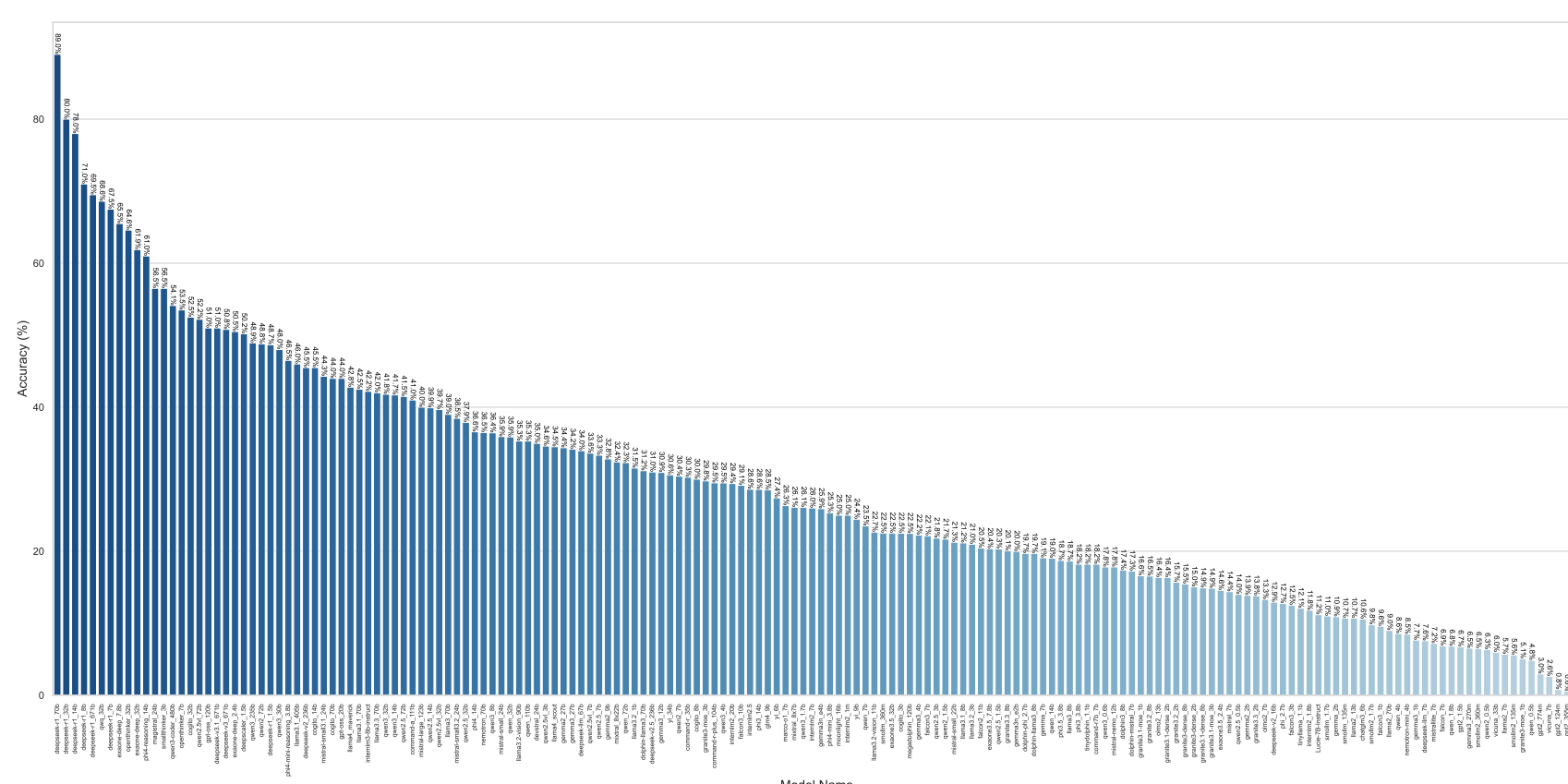

Figure S116: Bar chart of model accuracy rankings on the Chief Physician Anesthesiology examination, testing highest-level expertise in anesthetic pharmacology, airway management, regional anesthesia, perioperative care, and anesthesia for high-risk patients. Top reasoning models achieve exceptional accuracy through sophisticated understanding of physiological monitoring, drug interactions, and intraoperative decision-making required for safe anesthesia practice. Top performers: DeepSeek-R1-70b (89.0%), DeepSeek-R1-32b (80.0%), DeepSeek-R1-14b (78.0%).

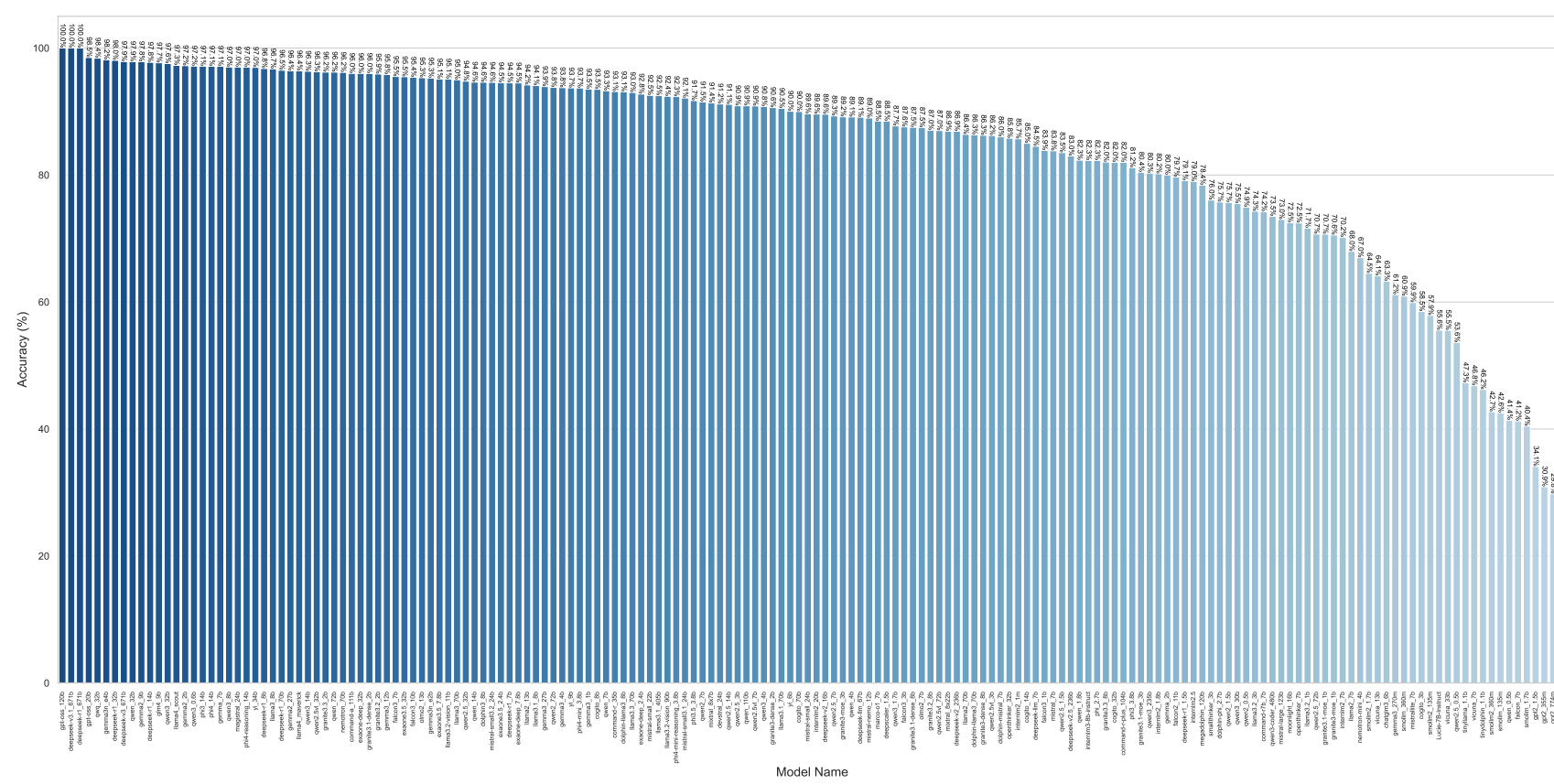

Figure S117: Bar chart of model accuracy rankings on the Health Classification Dataset, a specialized benchmark for medical entity classification and health-related categorization tasks. This dataset evaluates models' ability to accurately classify medical concepts, symptoms, and conditions into appropriate categories, where reasoning-enhanced models demonstrate consistent advantages through structured analytical approaches to classification problems. Top performers: GPT-OSS-120b (100.0%), DeepSeek-V3.1-671b (100.0%), DeepSeek-R1-671b (100.0%).
